# Evidence for a Unifying Ni^I^/Ni^III^ Mechanism in Light-Mediated Cross-Coupling Catalysis

**DOI:** 10.1021/jacs.4c16050

**Published:** 2025-04-11

**Authors:** Lucia Anghileri, Haralds Baunis, Aleksander R. Bena, Christos Giannoudis, John H. Burke, Susanne Reischauer, Christoph Merschjann, Rachel F. Wallick, Tarek Al Said, Callum E. Adams, Gianluca Simionato, Sergey Kovalenko, Luca Dell’Amico, Renske M. van der Veen, Bartholomäus Pieber

**Affiliations:** † Institute of Science and Technology Austria (ISTA), Am Campus 1, Klosterneuburg 3400, Austria; ‡ Department of Biomolecular Systems, Max-Planck-Institute of Colloids and Interfaces (MPICI), Am Mühlenberg 1, Potsdam 14476, Germany; § Department of Chemistry and Biochemistry, Freie Universität Berlin, Arnimallee 22, Berlin 14195, Germany; ∥ Department of Chemistry, University of Illinois Urbana−Champaign, Urbana, Illinois 61801, United States; ⊥ Helmholtz Zentrum Berlin für Materialien und Energie GmbH, Hahn-Meitner-Platz 1, Berlin 14109, Germany; # Department of Chemical Sciences, University of Padova, Via Francesco Marzolo 1, Padova 35131, Italy; ∇ Department of Chemistry, Humboldt-Universität zu Berlin, Brook-Taylor-Str. 2, Berlin 12489, Germany; ○ Institute of Optics and Atomic Physics, Technische Universität Berlin, Hardenbergstraße 36, Berlin 10623, Germany

## Abstract

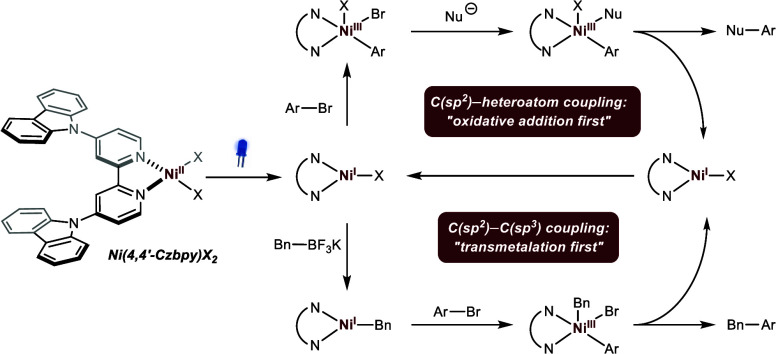

Advances in nickel catalysis have significantly broadened the synthetic chemists’ toolbox, particularly through methodologies leveraging paramagnetic nickel species via photoredox catalysis or electrochemistry. Key to these reactions is the oxidation state modulation of nickel via single-electron transfer events. Recent mechanistic studies indicate that C(sp^2^)–heteroatom bond formations proceed through Ni^I^/Ni^III^ cycles. Related C(sp^2^)–C(sp^3^) cross-couplings operate via the photocatalytic generation of C-centered radicals and a catalytic cycle that involves Ni^0^, Ni^I^, and Ni^III^ species. Here, we show that light-mediated nickel-catalyzed C(sp^2^)–C(sp^3^) bond formations can be carried out without using exogenous photoredox catalysts but with a photoactive ligand. In a pursuit of expanding the scope of C(sp^2^)–heteroatom couplings using donor–acceptor ligands, we identified a photoactive nickel complex capable of catalyzing cross-couplings between aryl halides and benzyltrifluoroborate salts. Mechanistic investigations provide evidence that transmetalation between a photochemically generated Ni^I^ species and the organoboron compound is the key catalytic step in a Ni^I^/Ni^III^ catalytic cycle under these conditions.

## Introduction

**Figure 1:**
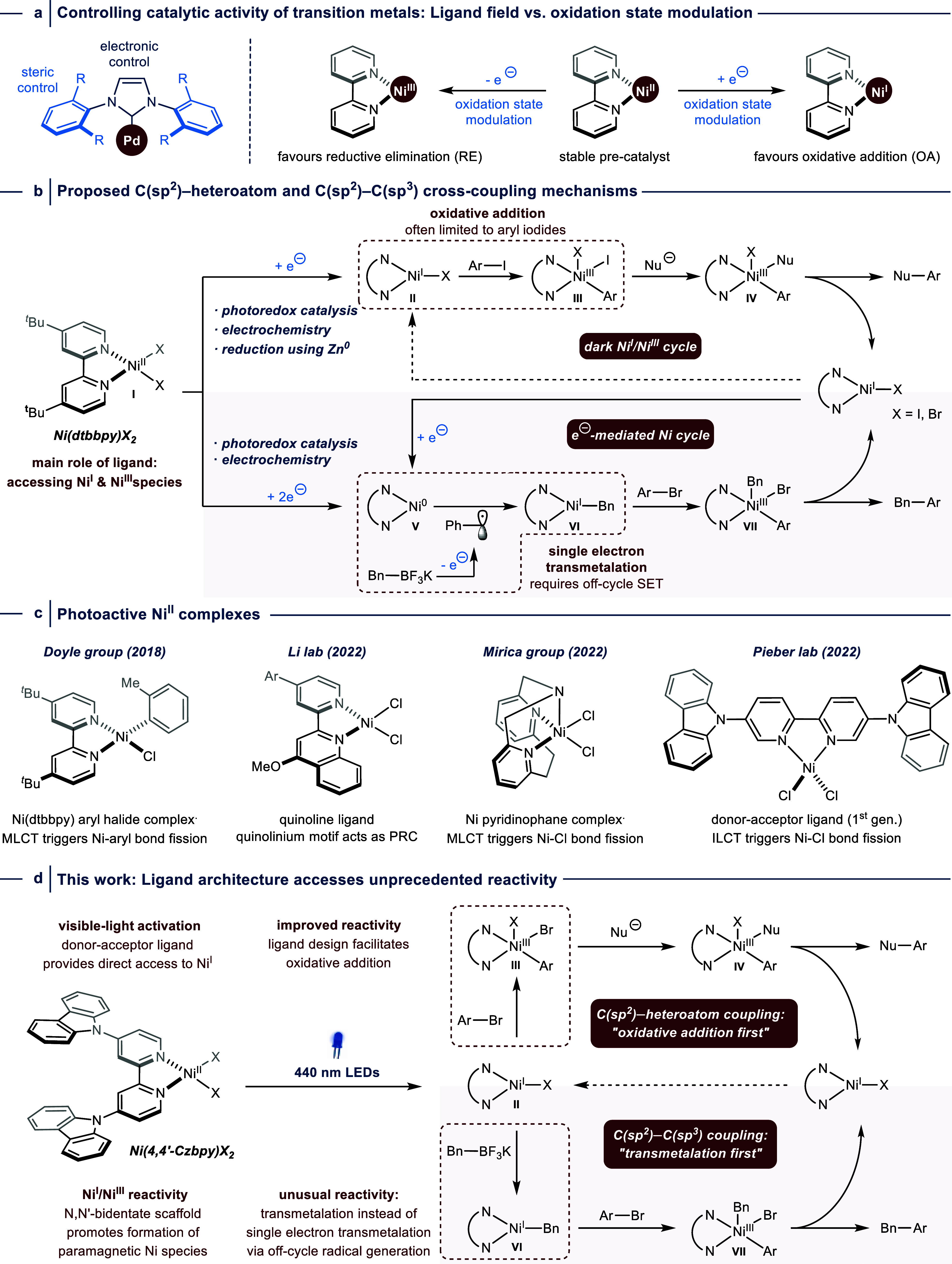
Nickel bipyridine complexes as catalysts for cross-couplings. (a) Controlling catalytic activity through the ligand field (left) or by modulating the metal’s oxidation state (right). (b) Proposed mechanisms of catalytic C(sp^2^)–heterotaom and C(sp^2^)–C(sp^3^) cross-couplings through oxidation state modulation of Ni(dtbbpy)X_2_. (c) Photoactive Ni^II^ pre-catalysts. (d) This work: studies on light-mediated cross-couplings with a photoactive nickel complex provide evidence that C(sp^2^)–heterotaom and C(sp^2^)–C(sp^3^) bond formations can both operate through Ni^I^/Ni^III^ manifolds.

The catalytic activity of palladium complexes for cross-coupling reactions can be fine-tuned through ligand modifications. This reactivity control has unlocked a broad substrate scope, low catalyst loadings, and mild reaction conditions (Figure 1a).^1−4^ Over the past decade, the integration of nickel catalysis with single-electron transfer (SET) reactivity has emerged as a pivotal platform for alternative and complementary cross-couplings, operating via a fundamentally distinct strategy.^5−10^ Instead of modulating the metal’s ligand field, these catalytic reactions are orchestrated by manipulating the oxidation state of nickel. This provides several plausible mechanisms that are being actively studied and debated. For example, the originally proposed mechanisms of C(sp^2^)–heteroatom cross-couplings were recently revised by showing that these reactions proceed through a “dark” Ni^I^/Ni^III^ cycle initiated by single-electron reduction of a Ni^II^ precatalyst (**I**) employing photoredox catalysis (PRC),^11−14^ cathodic reduction,^15^ or zinc (Figure 1b).^16,17^ The mechanism of C(sp^2^)–C(sp^3^) cross-couplings between aryl halides and radical precursors, such as alkyltrifluoroborates, is arguably more complex and was proposed to require several SET events facilitated by photoredox catalysis^7,18^ or electrochemistry.^19^ In these scenarios, single-electron reductions are assumed to produce a catalytically active Ni^0^ species (**V**) capable of trapping an alkyl radical, which is generated through an off-cycle single-electron oxidation of the nucleophile (single-electron transmetalation). The resulting Ni^I^ intermediate (**VI**) undergoes oxidative addition (OA) with the aryl halide, followed by reductive elimination to afford the desired product. A single-electron reduction of the resulting Ni^I^ species closes the nickel cycle. Typically, these C(sp^2^)–heteroatom and C(sp^2^)–C(sp^3^) cross-coupling protocols employ Ni^II^ salts in conjunction with 4,4′-di-*tert*-butyl-2,2′-bipyridine (dtbbpy) as a privileged ligand. The primary role of the *N,N*′-bidentate motif is to promote the formation of the key paramagnetic nickel species.^20^ Notably, recent studies indicated that alterations in ligand structure influence oxidative addition on Ni^I^ through steric and electronic effects.^21,22^

Photoactive nickel complexes obviate the need for exogenous photocatalysts, electrochemical setups, or the addition of chemical reductants in C(sp^2^)–heteroatom cross-couplings that proceed through the Ni^I^/Ni^III^ manifold (Figure 1c). Seminal studies by Doyle and colleagues have demonstrated that Ni^II^(dtbbpy) aryl halide complexes produce Ni^I^ species upon irradiation with light.^23,24^ Direct excitation generates a metal-to-ligand charge transfer (MLCT) state that transitions to a triplet metal-centered *d–d* state^23^ or a ligand-to-metal charge transfer (LMCT) state,^25,26^ resulting in homolysis of the Ni^II^–aryl bond. These complexes have been applied as effective catalysts for C–O and C–N cross-couplings using 390 nm irradiation.^27,28^ Li and coworkers followed a different strategy toward photoactive ligands by integrating a quinolinium photoredox catalyst^29^ into the bipyridine ligand scaffold.^30^ In combination with NiCl_2_ and a 390 nm light source, this photoredox-active ligand facilitates several transformations, including C(sp^2^)–C(sp^3^) couplings between aryl halides and alkyltrifluoroborates. Seminal work by Nocera and coworkers showed that halogen photoelimination from nickel complexes is possible.^31,32^ The Mirica group has demonstrated that a similar activation mechanism triggers Ni^II^–Cl bond fission in the case of a Ni(pyridinophane)Cl_2_ complex to promote C–O bond formations using purple LEDs (390 nm).^33^ In the same year, we demonstrated that a nickel complex featuring a donor–acceptor (D-A) ligand harnesses lower energy visible light (440 nm) through an intraligand charge transfer (ILCT) transition.^34^ This accessed the Ni^I^/Ni^III^ manifold for C(sp^2^)–heteroatom bond formations between aryl iodides and S–, N–, and O–nucleophiles via excited-state properties that depend solely on the electronics and structure of the ligand scaffold.

Here, we show that modifications of our first-generation photoactive ligand^34^ can fine-tune nickel’s catalytic activity to expand the scope of C(sp^2^)–heteroatom couplings. Furthermore, we discovered that this ligand modification enables light-mediated C(sp^2^)–C(sp^3^) cross-couplings between primary benzyltrifluoroborate salts and aryl halides without an exogenous photoredox catalyst. Mechanistic studies provide evidence that transmetalation between a photochemically generated Ni^I^ species and the organoboron nucleophile is the key catalytic step in a Ni^I^/Ni^III^ catalytic cycle.

## Results and Discussion

### Ligand Design, Characterization, and Evaluation

**Figure 2:**
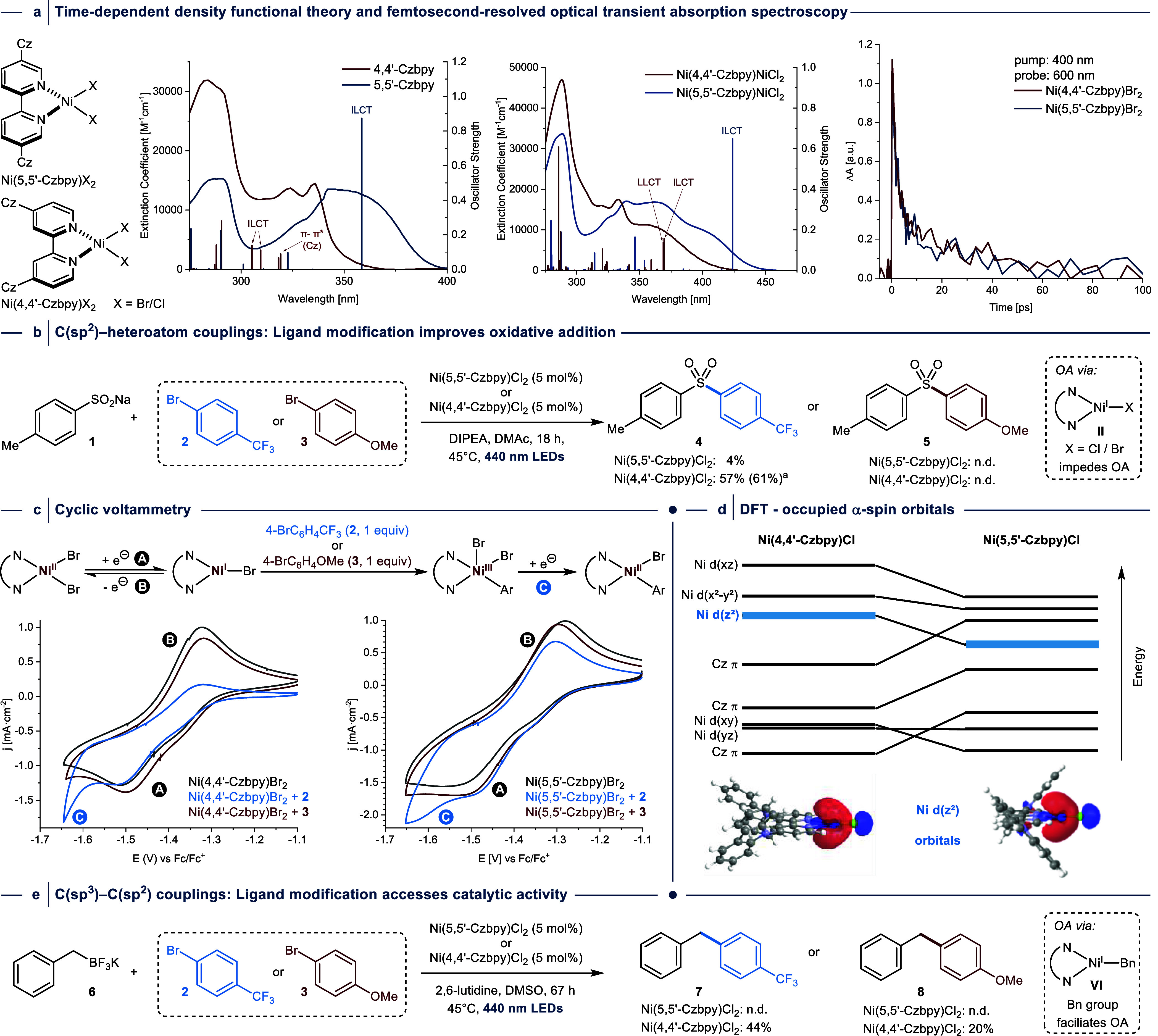
Structural modifications of donor–acceptor ligand impacts catalytic activity in light-mediated nickel-catalyzed cross-couplings. (a) Static absorption spectra of ligands (left) and NiCl_2_ complexes (middle) in DMSO. TD-DFT stick spectra are superimposed, showing the ILCT character of the lowest-energy absorption bands. Right: kinetic traces (OTA) of NiBr_2_ complexes (pump 400 nm) at a probe wavelength of 600 nm, showing a pronounced decrease in excited-state lifetime compared to the free ligands (see Figure S17). (b) 4,4′-Czbpy outperforms its regioisomer (5,5′-Czbpy) as ligand for light-mediated nickel catalyzed C–S couplings. (c) CV studies (1 M Ni(4,4′-Czbpy)Br_2_ with addition of **2** or **3** in DMAc, 0.1 M Bu_4_NBr as supporting electrolyte at 100 mV·s^–1^) show that use of 4,4′-Czbpy instead of 5,5′-Czbpy results in higher OA rates between **2** and an electrochemically generated Ni^I^ species. (d) Comparison of DFT orbital energies indicate that the *3d(z^2^)* orbital of Ni(4,4′-Czbpy)Cl is destabilized and reactive toward OA. CAM-B3LYP-GD3/6-311+G(d,p). (e) The modified D–A ligand enables C(sp^2^)–C(sp^3^) cross-couplings between benzyltrifluoroborates and aryl bromides. ^a^Yield in brackets refers to reaction carried out using 1 mol % of Ni(4,4′-Czbpy)Cl_2_. Yields were determined by ^1^H NMR using 1,3,5-trimethoxybenzene as an internal standard.

Our research endeavors began with the objective of expanding the applicability of our first-generation donor–acceptor complex Ni(5,5′-Czbpy)X_2_ (X = Cl or Br), which was confined to coupling aryl iodides with nucleophiles.^34^ We proposed that relocating the electron-donating carbazole motifs to the 4,4′-position of bipyridine^35^ could yield a nickel–ILCT complex with improved catalytic activity due to increased electron density on the Ni^I^ intermediate, which was expected to facilitate OA. To test this hypothesis, we synthesized 4,4′-Czbpy and compared its photophysical properties with its regioisomer 5,5′-Czbpy. The ligands have different static UV/visible absorption spectra (Figure 2a, left). While the low-energy band of 5,5′-Czbpy peaks at 350 nm, the band of 4,4′-Czbpy is blue-shifted and overlaps with the vibronically resolved carbazole-centered π–π* transition (335 nm, Figure S18). Time-dependent density functional theory (DFT) calculations confirm that these bands belong to absorption into states with ILCT character (sticks in Figure 2a, left; Figures S19–S37). In the case of 4,4′-Czbpy, the Cz-centered π–π* transitions also contribute to the absorption. Both ligands exhibit a redshift of the ILCT band upon NiX_2_ complexation, with an absorption onset in the visible region of the electromagnetic spectrum (Figure 2a, middle). In the case of Ni(4,4′-Czbpy)X_2_, the lowest-energy band also contains some ligand-to-ligand charge-transfer (LLCT) character (Figures S24, S26, and S27). Pump–probe femtosecond-resolved optical transient absorption (OTA) spectroscopy experiments were conducted on both the ligands (345 nm excitation) and the complexes (400 nm excitation). The complexes show transient spectra similar to those of the ligands (Figures S16, S28, and S29), but with dramatically reduced lifetimes of ∼20 ps (Figure 2a, right, Figure S17). This confirms that the lowest excited state of both ligands is quenched by NiX_2_, which likely occurs by a decay into a metal-centered *d–d* state manifold.^34^

These *d–d* states have antibonding character along the nickel–halide bonds, signifying their propensity for Ni^II^–halide bond homolysis and the formation of catalytically active Ni^I^ species.^23,33,34^ Alternatively, dissociative states that cross higher-energy charge-transfer states could be involved, as has been demonstrated for Ni^II^–aryl bond homolysis.^25^ The formation of the paramagnetic nickel species upon irradiation was indirectly confirmed by an electron paramagnetic resonance (EPR) spin-trapping experiment (Figure S39).^34^ Overall, these results show that nickel complexes of both ligands obey similar excited-state dynamics, but the difference in spectral band positions indicates that the electronic structure is modulated by 4,4′- versus 5,5′-carbazole functionalization of bpy, which ultimately impacts the electronic structure of the Ni center (*vide infra*).

The proposed fine-tuning of Ni^I^ reactivity toward OA through electronic control via the ligand field was demonstrated in a model C(sp^2^)–heteroatom cross-coupling (Figure 2b). Using 440 nm LEDs, 4,4′-Czbpy served as a suitable ligand for nickel to catalyze the coupling of sodium *p*-toluenesulfinate (**1**) with 4-bromobenzotrifluoride (**2**). The catalyst loading could be reduced from 5 to 1 mol % resulting in a similar cross-coupling yield. In contrast, only traces of the desired product were obtained when 5,5′-Czbpy was employed. Both ligands proved ineffective in coupling a more challenging electron-rich aryl bromide (**3**).

Mechanistic investigations using cyclic voltammetry (CV) corroborated these findings (Figure 2c). Reversible Ni^I^/Ni^II^ couples were obtained in the case of both carbazole-substituted bipyridine ligands. Previous studies necessitated the installation of methyl groups adjacent to the nitrogen atoms of bipyridine ligands to obtain interpretable CVs.^21^ This was rationalized by steric shielding of the metal center, which avoids speciation and disproportionation processes. In contrast, the donor–acceptor design allows to study the Ni^I^/Ni^II^ couple without ligand modifications in the 6,6′-position, indicating that Cz-substituted bpy ligands stabilize the low-valent paramagnetic nickel species through electronic or distal steric effects.^36^ This enabled us to link the electrochemical generation of the Ni^I^ species (E-step, peak **A**) with its chemical consumption (C-step) upon reaction with aryl halides (EC mechanism).^21,37^ A decrease in reversibility, indicated by a lowering of the intensity of the return peak (**B**) and the generation of a new species (signified by **C**), which was previously assigned to be a Ni^II^(aryl) species (formed by reduction of the Ni^III^ OA complex),^21^ signifies effective OA on the CV time scale. These features enable qualitative comparison of the reactivity of different aryl halides and nickel complexes.^21^ Consequently, and given that the halide identity of Ni^I^ bipyridine species was shown to have no significant effect on OA,^22^ CV studies were conducted using ligated NiBr_2_ instead of NiCl_2_ salts (to avoid the presence of multiple halide species potentially affecting the analysis). The electroanalytical approach was first validated using 4-iodobenzotrifluoride, which confirmed that both ligands generate Ni^I^ complexes that undergo facile OA with the aryl iodide (Figures S48 and S52). In agreement with observations from the model reaction, the CV of electrochemically generated Ni(4,4′-Czbpy)Br in the presence of electron-poor aryl bromide (**2**) revealed a notable loss in reversibility. This effect was significantly less pronounced with 5,5′-Czbpy. The electron-rich aryl bromide (**3**), which failed to yield the desired C–S coupling product with both ligands, induced no substantial alterations in the reversibility of the Ni^I^/Ni^II^ couples.

DFT calculations were employed to investigate the electronic structure of the reactive Ni^I^ species. Previous studies established the significance of the *3d(z*^*2*^*)* orbital in the OA of aryl halides to Ni^I^ complexes, with electron-donating substituents enhancing the rate of this reaction by destabilizing Ni orbitals (including the *3d*(*z*^*2*^*)* orbital).^22^ The isomeric nature of Ni(4,4′-Czbpy)Cl and Ni(5,5′-Czbpy)Cl allows for a direct comparison of their orbital energies through Kohn–Sham DFT (Figure 2d). The substitution pattern of 4,4′-Czbpy results in the destabilization of the *3d* orbitals of the respective Ni^I^–Cl complex compared to its regioisomer Ni(5,5′-Czbpy)Cl, leading to a higher energy of the *3d(z*^*2*^*)* orbital that is responsible for the observed difference in OA efficacy.

After identifying that the modification of a photoactive ILCT ligand expands the scope of C(sp^2^)–heteroatom couplings, we wondered whether these structural changes impact the (photo)catalytic activity of the corresponding nickel complex regarding C(sp^2^)–C(sp^3^) couplings. Previously, we demonstrated that Ni(5,5′-Czbpy)Cl_2_ has moderate catalytic activity toward the light-mediated coupling between an aryl iodide and an α-silylamine^34^ but failed to catalyze the coupling of aryl halides with potassium benzyltrifluoroborate (**6**) (Figure 2e).^7,18,19,38^ Employing 4,4′-Czbpy as a ligand overcomes this limitation: the second-generation ILCT complex facilitated C(sp^2^)–C(sp^3^) bond formation of (**6**) with both an electron-poor (**2**) and an electron-rich aryl bromide (**3**). The difference in aryl halide reactivity, when compared to C(sp^2^)–heteroatom couplings (OA of Ni^I^ halide into aryl bromide), is in agreement with observations made in dual photoredox/nickel catalysis and suggests that the electron-rich Ni^I^ benzyl intermediate (**VI**) rather than a Ni^I^ halide intermediate (**II**) undergoes oxidative addition.^39,40^

### Scope and Limitations

**Figure 3:**
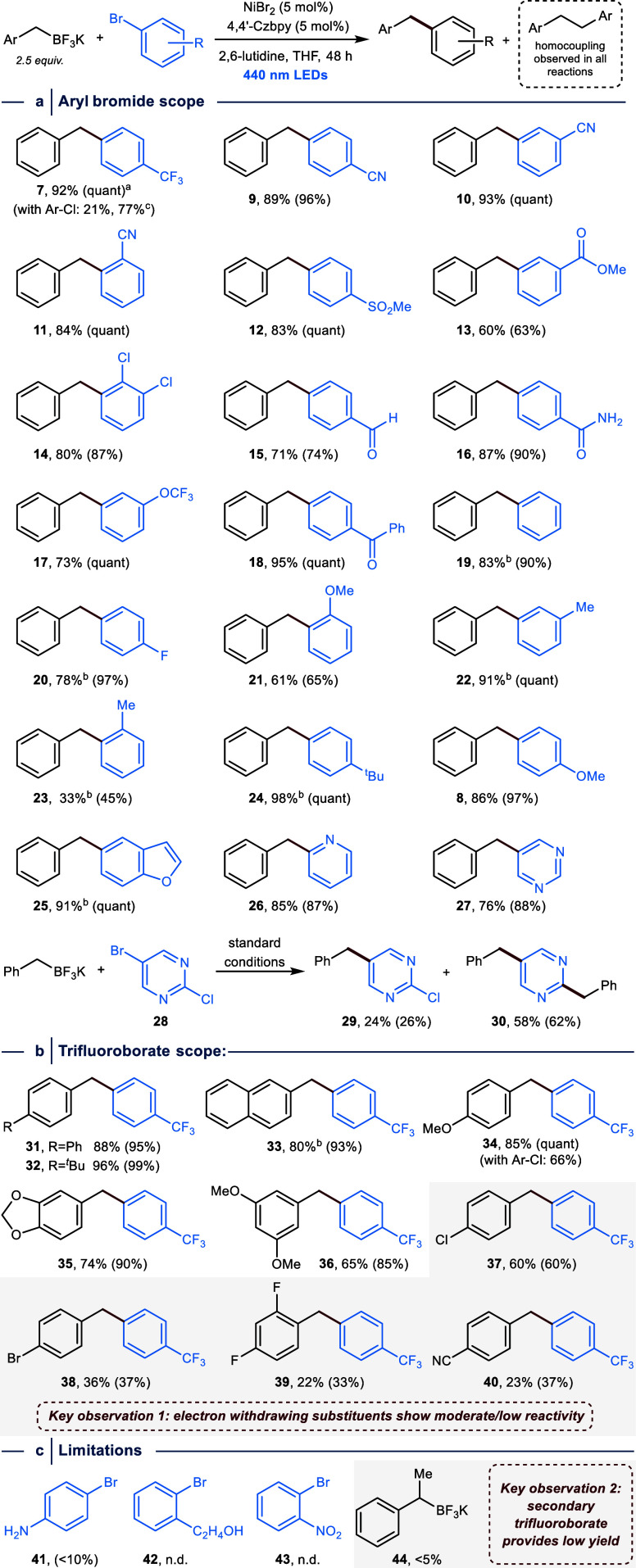
Scope of light-mediated C(sp^2^)–C(sp^3^) cross-couplings catalyzed by Ni(4,4′-Czbpy)Br_2_. (a) Aryl bromide scope. (b) Benzyltrifluoroborate scope. (c) Limitations. Isolated yields are reported. ^a^NMR yields in brackets were determined by ^1^H NMR using 1,3,5-trimethoxybenzene as internal standard. ^b^Isolated products contain 6–15% of the inseparable 1,2-diarylethane homocoupling side-product. ^c^Reaction was carried out at 60 °C. n.d. = not detected.

Optimizing reaction parameters in the coupling between **6** and **2** (Tables S6–S11) provided conditions that allowed for the quantitative formation of the desired product (**7**) (Figure 3). Notably, no reaction was observed in the absence of light, a nickel source, 4,4′-Czbpy, or when 4,4′-dimethoxy-2,2′-bipyridine or 4,4′-dimethylamino-2,2′-bipyridine were used instead of 4,4′-Czbpy (Table S12). A reaction using the respective aryl chloride resulted in 21% of the coupling product under the standard conditions. The low reactivity of this aryl chloride can be addressed using slightly elevated temperatures (60 °C instead of 45 °C; see Supporting Information for details), which provided the desired coupling product in 77%. Next, the catalytic protocol was evaluated using several aryl bromides and benzyltrifluoroborate salts (Figure 3). Substrates were selected to enable comparison with the scope of the seminal protocol using dual nickel/photoredox catalysis,^38^ to study whether the two catalytic systems operate through similar or different mechanisms. In all reactions, 1,2-diarylethane side-products were detected in the crude NMR spectrum, suggesting that C-centered radicals are formed during catalysis. High levels of versatility and functional group tolerance were observed regarding the (hetero)aryl bromide, and the corresponding coupling products were obtained in good to excellent yields (**7–27**) (Figure 3a). A reaction using 5-bromo-2-chloropyrimidine (**28**) resulted in a mixture of the mono-(**29**) and dibenzylated product (**30**).

Comparatively, the nature of the benzyltrifluoroborate component significantly affected the yield of the cross-coupling protocol (Figure 3b). Electron-rich benzyltrifluoroborates reacted smoothly and resulted in high cross-coupling yields (**31–36**). Notably, the coupling of 4-methoxybenzyltrifluoroborate (**34**) with an aryl chloride furnished the desired cross-coupling product in good yield (66%) without increasing the reaction temperature (*vide supra*). In contrast to the dual photoredox/nickel catalysis approach from the Molander group,^38^ our catalytic protocol resulted in significantly lower yields when electron-deficient trifluoroborates were used as nucleophiles (**37–40**). Moreover, we were surprised to find that the substrate limitations of Ni(4,4′-Czbpy)Br_2_ catalysis included the use of (α-methyl)benzyltrifluoroborate (**44**) (Figure 3c),^41^ a substrate that can be smoothly coupled in protocols that apply a nickel bipyridine complex in combination with a photoredox catalyst.^18,38,39,42^ These differences in the scope and efficacy were key observations that suggested that light-mediated Ni(4,4′-Czbpy)Br_2_ catalysis operates through a fundamentally different mechanism than methods that employ dual photoredox/nickel catalysis.

### Mechanistic Investigations

**Figure 4:**
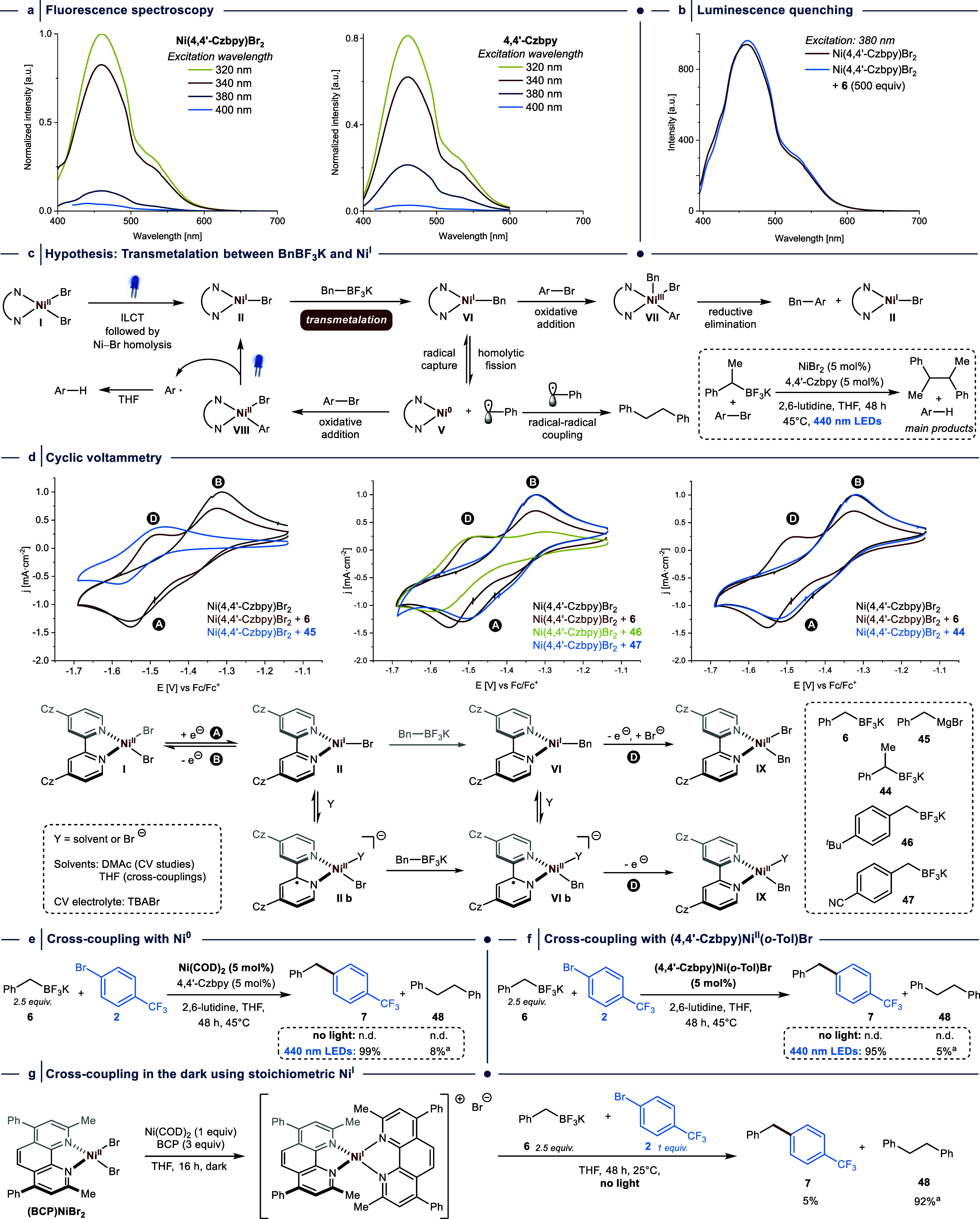
Mechanistic investigations. (a) Steady-state fluorescence spectroscopy of Ni(4,4′-Czbpy)Br_2_ and 4,4′-Czbpy are virtually identical and show no significant emission when irradiated >380 nm. (b) Luminescence quenching of Ni(4,4′-Czbpy)Br_2_ is not observed in the presence of BnBF_3_K (**6**). (c) Proposed mechanism. (d) CV analysis (1 mM of Ni(4,4′-Czbpy)Br_2_ with addition of **6** in DMAc, 0.1 M Bu_4_NBr as supporting electrolyte at 100 mV·s^–1^) confirms transmetalation between Ni^I^ halide and BnBF_3_K (**6**), and shows that the electroanalytical technique provides a tool to qualitatively assess the reactivity of benzyltrifluoroborate salts in the crucial transmetalation step. (e) Cross-coupling reactions with Ni(COD)_2_ instead of NiBr_2_ in the dark and with 440 nm LED irradiation. (f) Cross-coupling reactions with (4,4′-Czbpy)Ni(*o*-Tol)Br Ni(COD)_2_ in the dark and with 440 nm LED irradiation. (g) Stoichiometric coupling experiment with a synthetically accessible Ni^I^ species without light. ^a^Yield determined based on the amount of BnBF_3_K (**6**) starting material. Yields were determined by ^1^H NMR or ^19^F-NMR using 1,3,5-trimethoxybenzene or fluorobenzene as internal standard.

Mechanistic investigations shed light on the cross-coupling mechanism by using the photoactive nickel complex (Figure 4). In contrast to photoredox catalysts that have sufficiently long excited-state lifetimes (>1 ns^43^) resulting in characteristic fluorescence or phosphorescence spectra upon excitation,^44^ Ni(4,4′-Czbpy)Br_2_ does not exhibit pronounced steady-state luminescence when irradiated at wavelengths that are employed for cross-coupling catalysis (>380 nm) (Figure 4a, left). This is in agreement with our observations during OTA experiments, which showed that the excited-state ILCT lifetime of Ni(4,4′-Czbpy)Br_2_ is not sufficient (∼20 ps) for a bimolecular SET event between the excited nickel complex and a benzyltrifluoroborate salt when pumped at 400 nm (Figure 2a, right).^43^ Interestingly, the fluorescence spectra recorded from solutions of the photoactive nickel complex at various excitation wavelengths are qualitatively indistinguishable from those obtained from the measurements employing the free ligand 4,4′-Czbpy (Figure 4a, right). Similarly, time-correlated single-photon counting experiments using 340 nm irradiation showed that the fluorescence lifetimes of Ni(4,4′-Czbpy)Br_2_ (14.14 ns) and 4,4′-Czbpy (14.15 ns) are virtually identical (Figures S14 and S15). Together, these observations indicate that the steady-state fluorescence of solutions containing Ni(4,4′-Czbpy)Br_2_ (1:1) is dominated by unbound ligand molecules (*K*_eq_(DMSO) = 5.5 × 10^4^ M^–1^; *K*_eq_(THF) = 7.7 × 10^5^ M^–1^; Figures S7–S11) that does not absorb visible light.

The emission of the 440 nm LEDs used in synthetic cross-coupling experiments does not overlap with the absorption profile of 4,4′-Czbpy (Figure S3) that is responsible for the observed steady-state fluorescence. However, since Ni(4,4′-Czbpy)Br_2_ is in equilibrium with unbound ligand in solution, and because 4,4′-Czbpy has an excited-state lifetime that meets the requirements for photocatalysis, we performed fluorescence quenching studies at 380 (Figure 4b) and 330 nm (Figure S56) using potassium benzyltrifluoroborate (**6**) in large excess (500 equiv). Both experiments showed that the presence of **6** does not impact the emission of Ni(4,4′-Czbpy)Br_2_ and suggested that the C(sp^2^)–C(sp^3^) cross-coupling does not proceed via photoredox catalytic single-electron oxidation of the organoboron compound. Similarly, no luminescence quenching was observed in the presence of 4-bromobenzotrifluoride (**2**) (Figure S58).

Due to these results, we departed from the idea that Ni(4,4′-Czbpy)Br_2_ triggers C(sp^2^)–C(sp^3^) bond formations between benzyltrifluoroborate salts and aryl halides through the dual photoredox/nickel catalysis mechanism. Instead, we hypothesized that the generation of Ni^I^ halide (**II**) through a light-induced ILCT transition^34^ could be followed by a direct transmetalation step with benzylic trifluoroborate salts (Figure 4c). This would provide a different pathway to access a Ni^I^ alkyl intermediate (**VI**) that was reported to undergo facile and irreversible oxidative addition of aryl halides^39^ to produce **VII**, which would furnish the desired coupling product through reductive elimination. Formation of the observed homocoupling side-products is known to result from the reversible homolysis of benzylic Ni–C bonds.^45^ This generates a Ni^0^ species (**V**) that readily undergoes OA to give **VIII**. Photochemical formation of such species is known to produce **II** and an aryl radical,^22−26^ which ultimately abstracts a hydrogen atom from THF. Hence, the low amount of product formation during the cross-coupling between (α-methyl)benzyltrifluoroborate (**44**) and 4-bromobenzotrifluoride (**2**), which resulted in the formation of trifluorotoluene and 2,3-diphenylbutane as main products,^41^ might be a result from the high stability of the secondary benzyl radical, which reduces the propensity for radical capture after homolytic Ni–C fission of **VI**.

To test this mechanistic proposal, we conducted a CV experiment using a mixture of Ni(4,4′-Czbpy)Br_2_ and **6** (Figure 4d, left CV). Since 4,4′-Czbpy was shown to allow electroanalytic studies of the paramagnetic Ni^I^ halide species **II** (*vide infra*), we assumed that this donor–acceptor ligand design could also stabilize the labile Ni^I^ alkyl species (**VI**)^46^ allowing for CV studies of the transmetalation event. We indeed observed the expected change in the CV compared with a reference experiment using only Ni(4,4′-Czbpy)Br_2_: a decrease in the return oxidation peak height (**B**) and the appearance of a new peak (**D**). The lower potential of **D** compared to **B** is indicative of a Ni^I^ species that is easier to oxidize than **II**, such as the proposed transmetalation product **VI**. Grignard reagents are known to undergo transmetalation with *L*_*n*_Ni^I^ halides,^46,47^ which provided the opportunity for a reference CV study using BnMgBr (**45**) and Ni(4,4′-Czbpy)Br_2_ (Figure 4d). This experiment also resulted in the appearance of **D**, confirming that this peak is characteristic of Ni^I^ alkyl species **VI**.

Diao and coworkers showed that the presence of coordinating species, such as bromide anions (TBABr was used as the electrolyte in our CV studies) or solvent molecules (THF was used for catalytic reactions, while DMAc was the solvent of choice for CV experiments), can shift the coordination number of a Ni^I^ halide complex (**II**) bearing redox-active *N,N′*-bidentate complexes from three to four.^48^ The resulting ligand-centered radical is a formal Ni^II^ complex (**II b**) with an empty *3d*(*x*^2^–*y*^2^) orbital that is a better electrophile than the metal-centered species (**II**) and proposed to be responsible for the observed TM reactivity. However, we cannot entirely exclude TM events between trifluoroborates and **II** (the Ni center is sterically less hindered). Importantly, UV–vis experiments showed that the absorption spectrum of Ni(4,4′-Czbpy)Br_2_ remains unaltered upon the addition of BnBF_3_K (**6**) in excess, which indicates that transmetalation between **6** and the Ni^II^ precatalyst is unlikely (Figure S64).^49^

With this evidence for the Ni^I^ transmetalation hypothesis in hand, we next performed CV experiments in the presence of an electron-rich benzyltrifluoroborate salt (**46**) that reacted smoothly under standard conditions (yield of cross-coupling product (**33**): 96%), and an electron-poor derivative (**47**) that gave a modest yield (yield of cross-coupling product (**40**): 23%) (Figure 4d, middle CV). These electroanalytical experiments showed that transmetalation is indeed facile for **46**; the decrease in the return oxidation peak height (**B**) is more pronounced compared to BnBF_3_K (**6**).^50^ Using **47**, on the contrary, we did not observe the diagnostic peak **D.** A CV study using Ni(4,4′-Czbpy)Br_2_ in the presence of potassium (α-methyl)benzyltrifluoroborate (**44**) (Figure 4d, right CV) was similar to the reference experiment (only Ni(4,4′-Czbpy)Br_2_) and does not indicate the formation of a transmetalated species on the CV time scale. Together, these CV experiments demonstrate that the electroanalytical technique provides a tool to qualitatively assess the reactivity of benzyltrifluoroborate salts in the crucial transmetalation step, which can be translated to their performance in light-mediated C(sp^2^)–C(sp^3^) cross-couplings catalyzed by Ni(4,4′-Czbpy)Br_2_.

Having confirmed that neither Ni(4,4′-Czbpy)Br_2_ nor 4,4′-Czbpy are effective photoredox catalysts for oxidizing **6**, and that transmetalation between Ni(4,4′-Czbpy)Br (**II**) and primary benzylic trifluoroborates is feasible, we next sought to study whether the reaction can proceed through a Ni^0^/Ni^II^ mechanism by studying the cross-coupling reaction between benzyltrifluoroborate **6** and aryl bromide **2** in the dark using Ni(COD)_2_ (COD = 1,5-cyclooctadiene) instead of NiBr_2_ (Figure 4e). No cross-coupling or homocoupling product was obtained (the aryl halide was not consumed). This indicates that reversible OA between Ni^0^ and **2** does not result in a Ni^II^ complex that undergoes TM with **6**, followed by reductive elimination of the product from a Ni^II^ intermediate. When the reaction was carried out with 440 nm LED irradiation, quantitative formation of the cross-coupling product and 8% (based on the amount of BnBF_3_K (**6**) used) of 1,2-diphenylethane (**48**) were observed. These results are in line with our mechanistic proposal (Figure 4c): OA between (4,4′-Czbpy)Ni^0^ and an aryl halide results in a photochemically active Ni^II^ complex (**VIII**) that readily undergoes homolytic fission in the presence of light to produce the catalytically active Ni^I^ halide complex (**II**).^22−26^ This was further supported by cross-coupling experiments with (4,4′-Czbpy)Ni(*o*-Tol)Br as a precatalyst (in the dark and using 440 nm LED irradiation), which gave similar results to the reactions carried out with Ni(COD)_2_ and 4,4′-Czbpy (Figure 4f).

Next, we sought to study whether a synthetically accessible Ni^I^ species shows TM and cross-coupling reactivity in the dark. We prepared a solution of (BCP)_2_NiBr (BCP = bathocuproine)^51^ in THF and added stoichiometric amounts of **2** and **6** (Figure 4g).^52^ After stirring this reaction mixture for 48 h in the absence of light, we observed the formation of 1,2-diphenylethane (**48**, 92% based on BnBF_3_K) and small amounts of the cross-coupling product (**7**, 5%).^53^ The low selectivity toward **7** likely results from the ligand’s methyl substituents adjacent to the coordinating nitrogen atoms that stabilize Ni^I^. While TM between Ni^I^ and organometallic reagents in the presence of these sterically encumbering ligands is known to be feasible,^46^ these substituents are known to be detrimental to cross-coupling reactivity (presumably due to steric inhibition of OA to *L*Ni(benzyl) complexes).^19^

**Figure 5:**
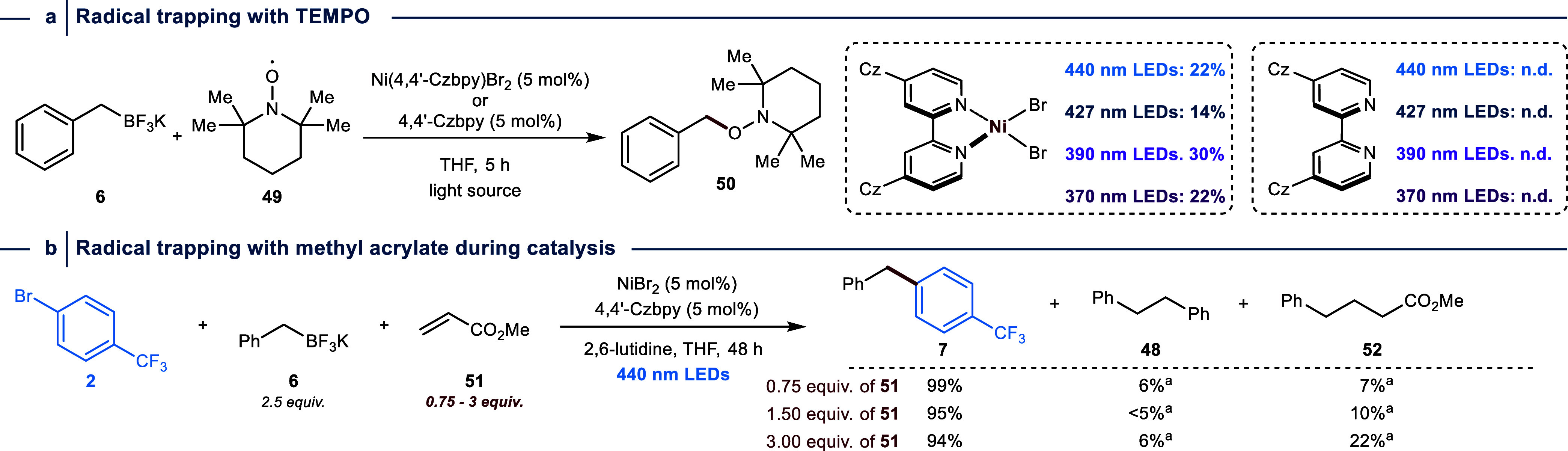
Radical trapping experiments. (a) Radical trapping experiments with TEMPO (**49**) provide evidence for the formation of a C-centered radical upon transmetalation between Ni(4,4′-Czbpy)Br and BnBF_3_K (**6**). (b) Radical trapping experiments using methyl acrylate (**51**) during a catalytic cross-coupling reaction between **2** and **6** provide evidence for off-cycle radical generation. ^a^Yield determined based on the amount of BnBF_3_K starting material. Yields were determined by ^1^H NMR or ^19^F-NMR using 1,3,5-trimethoxybenzene or fluorobenzene as internal standard.

Finally, we performed a series of experiments to study the formation of 1,2-diphenylethane (**48**) in more detail (Figure 5). We carried out radical trapping experiments by irradiating a mixture of Ni(4,4′-Czbpy)Br_2_, **6** and (2,2,6,6-tetramethylpiperidin-1-yl)oxyl (TEMPO, **49**) using various wavelengths. These experiments yielded the desired adduct (**50**) in moderate yields, whereas no trapping product was detected using the bare ligand (Figure 5a). Similar results were obtained using a Michael acceptor instead of TEMPO as a radical trap (Table S14). This provided us with the opportunity to conduct radical trap experiments under catalytic conditions using the model reaction between **2** and **6** in the presence of varying amounts of methyl acrylate (**51**, 0.75, 1.5, and 3 equiv) (Figure 5b). All reactions resulted in the quantitative formation of the cross-coupling product **7** and small amounts of 1,2-diphenylethane (**48**). The amount of the radical trapping product (**52**) gradually increased with higher amounts of **51.** These results are in agreement with the proposed off-cycle formation of benzylic radicals.

## Conclusion

In summary, we demonstrated that the catalytic activity of photoactive nickel complexes can be adjusted through ligand modifications. Tuning nickel’s OA reactivity through the ligand field enabled the expansion of the C(sp^2^)–S cross-coupling scope to a previously unreactive aryl bromide by facilitating oxidative addition. The same ligand modification accessed light-mediated C(sp^2^)–C(sp^3^) cross-couplings between aryl halides and benzyltrifluoroborate salts in the absence of an exogenous photoredox catalyst. Mechanistic investigations provided evidence that transmetalation between a photochemically generated Ni^I^ species and benzyltrifluoroborates is feasible. Our findings suggest that photochemically mediated nickel-catalyzed C(sp^2^)–heteroatom and C(sp^2^)–C(sp^3^) cross-couplings can both proceed through Ni^I^/Ni^III^ cycles.

## Supporting Information Available

Additional experimental details, materials, and methods, including photographs of experimental setup (PDF)
