# Supplementary material for: Evidence for a Unifying NiI/NiIII Mechanism in Light-Mediated Cross-Coupling Catalysis
Source: J Am Chem Soc. 2025 Apr 11;147(16):13169–79. doi: 10.1021/jacs.4c16050 (PMC12022987; doi:10.1021/jacs.4c16050)
Supplement: Supplementary file 1 — ja4c16050_si_001.pdf [file ja4c16050_si_001.pdf]

SUPPORTING INFORMATION

Evidence for a unifying Ni^{I}/Ni^{III} mechanism in light-mediated cross-coupling catalysis

Lucia Anghileri[1,2,3], Haralds Baunis[1,2,†], Aleksander R. Bena[1,2,†], Christos Giannoudis[1,2,†], John H. Burke[4], Susanne Reischauer[2], Christoph Merschjann[5], Rachel F. Wallick[4], Tarek Al Said[5], Callum E. Adams[1], Gianluca Simionato[6], Sergey Kovalenko[7], Luca Dell′Amico[6], Renske M. van der Veen[4,5,8] and Bartholomäus Pieber[1,2*]

[1]*Institute of Science and Technology Austria (ISTA), Am Campus 1, 3400 Klosterneuburg, Austria*

[2]*Department of Biomolecular Systems, Max-Planck-Institute of Colloids and Interfaces (MPICI), Am Mühlenberg 1, 14476 Potsdam, Germany*

[3]*Department of Chemistry and Biochemistry, Freie Universität Berlin; Arnimallee 22, 14195 Berlin, Germany*

[4]*Department of Chemistry, University of Illinois Urbana−Champaign; Urbana, Illinois 61801, United States*

[5]*Helmholtz Zentrum Berlin für Materialien und Energie GmbH; Hahn-Meitner-Platz 1, 14109 Berlin, Germany*

[6]*Department of Chemical Sciences, University of Padova, Via Francesco Marzolo 1, 35131 Padova, Italy*

[7]*Department of Chemistry, Humboldt-Universität zu Berlin, Brook-Taylor-Str. 2, 12489 Berlin, Germany*

[8]*Institute of Optics and Atomic Physics, Technische Universität Berlin, Hardenbergstraße 36, 10623 Berlin, Germany*

†These authors contributed equally to this work

**Corresponding author. Email: bartholomaeus.pieber@ist.ac.at*

Table of Contents

Materials and Methods 3
Experimental setup for photochemical reactions 4
Ligands and complexes 5
Synthesis of 4,4′-Czbpy 5
Cyclic voltammetry 6
UV-Vis spectroscopy 7
Determination of Complex Equilibrium Constant of 4,4′-Czbpy and $NiBr_2$ in DMSO and THF 10
Fluorescence spectroscopy 14
Time-Correlated Single Photon Counting (TC-SPC) of 4,4′-Czbpy and Ni(4,4′-Czbpy)Br_2 16
Optical transient absorption spectroscopy (OTA) 17
Time-Dependent Density Functional Theory Studies (TD-DFT) 20
EPR experiments 33
C–S cross-coupling 42
Experimental procedure 42
CV studies: Oxidative addition 44
Ni(5,5′-Czbpy)Br_2 44
Ni(4,4′-Czbpy)Br_2 46
C–C cross-coupling 48
Initial experiments and reaction optimization 48
Scope 54
Mechanistic Studies 71
Luminescence quenching 71
CV studies 73
UV/Vis studies 76
Experiments using $Ni(COD)_2$ and (4,4′-Czbpy)Ni(o-Tol)Br 77
Experiments using $(BCP)_2$NiBr 80
Radical trapping 82
References 84
Copies of NMR spectra 87
Optimized Geometries - Time-Dependent Density Functional Theory Studies 129

Materials and Methods

All reagents and solvents were purchased from commercial suppliers and used without further purification. All cross-coupling experiments were carried out with dry, unstabilized solvents. 5,5′-Czbpy was synthesized according to a previously reported procedure.[1] Trifluoroborate salts were synthesized using literature procedures.[2, 3] Analytical thin layer chromatography (TLC) was performed on pre-coated TLC-sheets (ALUGRAM Xtra SIL G/UV_{254} sheets, Macherey-Nagel) and visualized with 254 nm light. Purification of final compounds was carried out by preparative TLC on pre-coated TLC-sheets (ALUGRAM Xtra SIL G/UV_{254} sheets, Macherey-Nagel), manual flash chromatography using Silica 60 M (0.04-0.063 mm, Sigmal Aldrich), or by flash chromatography on the Reveleris X2 Flash Chromatography System from GRACE, or on the CombiFlash® NextGen 300 (Teledyne LABS) using prepacked columns with 40 μm silica gel, or amino functionalized silica columns (FlashPure EcoFlex Amino (Buchi) or Redisep Gold® (Teledyne LABS)). High-resolution mass spectral data were obtained using a GC-EI-Q-TOF 7250 A (Agilent), and a LC-ESI-Q-TOF maXis (Bruker Daltonics GmbH & Co KG). ^{1}H-, ^{13}C-, and ^{19}F spectra were recorded on a Varian 400 spectrometer (400 MHz, Agilent), an Ascend™ 400 spectrometer (400 MHz, Bruker), an Avance IV (600 MHz, Bruker) and a Varian 600 spectrometer (600 MHz, Agilent) at 298 K, and are reported in ppm relative to the residual solvent peaks. Peaks are reported as: s = singlet, d = doublet, t = triplet, q = quartet, m = multiplet or unresolved, with coupling constants in Hz. UV/Vis spectra were recorded using a UV-1900 spectrometer (Shimadzu). Fluorescence spectroscopy studies were performed at room temperature using a Jasco FP-8350 fluorescence spectrophotometer. Samples were prepared in a glove box and measured in 10x10 mm path length and 1.5x1.5 mm path length quartz cuvettes. Time-Correlated Single Photon Counting (TC-SPC) experiments were performed at room temperature on a FLS1000 UV/Vis/NIR photoluminescence spectrometer (Edinburgh Instruments). The instrument was coupled with a high-speed detector with amplifier, operating in the spectral range: 230-850 nm and with a response time width <180 ps. Excitations were performed with EPLED 340 (Edinburgh Instruments), emitting at 341.6 nm. X-band EPR measurements were performed at room temperature using an Elexsys E580 spectrometer (Bruker Biospin) equipped with a critically coupled ER 4118X-MD5 resonator (Bruker Biospin). Cyclic voltammetry (CV) experiments were conducted in a custom-made three-electrode cell using a Emstat 4 potentiostat (PalmSens). A glassy carbon electrode was used as the working electrode (Ø 1.6 mm) and a glassy carbon rod as

the counter electrode (Ø 1.6 mm). The working electrode was polished using a polishing alumina suspension (0.05 µm). As reference, an $Ag/AgNO_3$ electrode (silver wire in 0.010M $AgNO_3$, 0.100M Bu_4NPF_6 solution in MeCN, separated from the solution with a glass frit) was used and referenced against the ferrocene/ferrocenium Fc/Fc^+ couple (1 mM in MeCN, 0.1M Bu_4NPF_6 as supporting electrolyte). All CV experiments were carried out under inert conditions. The electrolyte solution was sparged with argon for 5 min prior to recording each measurement. GC-MS analysis was performed on Agilent 8860/5977B GCMS equipped with a 7693A autosampler using a HP-5MS ultra inert column (30 m×0.25 mm×0.25µm).

Experimental setup for photochemical reactions

All photochemical synthetic experiments were carried out using Kessil lamps (PR160L-440, PR160L-427, PR160L-390, PR160L-370 with the respective power settings. The emission spectra of these lamps and further specifications can be found online (https://kessil.com/products/science_PR160L.php).

One or two lamps were used to irradiated the reaction vessel that was placed on a stirring plate (Fig. S1) (lamp-vessel distance: 4.5 cm; stirring speed: 800 rpm). The reacitons mixtures were cooled with CPU fans. Under optimized conditions (double-lamp setup with two fans, see Fig S1, right) the inner temperature of the reaction mixture was 45 °C. Without fan cooling the reaction temperature was 60 °C.

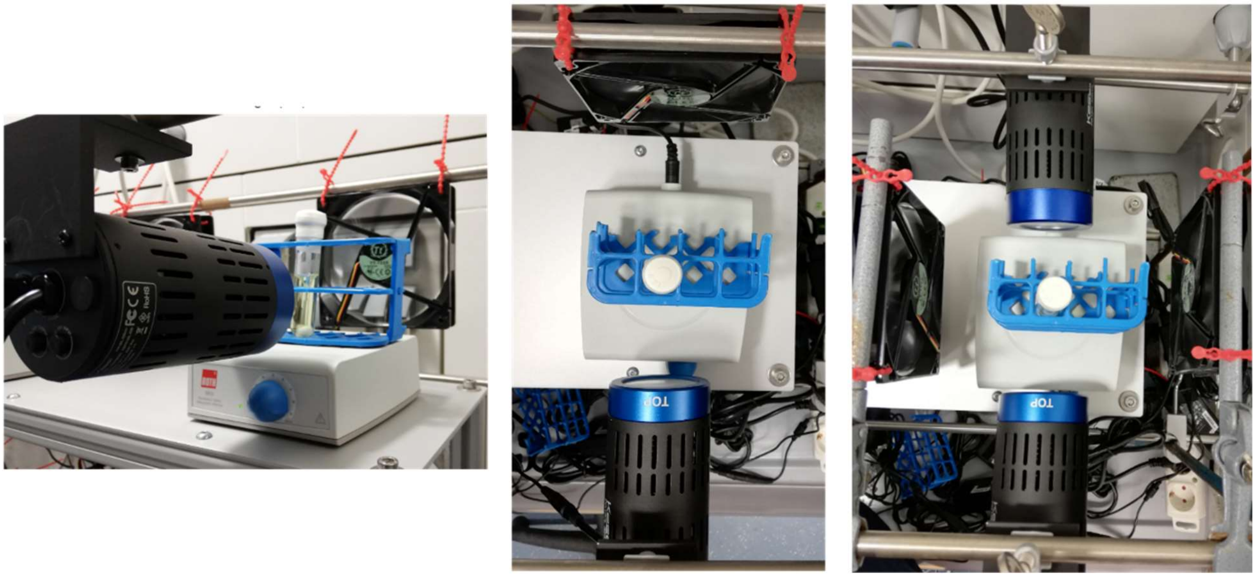

Fig. S1: Experimental setups for double- and single-lamp experiments.

Ligands and complexes

Synthesis of 4,4′-Czbpy

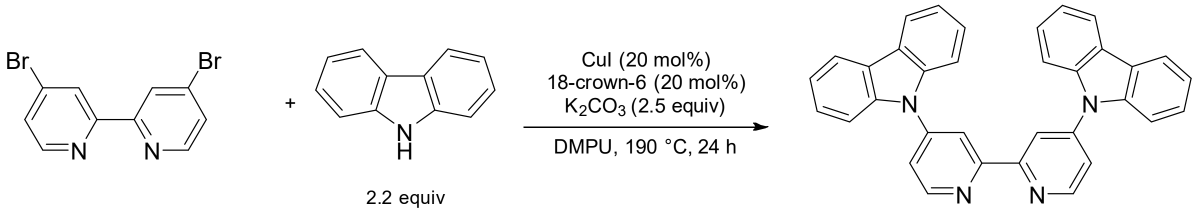

A 100 mL Schlenk flask was charged with 4,4′-dibromo-2,2′-bipyridine (0.50 g, 1.59 mmol), carbazole (0.58 g, 3.5 mmol, 2.2 equiv), copper(I) iodide (61 mg, 0.32 mmol, 20 mol%), 18-crown-6 (84 mg, 0.32 mmol, 20 mol%), potassium carbonate (549 mg, 3.98 mmol, 2.5 equiv.) and 1,3-dimethyl-3,4,5,6-tetrahydro-2(1H)-pyrimidinone (DMPU, 1.67 mL). The flask was connected to a reflux condenser and the mixture was stirred for 24 h at 190 °C using an oil bath. Over time, the yellow reaction mixture became a black viscous oil. After 24 h, the mixture was quenched with 2 M HCl (100 mL). Aqueous NH_3 was added until the pH was adjusted to ~8. The resulting mixture was extracted with CH_2Cl_2 (2 x 125 mL). The combined organic phases were washed with brine, dried over magnesium sulfate and the solvent was removed under reduced pressure. The crude product was purified by flash chromatography using functionalized amine silica gel prepacked columns (CH_2Cl_2/hexane, 0-100%). Subsequent crystallization from CH_2Cl_2/hexane (9:1) to afford the title compound as grey powder (266 mg, 0.55 mmol, 35%).

^{1}H NMR (400 MHz, $CDCl_3$) δ 8.96 (d, *J* = 28.4 Hz, 4H), 8.20 – 8.14 (m, 4H), 7.74 (d, *J* = 8.3 Hz, 6H), 7.51 (ddd, *J* = 8.3, 7.1, 1.2 Hz, 4H), 7.39 (t, *J* = 7.4 Hz, 4H).^{13}C NMR (101 MHz, 40°C, $CDCl_3$) 157.74, 150.97, 146.88, 139.71, 126.44, 124.38, 121.13, 120.77, 120.52, 118.31, 110.06. HRMS (ESI) m/z calcd for $C_{34}H_{22}N_4$ [$(M+H)^+$] 487.1917, found 487.1912.

Cyclic voltammetry

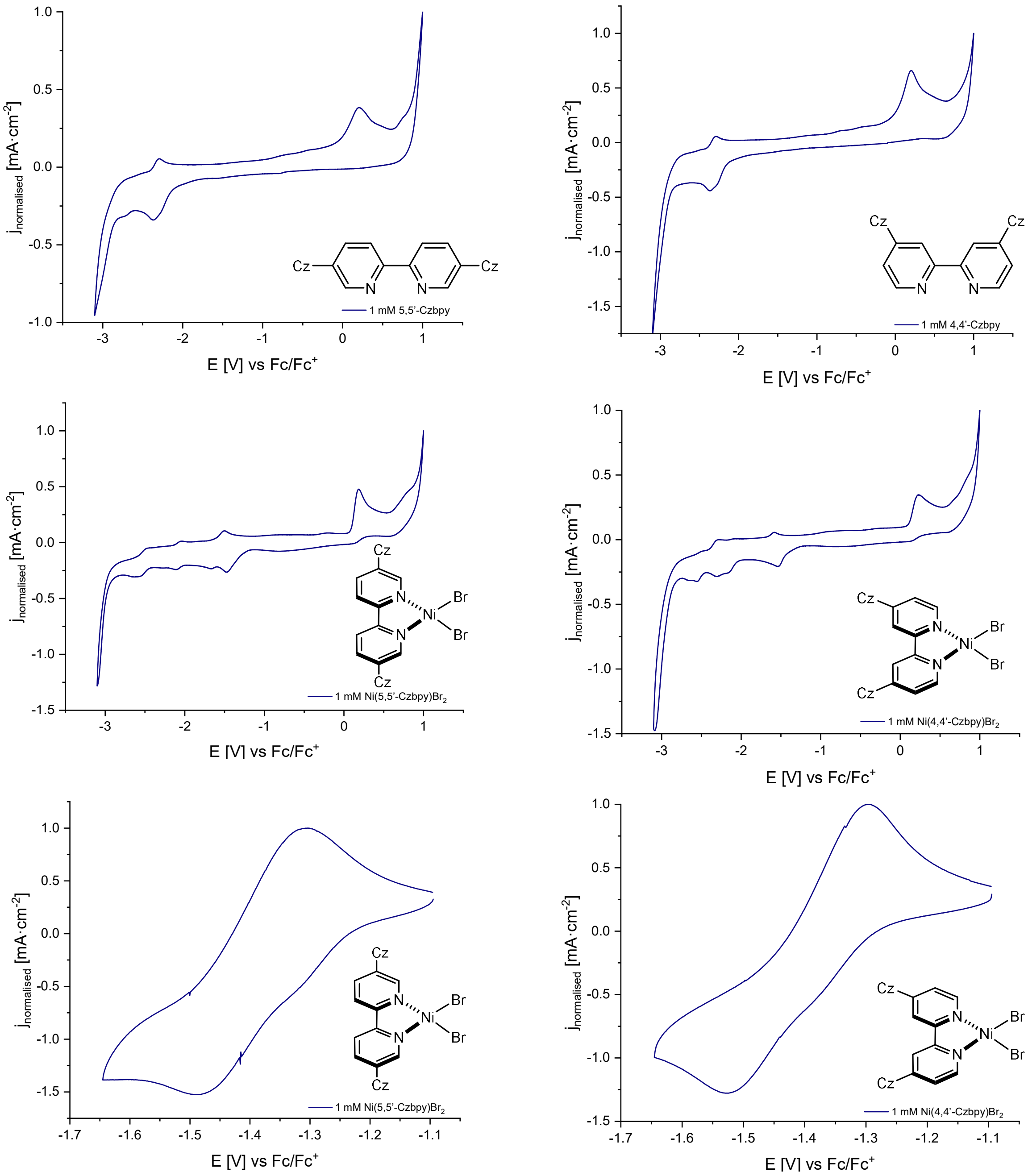

Fig. S2: CVs of 1 mM solution of 5,5′-Czbpy, and Ni(5,5′-Czbpy)Br_2, 4,4′-Czbpy, Ni(4,4′-Czbpy)Br_2 in DMAc with 0.1M Bu_4NPF_6 as supporting electrolyte at 100 mV·s^{-1}. Ni^{II}/Ni^{I} couples (bottom) were measured with 0.1M Bu_4NBr as supporting electrolyte.

UV-Vis spectroscopy

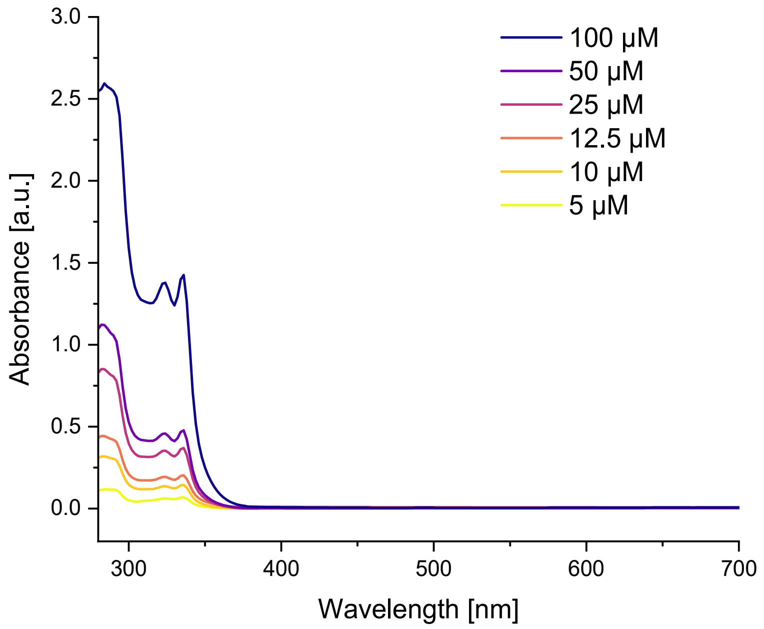

Fig. S3: UV-Vis absorption spectra of 4,4′-Czbpy in DMSO at different concentrations.

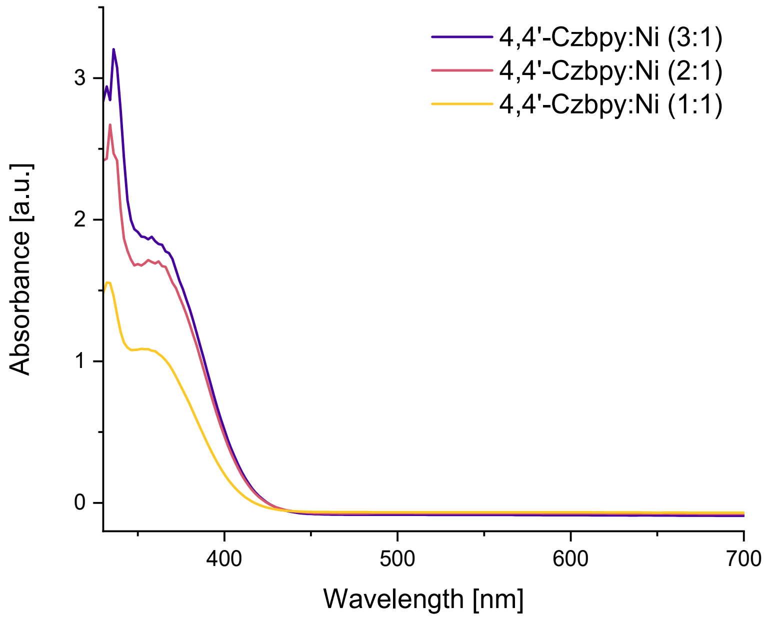

Fig. S4: UV-Vis absorption spectra of different ratios of 4,4′-Czbpy ($1\cdot10^{-4}$ M) and $NiBr_2$•glyme in DMSO

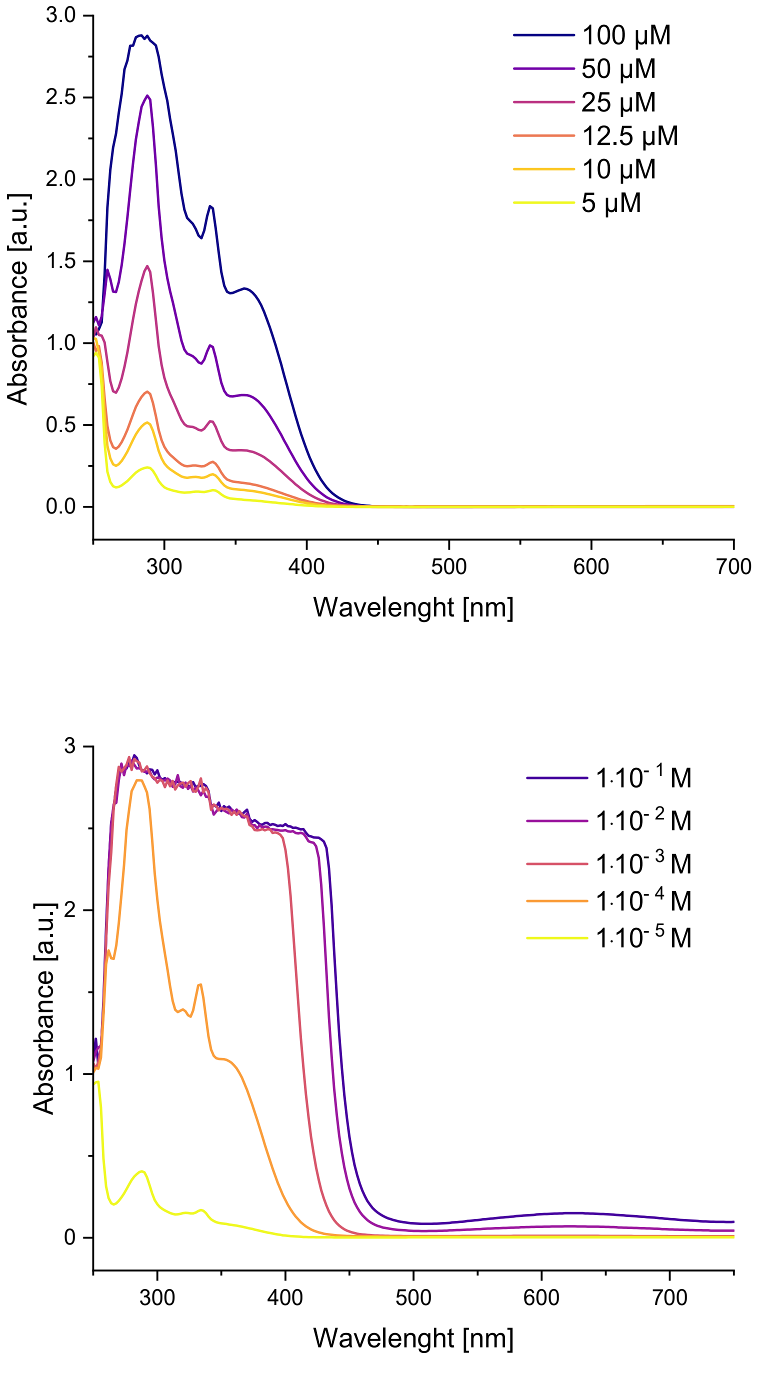

Fig. S5: Concentration study of Ni(4,4′-Czbpy)Br_2: UV-Vis absorption spectra of a 1:1 mixture of 4,4′-Czbpy and $NiBr_2$·glyme in DMSO.

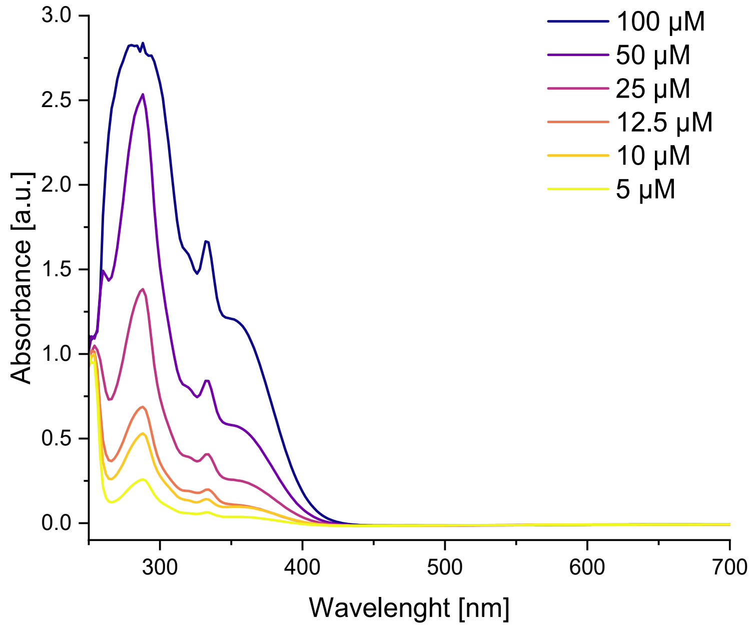

Fig. S6: Concentration study of Ni(4,4′-Czbpy)Cl_2: UV-Vis absorption spectra of a 1:1 mixture of 4,4′-Czbpy and $NiCl_2$·glyme in DMSO.

Determination of Complex Equilibrium Constant of 4,4′-Czbpy and $NiBr_2$ in DMSO and THF

Concentration-dependent UV-vis absorption spectroscopy experiments were performed to determine the equilibrium constant of complex formation (K_{eq}.) between $NiBr_2$ (using the commercially available glyme adduct) and the donor-acceptor ligands (L)

$$\mathrm{L} + \mathrm{NiBr_2} \rightleftharpoons \mathrm{LNiBr_2}$$

$$\mathrm{K_{eq}} = \frac{[\mathrm{LNiX_2}]}{[\mathrm{L}][\mathrm{NiX_2}]} = \frac{y}{(C_L - y)(x - y)}$$

Solutions were prepared with a constant initial ligand concentration (C_L) while varying the concentration of nickel salt added (x). Solving for the final concentration of complex, y, gives:

$$y(x) = \frac{-\sqrt{\left(C_L K_{eq} + K_{eq} x + 1\right)^2 - 4 C_L K_{eq}^2 x} + C_L K_{eq} + K_{eq} x + 1}{2K_{eq}}$$

The concentration of complex was determined by UV-vis absorption spectroscopy by measuring the absorbance (A) at a wavelength corresponding to absorption from the complex.

$$A(x) = (\varepsilon b)\frac{-\sqrt{\left(C_L K_{eq} + K_{eq} x + 1\right)^2 - 4 C_L K_{eq}^2 x} + C_L K_{eq} + K_{eq} x + 1}{2K_{eq}}$$

The concentration of the complex is related to the absorbance through Beer′s law given the path-length (b) and extinction coefficient (ε). Fitting curves of absorbance versus nickel salt concentration allows extraction of K_{eq}. The extinction coefficient, ε, was either held constant or allowed to vary as a fit parameter.

Complexation of $NiBr_2$·glyme by 4,4′-Czbpy in DMSO

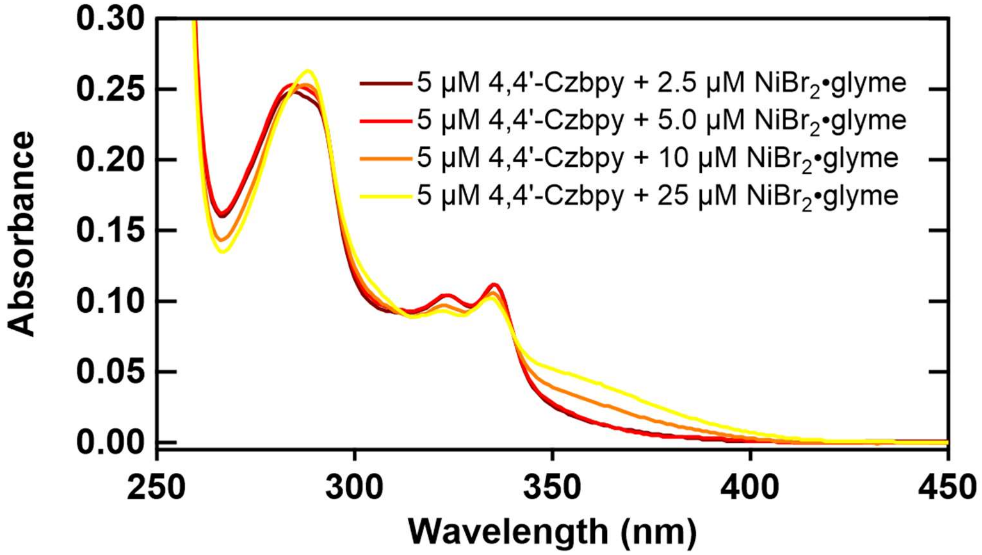

Fig. S7: UV-Vis absorption spectra of 5 μM 4,4′-Czbpy in DMSO with varying concentrations of $NiBr_2$•glyme. Path length is 1 cm.

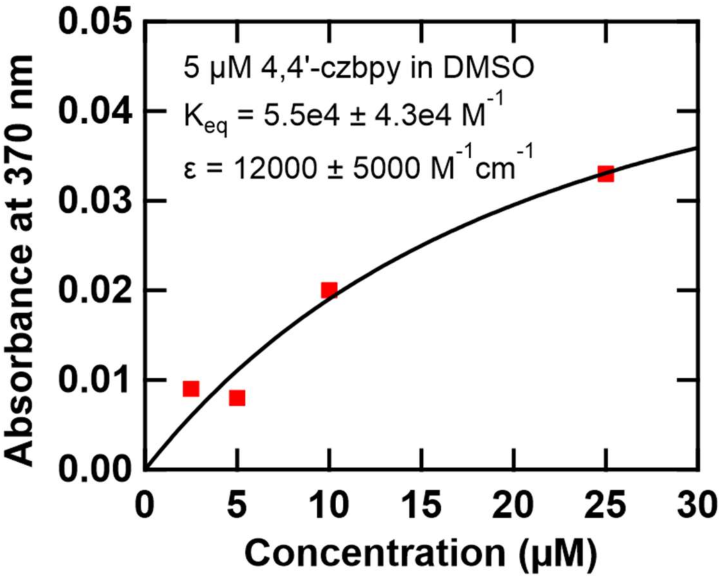

Fig. S8: Absorbance at 370 nm (red squares) for 5 μM 4,4′-Czbpy in DMSO with varying concentrations of $NiBr_2$•glyme (x axis). Solid line is fit to Equation S3.

Complexation of $NiBr_2$·glyme by 4,4'-Czbpy in THF

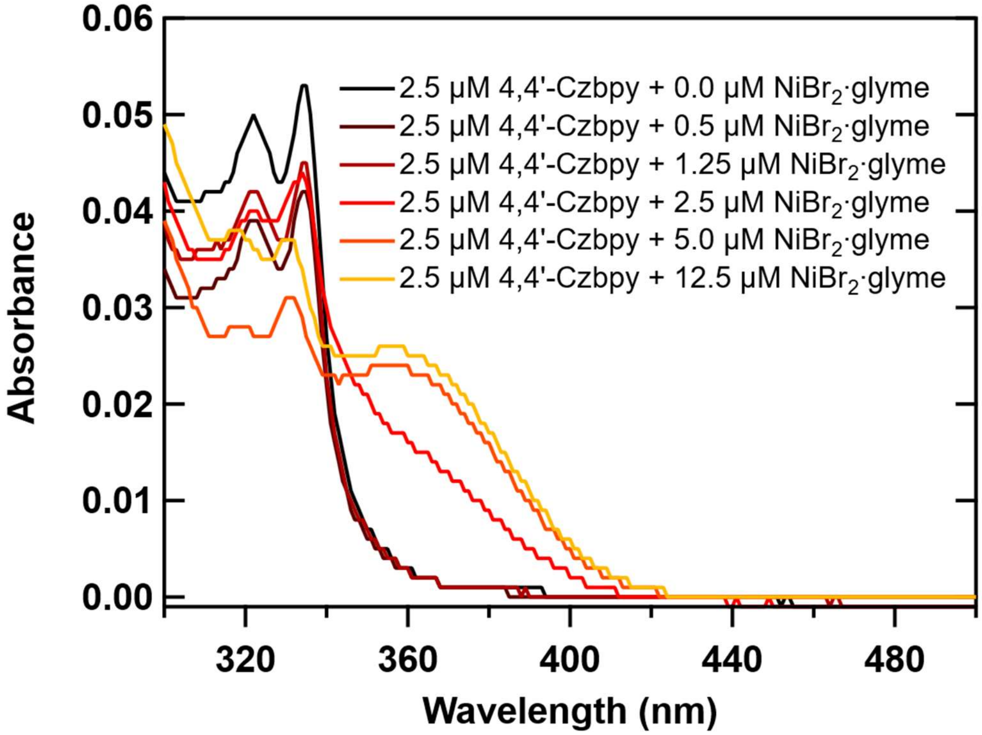

Fig. S9: UV-Vis absorption spectra of 2.5 µM 4,4′-Czbpy in THF with varying concentrations of $NiBr_2$•glyme. Path length is 1 cm.

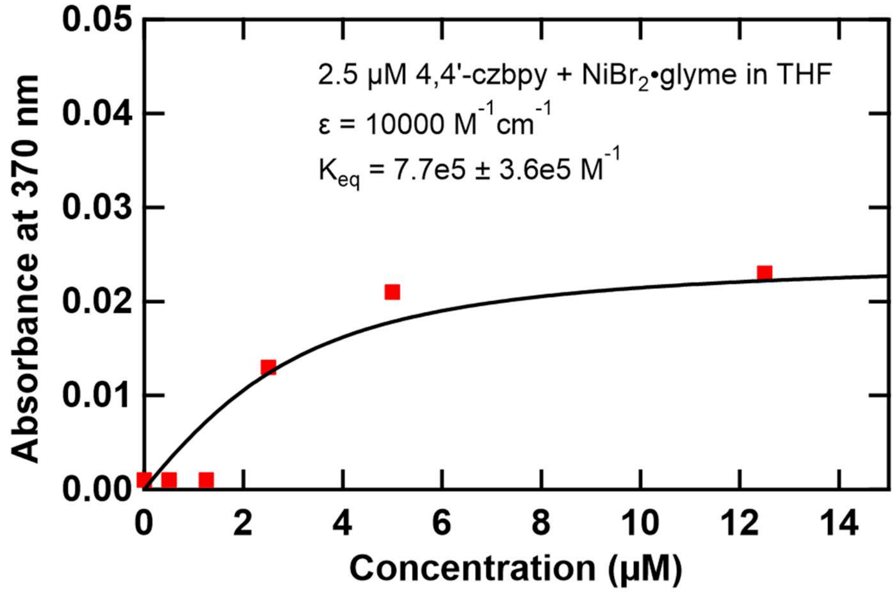

Fig. S10: Absorbance at 370 nm (red squares) for 2.5 µM 4,4′-Czbpy in THF with varying concentrations of $NiBr_2$•glyme (x axis). Solid line is fit to Equation S3 with the extinction coefficient, ε, assumed to be 10000 M-1cm-1 and path-length, b, of 1 cm.

Determination of free ligand concentration

To estimate the amount of free ligand present in photocatalytic reaction mixtures, we utilized the equilibrium constants determined above to calculate the amount of free ligand that would be present in a 1:1 mixture of ligand and nickel salt. Given equal amounts of ligand and nickel salt, the equation for K_{eq} is given by:

$$\mathrm{K_{eq}} = \frac{[\mathrm{LNiX_2}]}{[\mathrm{L}][\mathrm{NiX_2}]} = \frac{y}{(x-y)(x-y)} = \frac{y}{x^2 - 2yx + y^2}$$

Solving for the concentration of complex (y) as a function of initial 1:1 ligand:nickel salt concentration (x) gives:

$$y(x) = \frac{-\sqrt{4K_{eq}x + 1} + 2xK_{eq} + 1}{2K_{eq}}$$

This equation allows us to calculate the concentration of complex and free ligand as a function of catalyst loading.

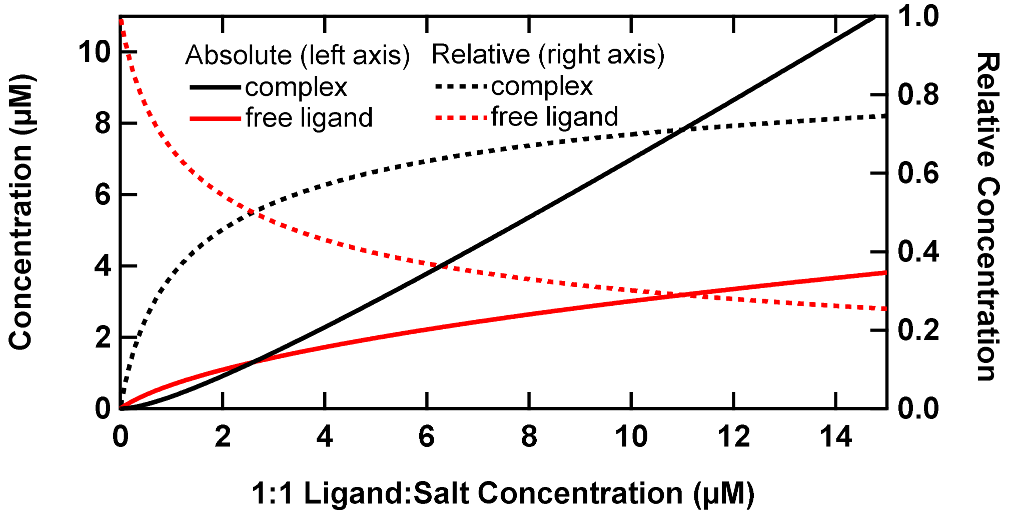

Fig. S11: Calculated concentrations of complex and free ligand for varying 1:1 concentrations of ligand and salt, given an equilibrium constant of 7.7e5 M-1.

Fluorescence spectroscopy

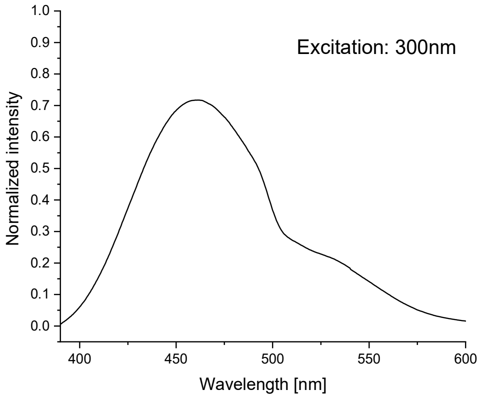

c = 10 μM, ex./em. bandwidth = 2.5/5 nm, response = 0.2 sec, optical path = 1 cm

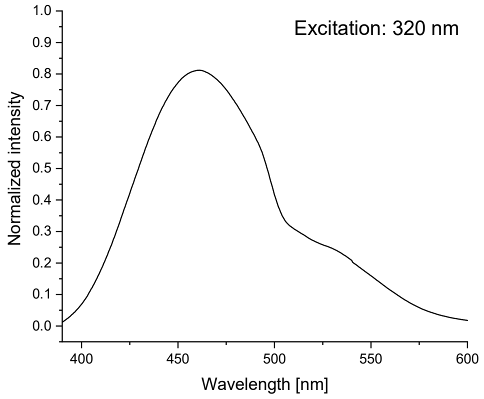

c = 10 μM, ex./em. bandwidth = 2.5/5 nm, response = 0.2 sec, optical path = 1 cm

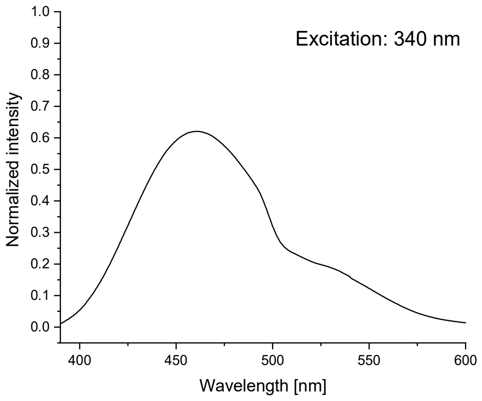

c = 10 μM, ex./em. bandwidth = 2.5/5 nm, response = 0.2 sec, optical path = 1 cm

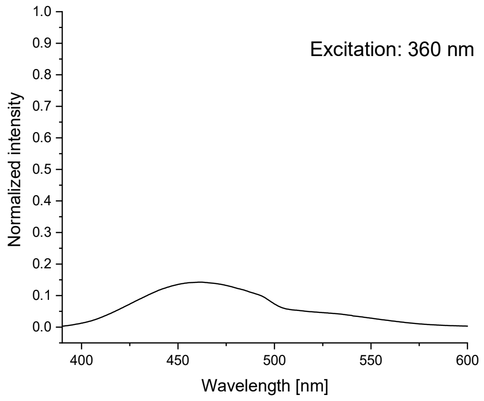

c =100 μM, ex./em. bandwidth = 2.5/2.5 nm, response = 0.2 sec, optical path = 1 cm

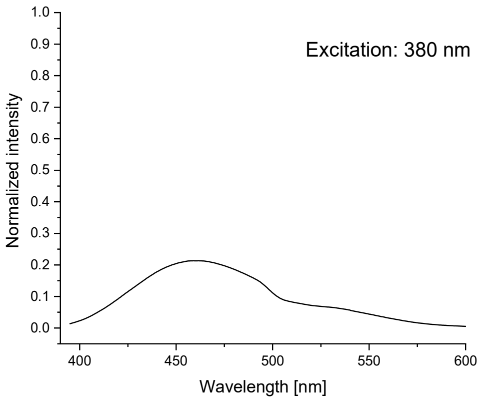

c =100 μM, ex./em. bandwidth = 5/5 nm, response = 0.2 sec, optical path = 1 cm

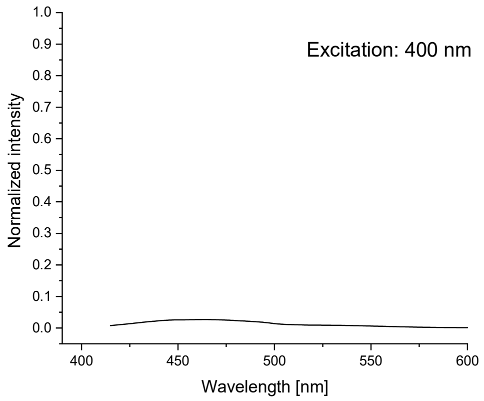

c =100 μM, ex./em. bandwidth = 10/5 nm, response = 0.2 sec, optical path = 1 cm

Fig. S12: Fluorescence spectra of 4,4′-Czbpy in DMSO

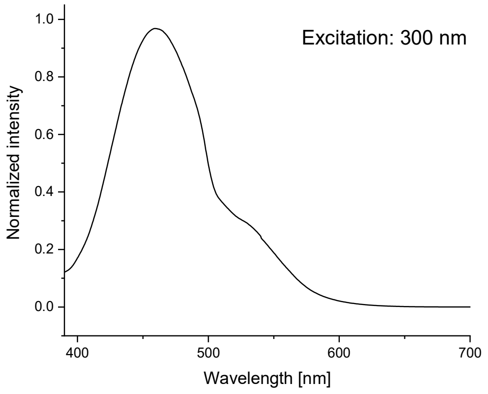

c = 2.5 µM, ex./em. bandwidth = 5/5 nm,
response = 0.2 sec, optical path = 1 cm

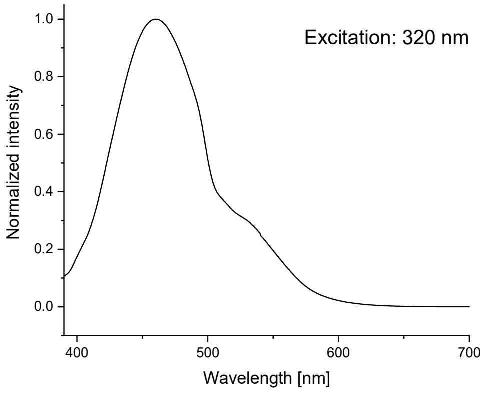

c = 2.5 µM, ex./em. bandwidth = 5/5 nm,
response = 0.2 sec, optical path = 1 cm

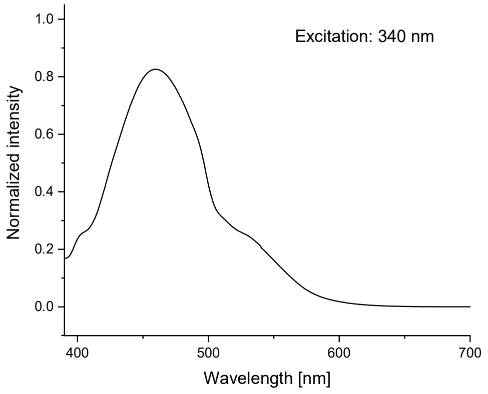

c = 2.5 µM, ex./em. bandwidth = 5/5 nm,
response = 0.2 sec, optical path = 1 cm

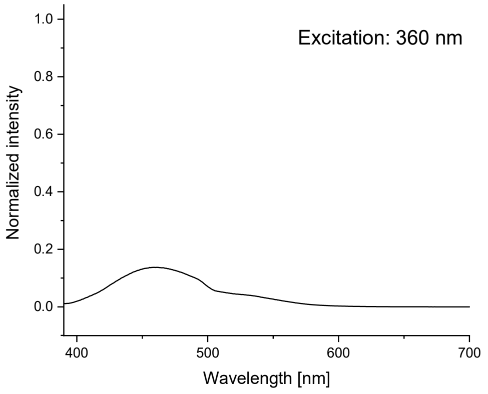

c = 10 µM, ex./em. bandwidth = 5/5 nm,
response = 50 msec, optical path = 1 cm

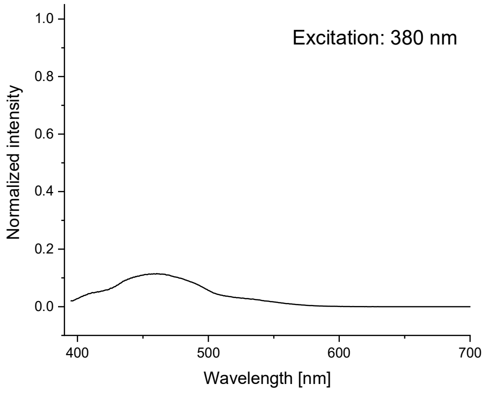

c =10 µM, ex./em. bandwidth = 10/10 nm,
response = 50 msec, optical path = 1 cm

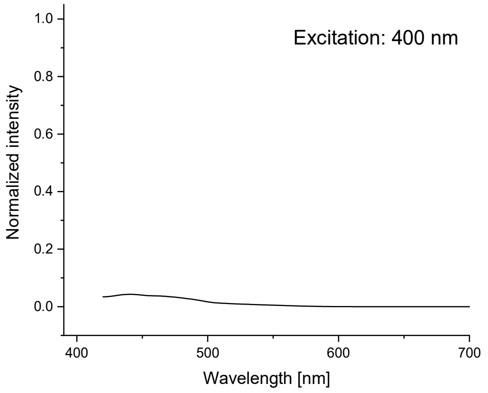

c = 1 mM, ex./em. bandwidth = 5/5 nm,
response = 0.5 sec, optical path = 1.5 mm

Fig. S13: Fluorescence spectra of Ni(4,4′-Czbpy)Br_2 in DMSO

Time-Correlated Single Photon Counting (TC-SPC) of 4,4′-Czbpy and Ni(4,4′-Czbpy)Br_2

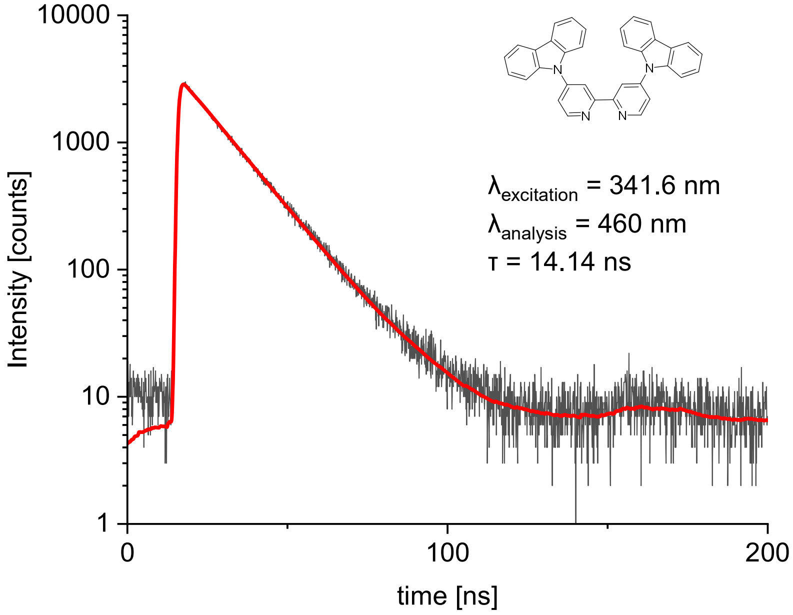

Fig. S14: Time-resolved emission decay (excitation at 341.6 nm, analysis at 460 nm) of 4,4′-Czbpy in DMSO solution measured by TC-SPC (τ = 14.14 ns, from deconvolution and single-exponential fitting, X^2 = 1.307).

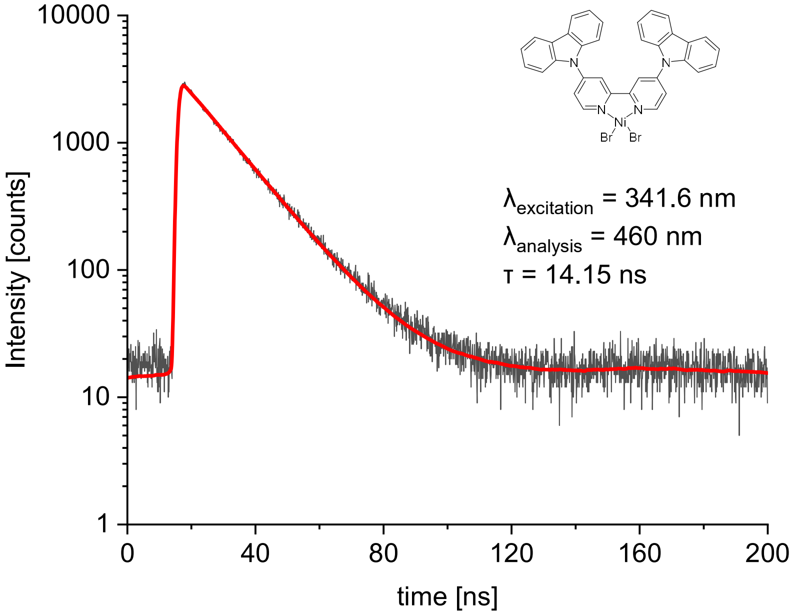

Fig. S15: Time-resolved emission decay (excitation at 341.6 nm, analysis at 460 nm) of Ni(4,4′-Czbpy)Br_2 in DMSO solution measured by TC-SPC (τ = 14.15 ns, from deconvolution and single-exponential fitting, X^2 = 1.172).

Optical transient absorption spectroscopy (OTA)

Optical transient absorption (OTA) spectroscopy was performed using two different experimental setups for the ligand and complex measurements, respectively.

Measurements of Ni complexes:

Here, a custom-built setup in standard transmission geometry with near-vertical incidence of pump and probe light was applied. The setup used was pumped using a commercial Ti:sapphire laser system (Coherent Inc., Legend Elite Duo), delivering pulses of 25 fs duration at 800 nm central wavelength and 2.5 mJ pulse energy with a repetition rate of 5 kHz.

Pump-light generation: Visible excitation light centred at 400 nm (3.1 eV) was generated by frequency doubling about 0.2 mJ of the fundamental pulse in a β-BaB2O4 (BBO) crystal. The maximum energy of the pump beam at the sample position was about 1.4 µJ per pulse. The excitation spot had a $1/e^2$ diameter of about 600 µm, resulting in a radiant exposure of about 0.5 mJ cm^{-2}.

Probe-light generation: The transient absorption changes were probed over the ultraviolet-visible spectral range with a white light continuum (WLC). Therefore, a suitable proportion of the fundamental pulse energy was split off and focused into a 3 mm thick CaF_2 substrate. The obtained WLC covers a usable range of 380 – 750 nm. The probe spot had a $1/e^2$ diameter of about 150 µm, and the probe radiant exposure was typically below 1 nJ cm^{-2} nm^{-1}.

Sample preparation and data acquisition: Sample solutions were prepared by dissolving a specified amount of ligand and $NiBr_2$·glyme (1:1 ratio) in DMSO. These solutions were filled into a 1 mm pathlength fused-silica cuvette. To reduce sample damage, the cuvette was moved perpendicular to the optical axis in a zig-zag scheme at a velocity of 2.4 mm s^{-1}. Both pump and probe pulses could be individually polarized linearly. Here, a combination of vertical probe and magic-angle (54.7° from vertical) pump polarization was used throughout the experiments. After passing through the sample, the probe pulse was spectrally dispersed using a Czerny-Turner spectrograph (Andor Technology, Shamrock 303) and detected with a multichannel detector (Andor Technology, Newton DU920P-BEX2-DD). Transient spectra were recorded up to a pump–probe delay of 1800 ps using an optical delay stage. A semi-logarithmic delay scheme was used, providing a dense linear sampling during the ultrafast period, and logarithmic sampling afterwards. The maximum detector read-out speed was about 200 Hz, limiting the number of integrated pump–probe spectra per delay time to a few hundred.

Ligand measurements:

The experimental setup with applications has been described elsewhere.[4-7] TA spectra $\Delta A(\lambda,t)$ are recorded with 0.02 and 8 ps time steps at the magic angle polarizations. Measurements are performed in the range 275-690 nm with 0.09 ps (fwhm) instrument response. Multiple (8-48) back-and-forth pump-probe scans are used to improve the signal-to-noise.

A 10 ml solution of the respective in DMSO flows through a sample cell of 0.3 mm thick. Absorbance $A(\lambda)$ in the measurement cell was less than 0.5 at the peak of the band to ensure a high-quality signal in the bleach region. The sample was excited at 345 nm with pulse energy 8-24 nJ. The pump and probe beams, with 15° between them, are focused onto the cell with spot diameter of 0.15 mm.

Data treatment:

Transient absorption data are subject to a wavelength-dependent time of pump-probe interaction, caused by the chirp of the broadband probe light.[8] This chirp was manually modelled using a polynomial of degree 2 on the photon-energy scale, and the data were thus corrected for time-zero dispersion.[8] TA signals were additionally background-corrected by subtracting a spectrum averaged over a suitable delay time range before the pump-probe interaction from the dataset.

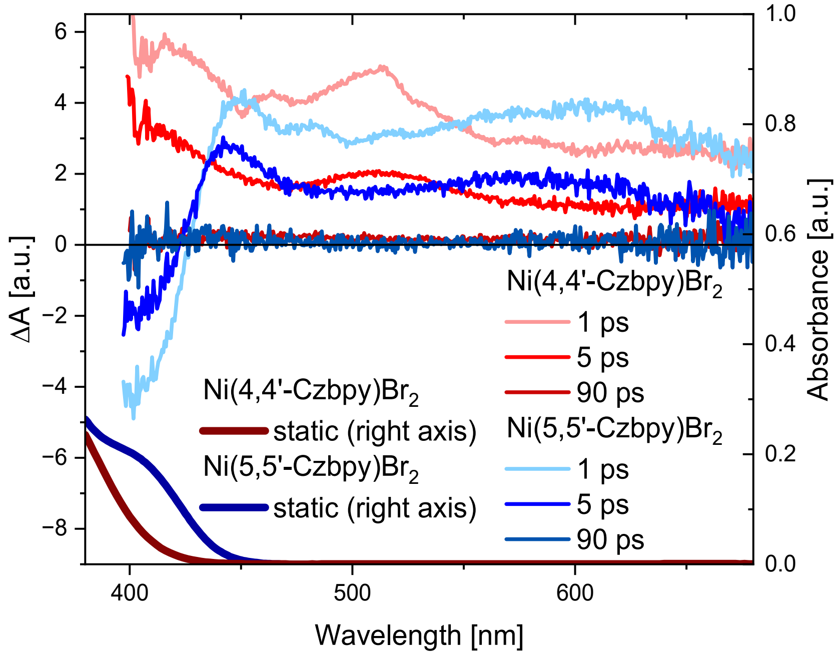

Fig. S16: Optical transient absorption spectra (400 nm pump) at select time delays of Ni(4,4′-Czbpy)Br_2 and Ni(5,5′-Czbpy)Br_2 in DMSO.

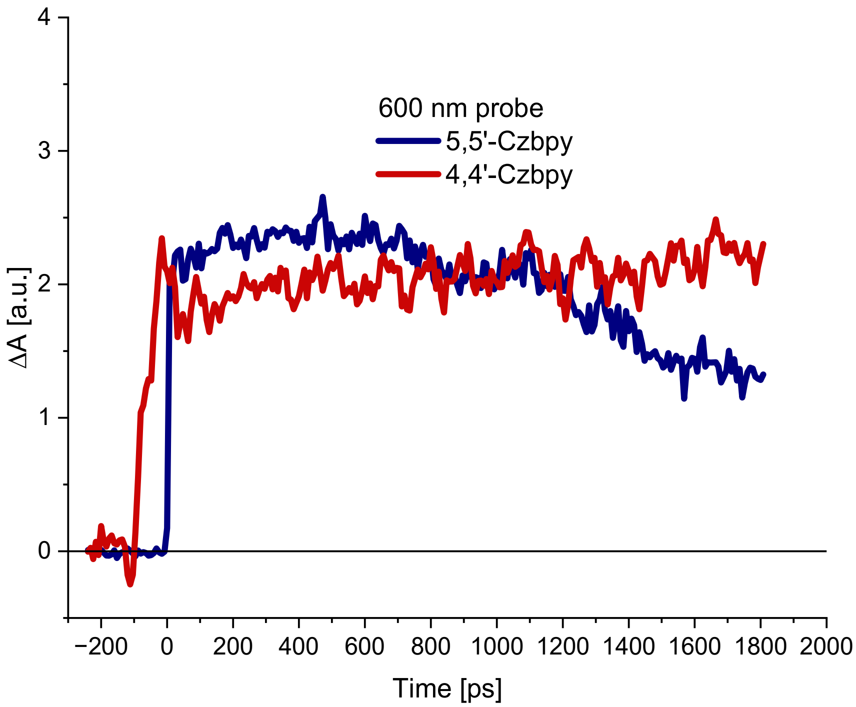

Fig. S17: Kinetic traces at a probe wavelength of 600 nm of 4,4′-Czbpy and 5,5′-Czbpy in DMSO. 345 nm pump.

Time-Dependent Density Functional Theory Studies (TD-DFT)

Computational Details

Density functional theory calculations were performed with Gaussian 16. Geometry optimization, frequency, and single-point energy calculations were performed at the same level of theory. All calculations were performed using the B3LYP[9] or CAM-B3LYP[9, 10] functionals with Grimme′s GD3 emperical dispersion correction[11] and 6-311+G(d,p) basis set.[12-15] Structures were optimized to stationary points and verified as local minima by performing frequency calculations and confirming the absence of imaginary frequencies. Electron densities, molecular orbitals, and molecular orbital diagrams were visualized with Chemissian. Extinction coefficient (in units of $M^{-1}cm^{-1}$) spectra were calculated by broadening the TD-DFT-calculated oscillator strength sticks with gaussian lineshape functions according to the equation.[16]

$$\varepsilon(\tilde{\upsilon}) = \sum_i 1.3062974 \cdot 10^8 \cdot \frac{f_i}{\sigma} \exp\left[-\left(\frac{(\tilde{\upsilon} - \tilde{\upsilon}_i)}{\sigma}\right)^2\right]$$

where f_i and $\tilde{\upsilon}_i$ are the oscillator strength and transition frequency (in cm^{-1}) of the *i*-th transition, and σ is the gaussian width (standard deviation), which was taken to be 0.3 eV here. To achieve agreement with experimental absorption spectra, the CAM-B3LYP transition energies were shifted by -0.5 eV (prior to broadening). Difference spectra were obtained by subtracting the broadened "ground-state" (lowest singlet, S0) spectrum from the "excited state" (lowest triplet, T1) spectrum, simulating the pump-on minus pump-off spectra of optical transient absorption experiments.

Full Gaussian Citation:

Gaussian 16, Revision C.01, M. J. Frisch, G. W. Trucks, H. B. Schlegel, G. E. Scuseria, M. A. Robb, J. R. Cheeseman, G. Scalmani, V. Barone, G. A. Petersson, H. Nakatsuji, X. Li, M. Caricato, A. V. Marenich, J. Bloino, B. G. Janesko, R. Gomperts, B. Mennucci, H. P. Hratchian, J. V. Ortiz, A. F. Izmaylov, J. L. Sonnenberg, D. Williams-Young, F. Ding, F. Lipparini, F. Egidi, J. Goings, B. Peng, A. Petrone, T. Henderson, D. Ranasinghe, V. G. Zakrzewski, J. Gao, N. Rega, G. Zheng, W. Liang, M. Hada, M. Ehara, K. Toyota, R. Fukuda, J. Hasegawa, M. Ishida, T. Nakajima, Y.

Honda, O. Kitao, H. Nakai, T. Vreven, K. Throssell, J. A. Montgomery, Jr., J. E. Peralta, F. Ogliaro, M. J. Bearpark, J. J. Heyd, E. N. Brothers, K. N. Kudin, V. N. Staroverov, T. A. Keith, R. Kobayashi, J. Normand, K. Raghavachari, A. P. Rendell, J. C. Burant, S. S. Iyengar, J. Tomasi, M. Cossi, J. M. Millam, M. Klene, C. Adamo, R. Cammi, J. W. Ochterski, R. L. Martin, K. Morokuma, O. Farkas, J. B. Foresman, and D. J. Fox, Gaussian, Inc., Wallingford CT, 2019.

The optimized geometries of the following molecules are given at the very end of the Supporting Information document.

4,4′-Czbpy (neutral, spin singlet)
4,4′-Czbpy (neutral, spin triplet)
5,5′-Czbpy (neutral, spin singlet)
5,5′-Czbpy (neutral, spin triplet)
Ni(5,5′-Czbpy)Cl (neutral, spin doublet)
Ni(4,4′-Czbpy)Cl (neutral, spin doublet)
Ni(5,5′-Czbpy)Cl_2 (neutral, spin triplet)
Ni(4,4′-Czbpy)Cl_2 (neutral, spin triplet)
Ni(4,4′-Czbpy)Br_2 (neutral, spin triplet)
4,4′-Czbpy (neutral, spin singlet)
4,4′-Czbpy (neutral, spin triplet)
5,5′-Czbpy (neutral, spin singlet)
5,5′-Czbpy (neutral, spin triplet)

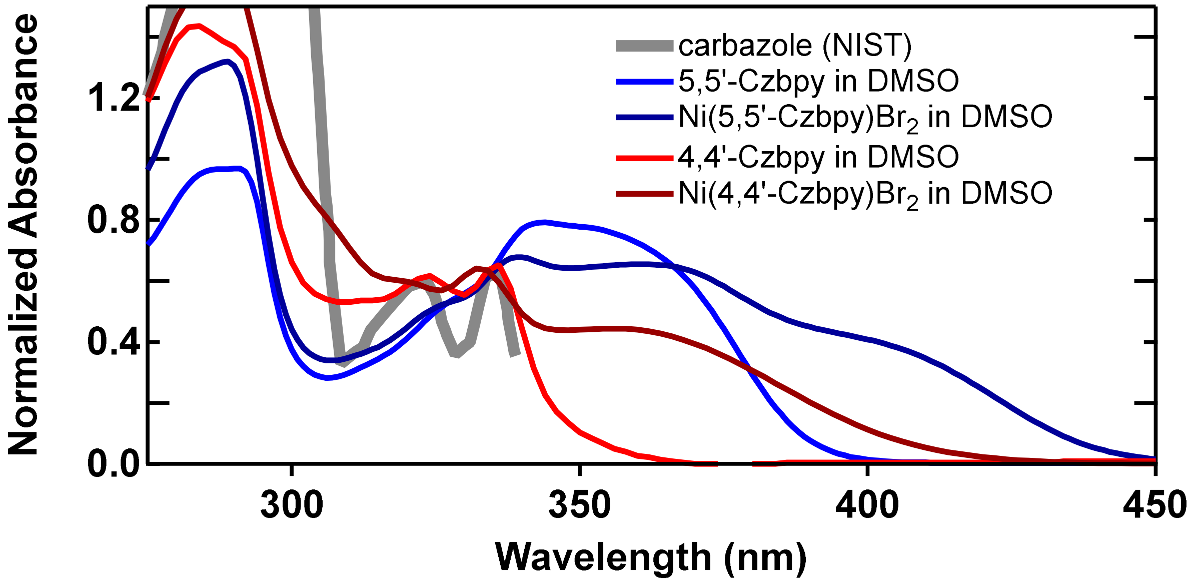

Fig. S18: UV-Vis absorption spectra of 5,5′- and 4,4′-Czbpy ligands and $NiBr_2$ complexes compared with the absorption spectrum of carbazole.

As shown in UV-vis spectra (Fig. S18), the Czbpy ligands and NiX_2 complexes show a vibronically resolved band at ~330 nm. Comparison to the spectrum of carbazole[17,18] demonstrates that this is a carbazole-centered transition that is sufficiently localized to not be affected by 4,4′ vs 5,5′ substitution on the bipyridine (bpy) or by the coordination of NiX_2 to the ligand. This carbazole (Cz)-centered π-π* transition, as well as the low-energy Cz intraligand charge transfer (ILCT) transition, were identified in TD-DFT calculations and used to calibrate the rigid-shift in transition energies required to achieve the absolute energy agreement between theory and experiment. Through these comparisons, we found that a -0.5 eV shift in transition energies was necessary for the CAM-B3LYP calculations, while no shift was required for the B3LYP calculations. The B3LYP calculations overestimated the 4,4′-Czbpy transition energies while underestimating the 5,5′-Czbpy transition energies (Fig. S29)

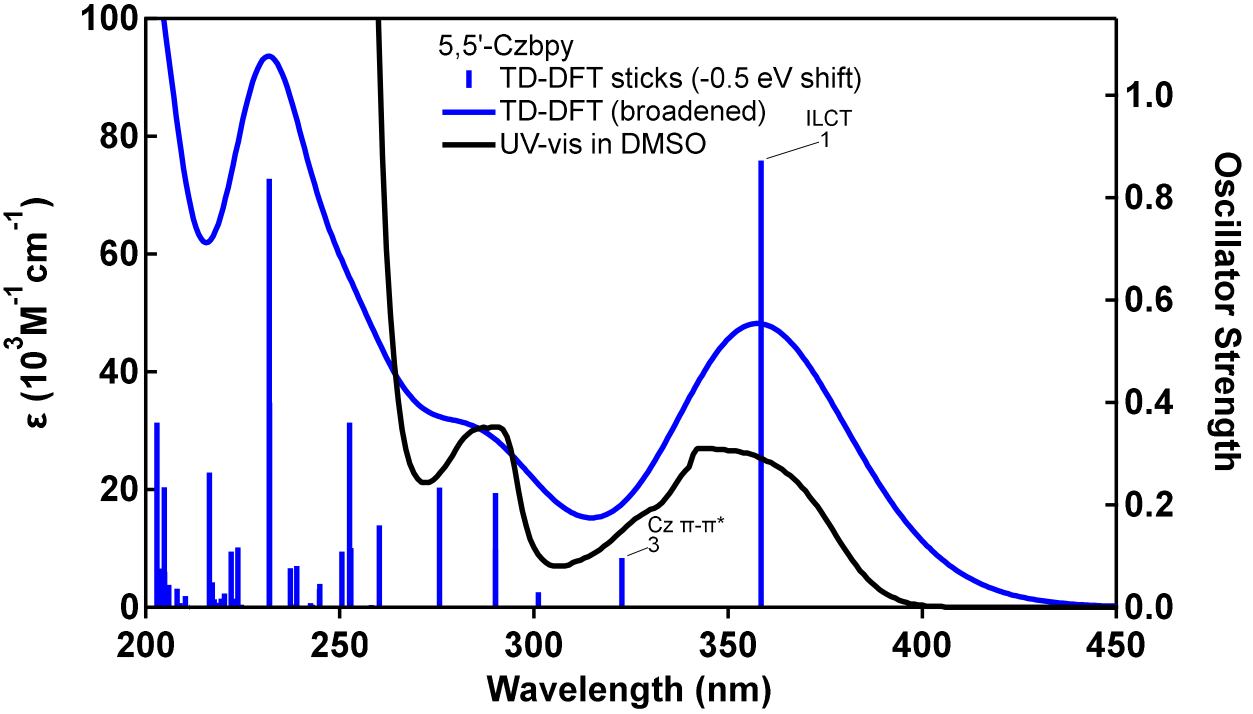

Fig. S19: Comparison of experimental UV-vis with TD-DFT of 5,5′-Czbpy. CAM-B3LYP/6-311+G(d,p) with GD3 empirical dispersion. UV-vis: 5 μM of 5,5′-Czbpy in DMSO in 1 cm cuvette.

1 3.958 eV 313.24 nm 358.53 nm (-0.5 eV shift) f= 0.873

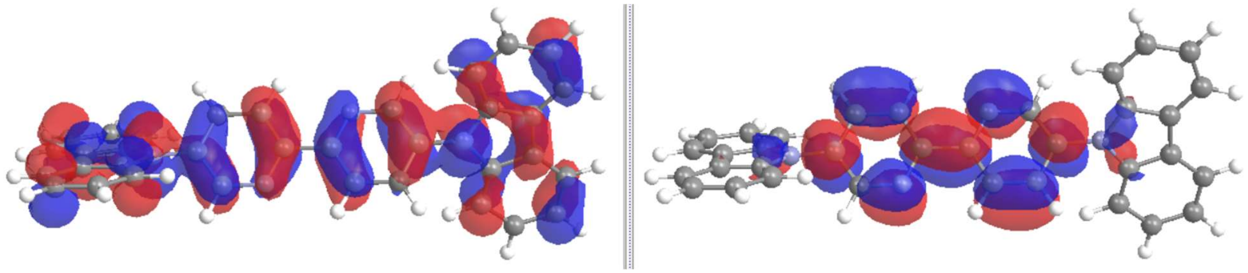

3 4.342 eV 285.52 nm 322.67 nm (-0.5 eV shift) f = 0.0962

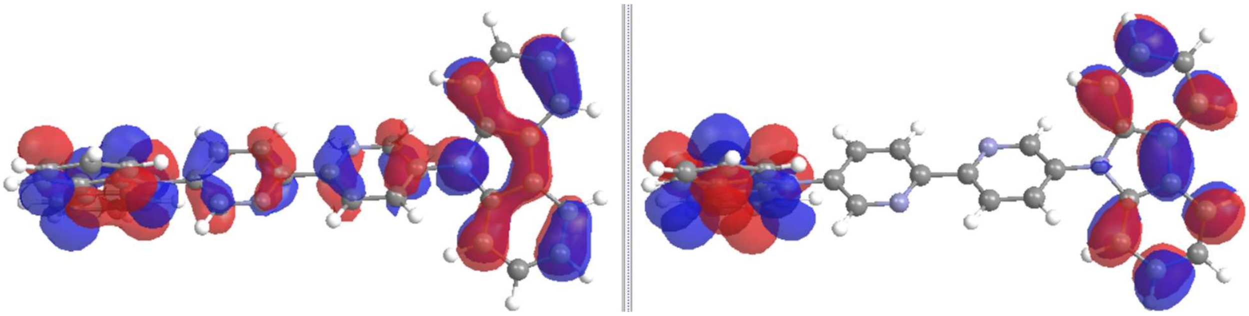

Fig. S20: Natural transition orbitals (0.02 isovalue) of bright, low-energy, ILCT and carbazole-centered π-π* TD-DFT transitions of spin-singlet 5,5′-Czbpy. CAM-B3LYP/6-311+G(d,p) with GD3 empirical dispersion.

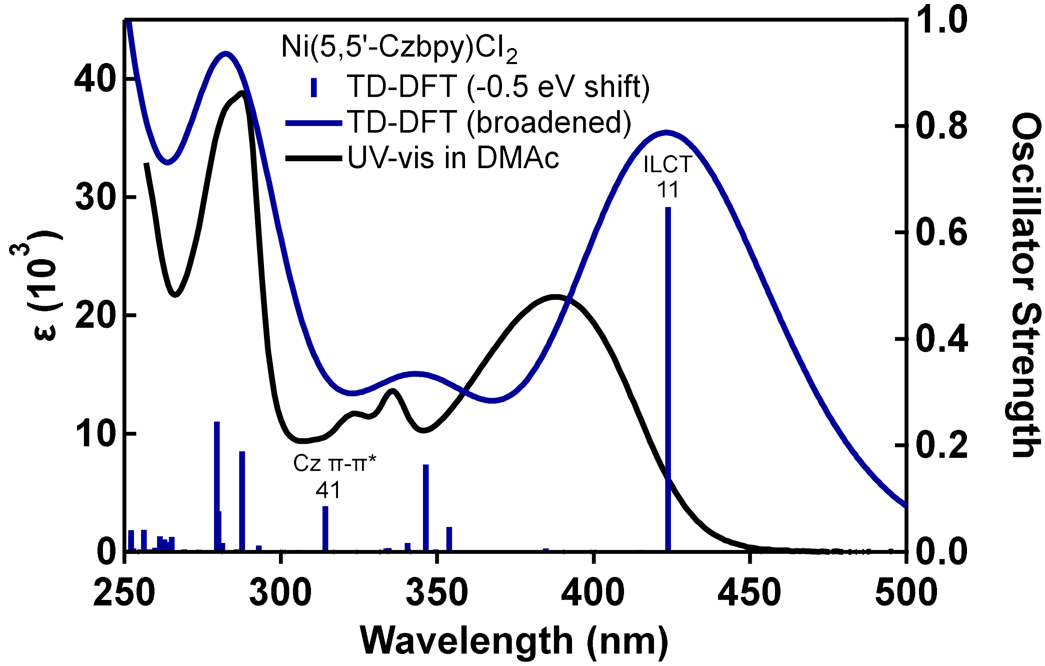

Fig. S21: Comparison of experimental UV-vis with TD-DFT of Ni(5,5′-Czbpy)Cl_2. CAM-B3LYP/6-311+G(d,p) with GD3 empirical dispersion. UV-Vis: 20 μM 1:1 mixture of 5,5′-Czbpy and $NiCl_2$•glyme in DMSO in 1 cm cuvette.

41 4.446 eV 278.88 nm 314.22 nm (-0.5 eV shift) f = 0.0858

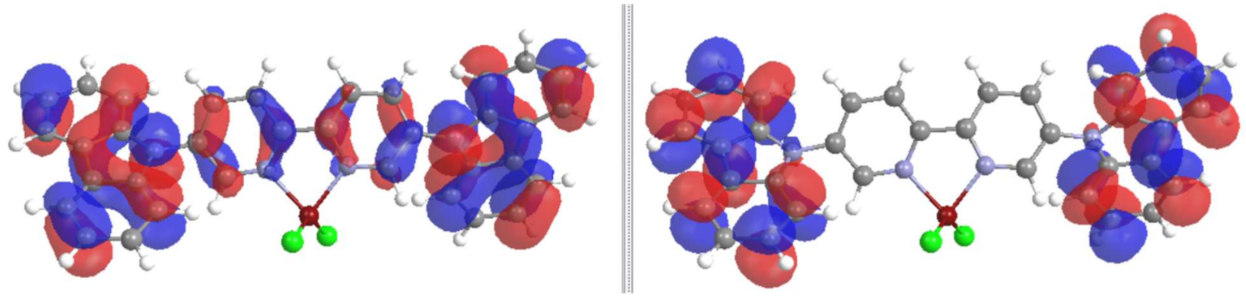

11 3.426 eV 361.94 nm 423.80 nm (-0.5 eV shift) f = 0.6481

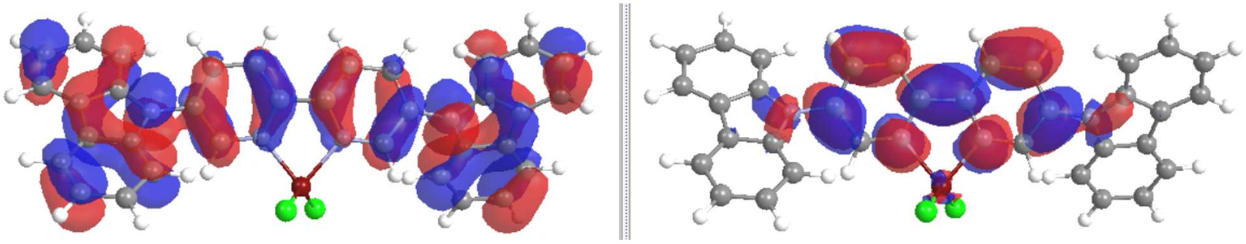

Fig. S22: Natural transition orbitals (0.02 isovalue) of bright, low-energy, ILCT and carbazole-centered π-π* TD-DFT transitions of spin-triplet Ni(5,5′-Czbpy)Cl_2. CAM-B3LYP/6-311+G(d,p) with GD3 empirical dispersion.

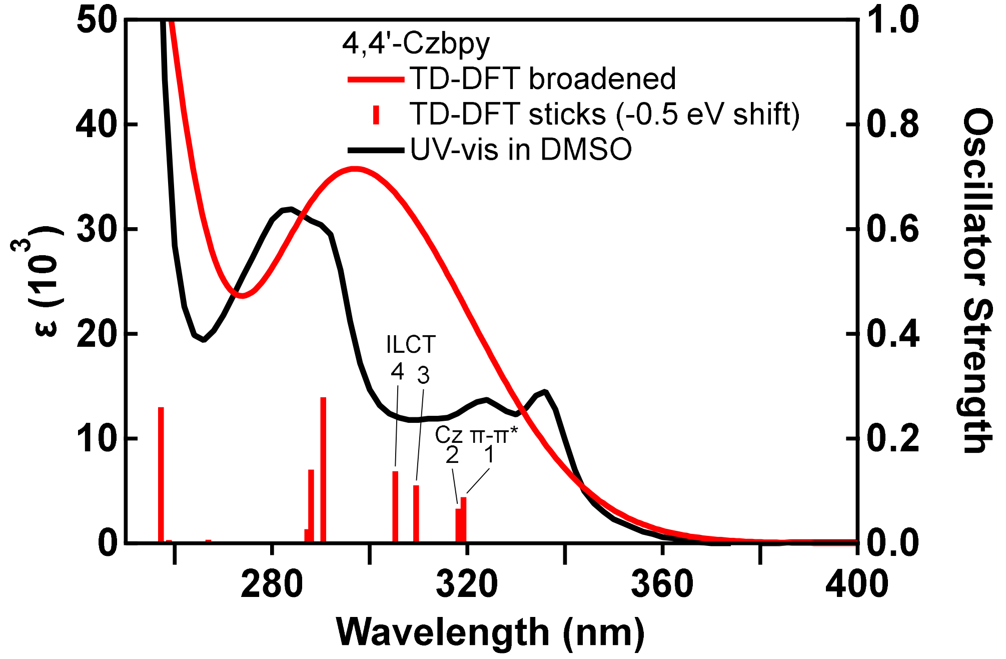

Fig. S23: Comparison of experimental UV-Vis with TD-DFT of 4,4′-Czbpy. CAM-B3LYP/6-311+G(d,p) with GD3 empirical dispersion. UV-Vis: 10 μM 4,4′-Czbpy in DMSO in 1 cm cuvette.

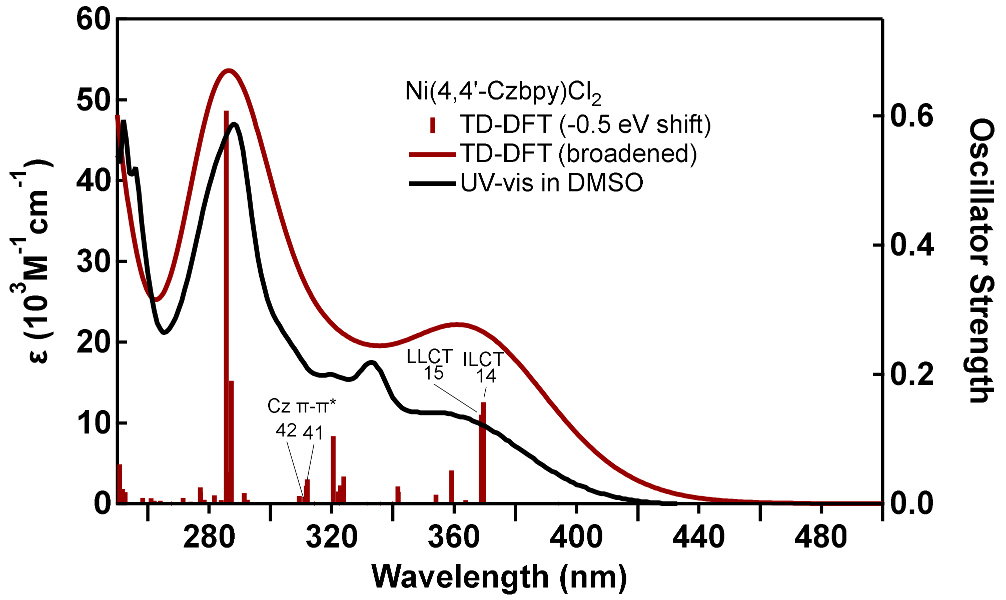

Fig. S24: Comparison of experimental UV-vis with TD-DFT of Ni(4,4′-Czbpy)Cl_2. CAM-B3LYP/6-311+G(d,p) with GD3 empirical dispersion. UV-vis: 8 μM 1:1 mixture of 4,4′-Czbpy and $NiCl_2$•glyme in DMSO in 1 cm cuvette.

1 4.384 eV 282.82 nm 319.23 nm (-0.5 eV shift) f = 0.0882

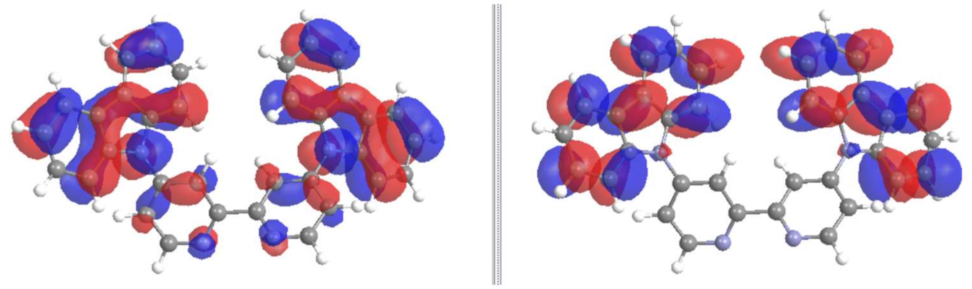

2 4.397 eV 282.01 nm 318.19 nm (-0.5 eV shift) f = 0.0662

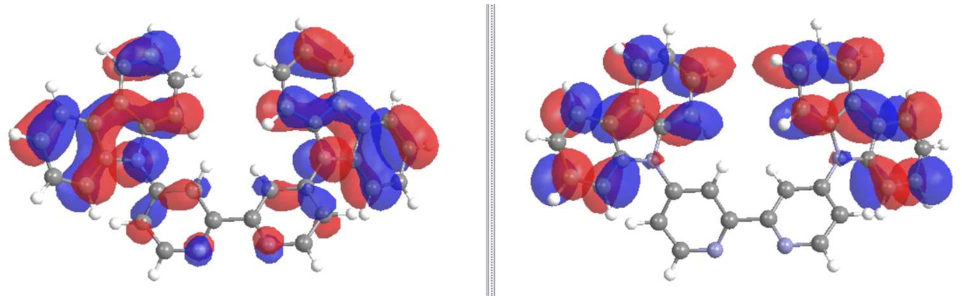

3 4.506 eV 275.14 nm 309.48 nm (-0.5 eV shift) f = 0.111

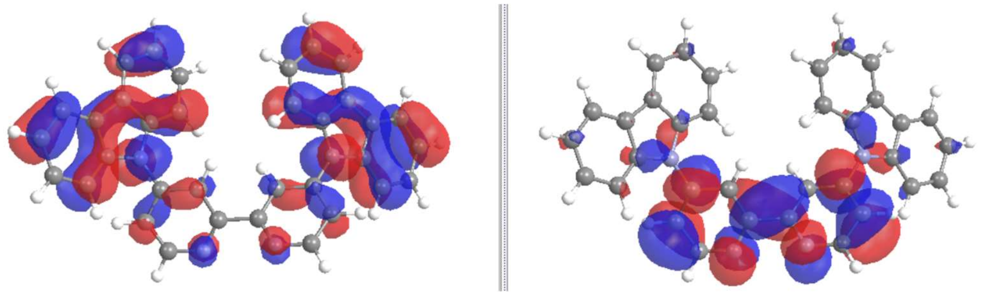

4 4.562 eV 271.79 nm 305.25 nm (-0.5 eV shift) f = 0.1381

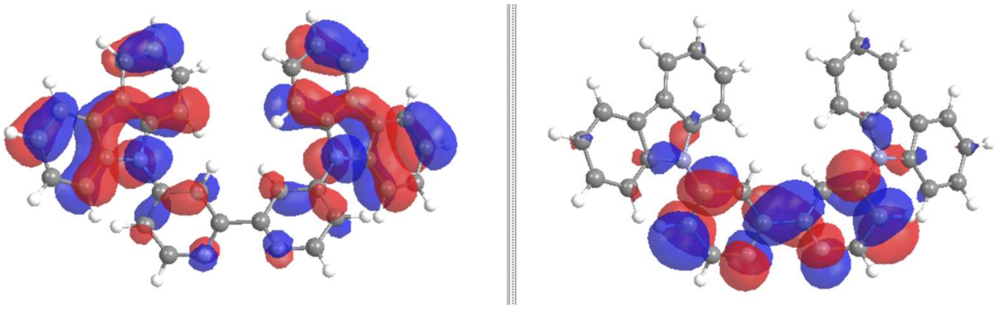

Fig. S25: Natural transition orbitals (0.02 isovalue) of bright, low-energy, ILCT and carbazole-centered π-π* TD-DFT transitions of spin-singlet 4,4′-Czbpy. CAM-B3LYP/6-311+G(d,p) with GD3 empirical dispersion.

14 3.855 eV 321.60 nm 369.53 nm (-0.5 eV shift) f = 0.1568

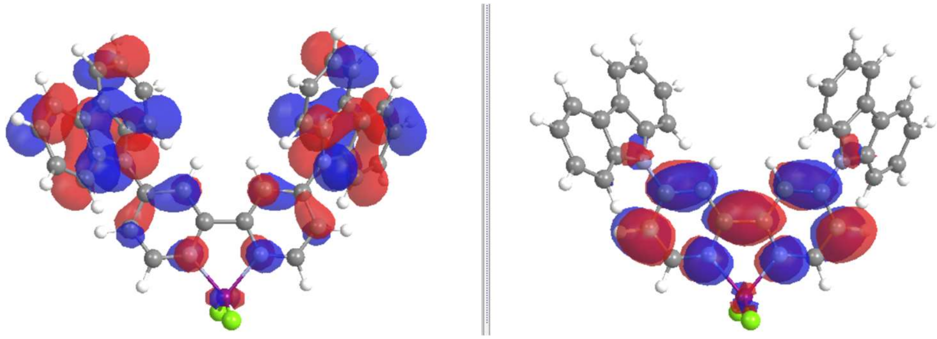

15 3.86 eV 321.09 nm 368.86 nm (-0.5 eV shift) f = 0.1377

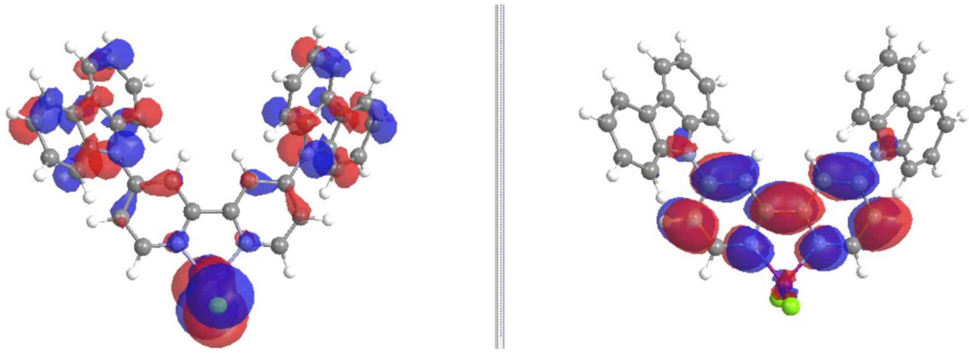

41 4.473 nm 277.18 nm 312.07 nm (-0.5 eV shift) f = 0.0378

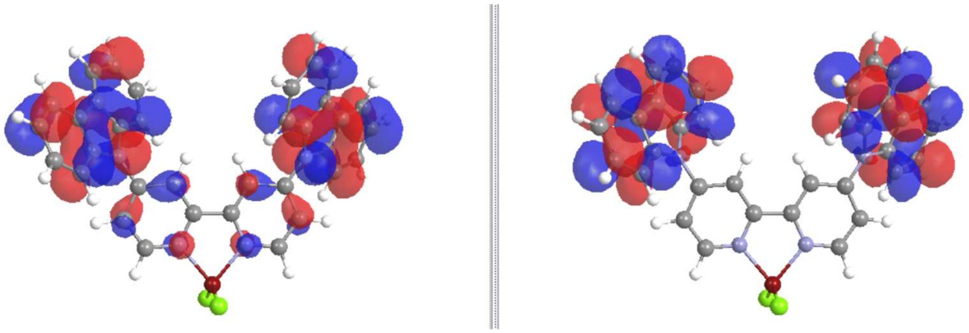

42 4.477 eV 276.92 nm 311.73 nm (-0.5 eV shift) f = 0.0277

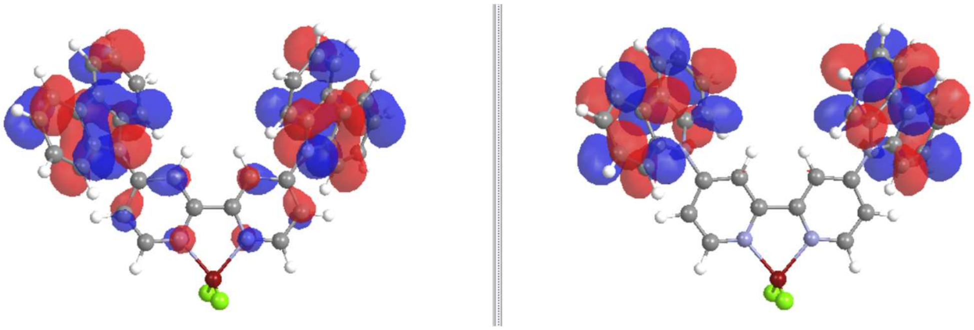

Fig. S26: Natural transition orbitals (0.02 isovalue) of bright, low-energy, ILCT and carbazole-centered π-π* TD-DFT transitions of spin-triplet Ni(4,4′-Czbpy)Cl_2. CAM-B3LYP/6-311+G(d,p) with GD3 empirical dispersion.

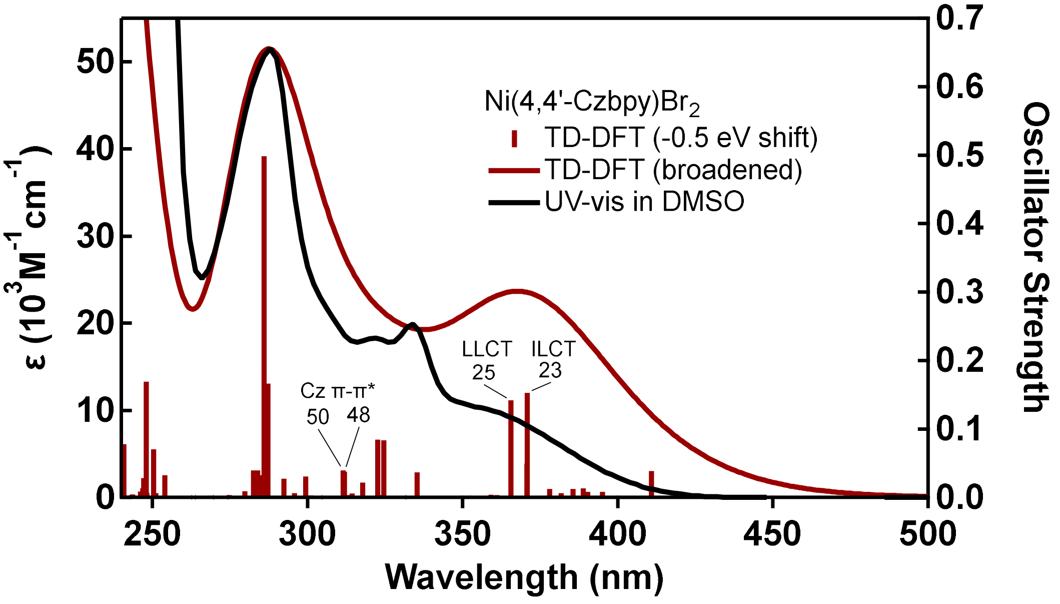

Fig. S 27: Comparison of experimental UV-vis with TD-DFT of Ni(4,4′-Czbpy)Br_2. CAM-B3LYP/6-311+G(d,p) with GD3 empirical dispersion. UV-vis: 10 μM 1:1 mixture of 4,4′-Czbpy and $NiBr_2$•glyme in DMSO in 1 cm cuvette.

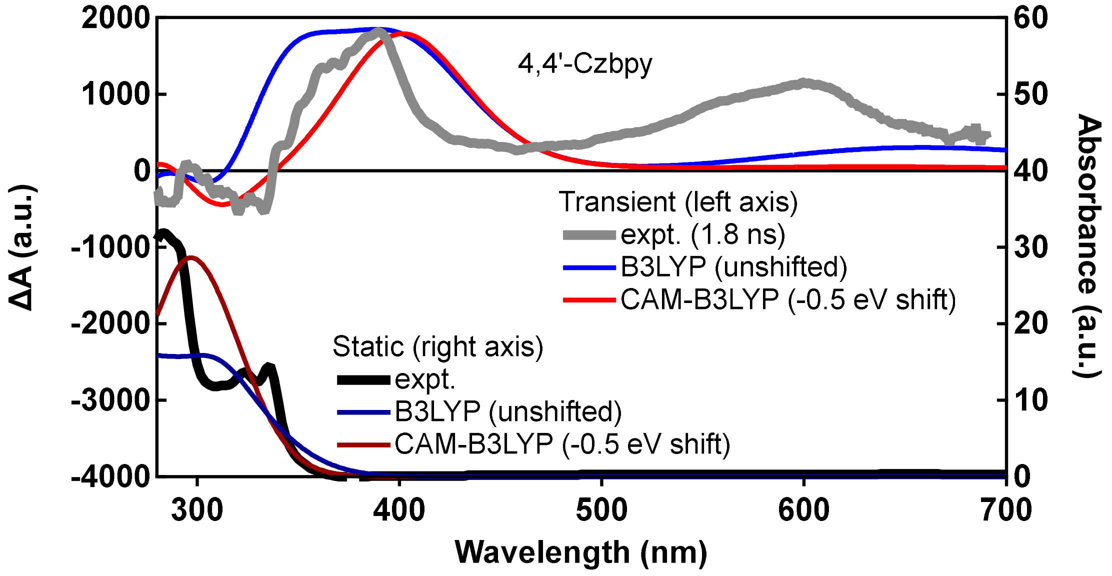

Fig. S28: Comparison of TD-DFT simulated transient spectra of 4,4′-Czbpy calculated with CAM-B3LYP and B3LYP functionals, both with 6-311+G(d) basis sets and GD3 dispersion. Transient spectra were calculated by taking the difference of the T1 and S0 TD-DFT spectra. Experimental spectra were measured in DMSO.

TD-DFT calculations with the CAM-B3LYP functional on the T1 state of 4,4′-Czbpy did not reproduce the excited-state absorption observed at ~600 nm in the experimental OTA. We therefore redid the calculations with the B3LYP functional, which showed increased ESA intensity (at ~650 nm), better reproducing the ESA feature observed experimentally (Fig. S29). As shown by the spin density plots and SOMOs below, while the T1 state of 4,4′-Czbpy calculated by CAM-B3LYP and B3LYP have the same electron configuration, CAM-B3LYP shows less ILCT character than B3LYP. While the B3LYP functional faithfully reproduced the 4,4′-Czbpy transient spectrum, TD-DFT with this functional underestimated the transition energies of 5,5′-Czbpy (Fig. S29).

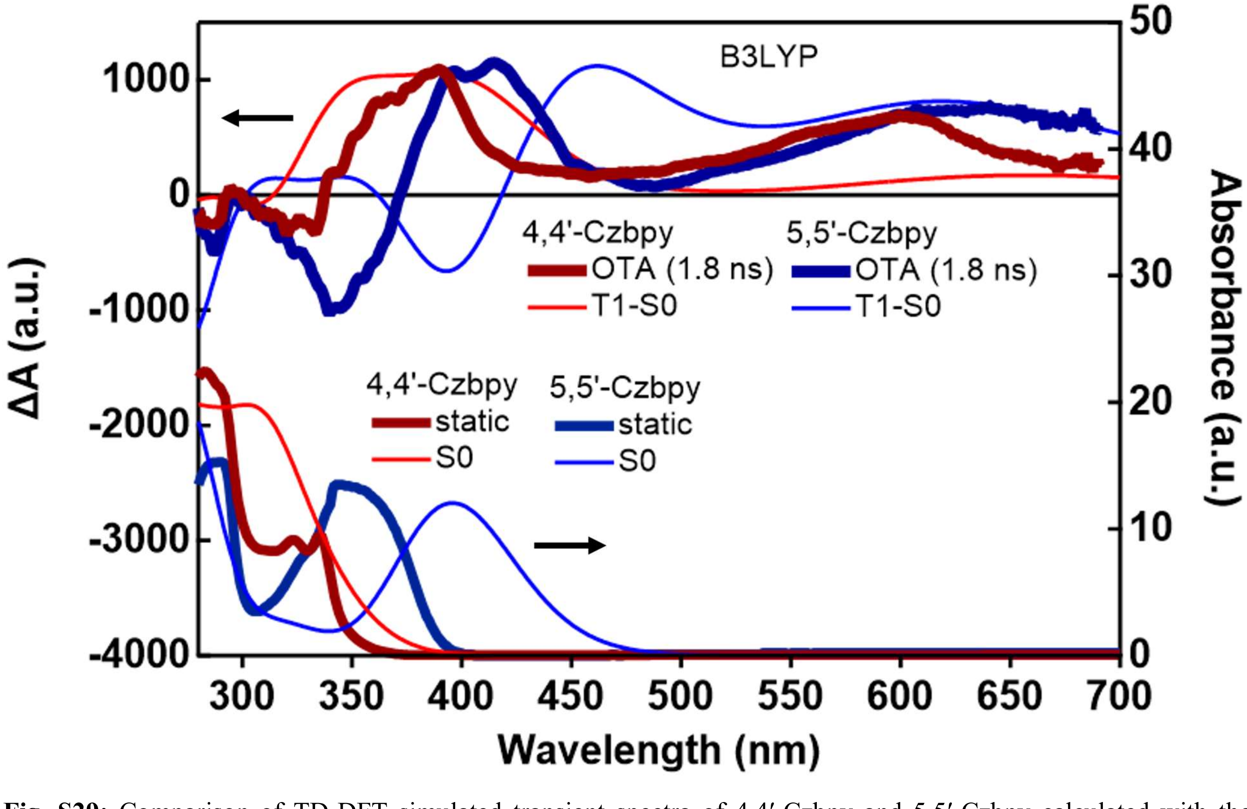

Fig. S29: Comparison of TD-DFT simulated transient spectra of 4,4′-Czbpy and 5,5′-Czbpy calculated with the B3LYP-GD3/6-311+G(d). Transient spectra were calculated by taking the difference of the T1 and S0 TD-DFT spectra. Experimental spectra were measured was taken in DMSO. Arrows indicate which axis the traces are plotted on (transient spectra on left axis, static spectra on right axis).

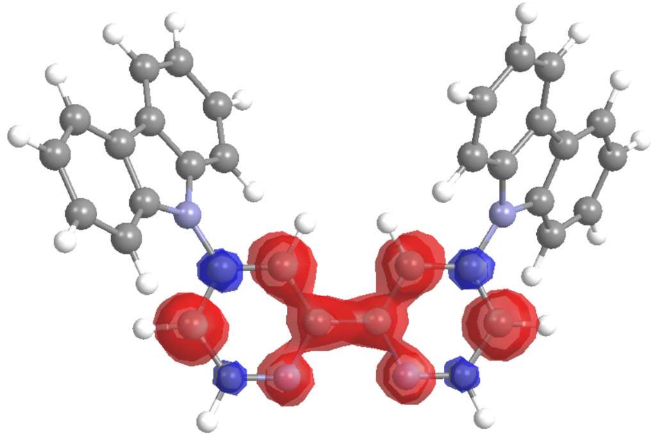

Fig. S30: Spin density plot (0.005 isovalue) of 4,4′-Czbpy triplet state. Red = alpha spin; blue = beta spin. CAM-B3LYP/6-311+G(d,p) with GD3 empirical dispersion.

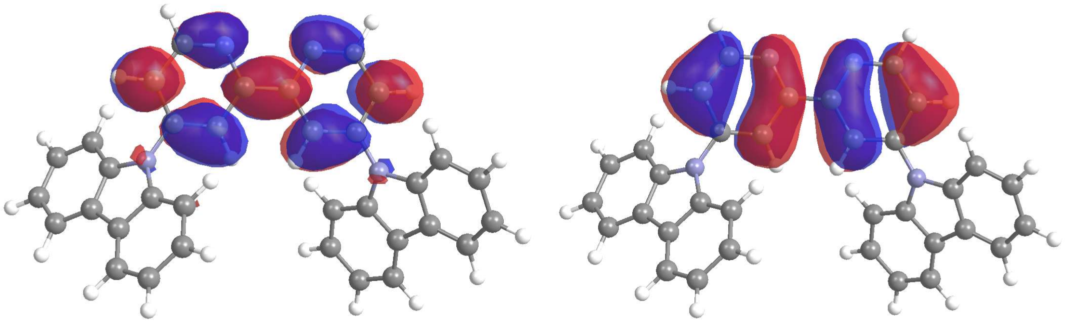

Fig. S31: SOMOs (0.02 isovalue) of 4,4′-Czbpy triplet state. Left: Alpha-spin HOMO. Right: beta-spin LUMO. CAM-B3LYP/6-311+G(d,p) with GD3 empirical dispersion.

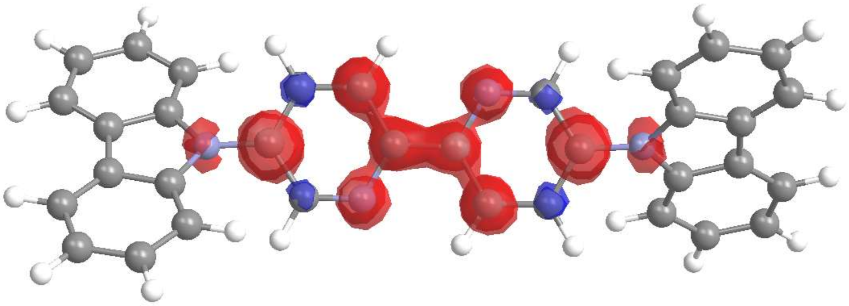

Fig. S32: Spin density plot (0.005 isovalue) of 5,5′-Czbpy triplet state. Red = alpha spin; blue = beta spin. CAM-B3LYP/6-311+G(d,p) with GD3 empirical dispersion.

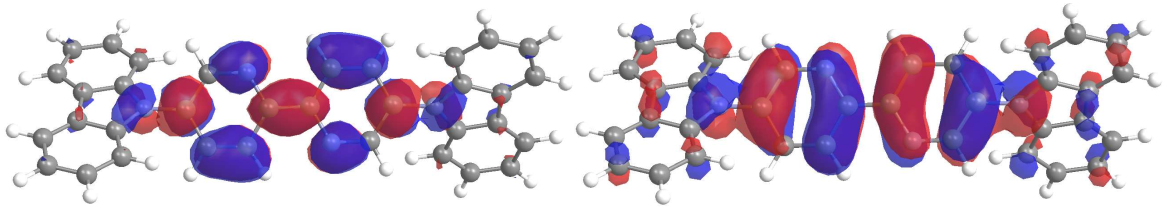

Fig. S33: SOMOs (0.02 isovalue) of 5,5′-Czbpy triplet state. Left: Alpha-spin HOMO. Right: beta-spin LUMO. CAM-B3LYP/6-311+G(d,p) with GD3 empirical dispersion.

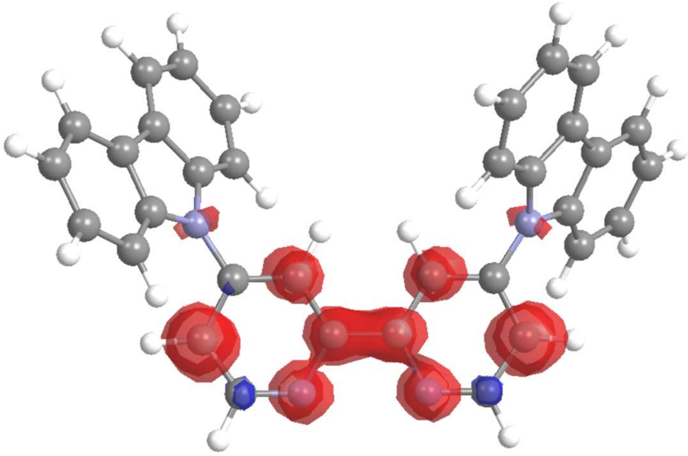

Fig. S34: Spin density plot (0.005 isovalue) of 4,4′-Czbpy triplet state. Red = alpha spin; blue = beta spin. B3LYP/6-311+G(d,p) with GD3 empirical dispersion.

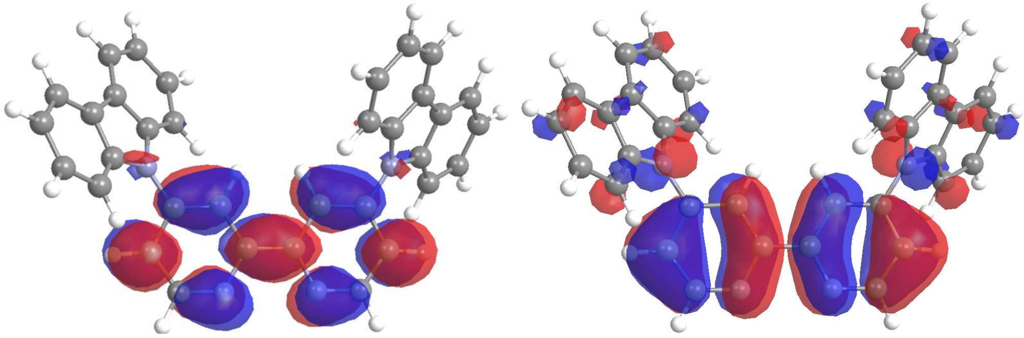

Fig. S35: SOMOs (0.02 isovalue) of 4,4′-Czbpy triplet state. Left: Alpha-spin HOMO. Right: beta-spin LUMO. B3LYP/6-311+G(d,p) with GD3 empirical dispersion.

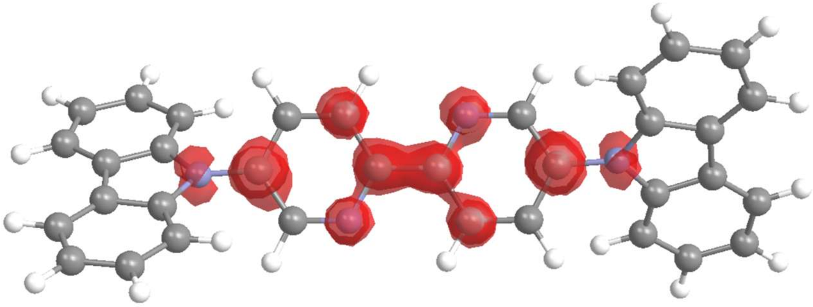

Fig. S36: Spin density plot (0.005 isovalue) of 5,5′-Czbpy triplet state. Red = alpha spin; blue = beta spin. B3LYP/6-311+G(d,p) with GD3 empirical dispersion.

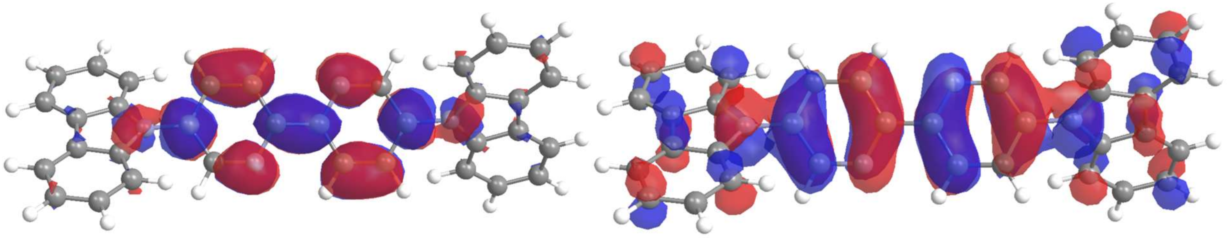

Fig. S37: SOMOs (0.02 isovalue) of 5,5′-Czbpy triplet state. Left: Alpha-spin HOMO. Right: beta-spin LUMO. B3LYP/6-311+G(d,p) with GD3 empirical dispersion.

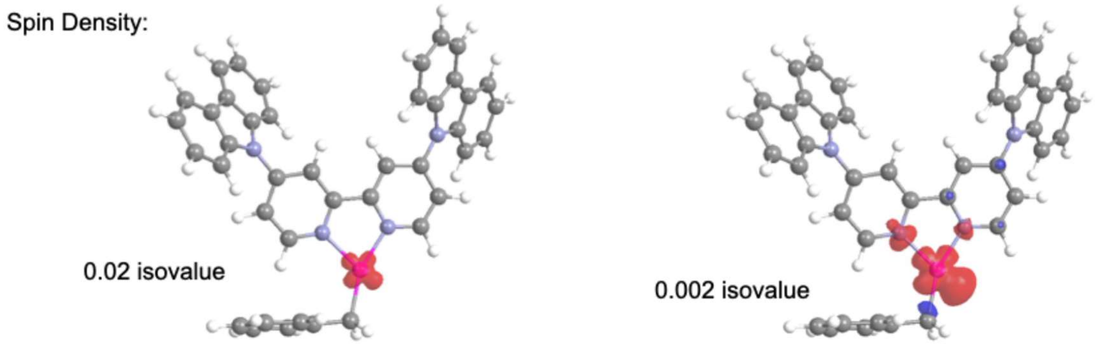

Natural Population Analysis:

Atom	Spin Population
Ni	0.96242
N	0.05156
N	0.02903
C	-0.00096

Fig. S38: Spin density and natural population analysis of the Ni(4,4'-czbpy)Bn complex. Spin population was calculated as the difference between the alpha-spin and beta-spin populations summed to the atoms through natural population analysis (Reed, A. E.; Weinstock, R. B.; Weinhold, F. Natural Population Analysis. *The Journal of Chemical Physics* **1985**, *83* (2), 735–746.). Spin density plots were calculated and visualized with Chemissian software.

EPR experiments

The low solubility of Ni(4,4′-Czbpy)Br_2 and the labile nature of the related Ni^I species did not allow for a direct detection of the catalytically active Ni^I species via EPR spectroscopy. Related Ni^I complexes are typically synthesized and studied using EPR by applying sterically encumbered ligands[19, 20] or electron-deficient bpy derivatives.[21] An EPR study of Ni^I complexes with bpy ligands that do not provide such stabilizing effects was recently performed at 5 K in 2Me-THF.[22] However, this study also showed that complexes that do not allow for sufficiently high concentrations at low temperatures do not enable EPR analysis. This is also the case for Ni(4,4′-Czbpy)Br_2, which generally suffers from low solubility in all the organic solvents tested.

As a consequence, we provide indirect evidence for Ni^I formation through a spin trap experiment using a mixture of Ni(4,4′-Czbpy)Br_2 and PBN in DMSO/toluene, analogous to a previous study.[23] The EPR spectrum resulting from illumination of Ni(4,4′-Czbpy)Br_2 and α-phenyl-N-tert-butyl nitron (PBN) in DMSO/toluene is characteristic for a spin adduct of a C- or S-centered radical (Fig. S38). This provides evidence that the bromine radical, which is generated upon Ni–Br bond fission, reacts with DMSO generating PBN-DMSO adducts.[24, 25]

Experimental procedure for spin trapping experiments

Samples for electron paramagnetic resonance (EPR) spin trapping experiments were prepared by dissolving 4,4′-Czbbpy and $NiBr_2$ in a mixture of DMSO/toluene (1:1) to yield a 2 mM concentrated solution of Ni(4,4′-Czbpy)Br_2. PBN was added, corresponding to a concentration of 20 mM. X-Band EPR measurements were performed on an Elexsys E580 spectrometer (Bruker Biospin) equipped with a critically coupled dielectric ring resonator (ER 4118X-MD5, Bruker Biospin).The sample was illuminated using a 405 nm laser diode (DL5146-101S, Thorlabs) at a power of (13±1) mW, measured before passing the optical window of the resonator. Spectra were recorded after different illumination times with the light source switched off. After 125 min, when no substantial changes in the signal intensity were observed, the final spectrum was recorded using a modulation frequency of 100 kHz, a modulation amplitude of 0.25 G, and a microwave power of 1.54 mW. The magnetic field was calibrated with a DPPH standard.

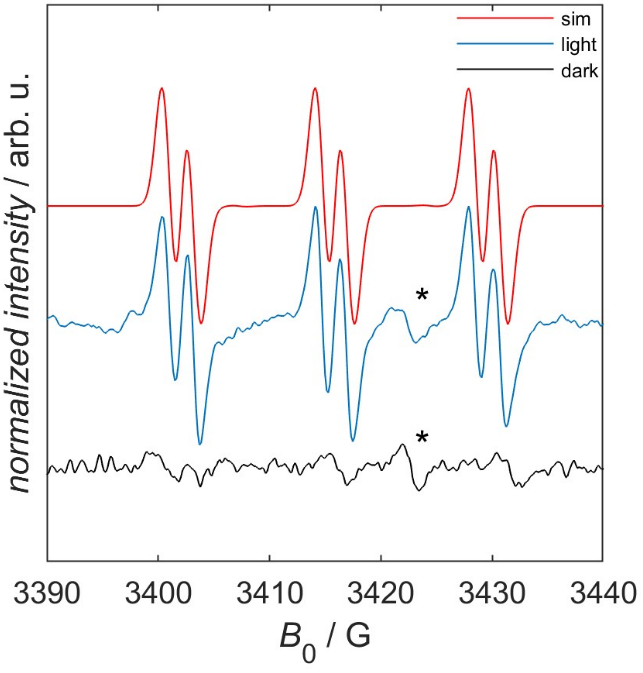

Fig. S39: Room temperature X-band CW-EPR spectra at 9.596 GHz of Ni(4,4′-Czbpy)Br_2 with added PBN in DMSO/toluene before (black) and after 125 min of illumination (blue). The simulation of the spectrum after illumination is shown in red. The signal marked with an asterisk at g = 2.0033 is assigned to a background signal.

Spectral simulations were performed with the EasySpin[26] toolbox for MATLAB based on the spin Hamiltonian

$$\widehat{H} = \mu_{\mathrm{B}} g \boldsymbol{B} \cdot \hat{S} + a_{\mathrm{N}} \hat{S} \cdot \hat{I}_{\mathrm{N}} + a_{\mathrm{H}} \hat{S} \cdot \hat{I}_{\mathrm{H}},$$

where g is the isotropic g value and $a_{N/H}$ are the isotropic hyperfine coupling constants of the ^{14}N and ^{1}H nuclei, respectively.

EPR simulation parameters (uncertainties in parenthesis)

S = ½

g = 2.0071 (1)

a_N = 13.8 (2) G

a_H = 2.06 (7) G

Gaussian linewidth = 1.5 (1) G

Spin quantification

Samples for the quantification of the product of spin trapping experiments were prepared by dissolving 4,4'-Czbbpy and $NiBr_2$ in a mixture of DMSO/toluene (1:1) to yield a 2 mM concentrated solution of Ni(4,4'-Czbpy)Br_2. α-phenyl-N-tert-butyl nitron (PBN) was added corresponding to a concentration of 20 mM. A calibration of the method was performed based on the stable radical TEMPOL (4-hydroxy-2,2,6,6-tetramethylpiperidin-1-oxyl) as a spin standard. Therefore, three standard solutions at a concentration of 3 mM in DMSO/toluene (1:1) were prepared and diluted accordingly to prepare standards over the expected range of concentrations to calculate a calibration curve. The area under the absorption signal was calculated by double integration after a polynomial baseline correction and served as a measure of the concentration.
The X-band EPR measurements were performed at room temperature using an Elexsys E580 spectrometer (Bruker Biospin) equipped with a critically coupled Super High-Q resonator (ER 4122SHQE-W1, Bruker Biospin) at a frequency of 9.872 GHz. The sample was illuminated for 90 minutes using two 440 nm LED (Kessil PR160L-440) before transferring 10 µL of the sample solution into the quartz sample tube which was then placed in the spectrometer. For measurements of the spin standard solutions (TEMPOL) 10 µL was used as well without prior illumination. Spectra were recorded at different power between 0.015 and 40 mW to check for saturation. A modulation frequency of 100 kHz and a modulation amplitude between 0.25 and 2.0 G were used, as indicated in the results.

Calibration of the Method

Power saturation experiment: To evaluate the optimal MW power for the calibration, the spectrum of a 3mM solution of TEMPOL was recorded at different power and evaluated for saturation of the signal.

Table S1: Calculated double integrals of the spectrum of TEMPOL (3 mM solution) at different MW power.

MW Power / mW	Double integral / arb. u.
0.015	47.2
0.024	55.8
0.038	67.2
0.061	92.8
0.097	109.9
0.154	148.1
0.244	180.3
0.386	233.5
0.612	282.7
0.969	364.2
1.54	454.1
2.44	572.1
3.86	713.0
6.12	892.9
9.7	1119
15 .4	1412
24.4	1773
38.3	2207

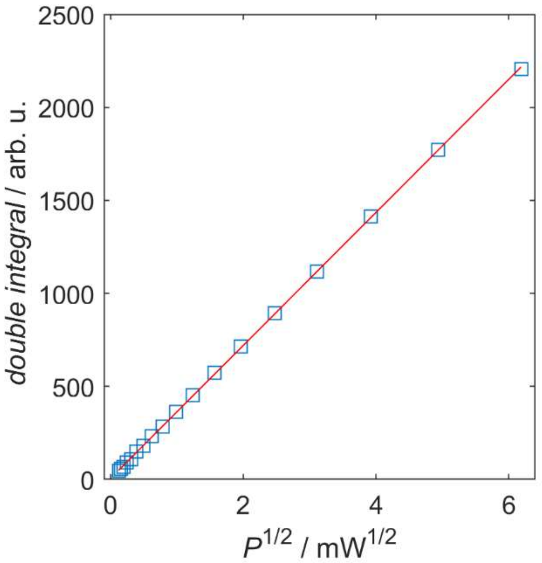

Fig. S40: EPR intensity evaluated as the double integral of a 3 mM solution of TEMPOL in DMSO/toluene at different MW power. Modulation amplitude: 1 G.

The power saturation experiment of the TEMPOL solution shows a linear behavior (R^2 = 0.9999) over the whole probed power range. Based on this, a microwave power of 3.86 mW ($P^{1/2}$ = 1.96 $mW^{1/2}$) was chosen for the following experiments.

Standard curve

The determined double integrals of the three prepared standard solutions and their dilutions are listed below. As the quality factor Q slightly varied between the measurements, the DI was normalized to Q.

Standard solution A and dilutions

	C / mM	Q	Double integral (DI) / arb. u.	DI/Q
A,1	2.9	1700	161.9	0.0952
A,2	0.96	1800	52.02	0.0289
A,3	0.19	1700	10.08	0.00593
A,4	0.048	1900	2.65	0.00139

Standard solution B and dilutions

	C / mM	Q	Double integral (DI) / arb. u.	DI/Q
B,1	2.9	1700	173.2	0.10188
B,2	0.96	1800	60.57	0.03365
B,3	0.19	1800	12.54	0.00697
B,4	0.048	2000	2.61	0.00131

Standard solution C and dilutions

	C / mM	Q	Double integral (DI) / arb. u.	DI/Q
C,1	2.96	1700	176.2	0.10365
C,2	0.98	1800	57.97	0.03221
C,3	0.196	1800	12.44	0.00691
C,4	0,049	2000	2,32	0,00116

The average of the double integrals for each concentration was calculated, where slight differences between the concentrations of the three standard solutions were neglected.

Table S2: Average of double integral of three different TEMPOL standard solutions at different concentration.

	C /mM	DI	DI/Q
1	2.9	170.433	0.100254
2	0.96	56.8533	0.03159
3	0.19	11.6867	0.00660
4	0.048	2.52667	0.00129
5	0.0096	0.60417	0.000313

The fit result shows a linear behavior of the calculated double integrals as a function of the probed range of concentrations (slope: 0.03458, intercept: − 0.0004005, R^2 = 0.9997).

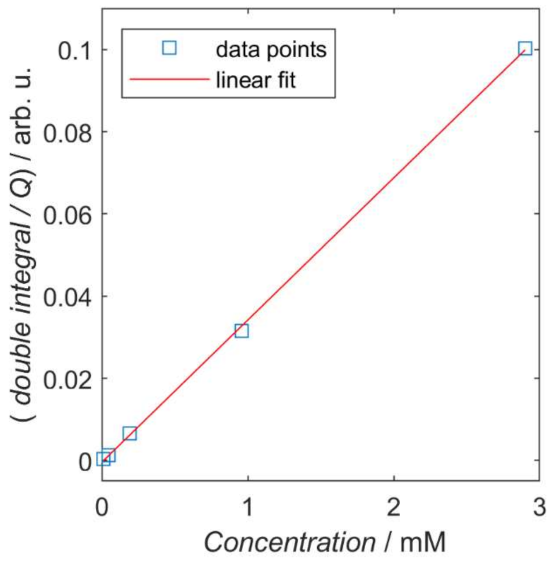

Fig. S41: Standard curve of a solution of TEMPOL in DMSO/toluene recorded at a MW power of 3.86 mW and a modulation amplitude of 0.25 G.

Determination of trapped spin concentration of illuminated (4,4'-Czbpy)Ni(II)Br_2

Power saturation

To evaluate the optimal MW power for the spin quantification experiment, the spectrum was recorded at different power and evaluated for saturation. Figure S42 shows the calculated double integrals as a function of the MW power together with a linear fit (red line). The linear model fits the data with an R^2 of 0.8236. Although no clear saturation regime could be detected, the scattering of data points increased drastically at higher MW power. The data points up to $P = 10$ mW ($P^{1/2}$ = 3.18 $mW^{1/2}$) however do not deviate much from the linear fit. Therefore, the following experiments could be performed with the same MW power as in the calibration experiments of $P = 3.86$ mW ($P^{1/2} = 2$ $mW^{1/2}$) which lies well in the linear regime.

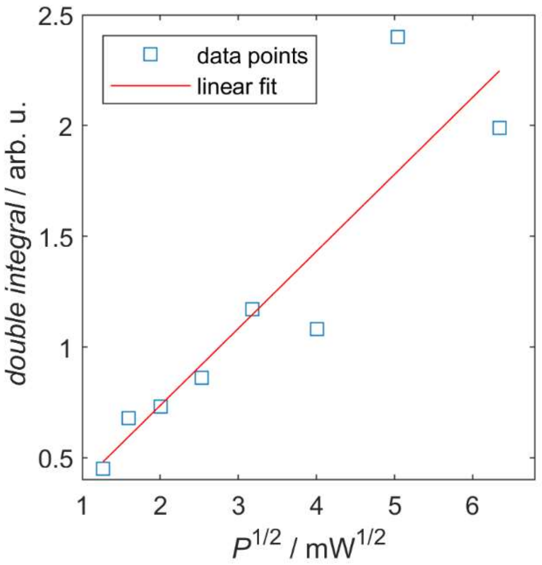

Fig. S42: EPR intensity evaluated as the double integral of a (4,4'-Czbpy)NiBr_2 solution in DMSO/toluene with added PBN after 90 min. of illumination (440nm) at different MW power. Spectra were recorded with a modulation amplitude of 1 G.

A representative EPR signal of the sample solution after 90 min of illumination recorded at a modulation amplitude of 1 and 2 G is shown in Figure S43 in the lower and upper trace, respectively. The higher modulation amplitude used here, as compared to that for the TEMPOL samples, was applied to increase the signal intensity. This was accepted by the expense of a line shape distortion especially pronounced using a 2 G modulation. However, the overmodulation does not affect the value of the double integral, which is linearly proportional to the modulation amplitude (See G. R. Eaton, S. S. Eaton, D. P. Barr, R. T. Weber, *Quantitative EPR,* 2010 (Springer)). Note that the signal-to-noise ratio is generally weaker as compared to the former trapping experiments using a 405 nm LASER for illumination, indicating that only a minor fraction of the sample was converted to Ni(I) and subsequently trapped by PBN according to the proposed trapping mechanism (*vide supra*).

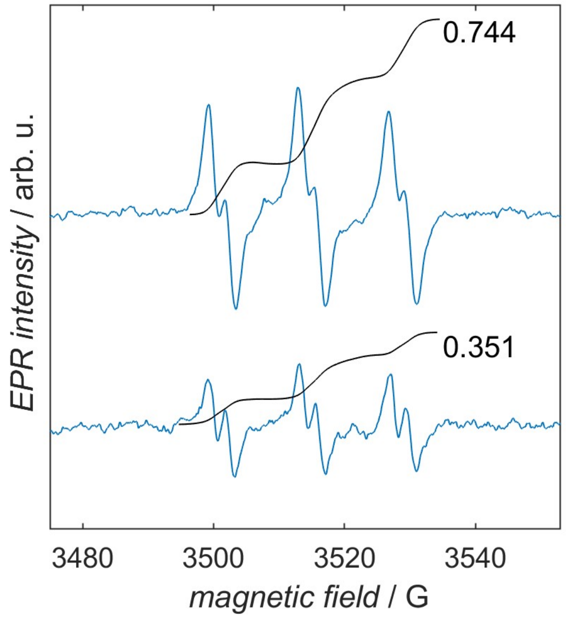

Fig. S43: EPR signal of the (4,4'-Czbpy)NiBr$_2$ solution with added PBN after 90 min of illumination at 440 nm. The lower and upper trace shows the signal at a modulation amplitude of 1 G and 2 G, respectively. The corresponding double integral is shown superimposed in black. The spectra were recorded at a MW power of 3.86 mW.

Quantification of the PBN adduct

To quantify the EPR signal of the PBN adduct the double integral was calculated. The values are shown in Fig S42 superimposed on the EPR signals. The value of the double integral is proportional to the strength of the modulation that must be considered while calculating the concentration. The results below show the calculated final concertation of the PBN adduct for both measurements. The calculated concentrations ranges between 0.0030 and 0.0044 mM, which is 0.15 – 0.22 % of in initial Ni(4,4'-Czbpy)Br$_2$ concentration. The slightly different results arise from the integration error, which is, at the low edge of the calibration curve, estimated as the major source of uncertainty in the result.

Table S3: Results of the quantification of the PBN adduct. Double integrals (DI) were normalized to *Q* and the final concertation was calculated by the calibration curve (see above) and normalized by the value of the modulation amplitude.

C(Ni(Czbpy)Br$_2$) / mM	DI	Q	DI/Q	modulation amplitude / G	*C*(PBN adduct) / mM
2.0	0.351	1700	0.00020618	1	0.0044
2.0	0.744	1700	0.00043782	2	0.0030

EPR spectra of Ni(4,4'-Czbpy)Br_2

The following experiments Fig. S44 - S46 were performed with the same experimental setup as the initial Spin trap experiment (Fig S39).

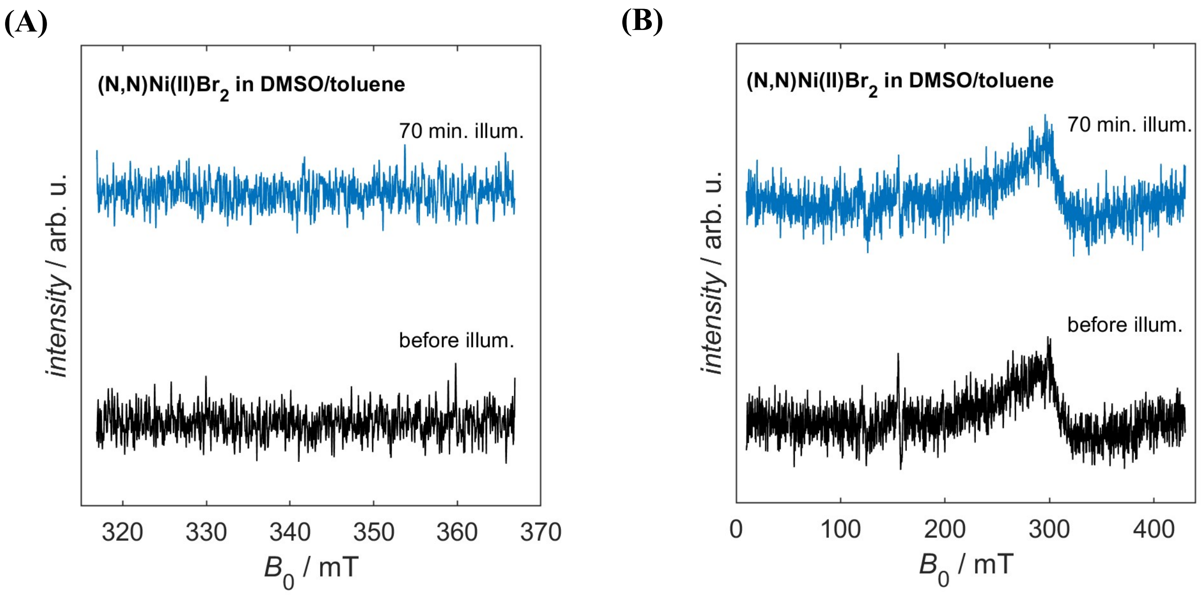

Fig. S44: Room temperature X-band EPR spectra at 9.603 GHz of Ni(4,4'-Czbpy)Br_2 in DMSO/toluene (around 2 mM) before (black) and after illumination (blue). (A) Narrow field scan around the g = 2 region. (B) Broad field scan.

Ni(4,4'-Czbpy)Br_2 + ArBr

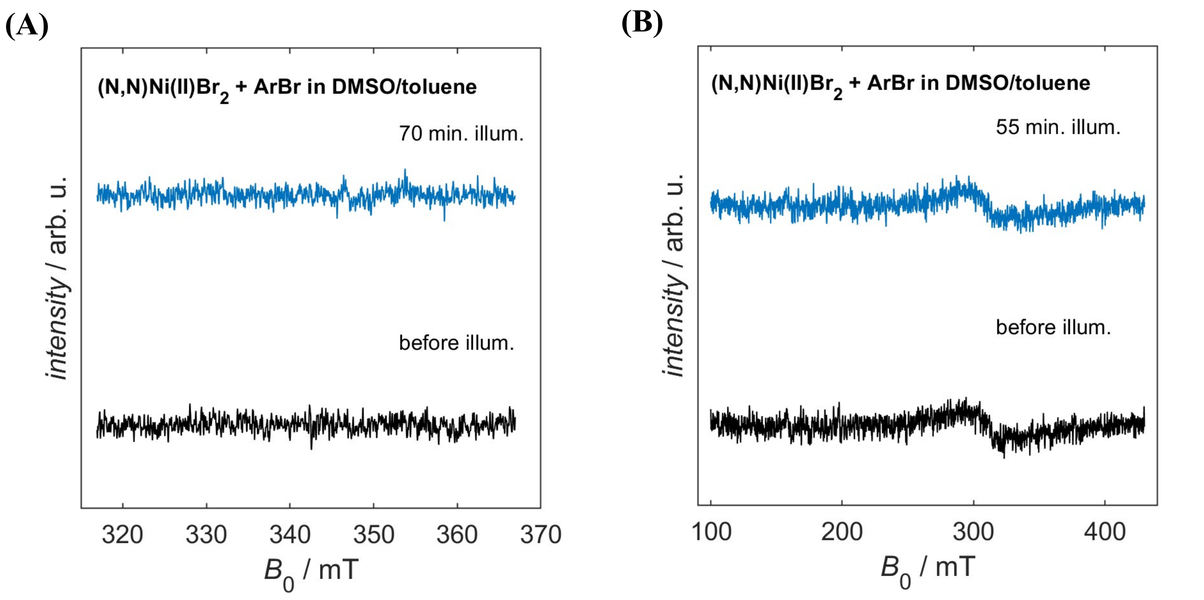

Fig. S45: Room temperature X-band EPR spectra at 9.603 GHz of Ni(4,4'-Czbpy)Br2 and ArBr in DMSO/toluene (around 2 mM) before (black) and after illumination (blue). (A) Narrow field scan around the g = 2 region. (B) Broad field scan.

Ni(4,4’-Czbpy)Br_2 + $BnBF_3K$

(B)

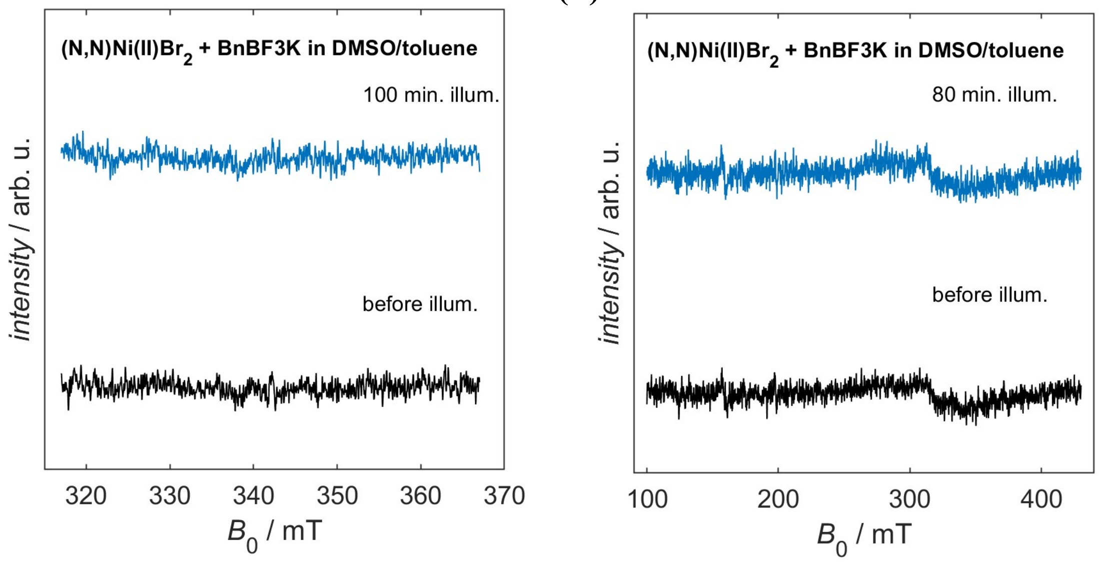

Fig. S46: Room temperature X-band EPR spectra at 9.603 GHz of Ni(4,4’-Czbpy)Br2 and BnBF3K in DMSO/toluene (around 2 mM) before (black) and after illumination (blue). (A) Narrow field scan around the g = 2 region. (B) Broad field scan.

C–S cross-coupling

Experimental procedure

An oven dried microwave vial (19 x 100 mm) equipped with a stir bar was charged with $NiCl_2$•glyme (3.3 mg, 15 µmol, 5 mol%), 4,4′-dicarbazolyl-2,2′-bipyridyl (4,4′-czbpy, 7.3 mg, 15 µmol, 5 mol%), 4-bromobenzotrifluoride (42 µL, 300 µmol), sodium *p*-toluenesulfonate (116.5 mg, 0.6 mmol, 2.0 equiv.) and *N,N*-diisopropylethylamine (DIPEA, 65.4 µL, 0.375 mmol, 1.25 equiv.). DMAc (anhydrous, 6 mL) was added and the vessel was sealed with a septum and Parafilm®. The mixture was sonicated for 5 minutes and degassed by bubbling argon for 10 minutes. The reaction mixture was stirred at 800 rpm and irradiated with two PR160L-440 lamps at full power. After 18 h, the vessel was removed from the light source, opened and 1,3,5-trimethoxybenzene (50.5 mg, 300 µmol, 1.0 equiv) was added. The mixture was stirred and an aliquote (~200 µL) was removed, diluted with DMSO-d_6 and analyzed by ^1H NMR to determine the NMR yield.
Isolation: The NMR sample was combined with the reaction mixture, diluted with H_2O (40 mL) and extracted with dichloromethane (3 x 40 mL). The combined organic phases were washed with brine (50 mL), dried over Na_2SO_4 and concentrated. The residue was purified by flash chromatography on silica gel using mixtures of hexane/ethyl acetate (gradient 0-8% ethyl acetate/hexane) to afford the title compound as a white solid (44.2 mg, 147 µmol, 49%).
^1H NMR (400 MHz, $CDCl_3$) δ 8.05 (d, J = 8.6 Hz, 2H), 7.84 (d, J = 8.3 Hz, 2H), 7.75 (d, J = 8.3 Hz, 2H), 7.33 (d, J = 8.0 Hz, 2H), 2.41 (s, 3H). ^{13}C NMR (101 MHz, $CDCl_3$) δ 145.71, 145.08, 137.70, 134.77 (q, J = 33.1 Hz), 130.31, 128.17, 128.08, 126.51 (q, J = 3.7 Hz), 123.25 (q, J = 273.1 Hz), 21.75. ^{19}F NMR (564 MHz, $CDCl_3$) δ–63.26.

These data are in full agreement with those reported in literature.[27]

The experiments in Table S1 (Entry 2-10) and Table S2 were carried out using the experimental procedure described above on a smaller scale (100 µmol ArBr).

Table S4: Studies concerning the coupling of 4-bromobenzotrifluoride with sodium *p*-toluenesulfinate.[a]

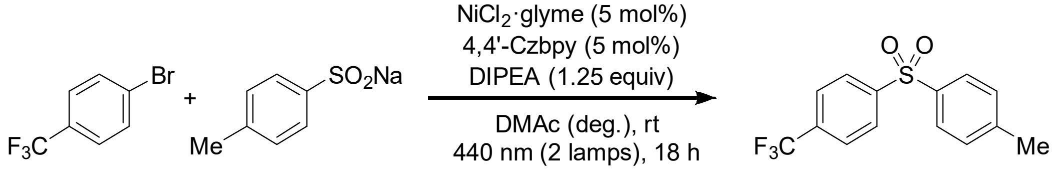

2 100 □mol 0.05 M | **1** 2 equiv | **4**

entry	variation	conversion [%][b]	4 [%][c]
1	-	99	57 (49)[d]
2	1 mol% ligand and nickel source	96	61
3	5,5′-Czbpy instead of 4,4′-Czbpy	20	4
4	w/o $NiCl_2$·glyme	5	n.d.
5	w/o 4,4′-Czbpy	10	n.d.
6	w/o 440 nm LEDs	5	n.d.
7	BIPA instead of DIPEA	24	10
8	Na_2HPO_4 instead of DIPEA	26	16
9	2,6-lutidine instead of DIPEA	27	20
10	DABCO instead of DIPEA	11	6

[a]Reaction conditions: 4-bromobenzotrifluoride (100 µmol), sodium *p*-toluensulfinate (200 µmol), $NiCl_2$•glyme (5 µmol), 4,4′-Czbpy (5 µmol), base (125 µmol), DMAc (anhydrous, 2 mL), reaction mixture was degassed before irradiation with 440 nm LED (2 lamps at full power). [b]Conversion of 4-bromobenzotrifluoride determined by ^{1}H-NMR using 1,3,5-trimethoxybenzene as internal standard. [c]NMR yields determined by ^{1}H-NMR using 1,3,5-trimethoxybenzene as internal standard. glyme = 1,2-dimethoxyethane, DIPEA = N,N-diisopropylethylamine, DABCO = 1,4-diazabicyclo[2.2.2]octane, BIPA = *N-tert*-butylisopropylamine. . n.d. = not detected

Table S5: Studies concerning the coupling of 4-bromoanisole with sodium *p*-toluenesulfinate.[a]

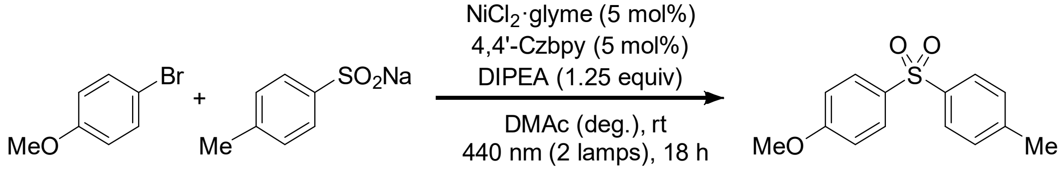

3 100 µmol 0.05 M | **1** 2 equiv | **5**

entry	variation	conversion [%][b]	4 [%][c]
1	-	14	n.d.
2	5,5′-Czbpy instead of 4,4′-Czbpy	39	n.d.

[a]Reaction conditions: 4-bromoanisole (100 µmol), sodium *p*-toluensulfinate (200 µmol), $NiCl_2$•glyme (5 µmol), 4,4′-Czbpy (5 µmol), base (125 µmol), DMAc (anhydrous, 2 mL), reaction mixture was degassed before irradiation with 440 nm LED (2 lamps at full power). [b]Conversion of 4-bromoanisole determined by ^{1}H-NMR using 1,3,5-trimethoxybenzene as internal standard. [c]NMR yields determined by ^{1}H-NMR using 1,3,5-trimethoxybenzene as internal standard. glyme = 1,2-dimethoxyethane, DIPEA = N,N-diisopropylethylamine, n.d. = not detected

CV studies: Oxidative addition

<u>Ni(5,5′-Czbpy)Br₂</u>

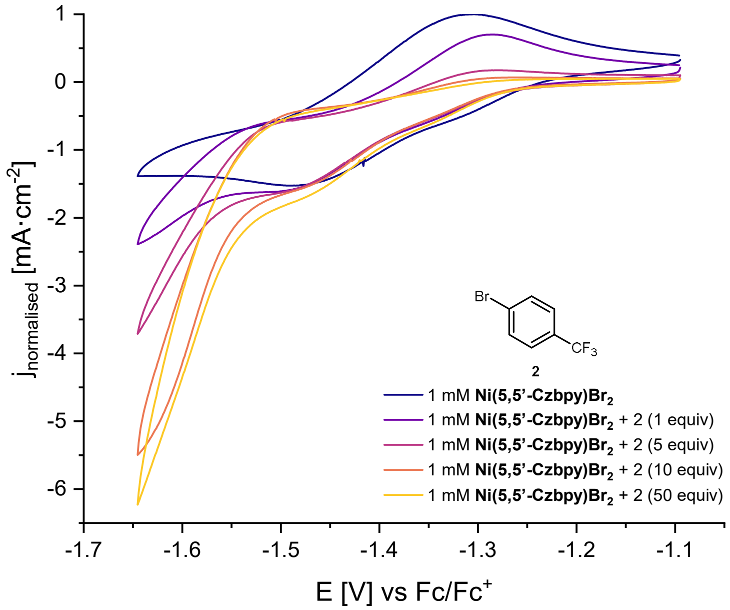

Fig. S47: CV of 1 mM Ni(5,5′-Czbpy)Br_2 with addition of 1-bromo-4-(trifluoromethyl)benzene (**2**) in DMAc with 0.1 M Bu_4NBr as supporting electrolyte at 100 mV·s^{-1}.

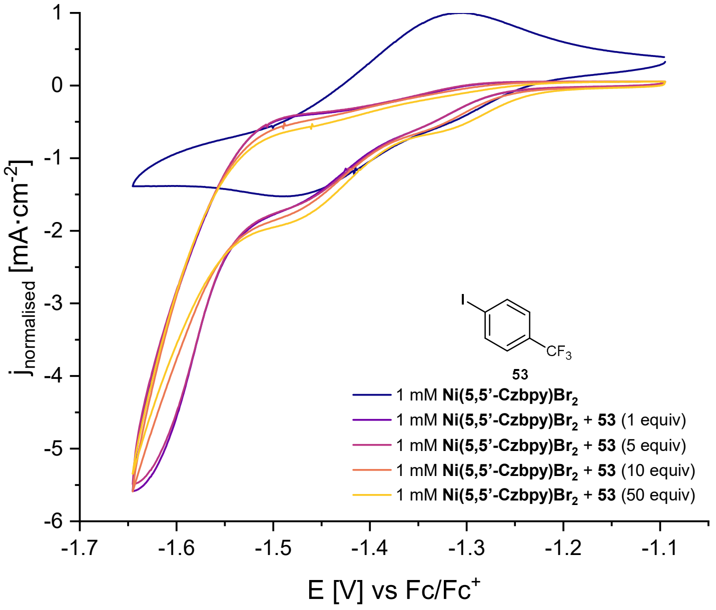

Fig. S48: CV of 1 mM of Ni(5,5′-Czbpy)Br_2 with addition of 1-iodo-4-(trifluoromethyl)benzene (**53**) in DMAc with 0.1 M Bu_4NBr as supporting electrolyte at 100 mV·s^{-1}.

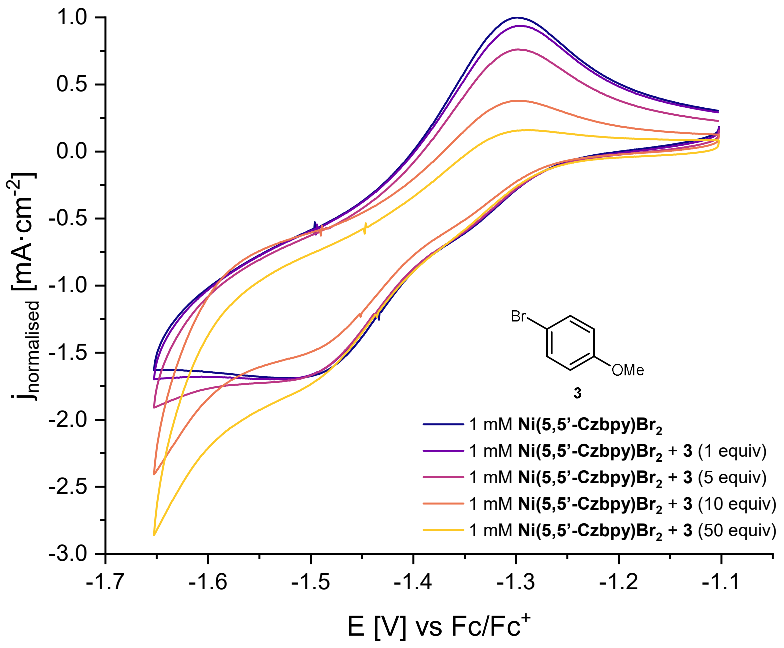

Fig. S49: CV of 1 mM of Ni(5,5′-Czbpy)Br_2 with addition of 1-bromo-4-methoxybenzene (**3**) in DMAc with 0.1 M Bu_4NBr as supporting electrolyte at 100 mV·s^{-1}.

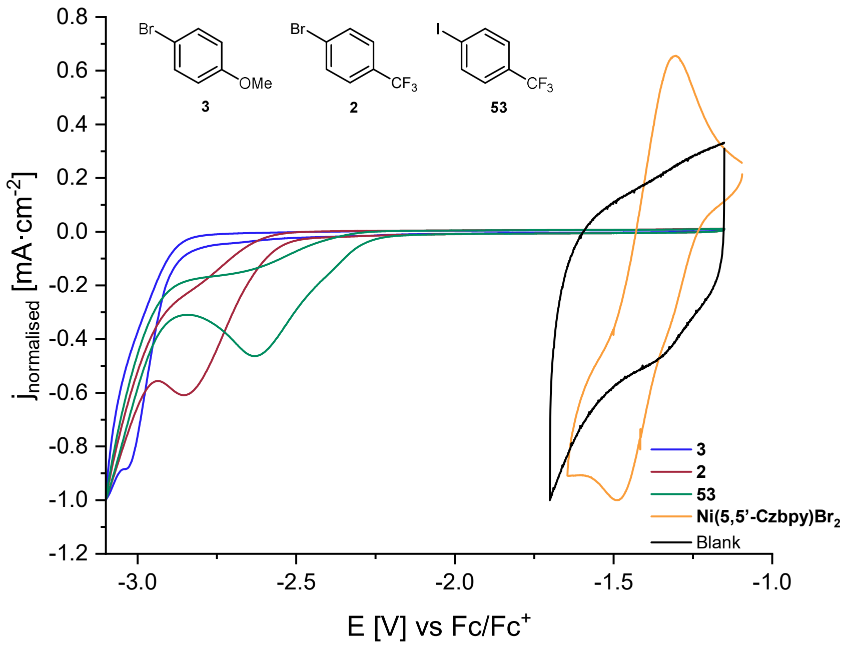

Fig. S50: CVs of blank (0.1 M Bu_4NBr), 1 mM Ni(5,5′-Czbpy)Br_2, 1 mM 1-iodo-4-(trifluoromethyl)benzene (**53**), 1 mM 1-bromo-4-(trifluoromethyl)benzene (**2**), 1 mM 1-bromo-4-methoxybenzene (**3**) in DMAc with 0.1 M Bu_4NBr as supporting electrolyte at 100 mV·s^{-1}.

Ni(4,4′-Czbpy)Br$_2$

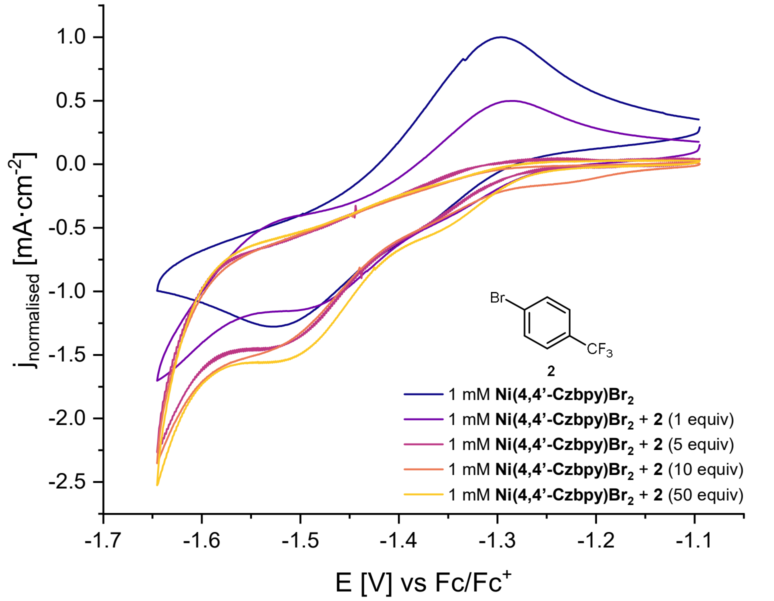

Fig. S51: CV of 1 mM of Ni(4,4′-Czbpy)Br$_2$ with addition of 1-bromo-4-(trifluoromethyl)benzene (**2**) in DMAc with 0.1 M Bu_4NBr as supporting electrolyte at 100 mV·s^{-1}.

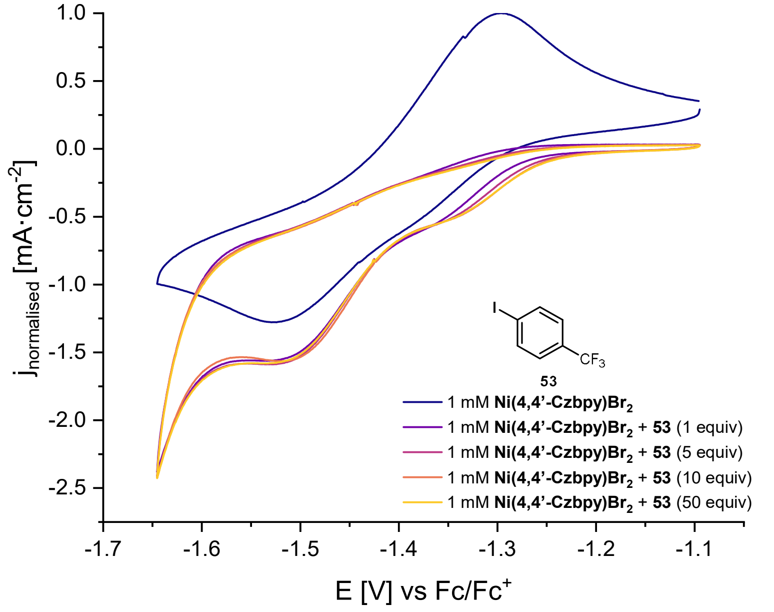

Fig. S52: CV of 1 mM of Ni(4,4′-Czbpy)Br$_2$ with addition of 1-iodo-4-(trifluoromethyl)benzene (**53**) in DMAc with 0.1 M Bu_4NBr as supporting electrolyte at 100 mV·s^{-1}.

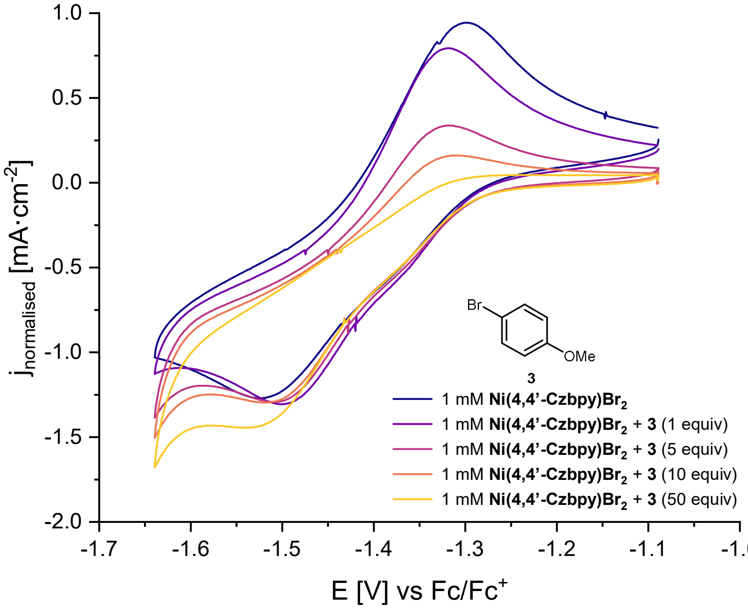

Fig. S53: CV of 1 mM of Ni(4,4′-Czbpy)Br_2 and 1-bromo-4-methoxybenzene (**3**) as additive in DMAc with 0.1 M Bu_4NBr as supporting electrolyte at 100 mV·s^{-1}.

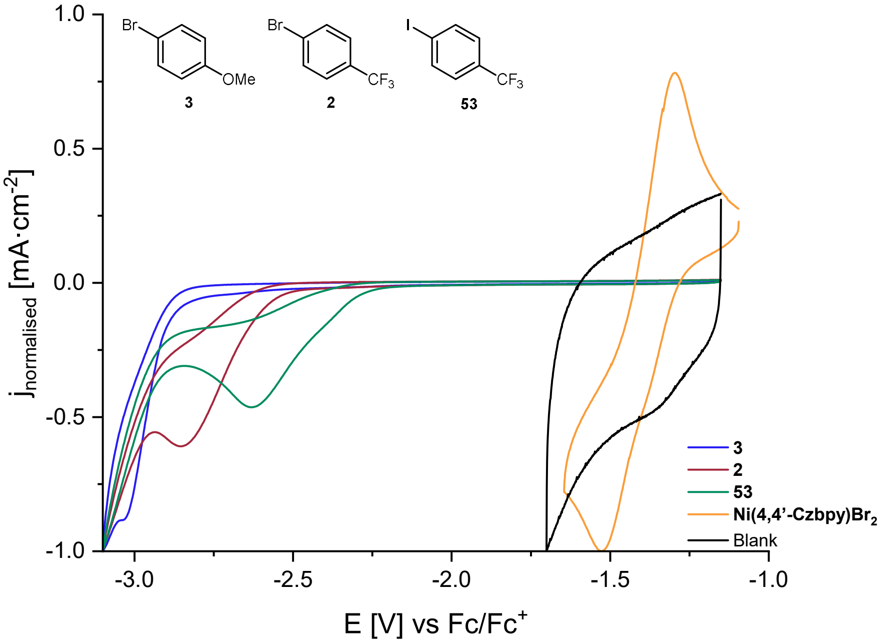

Fig. S54: CVs of blank (0.1 M Bu_4NBr), 1 mM Ni(4,4′-Czbpy)Br_2, 1 mM 1-iodo-4-(trifluoromethyl)benzene (**53**), 1 mM 1-bromo-4-(trifluoromethyl)benzene (**2**) and 1 mM 1-bromo-4-methoxybenzene (3) in DMAc with 0.1 M Bu_4NBr as supporting electrolyte at 100 mV·s^{-1}.

C–C cross-coupling

Initial experiments and reaction optimization

General procedure

An oven dried vial (19 x 100 mm) equipped with a stir bar was charged with the respective amount of nickel salt, ligand and substrates. Solvent (anhydrous) and base were added and the vessel was sealed with a septum and Parafilm®. The mixture was stirred for 1 minute at high speed, followed by sonication for 10 minutes and degassing by bubbling Ar, or freeze/pump/thaw (3 cycles). The reaction mixture was stirred at 800 rpm and irradiated with 440 nm LED lamps using the respective power settings. After the respective reaction time, 1,3,5-trimethoxybenzene (1 equiv) was added to the reaction vessel, the mixture was shaken and an aliquote (300 μL) was removed, diluted with DMSO-d_6 and analyzed by ^{1}H NMR.

Table S6: Initial results.[a]

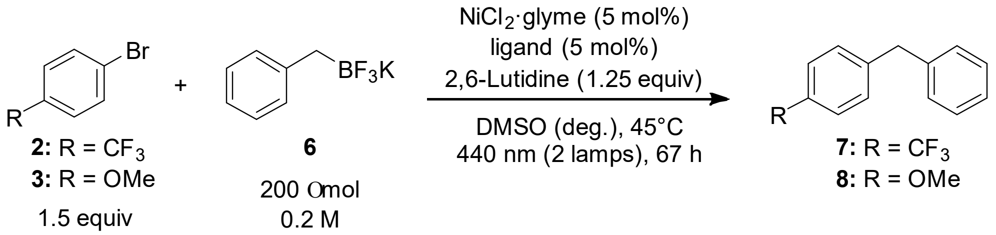

entry	Aryl bromide	ligand	conversion [%][b]	7/8 [%][c]
1	2	4,4′-Czbpy	91	44
2	2	5,5′-Czbpy	18	n.d.
3	3	4,4′-Czbpy	56	20
4	3	5,5′-Czbpy	17	n.d.

[a]Reaction conditions: aryl bromide (300 μmol), potassium benzyltrifluoroborate (200 μmol), $NiCl_2$·glyme (10 μmol), ligand (10 μmol), 2,6-lutidine (250 μmol), DMSO (anhydrous, 1 mL), reaction mixture was degassed before irradiation with 440 nm LED (2 lamps at full power). [b]Conversion of potassium benzyltrifluoroborate determined by ^{1}H-NMR using 1,3,5-trimethoxybenzene as internal standard. [c]NMR yields determined by ^{1}H-NMR using 1,3,5-trimethoxybenzene as internal standard. glyme = 1,2-dimethoxyethane. n.d. = not detected.

Table S7: Solvent screening for the coupling of 4-bromobenzotrifluoride and $BnBF_3K$.[a]

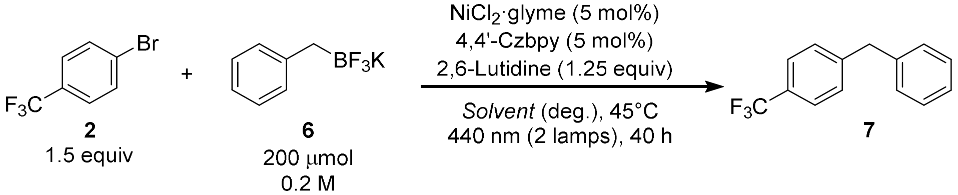

entry	solvent	conversion [%][b]	7 [%][c]
1	DMSO	60	27
2	DMF	60	30
3	DMAc	34	16
4	THF	99	60
5	MeCN	82	7
6	$CHCl_3$	99	8
7	Acetone	54	19

[a]Reaction conditions: 4-bromobenzotrifluoride (300 µmol), potassium benzyltrifluoroborate (200 µmol), $NiCl_2$·glyme (10 µmol), 4,4′-Czbpy (10 µmol), 2,6-lutidine (250 µmol), solvent (anhydrous, 1 mL), reaction mixture was degassed before irradiation with 440 nm LED (2 lamps at full power). [b]Conversion of potassium benzyltrifluoroborate determined by ^{1}H-NMR using 1,3,5-trimethoxybenzene as internal standard. [c]NMR yields determined by ^{1}H-NMR using 1,3,5-trimethoxybenzene as internal standard. glyme = 1,2-dimethoxyethane.

Table S8: Stoichiometry screening for the coupling of 4-bromobenzotrifluoride and $BnBF_3K$.[a]

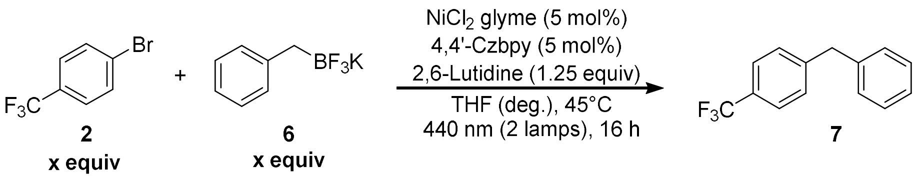

entry	Ar-Br (equiv)	$BnBF_3K$ (equiv)	conversion [%][b]	7 [%][c]
1	1.5	1	96	32
2	2	1	quant.	32
3	3	1	quant.	30
4	1	1.5	50	39
5	1	3	51	38

[a]Reaction conditions: 4-bromobenzotrifluoride (200-600 µmol), potassium benzyltrifluoroborate (200-600 µmol), $NiCl_2$·glyme (10 µmol), 4,4′-Czbpy (10 µmol), 2,6-lutidine (250 µmol), THF (anhydrous, 1 mL), reaction mixture was degassed before irradiation with 440 nm LED (2 lamps at full power). [b]Conversion of the limiting reagent determined by ^{1}H-NMR using 1,3,5-trimethoxybenzene as internal standard. [c]NMR yields determined by ^{1}H-NMR using 1,3,5-trimethoxybenzene as internal standard. glyme = 1,2-dimethoxyethane.

Table S 9. Base screening for the coupling of 4-bromobenzotrifluoride and potassium $BnBF_3K$.[a]

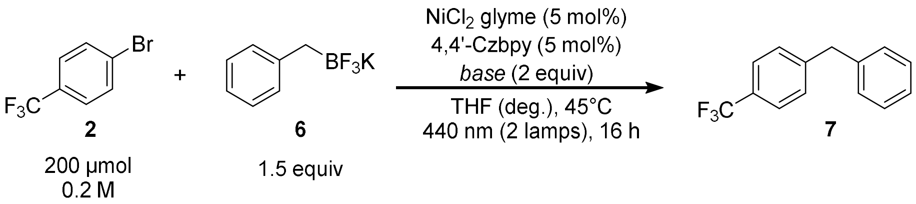

entry	base	conversion [%][b]	7 [%][c]
1	-	52	39
2	2,6-Lutidine	53	42
3	TMP	11	n.d.
4	BIPA	12	n.d.
5	DIPEA	23	3
6	DBU	12	n.d.
7	MTBD	13	n.d.
8	DABCO	10	n.d.
9	Na_2CO_3	33	n.d.
10	Cs_2CO_3	11	n.d.
12	K_2CO_3	13	n.d.

[a]Reaction conditions: 4-bromobenzotrifluoride (200 µmol), potassium benzyltrifluoroborate (300 µmol), $NiCl_2$·glyme (10 µmol), 4,4′-Czbpy (10 µmol), base (250 µmol), THF (anhydrous, 1 mL), reaction mixture was degassed before irradiation with 440 nm LED (2 lamps at full power). [b]Conversion of the limiting reagent determined by ^{1}H-NMR using 1,3,5-trimethoxybenzene as internal standard. [c]NMR yields determined by ^{1}H-NMR using 1,3,5-trimethoxybenzene as internal standard. glyme = 1,2-dimethoxyethane. n.d. = not detected. TMP = 2,2,6,6-tetramethylpiperidine. BIPA = *N*-*tert*-butylisopropylamine. DIPEA = N,N-diisopropylethylamine. DBU = 1,8-diazabicyclo[5.4.0]undec-7-ene. MTBD = 7-Methyl-1,5,7-triazabicyclo[4.4.0]dec-5-en. DABCO = 1,4-diazabicyclo[2.2.2]octane.

Table S10. Ni(II) salt screening for the coupling of 4-bromobenzotrifluoride and $BnBF_3K$.[a]

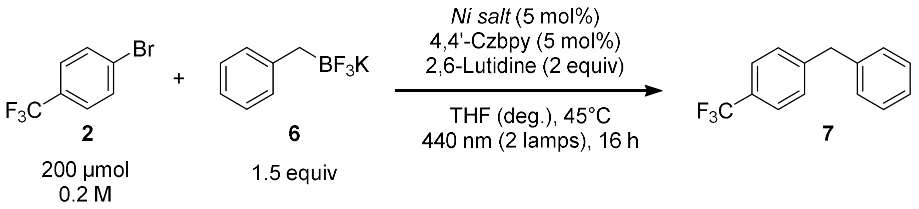

entry	Ni salt	base	conversion [%][b]	7 [%][c]
1	$NiCl_2$·glyme	2,6-Lutidine	53	42
2	$NiCl_2$·glyme	-	52	39
3	$NiBr_2$·glyme	2,6-Lutidine	57	46
4	$NiBr_2$·glyme	-	47	36
5	$NiCl_2 \cdot 6H_2O$	2,6-Lutidine	49	34
6	$NiCl_2 \cdot 6H_2O$	-	41	23
7	$Ni(ClO_4)_2 \cdot 6H_2O$	2,6-Lutidine	61	54
8	$Ni(ClO_4)_2 \cdot 6H_2O$	-	49	36
9	$NiBr_2$	2,6-Lutidine	79	67
10	$NiBr_2$	-	53	39
11	$Ni(OTf)_2$	2,6-Lutidine	49	25
12	$Ni(OTf)_2$	-	43	25
13	$Ni(NO_3)_2 \cdot 6H_2O$	2,6-Lutidine	46	19
14	$Ni(NO_3)_2 \cdot 6H_2O$	-	22	6

[a]Reaction conditions: 4-bromobenzotrifluoride (200 µmol), potassium benzyltrifluoroborate (300 µmol), Ni(II) salt (10 µmol), 4,4′-Czbpy (10 µmol), 2,6-lutidine (250 µmol), THF (anhydrous, 1 mL), reaction mixture was degassed before irradiation with 440 nm LED (2 lamps at full power). [b]Conversion of 4-bromobenzotrifluoride determined by ^{1}H-NMR using 1,3,5-trimethoxybenzene as internal standard. [c]NMR yields determined by ^{1}H-NMR using 1,3,5-trimethoxybenzene as internal standard. glyme = 1,2-dimethoxyethane.

Table S11. Final optimization for the coupling of 4-bromobenzotrifluoride and $BnBF_3K$.[a]

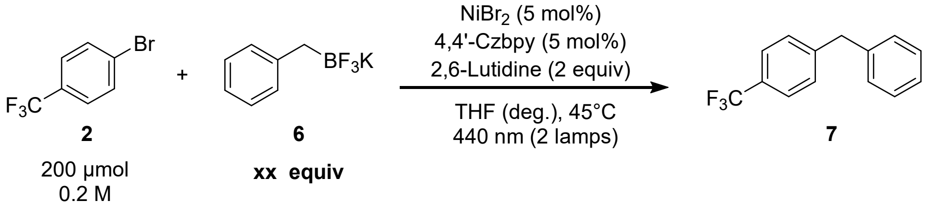

Entry	$BnBF_3K$ [equiv]	Time (h)	Conversion [%][b]	7 [%][c]
1	1.5	16	79	67
2	1.5	24	75	67
3	2	24	85	71
4	2	48	87	77
5	2.5	48	quant.	99

[a]Reaction conditions: 4-bromobenzotrifluoride (200 µmol), potassium benzyltrifluoroborate (200-500 µmol), $NiBr_2$ (10 µmol), 4,4′-Czbpy (10 µmol), 2,6-lutidine (400 µmol), THF (anhydrous, 1 mL), reaction mixture was degassed before irradiation with 440 nm LED (2 lamps at full power). [b]Conversion of 4-bromobenzotrifluoride determined by ^{1}H-NMR using 1,3,5-trimethoxybenzene as internal standard. [c]NMR yields determined by ^{1}H-NMR using 1,3,5-trimethoxybenzene as internal standard.

Table S12. Control studies for the coupling of 4-bromobenzotrifluoride and $BnBF_3K$.[a]

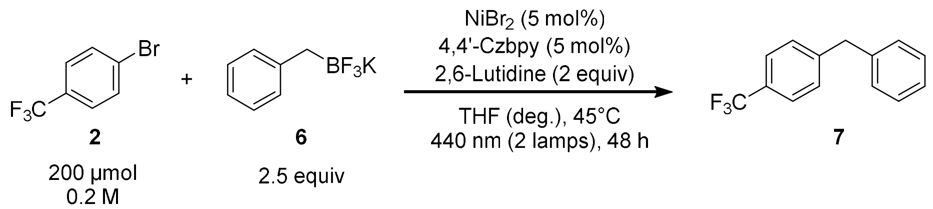

Entry	Variations	Conversion [%][b]	7 [%][c]
1	-	quant	99(92)[d]
2	4,4′-Czbpy & $NiBr_2$ (2.5 mol%)	96	84
3	4,4′-Czbpy & $NiBr_2$ (1 mol%)	14	n.d.
4	5,5′-Czbpy instead of 4,4′-Czbpy	27	20
5	4,4′-Dimethoxy-2,2′-bipyridine instead of 4,4′-Czbpy	0	n.d.
6	4,4′-Dimethoxy-2,2′-bipyridine instead of 4,4′-Czbpy; w/o 440 nm LEDs	0	n.d.
7	4,4′-Dimethylamino-2,2′-bipyridine instead of 4,4′-Czbpy	0	n.d.
8	4,4′-Dimethylamino-2,2′-bipyridine instead of 4,4′-Czbpy; w/o 440 nm LEDs	0	n.d.
9	+ 1 equiv Mn^0; w/o 440 nm LEDs	<5	n.d.
10	+ 1 equiv Zn^0; w/o 440 nm LEDs	<10	n.d.
11	+ 1 equiv Zn^0	75	37
12	w/o $NiCl_2$·glyme	4	n.d.
13	w/o 4,4′-Czbpy	3	n.d.
14	w/o 440 nm LEDs	1	n.d.
15	4-Chlorobenzotrifluoride	60	21

[a]Reaction conditions: 4-bromobenzotrifluoride (200 µmol), potassium benzyltrifluoroborate (500 µmol), $NiBr_2$ (10 µmol), 4,4′-Czbpy (10 µmol), 2,6-lutidine (400 µmol), THF (anhydrous, 1 mL), reaction mixture was degassed before irradiation with 440 nm LED (2 lamps at full power). [b]Conversion of 4-bromobenzotrifluoride determined by ^{1}H-NMR using 1,3,5-trimethoxybenzene as internal standard. [c]NMR yields determined by ^{1}H-NMR using 1,3,5-trimethoxybenzene as internal standard. [d]Isolated yield in brackets. n.d. = not detected.

Scope

General procedure

An oven dried microwave vial (19 x 100 mm) equipped with a stir bar was charged with $NiBr_2$ (2.2 mg, 10 μmol, 5 mol%), 4,4′-dicarbazolyl-2,2′-bipyridyl (4,4′-Czbpy, 4.9 mg, 10 μmol, 5 mol%), aryl bromide (200 μmol), potassium trifluoroborate salt (0.5 mmol, 2.5 equiv.) and 2,6-lutidine (47 μL, 0.4 mmol, 2 equiv.). THF (anhydrous, inhibitor-free, 1 mL) was added and the vessel was sealed. The mixture was sonicated for 20 minutes and degassed by freeze-pump-thaw (3 cycles). The reaction mixture was stirred at 800 rpm and irradiated with two LED lamps (440 nm) at full power. After 48 h, 1,3,5-trimethoxybenzene (33.6 mg, 200 μmol, 1.0 equiv.) was added to the reaction vessel, the mixture was stirred and an aliquote (~200 μL) was removed, diluted with CD_3Cl and analyzed by ^1H NMR to determine NMR yields. The NMR sample was combined with the reaction mixture, and purified by flash chromatography on silica gel using mixtures of hexane/ethyl acetate to afford the title compounds.

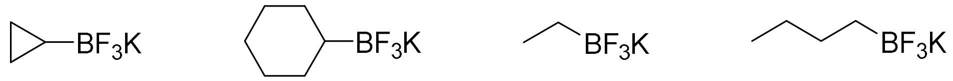

Fig. S55. Trifluoroborate salts that did not result in product formation under the optimized reaction conditions. In all cases, no consumption of starting materials was observed

1-benzyl-4-(trifluoromethyl)benzene (**7**)

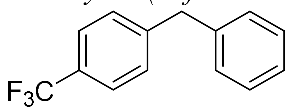

According to the optimized procedure from 4-bromobenzotrifluoride (112.5 mg, 0.5 mmol) and potassium benzyltrifluoroborate (247.5 mg, 1.25 mmol, 2.5 equiv.). NMR yield: >99%.
The title compound was isolated by column chromatography (100% hexane) as a colorless oil (108 mg, 0.46 mmol, 92%).
^1H NMR (400 MHz, $CDCl_3$) δ 7.55 (d, J = 8.2 Hz, 2H), 7.37 – 7.28 (m, 4H), 7.29 – 7.22 (m, 1H), 7.22 – 7.16 (m, 2H), 4.13 (s, 2H). ^{13}C NMR (151 MHz, $CDCl_3$) δ 145.34, 140.12, 129.33, 129.08, 128.80, 128.57 (q, J = 32.3 Hz), 126.61, 125.53 (q, J = 3.8 Hz), 124.45 (q, J = 271.46 Hz), 41.84.
^{19}F NMR (376 MHz, $CDCl_3$) δ -62.3.

These data are in full agreement with those reported in literature.[28]

4-benzylbenzonitrile (**9**)

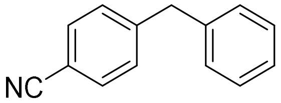

According to optimized procedure using 4-bromobenzonitrile (36 mg, 0.2 mmol) and potassium benzyltrifluoroborate (99 mg, 0.5 mmol, 2.5 equiv.). NMR yield: 96%.

The title compound was isolated by column chromatography (5% EA in hexanes) as a yellow oil (35 mg, 0.181 mmol, 89%).

^{1}H NMR (400 MHz, $CDCl_3$) δ 7.59 (d, J = 8.0 Hz, 2H), 7.37 – 7.23 (m, 5H), 7.18 (d, J = 8 Hz, 2H), 4.05 (s, 2H). ^{13}C NMR (101 MHz, $CDCl_3$) δ 146.85, 139.44, 132.41, 129.75, 129.07, 128.88, 126.79, 119.11, 110.15, 42.08.

These data are in full agreement with those reported in literature.[28]

3-benzylbenzonitrile (**10**)

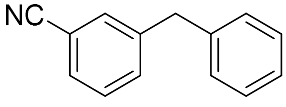

According to optimized procedure using 3-bromobenzonitrile (36 mg, 0.2 mmol) and potassium benzyltrifluoroborate (99 mg, 0.5 mmol, 2.5 equiv.). NMR yield: >99%.

The title compound was isolated by column chromatography (5% EA in hexanes) as a yellow oil (36 mg, 0.186 mmol, 93%).

^{1}H NMR (400 MHz, $CDCl_3$) δ 7.52 – 7.46 (m, 2H), 7.45 – 7.41 (m, 1H), 7.41 – 7.36 (m, 1H), 7.35 – 7.30 (m, 2H), 7.28 – 7.23 (m, 1H), 7.17 (d, J = 7.6 Hz, 2H), 4.01 (s, 2H). ^{13}C NMR (101 MHz, $CDCl_3$) δ 142.71, 139.51, 133.53, 132.45, 130.04, 129.34, 129.02, 128.89, 126.78, 119.05, 112.57, 41.49.

These data are in full agreement with those reported in literature.[28]

2-benzylbenzonitrile (**11**)

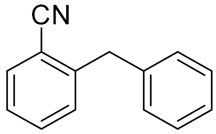

According to optimized procedure using methyl 2-bromobenzonitrile (41 mg, 0.5 mmol) and potassium benzyltrifluoroborate (247.5 mg, 1.25 mmol, 2.5 equiv.). NMR yield: >99%.

The title compound was isolated by column chromatography (100% hexane) as a colorless oil using hexane (81.1 mg, 0.42 mmol, 84%).

^{1}H NMR (600 MHz, $CDCl_3$) δ 7.65 (dd, *J* = 7.7, 1.4 Hz, 1H), 7.51 (td, *J* = 7.7, 1.4 Hz, 1H), 7.37 – 7.22 (m, 7H), 4.22 (s, 2H). ^{13}C NMR (151 MHz, $CDCl_3$) δ 145.05, 138.88, 133.02 (m), 130.15, 129.09, 128.83, 126.91, 126.82, 118.32, 112.63, 40.28.

These data are in full agreement with those reported in literature.[29]

1-benzyl-4-(methylsulfonyl)benzene (**12**)

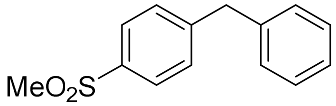

According to optimized procedure using 1-bromo-4-(methylsulfonyl)benzene (47 mg, 0.2 mmol) and potassium benzyltrifluoroborate (99 mg, 0.5 mmol, 2.5 equiv.). NMR yield: >99%.

The title compound was isolated by column chromatography (10% EA in hexanes) as a yellow solid (41 mg, 0.166 mmol, 83%).

^{1}H NMR (400 MHz, $CDCl_3$) δ 7.87 (d, *J* = 8.4 Hz, 2H), 7.40 (d, *J* = 8.4 Hz, 2H), 7.37 – 7.31 (m, 2H), 7.29 – 7.24 (m, 1H), 7.20 (d, *J* = 6.8 Hz, 2H), 4.09 (s, 2H), 3.05 (s, 3H). ^{13}C NMR (101 MHz, $CDCl_3$) δ 147.80, 139.51, 138.43, 129.88, 129.06, 128.86, 127.70, 126.76, 44.64, 41.89.

These data are in full agreement with those reported in literature.[28]

methyl 3-benzylbenzoate (**13**)

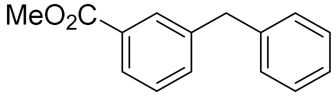

According to optimized procedure using methyl 4-bromobenzoate (43 mg, 0.2 mmol) and potassium benzyltrifluoroborate (99 mg, 0.5 mmol, 2.5 equiv.). NMR yield: 63%.

The title compound was isolated by column chromatography (2% EA in hexanes) as a yellow oil (27 mg, 0.12 mmol, 60%).

^{1}H NMR (400 MHz, $CDCl_3$) δ 7.93 – 7.87 (m, 2H), 7.41 – 7.34 (m, 2H), 7.33 – 7.27 (m, 2H), 7.24 – 7.17 (m, 3H), 4.03 (s, 2H), 3.90 (s, 3H). ^{13}C NMR (101 MHz, $CDCl_3$) δ 167.28, 141.58, 140.60, 133.68, 130.43, 130.15, 128.99, 128.71, 128.68, 127.56, 126.42, 52.23, 41.83.

These data are in full agreement with those reported in literature.[28]

1-benzyl-2,3-dichlorobenzene (**14**)

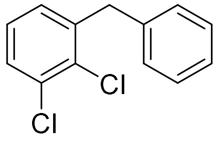

According to optimized procedure using 1-bromo-2,3-dichorobenzene (112.9 mg, 0.5 mmol) and potassium benzyltrifluoroborate (247 mg, 1.25 mmol, 2.5 equiv.). NMR yield: 87%.

The title compound was isolated by column chromatography (2% EA in hexanes) as a colorless oil (94.8 mg, 0.4 mmol, 80%).

^{1}H NMR (400 MHz, $CDCl_3$) δ 7.43 – 7.32 (m, 3H), 7.31 – 7.26 (m, 1H), 7.26 – 7.21 (m, 2H), 7.16 (t, J = 7.8 Hz, 1H), 7.10 (dd, J = 7.7, 1.7 Hz, 1H), 4.19 (s, 2H). ^{13}C NMR (101 MHz, $CDCl_3$) δ 141.20, 138.98, 133.37, 132.65, 129.21, 129.09, 128.70, 128.63, 127.25, 126.61, 40.23.

These data are in full agreement with those reported in literature.[30]

4-benzylbenzaldehyde (**15**)

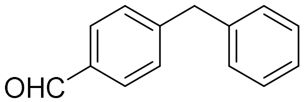

According to optimized procedure using 4-bromobenzaldehyde (37 mg, 0.2 mmol) and potassium benzyltrifluoroborate (99 mg, 0.5 mmol). NMR yield: 74%.

The title compound was isolated by column chromatography (2% EA in hexanes) as a yellow solid (28 mg, 0.142 mmol, 71%).

^{1}H NMR (400 MHz, $CDCl_3$) δ 9.99 (s, 1H), 7.83 (m, 2H), 7.40 – 7.31 (m, 4H), 7.28 – 7.23 (m, 1H), 7.21 (m, 2H), 4.08 (s, 2H). ^{13}C NMR (101 MHz, $CDCl_3$) δ 192.09, 148.56, 139.89, 134.81, 130.17, 129.69, 129.11, 128.82, 126.65, 42.22.

These data are in full agreement with those reported in literature.[2]

4-benzylbenzamide (**16**)

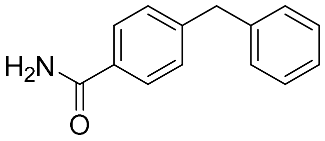

According to optimized procedure using 4-bromobenzamide (99.5 mg, 0.5 mmol) and potassium benzyltrifluoroborate (247 mg, 1.25 mmol, 2.5 equiv). NMR yield: 90%.

The title compound was isolated by column chromatography (0-50% EA in hexanes) as a yellow oil (92 mg, 0.43 mmol, 87%).

^{1}H NMR (400 MHz, $CDCl_3$) δ 7.80 – 7.72 (m, 2H), 7.37 – 7.16 (m, 7H), 4.06 (s, 2H). ^{13}C NMR (101 MHz, $CDCl_3$) δ 169.28, 145.73, 140.29, 131.26, 129.32, 129.07, 128.76, 127.74, 126.53, 41.91.

These data are in full agreement with those reported in literature.[31]

1-benzyl-3-(trifluoromethoxy)benzene (**17**)

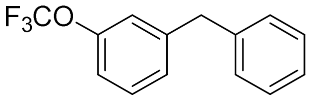

According to optimized procedure using 1-bromo-3-(trifluoromethoxy)benzene (120 mg, 0.5 mmol) and potassium benzyltrifluoroborate (247.5 mg, 1.25 mmol, 2.5 equiv.). NMR yield: >99%. The title compound was isolated by flash chromatography (100% hexane) as a colorless oil (92.0 mg, 0.36 mmol, 73%).

^{1}H NMR (400 MHz, $CDCl_3$) δ 7.44 – 7.29 (m, 4H), 7.29 – 7.24 (m, 2H), 7.19 (dd, *J* = 7.8, 1.4 Hz, 1H), 7.17 – 7.12 (m, 2H), 4.07 (s, 2H).^{13}C NMR (101 MHz, $CDCl_3$) δ 149.50 (q, *J* = 1.9 Hz), 143.55, 140.16, 129.83, 129.05, 128.77, 127.43, 126.56, 121.58 (m), 120.60 (q, *J* = 256.7 Hz), 118.63 (m), 41.70. ^{19}F NMR (564 MHz, $CDCl_3$) δ -57.70.

These data are in full agreement with those reported in literature.[28]

(4-benzylphenyl)(phenyl)methanone (**18**)

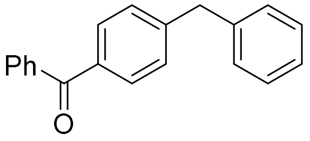

According to optimized procedure using (4-bromophenyl)(phenyl)methanone (52 mg, 0.2 mmol) and potassium benzyltrifluoroborate (99 mg, 0.5 mmol, 2.5 equiv.). NMR yield: >99%.

The title compound was isolated by column chromatography (2% EA in hexanes) as a yellow oil (52 mg, 0.19 mmol, 95%).

^{1}H NMR (400 MHz, $CDCl_3$) δ 7.71 (d, *J* = 8.4 Hz, 2H), 7.67 (d, *J* = 8.3 Hz, 2H), 7.52 – 7.45 (m, 1H), 7.39 (td, *J* = 7.4, 1.3 Hz, 2H), 7.24 (ddt, *J* = 8.2, 6.1, 1.7 Hz, 4H), 7.16 (ddd, *J* = 12.0, 5.5, 3.4 Hz, 3H), 3.98 (s, 2H). ^{13}C NMR (101 MHz, $CDCl_3$) δ 196.53, 146.27, 140.19, 137.85, 135.57, 132.37, 130.58, 130.08, 129.09, 128.92, 128.74, 128.33, 126.52, 42.03.

These data are in full agreement with those reported in literature.[32]

diphenylmethane (**19**)

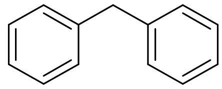

According to optimized procedure using bromobenzene (31 mg, 0.2 mmol) and potassium benzyltrifluoroborate (99 mg, 0.5 mmol, 2.5 equiv.). NMR yield: 90%.

The title compound was isolated by column chromatography (10% EA in hexanes) as a colorless oil (28 mg, 0.166 mmol, 83%) that contains 13% of 1,2-diphenylethane as inseparable impurity.

^{1}H NMR (400 MHz, $CDCl_3$) δ 7.38 – 7.31 (m, 4H: contains 1,2-diphenylethane), 7.29 – 7.22 (m, 6H: contains 1,2-diphenylethane), 4.05 (s, 2H), (2.97 (s, 4H), 1,2-diphenylethane[33]). ^{13}C NMR (101 MHz, $CDCl_3$) δ 141.24, 129.06, 128.59, 126.19, 42.06, (141.91, 128.47, 126.05, 38.09, 1,2-diphenylethane[33]).

These data are in full agreement with those reported in literature.[28]

1-benzyl-4-fluorobenzene (**20**)

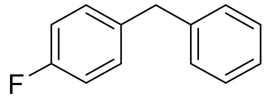

According to optimized procedure using 1-bromo-4-fluorobenzene (35 mg, 0.2 mmol) and potassium benzyltrifluoroborate (99 mg, 0.5 mmol, 2.5 equiv.). NMR yield: 97%.

The title compound was isolated by column chromatography (1% EA in hexanes) as a colorless oil (29 mg, 0.156 mmol, 78%) that contains 10% of 1,2-diphenylethane as inseparable impurity.

^{1}H NMR (400 MHz, $CDCl_3$) δ 7.38 – 7.32 (m, 2H), 7.29 – 7.17 (m, 5H: contains 1,2-diphenylethane), 7.05 – 6.99 (m, 2H), 4.01 (s, 2H), (2.99 (s, 4H), 1,2-diphenylethane[33]). ^{13}C NMR (101 MHz, $CDCl_3$) δ 161.49 (d, J = 243.9 Hz), 141.07, 136.89 (d, J = 3.2 Hz), 130.41 (d, J = 7.8 Hz), 128.96, 128.66, 126.33, 115.33 (d, J = 21.2 Hz), 41.19, (141.91, 128.58, 128.47, 126.05, 38.09, 1,2-diphenylethane[33]). ^{19}F NMR (564 MHz, $CDCl_3$) δ -117.40.

These data are in full agreement with those reported in literature.[28]

1-benzyl-2-methoxybenzene (**21**)

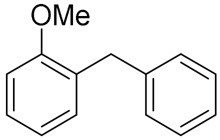

According to optimized procedure using methyl 2-bromoanisole (37 mg, 0.2 mmol) and potassium benzyltrifluoroborate (99 mg, 0.5 mmol, 2.5 equiv.). NMR yield: 65%.

The title compound was isolated by column chromatography (2% EA in hexanes) as a yellow oil (24 mg, 0.121 mmol, 61%).

^{1}H NMR (400 MHz, $CDCl_3$) δ 7.34 – 7.28 (m, 2H), 7.27 – 7.19 (m, 4H), 7.11 (d, J = 8 Hz, 1H), 6.94 – 6.89 (m, 2H), 4.02 (s, 2H), 3.85 (s, 3H). ^{13}C NMR (101 MHz, $CDCl_3$) δ 157.47, 141.16, 130.45, 129.79, 129.09, 128.37, 127.53, 125.89, 120.60, 110.53, 55.46, 35.98.

These data are in full agreement with those reported in literature[34]

1-benzyl-3-methylbenzene (**22**)

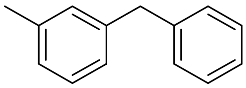

According to optimized procedure using 3-bromotoluene (37 mg, 0.2 mmol) and potassium benzyltrifluoroborate (99 mg, 0.5 mmol, 2.5 equiv.). NMR yield: >99%.

The title compound was isolated by column chromatography (100% hexane) as a colorless oil (33 mg, 0.18 mmol, 91%) that contains 15% of 1,2-diphenylethane as inseparable impurity.

^{1}H NMR (400 MHz, $CDCl_3$) δ 7.43 – 7.35 (m, 2H), 7.29 (dt, J = 7.5, 2.3 Hz, 4H), 7.15 – 7.05 (m, 3H), 4.05 (s, 2H), 2.41 (s, 3H), (3.03 (s, 4H), 1,2-diphenylethane[33]). ^{13}C NMR (101 MHz, $CDCl_3$) δ 141.36, 141.14, 138.14, 129.84, 129.04, 128.56, 128.47, 126.95, 126.14, 126.10, 42.01, 21.54, (141.89, 128.57, 38.08, 1,2-diphenylethane[33]).

These data are in full agreement with those reported in literature[28]

1-benzyl-2-methylbenzene (**23**)

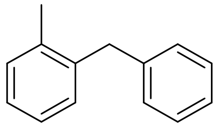

According to optimized procedure using methyl 2-bromotoluene (34 mg, 0.2 mmol) and potassium benzyltrifluoroborate (99 mg, 0.5 mmol, 2.5 equiv.). NMR yield: 45%.

The title compound was isolated by flash chromatography (100% hexane) as a colorless oil (12 mg, 0.07 mmol, 33%) that contains 6% of 1,2-diphenylethane as inseparable impurity.

^{1}H NMR (400 MHz, $CDCl_3$) δ 7.37 (t, J = 7.2 Hz, 2H), 7.31 – 7.17 (m, 7H), 4.09 (s, 2H), 2.34 (s, 3H), (2.95 (s, 4H) 1,2-diphenylethane[33]). ^{13}C NMR (101 MHz, $CDCl_3$) δ 140.49, 139.05, 136.78, 130.40, 130.06, 128.87, 128.51, 126.58, 126.11, 126.04, 39.56, 19.83, (141.91, 128.58, 128.46, 38.08, 1,2-diphenylethane[33]).

These data are in full agreement with those reported in literature.[28]

1-benzyl-4-(tert-butyl)benzene (**24**)

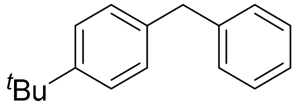

According to optimized procedure using 0.5 mmol 4-tert-butylbenzyl bromide (92 µl, 0.5 mmol) and potassium benzyltrifluoroborate (247.5 mg, 1.25 mmol). NMR yield: >99%.

The title compound was isolated by column chromatography (100% hexane) as a colorless oil (109.8 mg, 0.49 mmol, 98%) that contains 9% of 1,2-diphenylethane as inseparable impurity.

^{1}H NMR (400 MHz, $CDCl_3$) δ 7.44 – 7.34 (m, 4H), 7.33 – 7.27 (m, 3H), 7.25 – 7.18 (m, 2H), 4.05 (s, 2H), 1.40 (s, 9H), (3.02 (s, 4H), 1,2-diphenylethane[33]). ^{13}C NMR (101 MHz, $CDCl_3$) δ 148.93, 141.39, 138.21, 129.09, 128.63, 128.56, 126.12, 125.48, 41.55, 34.49, 31.53, (141.89, 128.46, 126.05, 38.10, 1,2-diphenylethane[33]).

These data are in full agreement with those reported in literature.[28]

1-benzyl-4-methoxybenzene (**8**)

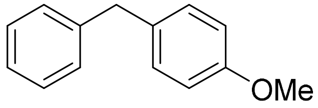

According to optimized procedure using 1-bromo-4-methoxybenzene (37 mg, 0.2 mmol) and potassium benzyltrifluoroborate (99 mg, 0.5 mmol, 2.5 equiv.). NMR yield: 97%.

The title compound was isolated by preperative thin layer chromatography (15% EA in hexanes) as a colorless liquid (34 mg, 0.171 mmol, 86%). ^{1}H NMR (400 MHz, $CDCl_3$) δ 7.33 – 7.25 (m, 2H), 7.23 – 7.16 (m, 3H), 7.11 (d, J = 8.0 Hz, 2H), 6.83 (d, J = 8.4 Hz, 2H), 3.94 (s, 2H), 3.79 (s, 3H). ^{13}C NMR (101 MHz, $CDCl_3$) δ 158.10, 141.72, 133.39, 130.00, 128.95, 128.57, 126.11, 114.01, 55.39, 41.17.

These data are in full agreement with those reported in literature.[35]

5-benzylbenzofuran (**25**)

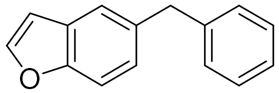

According to optimized procedure using 3-bromobenzofuran (39 mg, 0.2 mmol) and potassium benzyltrifluoroborate (99 mg, 0.5 mmol, 2.5 equiv.). NMR yield: >99%.

The title compound was isolated by column chromatography (10% EA in hexanes) as a colorless oil (38 mg, 0.182 mmol, 91%). that contains 8% of 1,2-diphenylethane as inseparable impurity. ^{1}H NMR (400 MHz, $CDCl_3$) δ 7.57 (s, 1H), 7.43 – 7.35 (m, 2H), 7.30 – 7.23 (m, 2H), 7.22 – 7.17 (m, 3H), 7.12 (d, J = 8.5 Hz, 1H), 6.68 (s, 1H), 4.06 (s, 2H), (2.92 (s, 4H) 1,2-diphenylethane[33]). ^{13}C NMR (101 MHz, $CDCl_3$) δ 153.82, 145.31, 141.79, 135.78, 129.02, 128.60, 127.74, 126.17, 125.58, 121.26, 111.32, 106.61, 41.92.

These data are in full agreement with those reported in literature.[34]

2-benzylpyridine (**26**)

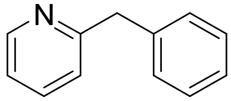

According to optimized procedure using methyl 2-bromopyridine (32 mg, 0.2 mmol) and potassium benzyltrifluoroborate (99 mg, 0.5 mmol, 2.5 equiv.). NMR yield: 87%.

The title compound was isolated by preperative thin layer chromatography (15% EA in hexanes) as a colorless oil (29 mg, 0.171 mmol, 85%). ^{1}H NMR (400 MHz, $CDCl_3$) δ 8.55 (d, *J* = 3.2 Hz, 1H), 7.57 (td, *J* = 7.6, 2.0 Hz, 1H), 7.35 – 7.19 (m, 5H), 7.14 – 7.07 (m, 2H), 4.16 (s, 2H). ^{13}C NMR (101 MHz, $CDCl_3$) δ 161.14, 149.46, 139.62, 136.70, 129.25, 128.73, 126.53, 123.27, 121.39, 44.83.

These data are in full agreement with those reported in literature.[36]

5-benzylpyrimidine (**27**)

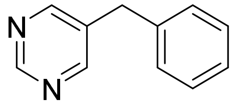

According to optimized procedure using methyl 5-bromopyrimidine (32 mg, 0.2 mmol) and potassium benzyltrifluoroborate (99 mg, 0.5 mmol, 2.5 equiv.). NMR yield: 88%.

The title compound was isolated by preperative thin layer chromatography (60% EA in hexanes) as a colorless liquid (26 mg, 0.152 mmol, 76%). ^{1}H NMR (400 MHz, $CDCl_3$) δ 9.08 (s, 1H), 8.58 (s, 2H), 7.37 – 7.30 (m, 2H), 7.29 – 7.23 (m, 1H), 7.17 (d, *J* = 7.2 Hz, 2H), 3.97 (s, 2H). ^{13}C NMR (101 MHz, $CDCl_3$) δ 157.14, 157.06, 138.41, 134.42, 129.10, 128.90, 127.11, 36.69.

These data are in full agreement with those reported in literature.[35]

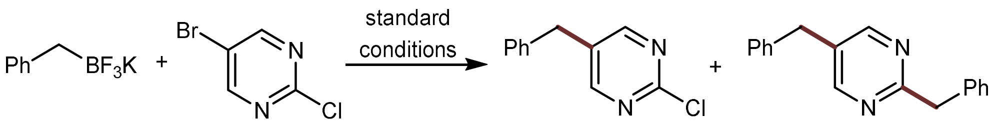

According to the optimized procedure using methyl 5-bromo-2-chloropyrimidine (39 mg, 0.2 mmol) and potassium benzyltrifluoroborate (99 mg, 0.5 mmol, 2.5 equiv.).

5-benzyl-2-chloropyrimidine (**29**)

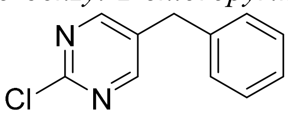

NMR yield: 24%. The title compound was isolated by preperative thin layer chromatography (50% EA in hexanes) as a colorless oil (10 mg, 0.049 mmol, 24%). ^{1}H NMR (400 MHz, $CDCl_3$) δ 8.46 (s, 2H), 7.38 – 7.31 (m, 2H), 7.30 – 7.24 (m, 1H), 7.16 (d, J = 6.8 Hz, 2H), 3.96 (s, 2H). ^{13}C NMR (101 MHz, $CDCl_3$) δ 159.76, 159.67, 137.85, 133.07, 129.25, 128.84, 127.34, 35.84. HRMS (ESI) m/z calcd for $C_{11}H_9ClN_2$ [$(M+H)^+$] 205.0527, found 205.0529.

2,5-dibenzylpyrimidine (**30**)

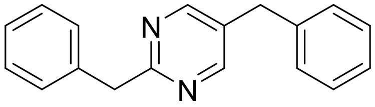

NMR yield: 62%.

The title compound was isolated by preperative thin layer chromatography (50% EA in hexanes) as a white solid (30 mg, 0.115 mmol, 58%). ^{1}H NMR (400 MHz, $CDCl_3$) δ 8.51 (s, 2H), 7.38 – 7.14 (m, 10H), 4.27 (s, 2H), 3.91 (s, 2H). ^{13}C NMR (101 MHz, $CDCl_3$) δ 168.03, 157.53, 138.65, 138.48, 131.30, 129.28, 129.02, 128.87, 128.66, 126.98, 126.69, 45.73, 36.35. HRMS (ESI) m/z calcd for $C_{18}H_{16}N_2$ [$(M+H)^+$] 261.1386, found 261.1386.

4-(4-(trifluoromethyl)benzyl)-1,1'-biphenyl (**31**)

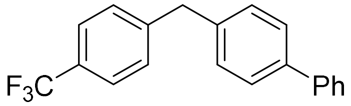

According to optimized procedure using 4-bromobenzotrifluoride (45 mg, 0.2 mmol) and potassium ([1,1′-biphenyl]-4-ylmethyl)trifluoroborate (137 mg, 0.5 mmol, 2.5 equiv.). NMR yield: 95%.

The title compound was isolated by column chromatography (1% EA in hexanes) as a white solid (55 mg, 0.176 mmol, 88%).

^{1}H NMR (400 MHz, $CDCl_3$) δ 7.61 – 7.52 (m, 6H), 7.43 (td, J = 7.7, 1.7 Hz, 2H), 7.37 – 7.30 (m, 3H), 7.25 (d, J = 8.2 Hz, 2H), 4.07 (s, 2H). ^{13}C NMR (101 MHz, $CDCl_3$) δ 145.25, 140.94, 139.61, 139.20, 129.48, 129.37, 128.92, 128.69 (q, J = 32.3 Hz), 127.54, 127.37, 127.16, 125.60 (q, J = 3.8 Hz), 124.46 (q, J = 271.8 Hz), 41.49. ^{19}F NMR (564 MHz, $CDCl_3$) δ -62.24.

These data are in full agreement with those reported in literature.[37]

1-(tert-butyl)-4-(4-(trifluoromethyl)benzyl)benzene (**32**)

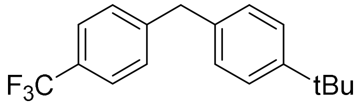

According to optimized procedure using 4-bromobenzotrifluoride (45 mg, 0.2 mmol) and (4-(tert-butyl)benzyl)trifluoroborate (127 mg, 0.5 mmol, 2.5 equiv.). NMR yield: >99%.

The title compound was isolated by column chromatography (1% EA in hexanes) as a yellow oil (56 mg, 0.191 mmol, 96%).

^{1}H NMR (400 MHz, $CDCl_3$) δ 7.55 (d, J = 8.0 Hz, 2H), 7.37 – 7.30 (m, 4H), 7.12 (d, J = 7.9 Hz, 2H), 4.01 (s, 2H), 1.32 (s, 9H). ^{13}C NMR (101 MHz, $CDCl_3$) δ 149.45, 145.55, 137.10, 129.35, 128.68, 128.51 (q, J = 32.3 Hz), 125.70, 125.50 (q, J = 3.9 Hz), 124.48 (q, J = 271.7 Hz), 41.35, 34.55, 31.50. ^{19}F NMR (564 MHz, $CDCl_3$) δ -62.24.

These data are in full agreement with those reported in literature.[39]

2-(4-(trifluoromethyl)benzyl)naphthalene (**33**)

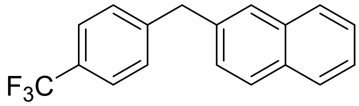

According to optimized procedure using of 4-bromobenzotrifluoride (45 mg, 0.2 mmol) and trifluoro(naphthalen-2-ylmethyl)borate (124 mg, 0.5 mmol, 2.5 equiv.). NMR yield: 93%.

The title compound was isolated by column chromathography (1% EA in hexanes) as a white solid (46 mg, 0.16 mmol, 80%).

^{1}H NMR (400 MHz, $CDCl_3$) δ 7.74 – 7.66 (m, 3H), 7.53 (s, 1H), 7.45 (d, *J* = 8 Hz, 2H), 7.41 – 7.32 (m, 2H), 7.26 – 7.21 (d, *J* = 7.6 Hz, 2H), 7.20 – 7.17 (dd, *J* = 8.4, 1.6 Hz, 1H), 4.09 (s, 2H). ^{13}C NMR (101 MHz, $CDCl_3$) δ 145.21, 137.57, 133.70, 132.31, 129.42, 128.60 (q, *J* = 31.3 Hz), 128.50, 127.81, 127.69, 127.51, 127.42, 126.34, 125.78, 125.56 (q, *J* = 3.8 Hz), 124.44 (q, *J* = 270.0 Hz), 41.98. ^{19}F NMR (564 MHz, $CDCl_3$) δ -62.27.

These data are in full agreement with those reported in literature.[38]

1-methoxy-4-(4-(trifluoromethyl)benzyl)benzene (**34**)

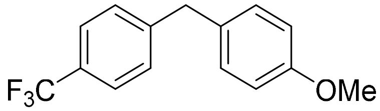

According to optimized procedure using 4-bromobenzotrifluoride (45 mg, 0.2 mmol) and potassium (4-methoxybenzy) trifluoroborate (114 mg, 0.5 mmol, 2.5 equiv.). NMR yield: >99%.

The title compound was isolated by preperative thin layer chromatography (4% EA in hexanes) as a pale yellow oil (45.1 mg, 0.169 mmol, 85%). ^{1}H NMR (400 MHz, $CDCl_3$) δ 7.53 (d, *J* = 8.0 Hz, 2H), 7.28 (d, *J* = 7.9 Hz, 2H), 7.12 – 7.05 (m, 2H), 6.87 – 6.82 (m, 2H), 3.97 (s, 2H), 3.79 (s, 3H). ^{13}C NMR (101 MHz, $CDCl_3$) δ 158.06, 145.52, 131.90, 129.73, 128.88, 128.2 (q, J= 32.3 Hz), 125.18 (q, J= 3.8 Hz), 124.14 (q, J= 272.7 Hz) 113.89, 55.10, 40.65. ^{19}F NMR (377 MHz, $CDCl_3$) δ -62.33.

These data are in full agreement with those reported in literature.[40]

5-(4-(trifluoromethyl)benzyl)benzo[d][1,3]dioxole (**35**)

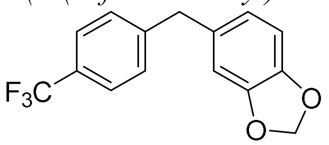

Reaction was performed in 0.1 mmol scale to avoid the formations of the homocoupling side product that is produced due the excess of the trifluoroborate. According to optimized procedure using of 4-bromobenzotrifluoride (22 mg, 0.1 mmol, 1 equiv.), and potassium (benzo[*d*][1,3]-dioxol-5-ylmethyl)trifluoroborate (60 mg, 0.25 mmol, 2.5 equiv.). NMR yield: 91%. The title compound was isolated by column chromatography (10-20% EA in hexanes) as a colorless oil, (21 mg, 0,074 mmol, 74%)

^{1}H NMR (400 MHz, $CDCl_3$) δ 7.53 (d, *J* = 8.1 Hz, 2H), 7.28 (d, *J* = 8.0 Hz, 2H), 6.75 (d, *J* = 7.7 Hz, 1H), 6.66 – 6.63 (m, 2H), 5.93 (s, 2H), 3.94 (s, 2H). ^{13}C NMR (101 MHz, CDCl3) δ= 148.00 146.29, 145.5, 133.91, 129.18, 128.62 (q, *J* = 32.2 Hz), 125.55 (q, *J* = 3.7 Hz), 124.41 (q, *J* = 272.9), 121.98, 109.50, 108.47, 101.11, 41.51 ppm. ^{19}F NMR (564 MHz, $CDCl_3$) δ -62.33.
These data are in full agreement with those reported in literature.[41]

1,3-dimethoxy-5-(4-(trifluoromethyl)benzyl)benzene (**36**)

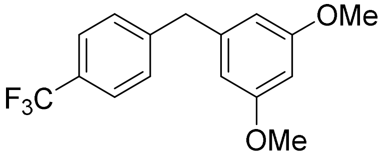

According to optimized procedure using 4-bromobenzotrifluoride (45 mg, 0.2 mmol) and potassium (3,5-dimethoxybenzyl)trifluoroborate (130 mg, 0.5 mmol, 2.5 equiv). NMR yield 85 %. The title compound was isolated by column chromatography (5-15% EA in hexanes) as a colorless liquid (38.5 mg, 0.13 mmol, 65%).

^{1}H NMR (400 MHz, $CDCl_3$) δ 7.53 (d, *J* = 8.0 Hz, 2H), 7.30 (d, *J* = 7.9 Hz, 2H), 6.34– 6.32 (m, 1H), 3.96 (s, 2H), 3.76 (s, 6H). ^{13}C NMR (201 MHz, $CDCl_3$) δ 161.28, 145.13, 142.53, 129.45, 128.83 (q, *J* = 32.4 Hz) 125.69 (q, *J* = 3.7 Hz), 124.59 (q, *J* = 271.9 Hz), 107.46, 98.45, 55.58, 42.21. ^{19}F NMR (654 MHz, $CDCl_3$) δ -62.33. HRMS (ESI) m/z calcd for $C_{16}H_{16}F_3O_2$ [$(M+H)^+$] 297.1102, found 297.0748.

1-chloro-4-(4-(trifluoromethyl)benzyl)benzene (**37**)

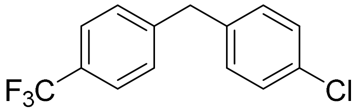

According to optimized procedure using 0.2 mmol of 4-bromobenzotrifluoride (45 mg, 0.2 mmol.) and potassium (4-chlorobenzyl)trifluoroborate (137 mg, 0.5 mmol, 2.5 equiv.). NMR yield: 60%. The title compound was isolated by column chromatography (5% EA in hexanes) as a yellow oil (32 mg, 0.120 mmol, 60%) that contains 8% of 1,2-bis(4-chlorophenyl)ethane as inseparable impurity.

^{1}H NMR (400 MHz, $CDCl_3$) δ 7.54 (d, *J* = 8.0 Hz, 2H), 7.27 (d, *J* = 8.2 Hz, 4H), 7.10 (d, *J* = 8.2 Hz, 2H), 4.00 (s, 2H), 2.88 (s, 4H, 1,2-bis(4-chlorophenyl)ethane[42]) ^{13}C NMR (101 MHz, $CDCl_3$) δ 144.75 (m), 138.56, 132.50, 130.40, 129.28, 128.93, 128.85 (d, *J* = 32.3 Hz), 125.65 (q, *J* = 3.8 Hz), 124.36 (d, *J* = 271.5z Hz), 41.14, (129.98, 128.60, 37.17: 1,2-bis(4-chlorophenyl)ethane[42]). ^{19}F NMR (564 MHz, $CDCl_3$) δ -62.36.

These data are in full agreement with those reported in literature.[43]

1-bromo-4-(4-(trifluoromethyl)benzyl)benzene (**38**)

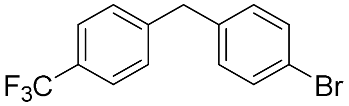

According to optimized procedure using 0.2 mmol of 4-bromobenzotrifluoride (45 mg, 0.2 mmol.) and potassium (4-bromobenzyl)trifluoroborate (138 mg, 0.5 mmol, 2.5 equiv.). NMR yield: 37%. The title compound was isolated by column chromatography (1% EA in hexanes) as a yellow oil (23 mg, 0.073 mmol, 36%).

^{1}H NMR (400 MHz, $CDCl_3$) δ 7.54 (d, *J* = 8.0 Hz, 2H), 7.45 – 7.40 (m, 2H), 7.27 (d, *J* = 8.0 Hz, 2H), 7.07 – 7.02 (m, 2H), 3.98 (s, 2H). ^{13}C NMR (101 MHz, $CDCl_3$) δ 144.63, 139.07, 131.88, 130.79, 129.28, 128.84 (q, *J* = 32.3 Hz), 125.65 (q, *J* = 3.8 Hz), 124.34 (q, *J* = 272.7 Hz), 120.52, 41.19. ^{19}F NMR (564 MHz, $CDCl_3$) δ -62.37. HRMS (EI) m/z calcd for $C_{14}H_{10}BrF_3$ [$(M)^+$] 314.9996, found 314.9959.

2,4-difluoro-1-(4-(trifluoromethyl)benzyl)benzene (**39**)

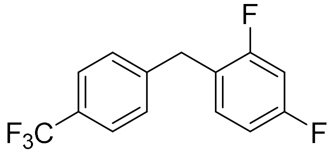

According to optimized procedure using 4-bromobenzotrifluoride (45 mg, 0.2 mmol) and potassium (2,4-difluorobenzyl)trifluoroborate (117 mg, 0.5 mmol, 2.5 equiv). NMR yield: 34 %. The title compound was isolated by column chromatography (100% hexane) as a colorless oil (15 mg, 0.044 mmol, 22%), which contained 16 % of 1,2-bis(2,4-difluorophenyl)ethane as inseparable impurity. Since the product was not reported in the literature, the experiment was repeated using less equivalents of the trifluoroborate salt (80 mg, 0.34 mmol, 1.7 equiv) to give the clean product as a colorless oil using hexanes (6 mg, 0.022 mmol, 11% yield).

^{1}H NMR (400 MHz, $CDCl_3$) δ 7.53 (d, *J* = 8.1 Hz, 2H), 7.29 (d, *J* = 8.0 Hz, 2H), 7.14 – 7.06 (m), 6.85 – 6.79 (m, 1H), 4.01 (s, 2H). ^{13}C NMR (101 MHz, $CDCl_3$) δ 162.69 (dd, *J* = 108.07, 11.81 Hz), 160.22 (dd, *J* = 108.07, 11.81 Hz), 143.79, 131.61 (dd, *J* = 9.5, 6 Hz), 129.08, 128.89 (q, *J* = 32.4 Hz), 125.66 (q, *J* = 3.7 Hz), 123.01 (dd, *J* = 16.16, 3.83 Hz), 124.33 (q, *J* = 272.90 Hz), 111.48 (dd, *J* = 21.10, 3.73 Hz), 104.11 (t, *J* = 25.65 Hz), 34,35 (d, *J* = 2 Hz). ^{19}F NMR (564 MHz, $CDCl_3$) δ -62.31 (3F), -112.21 (1F), -113.25 (1F). HRMS (EI) m/z calcd for $C_{14}H_{10}F_5$ [(M^+)] 272.0624, found 272.0631

4-(4-(trifluoromethyl)benzyl)benzonitrile (**40**)

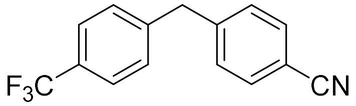

According to optimized procedure using 0.2 mmol of 4-bromobenzotrifluoride (45 mg, 1 equiv.) and potassium (4-cyanobenzyl)trifluoroborate (112 mg, 0.5 mmol, 2.5 equiv.). NMR yield: 37%. The title compound was isolated by column chromatography (1% EA in hexanes) as a yellow oil (12 mg, 0.046 mmol, 23%).

^{1}H NMR (400 MHz, $CDCl_3$) δ 7.61 – 7.54 (m, 4H), 7.26 (d, *J* = 8 Hz, 4H), 4.08 (s, 2H). ^{13}C NMR (101 MHz, $CDCl_3$) δ 145.61, 143.49, 132.62, 129.80, 129.41, 129.07, 125.85 (q, *J* = 3.8 Hz), 124.23 (q, *J* = 272.1 Hz), 118.92, 110.66, 41.80. ^{19}F NMR (564 MHz, $CDCl_3$) δ -62.46.

These data are in full agreement with those reported in literature.[44]

Mechanistic Studies

Luminescence quenching

In a glove box, a volumetric flask was charged with 4,4′-Czbpy (1 equiv.) and $NiBr_2$·glyme (1 equiv.). Samples with the respective concentration were prepared using spectroscopic grade DMSO, transferred into a sealable quartz cuvette equipped and taken out of the glove box for recording emission spectra at the respective excitation wavelength. Afterwards, $BnBF_3K$ or 4-bromobenztrifluoride (500 equiv.) was added to the quartz cuvette (inside a glove box) and the sample was measured again with the same instrument settings.

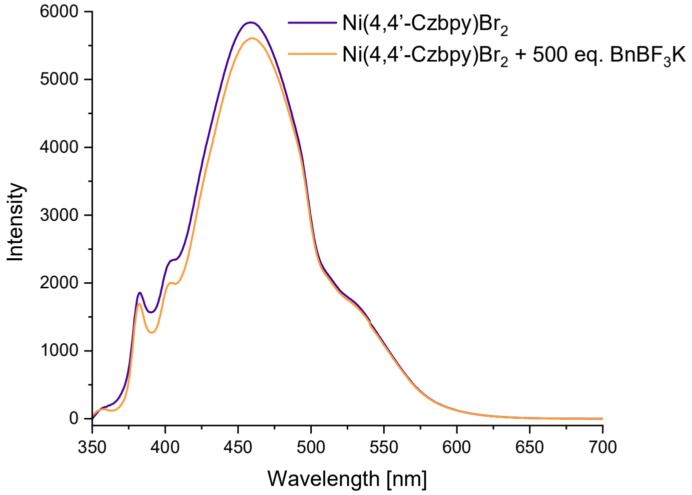

Fig. S56. Quenching study at $\lambda_{excitation}$ = 330 nm. Concentration of Ni(4,4′-Czbpy)Br_2 = 2.5 µM, ex./em. bandwidth = 5/5 nm, response = 0.2 sec, sensitivity = medium, optical path = 1 cm

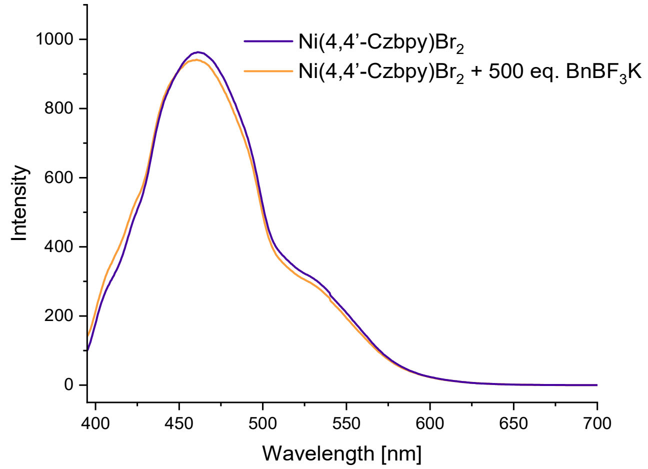

Fig. S 57. Quenching study at $\lambda_{excitation}$ = 380 nm. Concentration of Ni(4,4′-Czbpy)Br_2 = 10 µM, ex./em. bandwidth = 5/5 nm, response = 0.5 sec, sensitivity = high, optical path = 1 cm**.**

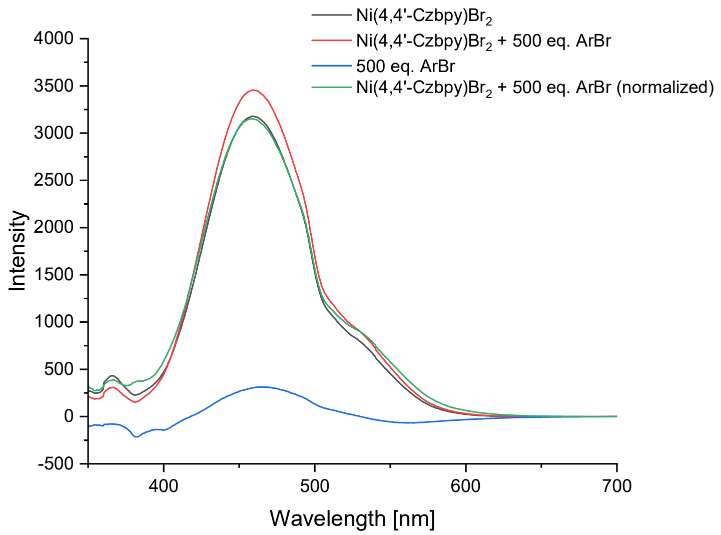

Fig. S58. Quenching study at $\lambda_{excitation}$ = 330 nm. Concentration of Ni(4,4′-Czbpy)Br_2 = 2.5 μM, ex./em. bandwidth = 5/5 nm, response = 0.2 sec, sensitivity = medium, optical path = 1 cm. ArBr = 4-bromobenzotrifluoride (**2**).

CV studies

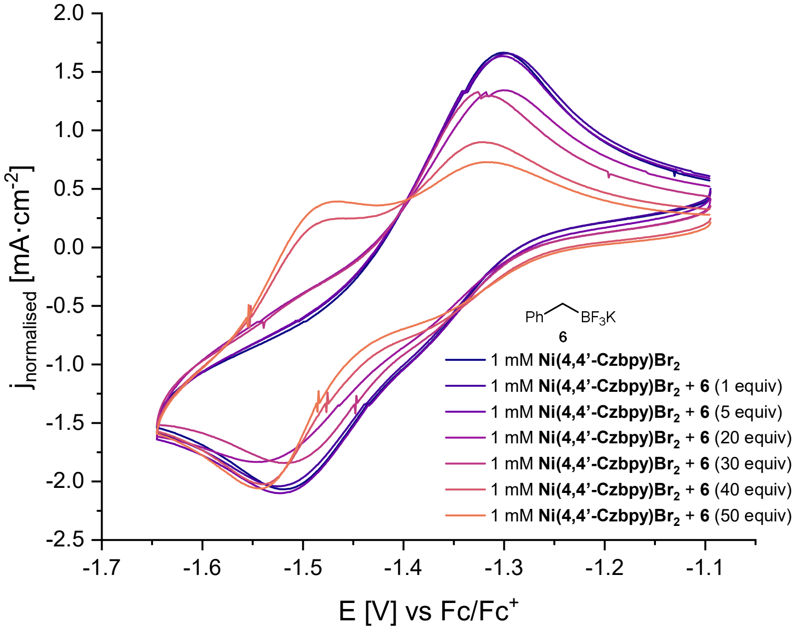

Fig. S59: CV of 1 mM of Ni(4,4′-Czbpy)Br_2 with addition of potassium benzyltrifluoroborate (**6**) in DMAc with 0.1 M Bu_4NBr as supporting electrolyte at 100 mV·s^{-1}.

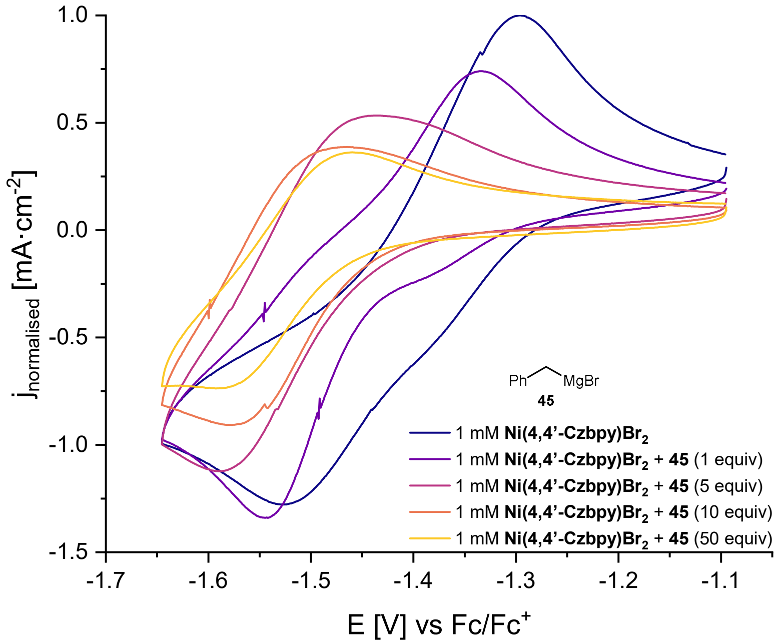

Fig. S60: CV of 1 mM of Ni(4,4′-Czbpy)Br_2 with addition of benzylmagnesium bromide (**45**) in DMAc with 0.1 M Bu_4NBr as supporting electrolyte at 100 mV·s^{-1}.

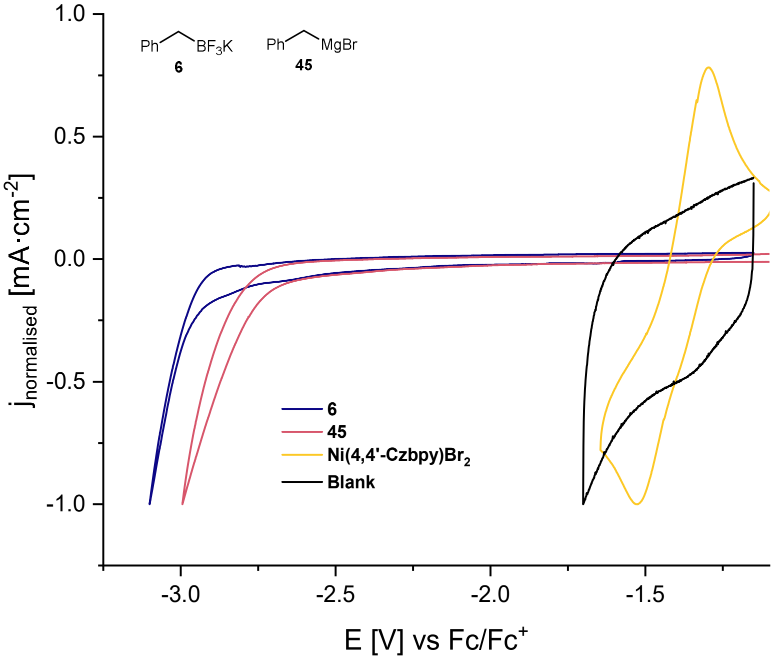

Fig. S61: CVs of blank, 1 mM Ni(4,4′-Czbpy)Br_2, 1 mM potassium benzyltrifluoroborate (**6**), and 1 mM benzylmagnesium bromide (**45**) in DMAc with 0.1M Bu_4NBr as supporting electrolyte at 100 mV·s^{-1}.

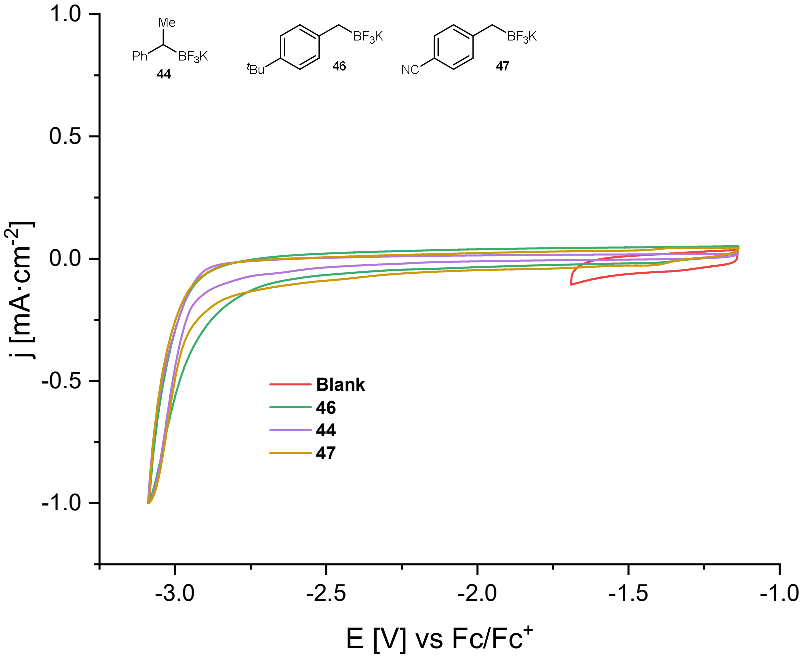

Fig. S62: CVs of blank, 1 mM potassium trifluoro(1-phenylethyl)borate (**44**), 1 mM potassium [4-(tert-butyl)benzyl]trifluoroborate (**46**) and 1 mM potassium [4-(cyano)benzyl]trifluoroborate (**47**) in DMAc with 0.1 M Bu_4NBr as supporting electrolyte at 100 mV·s^{-1}.

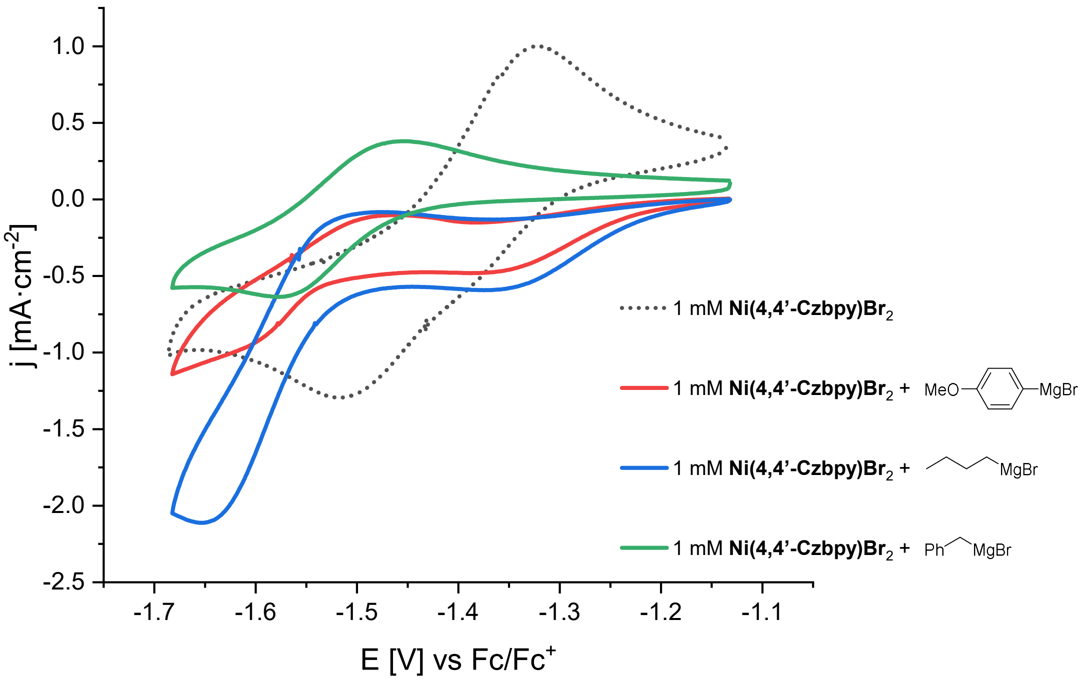

Fig. S63: CV of 1 mM of Ni(4,4′-Czbpy)Br_2 with addition of different Grignard reagents (50 equiv), 0.1 M Bu_4NBr as supporting electrolyte at 100 mV·s^{-1}.

UV/Vis studies

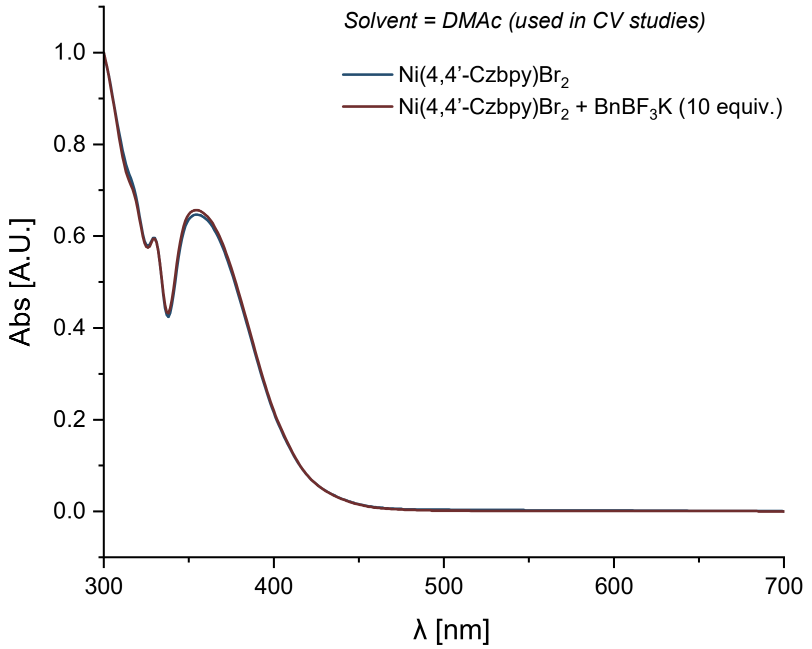

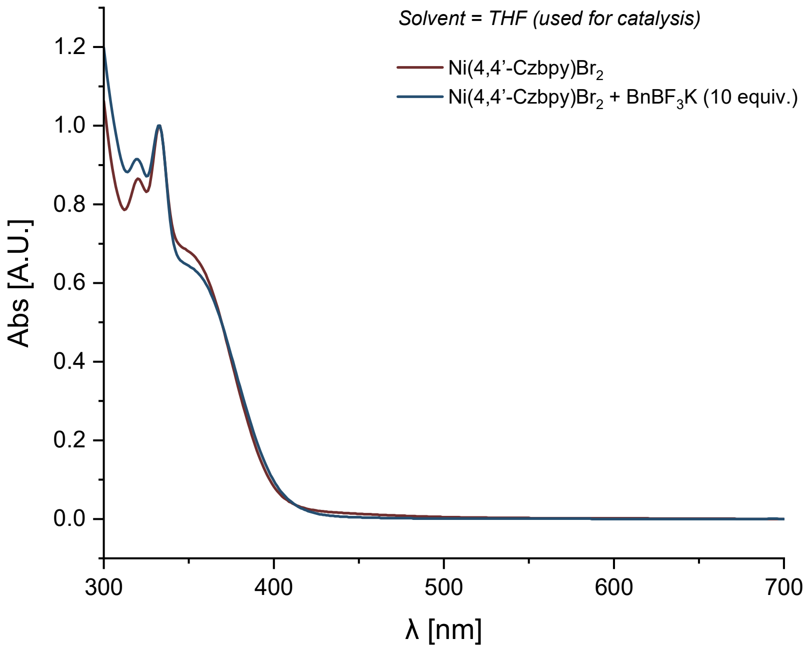

Fig. S64: UV-Vis absorption spectra of Ni(4,4′-Czbpy (50 μM) with and without addion of $BnBF_3K$ (10 equiv) in DMAc (top, solvent was used for CV studies) and THF (bottom, solvent was used in photocatalytic C(sp^3)–C(sp^2) cross-couplings) suggest that transmetalation does not occur in case of the Ni^{II} pre-catalyst.

Experiments using $Ni(COD)_2$ and (4,4′-Czbpy)Ni(o-Tol)Br

Catalytic reactions with $Ni(COD)_2$ or (4,4′-Czbpy)Ni(o-Tol)Br were carried out according to the standard procedure. The only difference is that reaction mixtures were prepared in a glove box.

Synthesis of (4,4′-Czbpy)Ni(o-Tol)Br

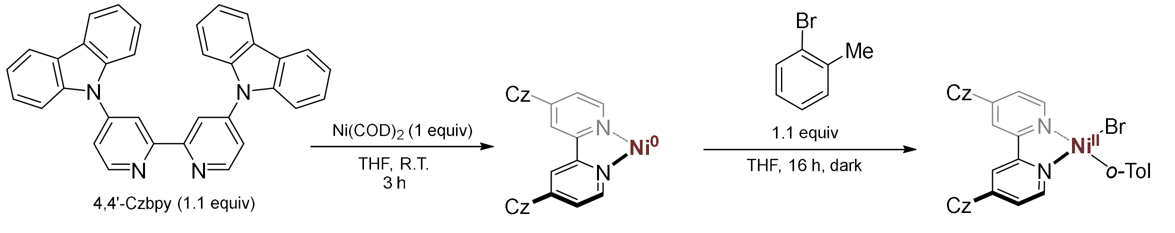

(4,4'-Czbpy)Ni(*o*-Tol)Br

Experimental procedure. In a glovebox, an oven dried microwave vial (19 x 100 mm) equipped with a stir bar was charged with $Ni(COD)_2$ (55 mg, 0.20 mmol, 1.0 equiv) and 4,4'-Czbpy (107 mg, 0.22 mmol, 1.1 equiv) in THF (anhydrous, inhibitor-free, 5 mL) sealed and stirred for 3 hours. To this solution, 2-bromotoluene (66 μL, 0.66 mmol, 3.3 equiv) was added dropwise. The resulting reaction mixture was stirred for 16 h in the dark. The resulting dark red solution was transferred to an amber glass vial and 20 mL of anhydrous pentane were added. The solution was kept in the freezer (-35 °C) for 16 h. The precipitate was filtered under vacuum, and dried over 48 h on a Schlenk line. The product was obtained as a dark red precipitate (105 mg, 0.15 mmol, 73%; containing ~15% of unbound 4,4'-Czbpy as an inseperable impuritiy). The complex was used in catalytic reactions without additional purification.

^{1}H NMR (400 MHz, THF) δ 9.80 (d, 1H), 8.71 (d, J = 7.6 Hz, 2H), 8.17 – 8.09 (m, 4H), 8.04 (d, J = 4.2 Hz, 1H), 7.78 – 7.66 (m, 6H), 7.62 (d, J = 7.3 Hz, 1H), 7.46 – 7.37 (m, 4H), 7.36 – 7.25 (m, 4H), 6.82 – 6.66 (m, 3H), 3.13 (s, 3H).

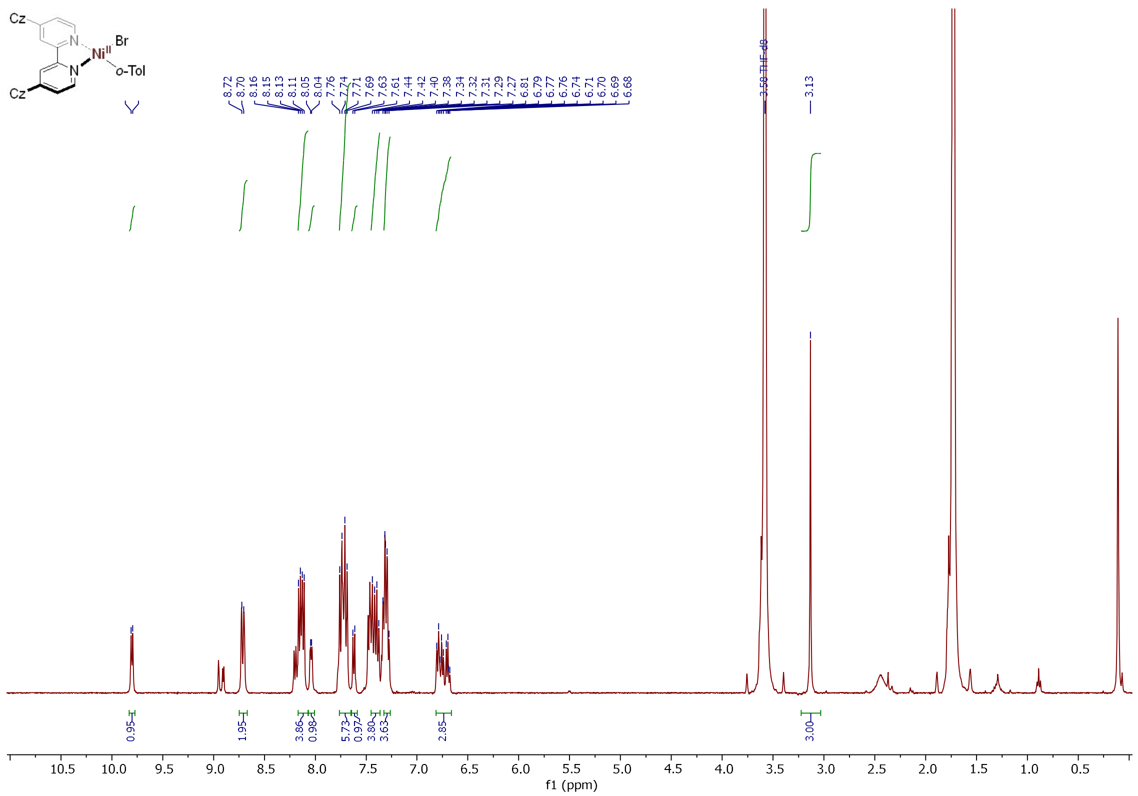

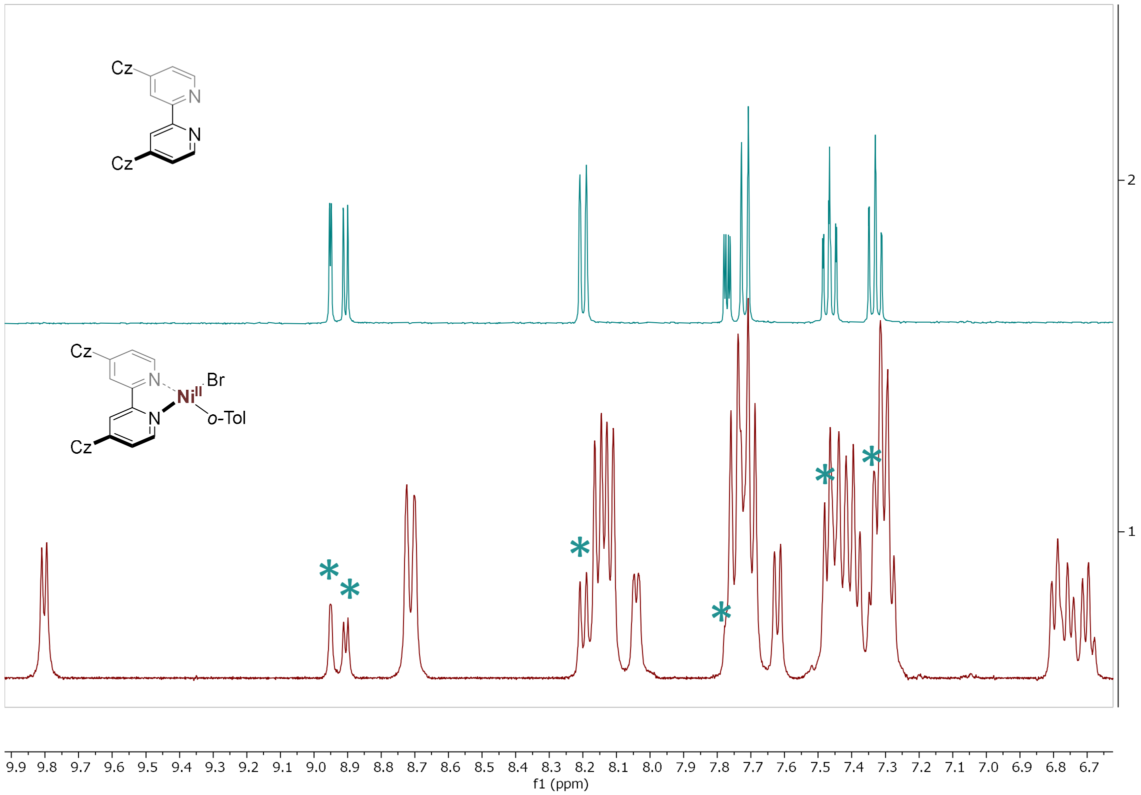

Fig. S65: ^{1}H-NMR of isolated (4,4′-Czbpy)Ni(o-Tol)Br used for catalytic experiments (top). The isolated complex contains unbound ligand (bottom, peaks highlighted with an asterisks) which was inseparable.

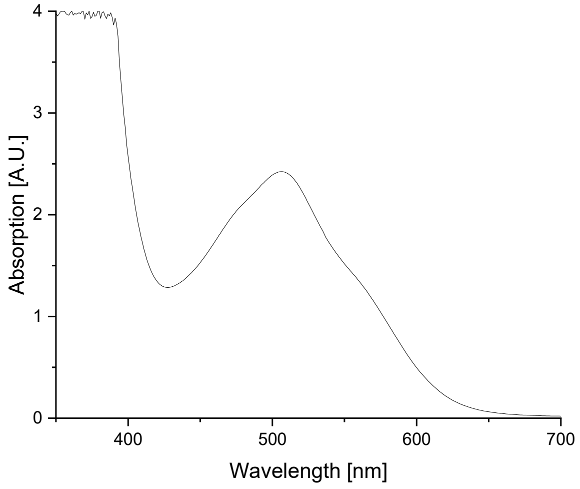

Fig. S66: UV-Vis absorption spectra of (4,4'-Czbpy)Ni(o-Tol)Br (0.5 mM) in THF.

Experiments using $(BCP)_2NiBr$

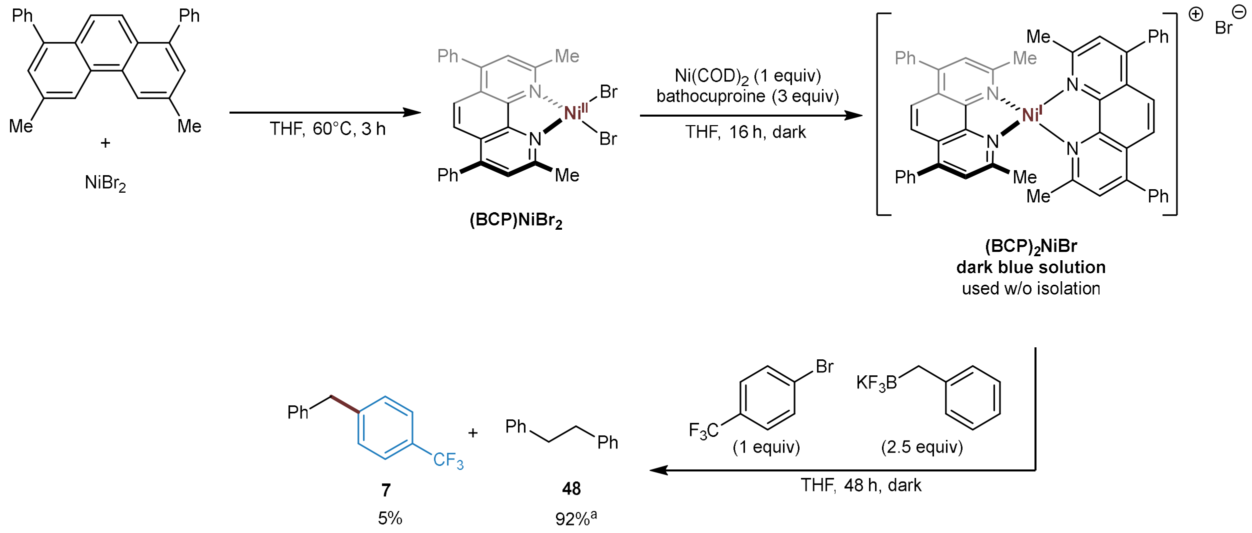

Experimental procedure.[45] An oven dried microwave vial (19 x 100 mm) equipped with a stir bar was charged with $NiBr_2$ (22 mg, 0.1 mmol) and bathocuproine (BCP, 36 mg, 0.1 mmol) in THF (anhydrous, inhibitor-free, 1 mL) sealed and stirred at 60 °C for 3 hours. Once cooled to room temperature, $(BCP)NiBr_2$ was precipitated with Et_2O (2 mL), filtered and washed with Et_2O (4 x 3 mL), to afford a pink powder that was dried overnight on a Schlenk line.

In a glovebox, a microwave vial (19 x 100 mm) wrapped in aluminium foil was equipped with a stir bar and charged with $(BCP)NiBr_2$ (58 mg, 0.1 mmol), $Ni(COD)_2$ (28 mg, 0.1 mmol), bathocuproine (108 mg, 0.3 mmol) and THF (anhydrous, inhibitor-free, 5 mL). The vial was sealed and the reaction mixture was stirred in the dark overnight. Formation of (BCP)NiBr was confirmed by the characteristic deep blue color of the resulting solution.[46]

To the reaction mixture, a solution of 4-bromobenzotrifluoride (23 mg, 14 μL, 0.1 mmol) and potassium benzyltrifluoroborate (50 mg, 0.25 mmol) in THF (anhydrous, inhibitor-free, 4 mL) were added via a syringe, the reaction mixture was subsequently stirred for an additional 48 hours and the resulting mixture was subsequently analyzed by by ^{1}H-NMR and ^{19}F-NMR using 1,3,5-trimethoxybenzene and fluorobenzene as internal standards.

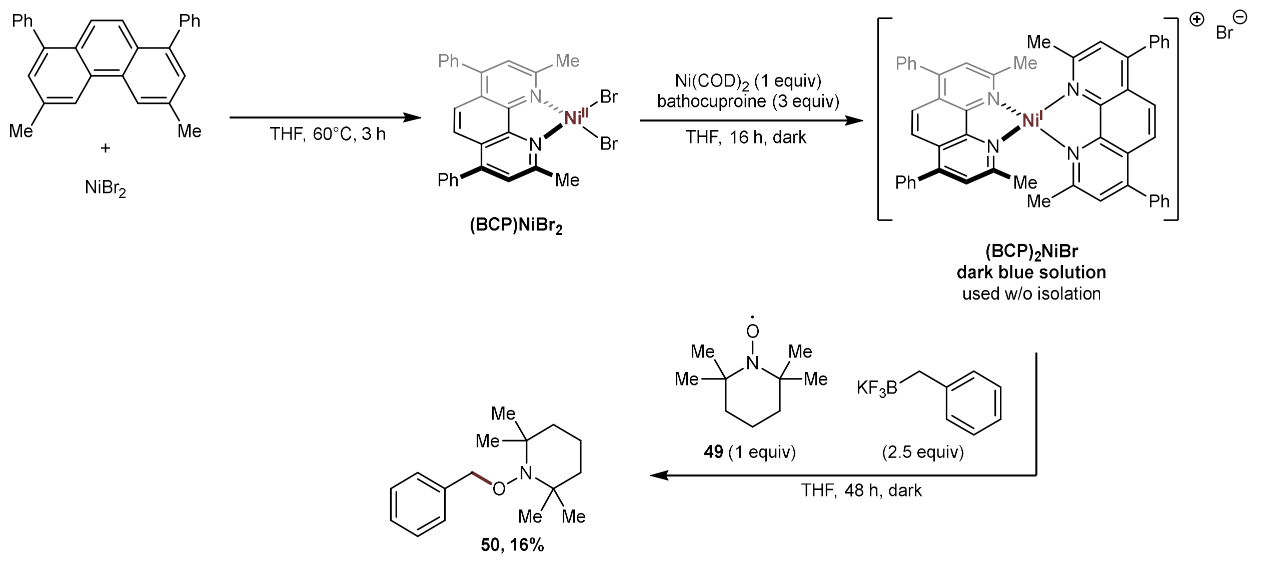

Experimental procedure.[45] An oven dried microwave vial (19 x 100 mm) equipped with a stir bar was charged with $NiBr_2$ (22 mg, 0.1 mmol) and bathocuproine (BCP, 36 mg, 0.1 mmol) in THF (anhydrous, inhibitor-free, 1 mL) sealed and stirred at 60 °C for 3 hours. Once cooled to room temperature, (BCP)$NiBr_2$ was precipitated with Et_2O (2 mL), filtered and washed with Et_2O (4 x 3 mL), to afford a pink powder that was dried overnight on a Schlenk line.

In a glovebox, a microwave vial (19 x 100 mm) wrapped in aluminium foil was equipped with a stir bar and charged with (BCP)$NiBr_2$ (58 mg, 0.1 mmol), Ni(COD)$_2$ (28 mg, 0.1 mmol), bathocuproine (108 mg, 0.3 mmol) and THF (anhydrous, inhibitor-free, 5 mL). The vial was sealed and the reaction mixture was stirred in the dark overnight. Formation of (BCP)NiBr was confirmed by the characteristic deep blue color of the resulting solution.[46]

To the reaction mixture, a solution of TEMPO (39 mg, 0.25 mmol) and potassium benzyltrifluoroborate (20 mg, 0.1 mmol) in THF (anhydrous, inhibitor-free, 4 mL) were added via a syringe, the reaction mixture was subsequently stirred for an additional 48 hours and the resulting mixture was analyzed by GC-MS and ^{1}H NMR. The product was detected in 16%, using 1,3,5-trimethoxybenzene as internal standard.

Radical trapping

An oven-dried microwave vial was charged with $NiBr_2$ (1.1 mg, 5 µmol, 5 mol%), 4,4′-dicarbazolyl-2,2′-bipyridyl (4,4′-Czbpy, 2.4 mg, 5 µmol, 5 mol%), 2,2,6,6-Tetramethylpiperidine 1-oxyl (TEMPO, 39 mg, 250 µmol, 2.5 equiv.), potassium trifluoroborate salt (19.8 mg, 0.1 mmol, 1 equiv.) and THF (anhydrous, inhibitor-free, 1 mL). The vessel was sealed, the mixture was sonicated for 20 minutes and degassed by freeze-pump-thaw (3 cycles). The reaction mixture was stirred at 800 rpm and irradiated with two LED lamps (440 nm) at full power. After 5 h, 1,3,5-trimethoxybenzene (33.6 mg, 200 µmol, 1.0 equiv.) was added to the reaction vessel, the mixture was stirred and an aliquote (~200 µL) was removed, diluted with $CDCl_3$ and analyzed by ^{1}H NMR to determine NMR yields.

Table S13: Radical trapping studies using TEMPO.[a]

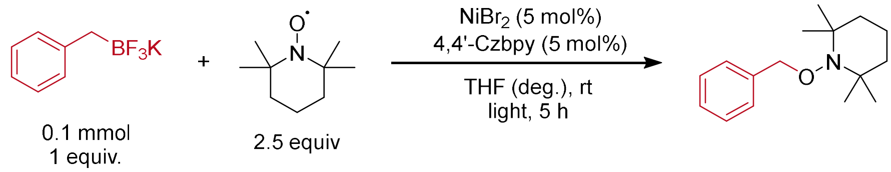

entry	variation	light source	Yield [%][b]
1	-	370 nm	22
2	w/o $NiBr_2$	370 nm	n.d.
3	-	390 nm	30
4	w/o $NiBr_2$	390 nm	n.d.
5	-	427 nm	14
6	w/o $NiBr_2$	427 nm	n.d.
7	-	440 nm	22
8	w/o $NiBr_2$	440 nm	n.d.

[a]Reaction conditions: potassium benzyltrifluoroborate (100 µmol), 2,2,6,6-Tetramethylpiperidine 1-oxyl (TEMPO, 250 µmol), $NiBr_2$ (5 µmol), 4,4′-Czbpy (5 µmol), THF (anhydrous, 1 mL), reaction mixture was degassed before irradiation with 440 nm LED (2 lamps at full power). [b]NMR yields determined by ^{1}H-NMR using 1,3,5-trimethoxybenzene as internal standard. n.d. = not detected.

An oven-dried microwave vial was charged with $NiBr_2$ (2.2 mg, 10 µmol, 5 mol%), 4,4'-dicarbazolyl-2,2'-bipyridyl (4,4'-Czbpy, 4.8 mg, 10 µmol, 5 mol%), methyl methacrylate (21.4 µL, 200 µmol, 1 equiv.), potassium benzyltrifluoroborate (99 mg, 500 µmol, 2.5 equiv.), 2,6-lutidine (47 µL, 400µmol, 2 equiv.) and THF (anhydrous, inhibitor-free, 1 mL). The vessel was sealed, and the mixture was sonicated for 20 minutes and degassed by freeze-pump-thaw (3 cycles). The reaction mixture was stirred at 800 rpm and irradiated with two LED lamps (440 nm) at full power. After 3 days, 1,3,5-trimethoxybenzene (33.6 mg, 200 µmol, 1.0 equiv.) was added to the reaction vessel, the mixture was stirred and the solvent was removed by rotary evaporation. Deuterated chloroform ($CDCl_3$) was added, and the mixture was sonicated and filtered through a pad of silica gel. An aliquot (~200 µL) was removed, diluted with $CDCl_3$, and analyzed by ^1H NMR to determine NMR yields.

Table S14: Radical trapping studies using methyl methacrylate.[a]

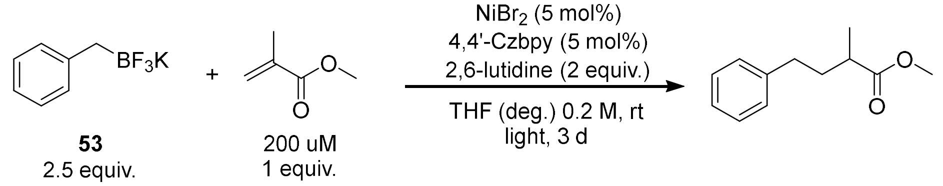

Entry	Variation	Light source	Yield [%][a]
1	dtbbpy (5 mol%) instead of 4,4'-Czbpy	440 nm	n.d.
2	-	440 nm	27
3	w/o $NiBr_2$	440 nm	n.d.
4	w/o 2,6-lutidine	440 nm	23
5	w/o 2,6-lutidine, w/o $NiBr_2$	440 nm	n.d.
6	w/o $NiBr_2$	370 nm	n.d.
7	w/o $NiBr_2$	390 nm	n.d.
7	w/o $NiBr_2$	427 nm	n.d.

Reaction conditions: potassium benzyltrifluoroborate (500 µmol), methyl methacrylate (200 µmol), $NiBr_2$ (10 µmol), 4,4'-Czbpy (10 µmol), THF (anhydrous, 1 mL), the reaction mixture was degassed before irradiation with 440 nm LED (2 lamps at full power). [a]NMR yields determined by ^1H-NMR using 1,3,5-trimethoxybenzene as internal standard. n.d. = not detected.

Radical trapping studies shown in Figure 5b were carried out using the optimized catalytic conditions. For each experiment, the respective amount of methyl acrylate was added.

References

(1) Li, H.-Y.; Wu, J.; Zhou, X.-H.; Kang, L.-C.; Li, D.-P.; Sui, Y.; Zhou, Y.-H.; Zheng, Y.-X.; Zuo, J.-L.; You, X.-Z. Synthesis, structural characterization and photoluminescence properties of rhenium(I) complexes based on bipyridine derivatives with carbazole moieties. *Dalton Trans.* **2009**, 10563-10569

(2) Luo, J.; Hu, B.; Wu, W.; Hu, M.; Liu, T. L. Nickel-Catalyzed Electrochemical C (sp3)− C (sp2) Cross-Coupling Reactions of Benzyl Trifluoroborate and Organic Halides. *Angew. Chem. Int. Ed.* **2021**, *60*, 6107-6116

(3) Liu, S.-P.; He, Y.-H.; Guan, Z. Photoredox-Catalyzed Radical–Radical Cross-Coupling of Sulfonyl Chlorides with Trifluoroborate Salts. *J. Org. Chem.* **2023**, *88*, 11161-11172

(4) Kovalenko, S. A.; Dobryakov, A. L.; Ruthmann, J.; Ernsting, N. P. Femtosecond spectroscopy of condensed phases with chirped supercontinuum probing. *Phys. Rev. A* **1999**, *59*, 2369-2384

(5) Kovalenko, S. A.; Schanz, R.; Hennig, H.; Ernsting, N. P. Cooling dynamics of an optically excited molecular probe in solution from femtosecond broadband transient absorption spectroscopy. *J. Chem. Phys.* **2001**, *115*, 3256-3273

(6) Moreno, J.; Dobryakov, A. L.; Ioffe, I. N.; Granovsky, A. A.; Hecht, S.; Kovalenko, S. A. Broadband transient absorption spectroscopy with 1- and 2-photon excitations: Relaxation paths and cross sections of a triphenylamine dye in solution. *J. Chem. Phys.* **2015**, *143*,

(7) Ioffe, I. N.; Quick, M.; Quick, M. T.; Dobryakov, A. L.; Richter, C.; Granovsky, A. A.; Berndt, F.; Mahrwald, R.; Ernsting, N. P.; Kovalenko, S. A. Tuning Stilbene Photochemistry by Fluorination: State Reordering Leads to Sudden Polarization near the Franck–Condon Region. *J. Am. Chem. Soc.* **2017**, *139*, 15265-15274

(8) Megerle, U.; Pugliesi, I.; Schriever, C.; Sailer, C. F.; Riedle, E. Sub-50 fs broadband absorption spectroscopy with tunable excitation: putting the analysis of ultrafast molecular dynamics on solid ground. *Appl. Phys. B* **2009**, *96*, 215-231

(9) Becke, A. D. Density-functional thermochemistry. III. The role of exact exchange. *J. Chem. Phys.* **1993**, *98*, 5648-5652

(10) Yanai, T.; Tew, D. P.; Handy, N. C. A new hybrid exchange–correlation functional using the Coulomb-attenuating method (CAM-B3LYP). *Chem. Phys. Lett.* **2004**, *393*, 51-57

(11) Grimme, S.; Antony, J.; Ehrlich, S.; Krieg, H. A consistent and accurate ab initio parametrization of density functional dispersion correction (DFT-D) for the 94 elements H-Pu. *J. Chem. Phys.* **2010**, *132*,

(12) McLean, A. D.; Chandler, G. S. Contracted Gaussian basis sets for molecular calculations. I. Second row atoms, Z=11–18. *J. Chem. Phys.* **1980**, *72*, 5639-5648

(13) Krishnan, R.; Binkley, J. S.; Seeger, R.; Pople, J. A. Self-consistent molecular orbital methods. XX. A basis set for correlated wave functions. *J. Chem. Phys.* **1980**, *72*, 650-654

(14) Wachters, A. J. H. Gaussian Basis Set for Molecular Wavefunctions Containing Third-Row Atoms. *J. Chem. Phys.* **1970**, *52*, 1033-1036

(15) Hay, P. J. Gaussian basis sets for molecular calculations. The representation of 3d orbitals in transition-metal atoms. *J. Chem. Phys.* **1977**, *66*, 4377-4384

(16) Creating UV/Visible Plots from the Results of Excited States Calculations. https://gaussian.com/uvvisplot/.

(17) Victor Talrose; Alexander N. Yermakov; Antonina A. Goncharova; Natalia A. Messineva; Natalia V. Trusova; Margarita V. Efimkina. UV/Visible Spectra. In NIST Chemistry WebBook, NIST Standard Reference Database Number 69; P.J. Linstrom, W.G. Mallard, Eds.; National Institute of Standards and Technology: Gaithersburg MD, 20899.

(18) Walba, H.; Branch, G. E. K. The Absorption Spectra of Some N-Substituted p-Aminotriphenylmethyl Ions. *J. Am. Chem. Soc.* **1951**, *73*, 3341-3348

(19) Davies, J.; Janssen-Müller, D.; Zimin, D. P.; Day, C. S.; Yanagi, T.; Elfert, J.; Martin, R. Ni-Catalyzed Carboxylation of Aziridines en Route to β-Amino Acids. *J. Am. Chem. Soc.* **2021**, *143*, 4949-4954

(20) Lin, Q.; Diao, T. Mechanism of Ni-Catalyzed Reductive 1,2-Dicarbofunctionalization of Alkenes. *J. Am. Chem. Soc.* **2019**, *141*, 17937-17948

(21) Ting, S. I.; Williams, W. L.; Doyle, A. G. Oxidative Addition of Aryl Halides to a Ni(I)-Bipyridine Complex. *J. Am. Chem. Soc.* **2022**, *144*, 5575-5582

(22) Cagan, D. A.; Bím, D.; McNicholas, B. J.; Kazmierczak, N. P.; Oyala, P. H.; Hadt, R. G. Photogenerated Ni(I)–Bipyridine Halide Complexes: Structure–Function Relationships for Competitive C(sp2)–Cl Oxidative Addition and Dimerization Reactivity Pathways. *Inorg. Chem.* **2023**, *62*, 9538-9551

(23) Cavedon, C.; Gisbertz, S.; Reischauer, S.; Vogl, S.; Sperlich, E.; Burke, J. H.; Wallick, R. F.; Schrottke, S.; Hsu, W.-H.; Anghileri, L.; Pfeifer, Y.; Richter, N.; Teutloff, C.; Müller-Werkmeister, H.; Cambié, D.; Seeberger, P. H.; Vura-Weis, J.; van der Veen, R. M.; Thomas, A.; Pieber, B. Intraligand Charge Transfer Enables Visible-Light-Mediated Nickel-Catalyzed Cross-Coupling Reactions. *Angew. Chem. Int. Ed.* **2022**, *61*, e202211433

(24) Saprin, A. N.; Piette, L. H. Spin trapping and its application in the study of lipid peroxidation and free radical production with liver microsomes. *Archives of Biochemistry and Biophysics* **1977**, *180*, 480-492

(25) Ghiazza, C.; Khrouz, L.; Billard, T.; Monnereau, C.; Tlili, A. Fluoroalkylselenolation of Alkyl Silanes/Trifluoroborates under Metal-Free Visible-Light Photoredox Catalysis. *Eur. J. Org. Chem.* **2020**, *2020*, 1559-1566

(26) Stoll, S.; Schweiger, A. EasySpin, a comprehensive software package for spectral simulation and analysis in EPR. *J. Magn. Res.* **2006**, *178*, 42-55

(27) Cavedon, C.; Gisbertz, S.; Reischauer, S.; Vogl, S.; Sperlich, E.; Burke, J. H.; Wallick, R. F.; Schrottke, S.; Hsu, W. H.; Anghileri, L. Intraligand charge transfer enables visible-light-mediated nickel-catalyzed cross-coupling reactions. *Angew. Chem. Int. Ed.* **2022**, *61*, e202211433

(28) Sirvinskaite, G.; Reisenbauer, J. C.; Morandi, B. Deaminative coupling of benzylamines and arylboronic acids. *Chem. Sci.* **2023**, *14*, 1709-1714

(29) Gil-Negrete, J. M.; Sestelo, J. P.; Sarandeses, L. A. Synthesis of bench-stable solid triorganoindium reagents and reactivity in palladium-catalyzed cross-coupling reactions. *Chem. Commun.* **2018**, *54*, 1453-1456

(30) Tang, J.; Lv, L.; Dai, X.-J.; Li, C.-C.; Li, L.; Li, C.-J. Nickel-catalyzed cross-coupling of aldehydes with aryl halides via hydrazone intermediates. *Chem. Commun.* **2018**, *54*, 1750-1753

(31) Srimani, D.; Bej, A.; Sarkar, A. Palladium nanoparticle catalyzed Hiyama coupling reaction of benzyl halides. *J. Org. Chem.* **2010**, *75*, 4296-4299

(32) Zhang, J.; Lu, G.; Xu, J.; Sun, H.; Shen, Q. Nickel-catalyzed reductive cross-coupling of benzyl chlorides with aryl chlorides/fluorides: a one-pot synthesis of diarylmethanes. *Org. Lett.* **2016**, *18*, 2860-2863

(33) Lundevall, F. J.; Elumalai, V.; Drageset, A.; Totland, C.; Bjørsvik, H. R. A Co2B mediated NaBH4 reduction protocol applicable to a selection of functional groups in organic synthesis. *Eur. J. Org. Chem.* **2018**, *2018*, 3416-3425

(34) Vasilopoulos, A.; Zultanski, S. L.; Stahl, S. S. Feedstocks to pharmacophores: Cu-catalyzed oxidative arylation of inexpensive alkylarenes enabling direct access to diarylalkanes. *J. Am. Chem. Soc.* **2017**, *139*, 7705-7708

(35) Dan, X.; Yang, Q.; Xing, L.; Tang, Y.; Wang, W.; Cai, Y. Heterogeneous Metallaphotocatalytic C(sp2)–C(sp3) Cross-Coupling Reactions with Integrated Bipyridyl-Ni(II)-Carbon Nitride. *Org. Lett.* **2023**, *25*, 4124-4129

(36) Pal, S.; Chowdhury, S.; Rozwadowski, E.; Auffrant, A.; Gosmini, C. Cobalt-Catalyzed Reductive Cross-Coupling Between Benzyl Chlorides and Aryl Halides. *Adv. Synth. Catal.* **2016**, *358*, 2431-2435

(37) Chen, Y.; Wang, X.; He, X.; An, Q.; Zuo, Z. Photocatalytic dehydroxymethylative arylation by synergistic cerium and nickel catalysis. *J. Am. Chem. Soc.* **2021**, *143*, 4896-4902

(38) Tobisu, M.; Yasutome, A.; Kinuta, H.; Nakamura, K.; Chatani, N. 1,3-Dicyclohexylimidazol-2-ylidene as a Superior Ligand for the Nickel-Catalyzed Cross-Couplings of Aryl and Benzyl Methyl Ethers with Organoboron Reagents. *Org. Lett.* **2014**, *16*, 5572-5575

(39) Charboneau, D. J.; Huang, H.; Barth, E. L.; Germe, C. C.; Hazari, N.; Mercado, B. Q.; Uehling, M. R.; Zultanski, S. L. Tunable and Practical Homogeneous Organic Reductants for Cross-Electrophile Coupling. *J. Am. Chem. Soc.* **2021**, *143*, 21024-21036

(40) Guo, P.; Wang, K.; Jin, W.-J.; Xie, H.; Qi, L.; Liu, X.-Y.; Shu, X.-Z. Dynamic Kinetic Cross-Electrophile Arylation of Benzyl Alcohols by Nickel Catalysis. *J. Am. Chem. Soc.* **2021**, *143*, 513-523

(41) Vara, B. A.; Patel, N. R.; Molander, G. A. O-benzyl xanthate esters under Ni/photoredox dual catalysis: selective radical generation and Csp3–Csp2 cross-coupling. *ACS Catal.* **2017**, *7*, 3955-3959

(42) Park, G.; Yi, S. Y.; Jung, J.; Cho, E. J.; You, Y. Mechanism and Applications of the Photoredox Catalytic Coupling of Benzyl Bromides. *Chem. Eur. J.* **2016**, *22*, 17790-17799

(43) Geniller, L.; Taillefer, M.; Jaroschik, F.; Prieto, A. Nickel Metallaphotoredox Catalysis Enabling Desulfurative Cross Coupling Reactions. *Adv. Synth. Catal.* **2022**, *364*, 4249-4254

(44) Wang, T.; Yang, S.; Xu, S.; Han, C.; Guo, G.; Zhao, J. Palladium catalyzed Suzuki cross-coupling of benzyltrimethylammonium salts via C–N bond cleavage. *RSC Adv.* **2017**, *7*, 15805-15808

(45) Day, C. S.; Rentería-Gómez, Á.; Ton, S. J.; Gogoi, A. R.; Gutierrez, O.; Martin, R. Elucidating electron-transfer events in polypyridine nickel complexes for reductive coupling reactions. *Nat. Catal.* **2023**, *6*, 244-253

(46) Yanagi, T.; Somerville, R. J.; Nogi, K.; Martin, R.; Yorimitsu, H. Ni-Catalyzed Carboxylation of C(sp2)–S Bonds with CO2: Evidence for the Multifaceted Role of Zn. *ACS Catal.* **2020**, *10*, 2117-2123

Copies of NMR spectra

^{1}H NMR of 4,4′-Czbpy

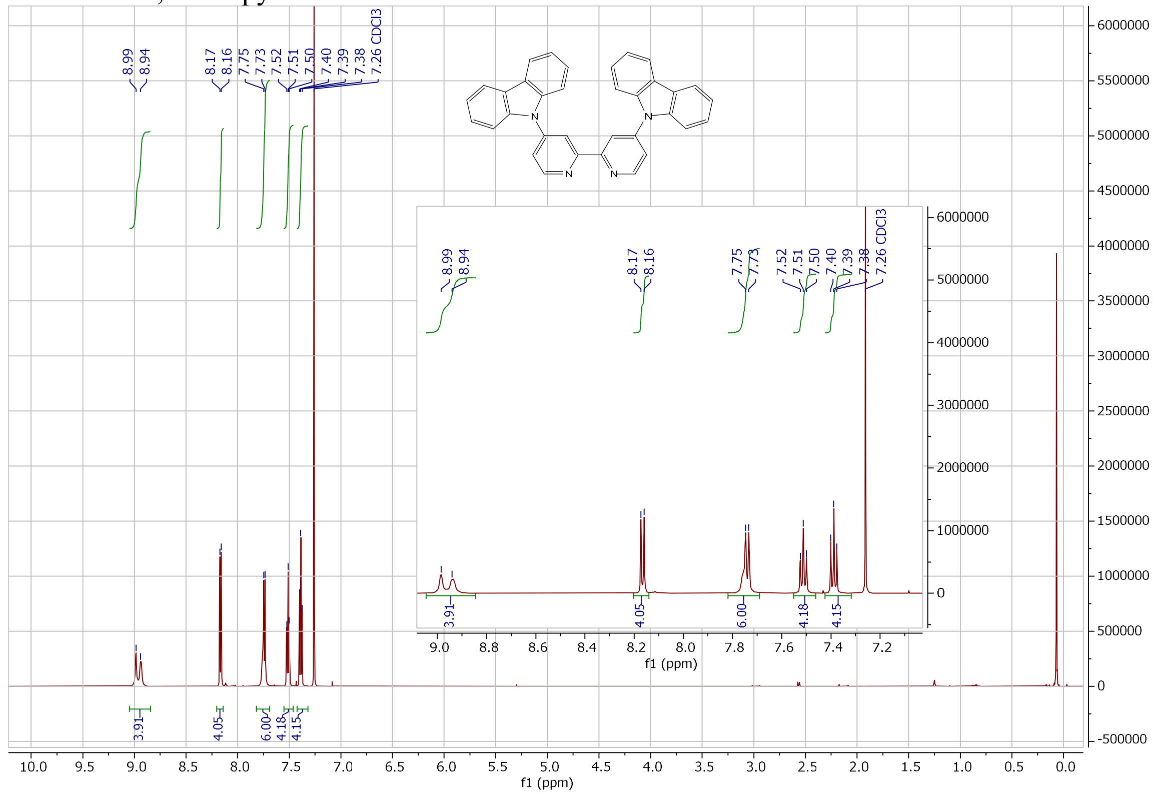

^{13}C NMR of 4,4′-Czbpy

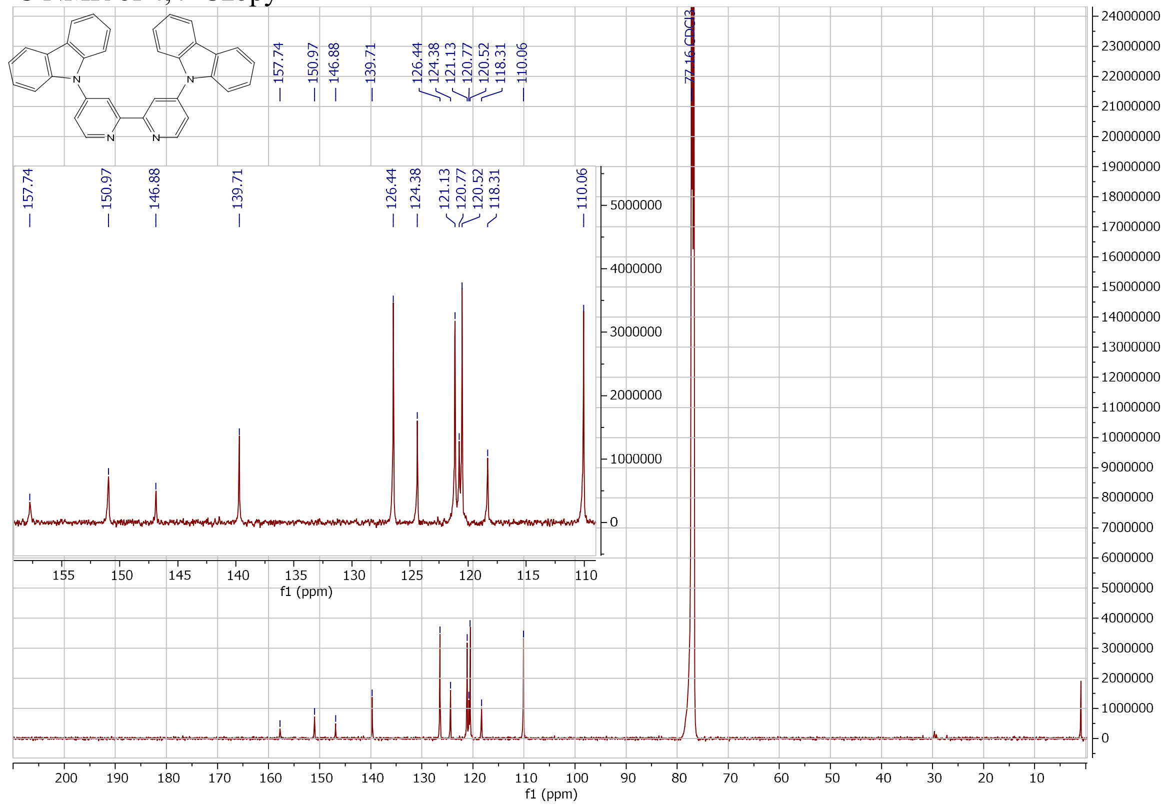

^{1}H NMR of 1-methyl-4-((4-(trifluoromethyl)phenyl)sulfonyl)benzene (**4**)

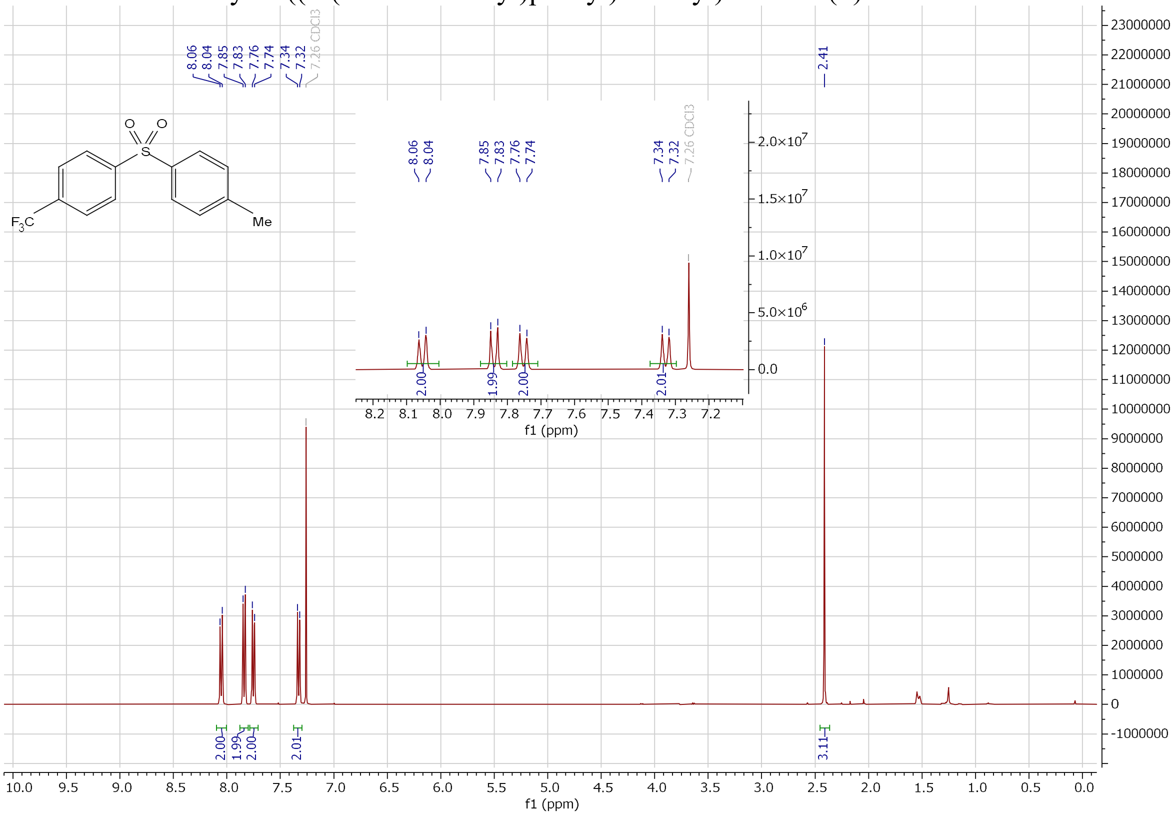

^{13}C NMR of 1-methyl-4-((4-(trifluoromethyl)phenyl)sulfonyl)benzene (**4**)

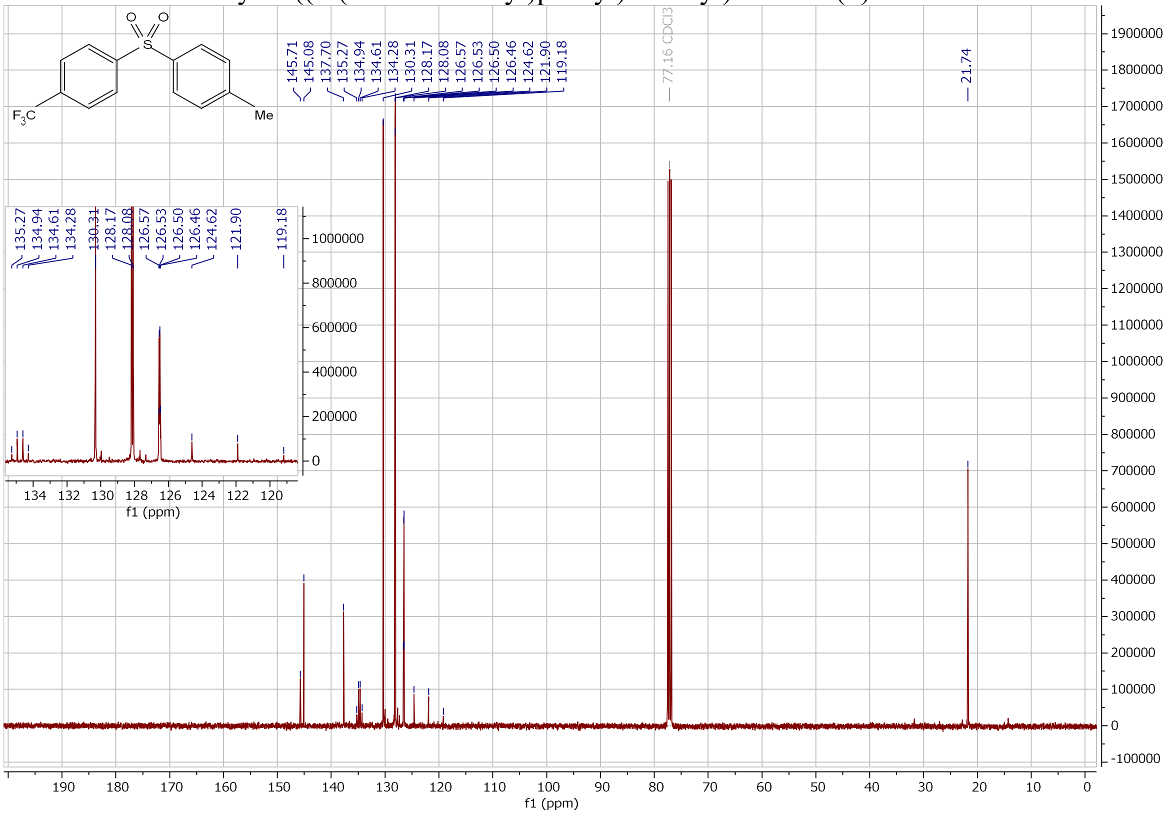

^{19}F NMR of 1-methyl-4-((4-(trifluoromethyl)phenyl)sulfonyl)benzene (**4**)

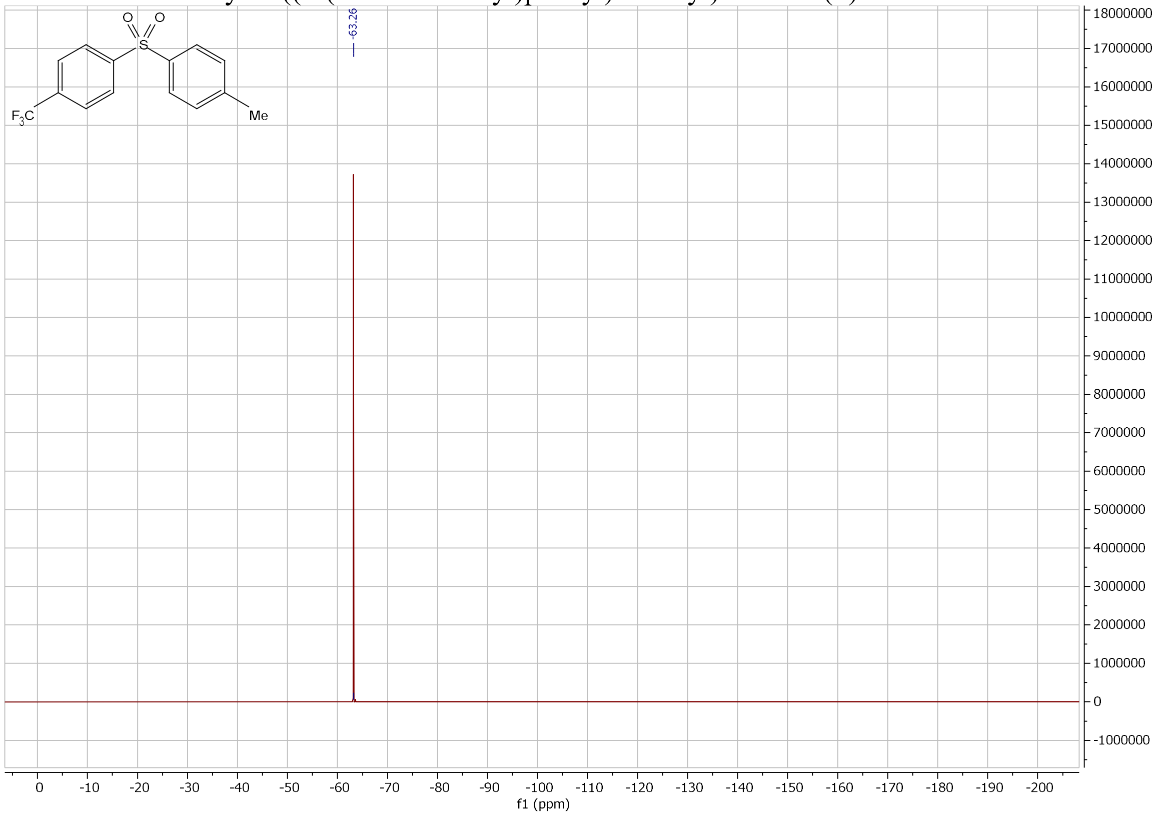

^{1}H NMR of 1-benzyl-4-(trifluoromethyl)benzene (**7**)

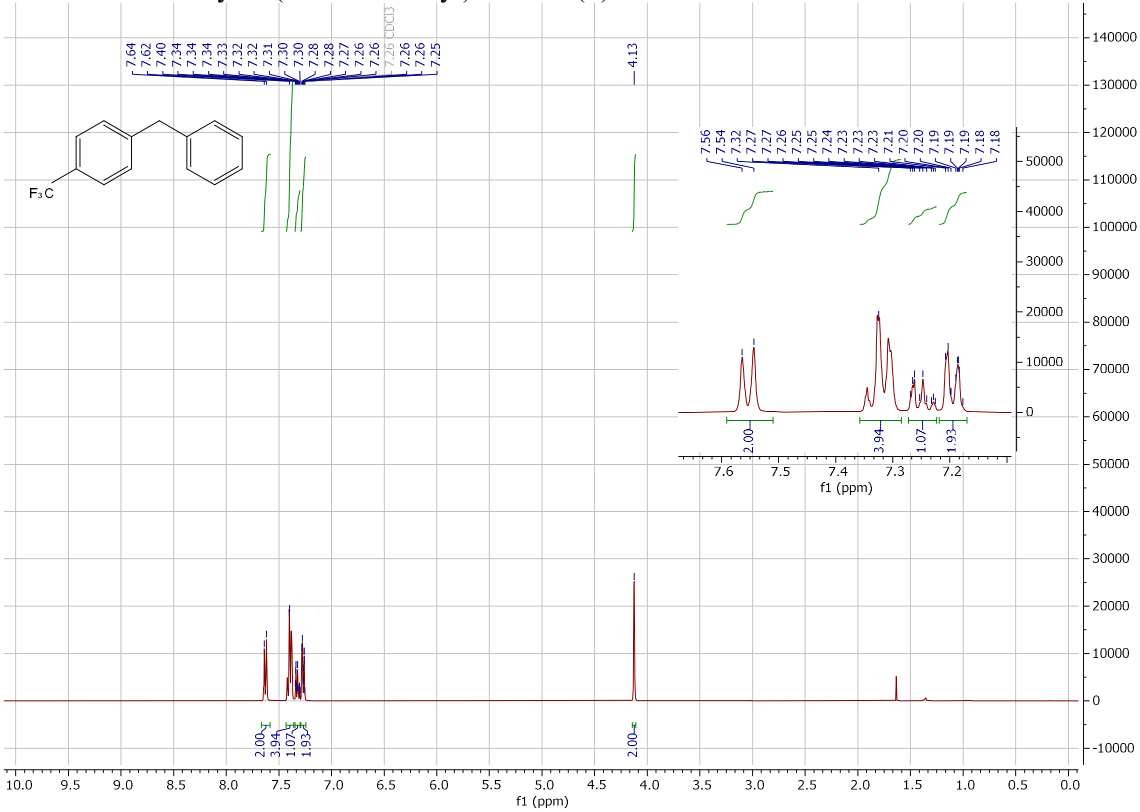

^{13}C NMR of 1-benzyl-4-(trifluoromethyl)benzene (**7**)

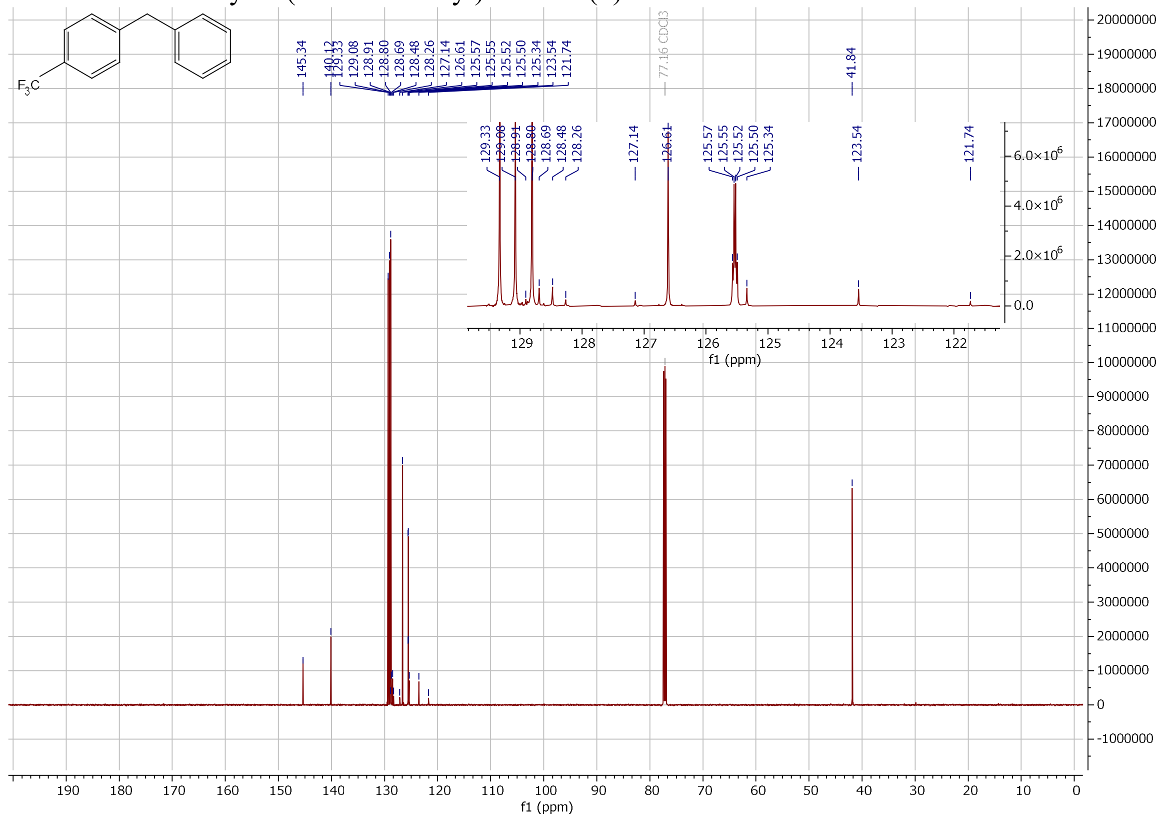

^{19}F NMR of 1-benzyl-4-(trifluoromethyl)benzene (**7**)

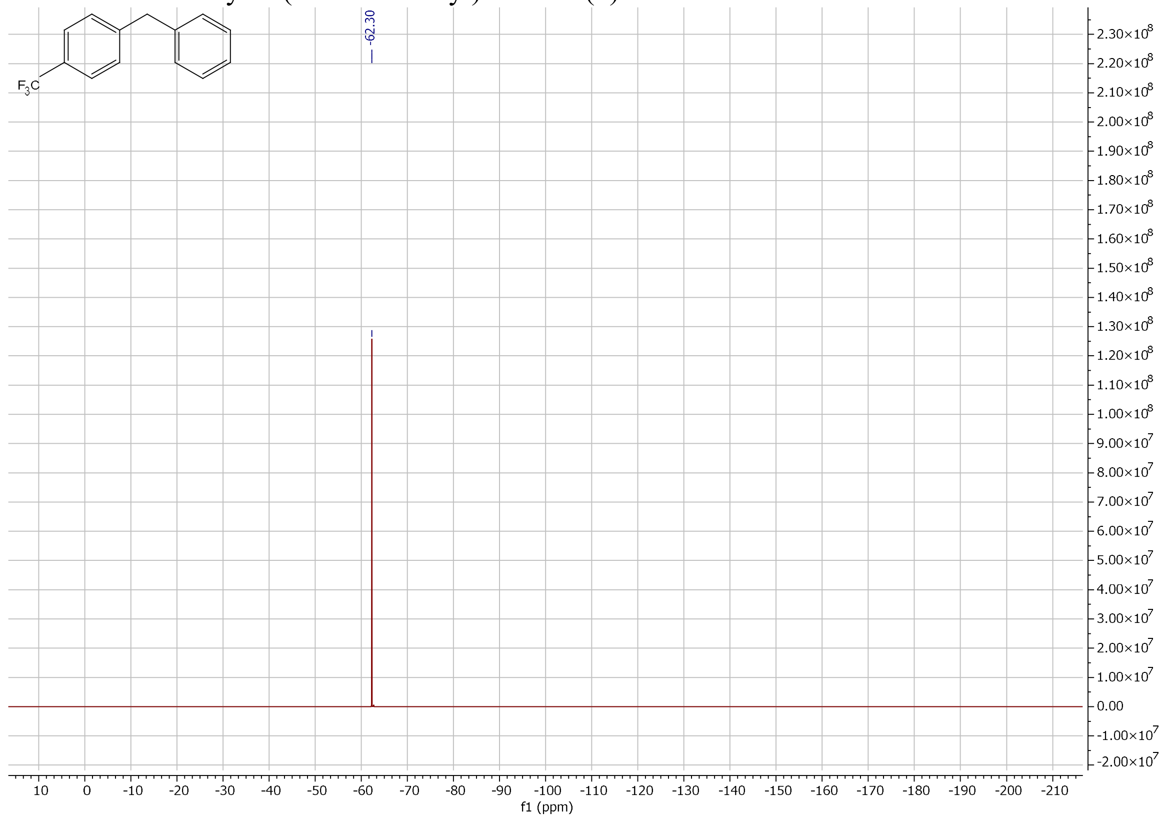

^{1}H NMR of 4-benzylbenzonitrile (**9**)

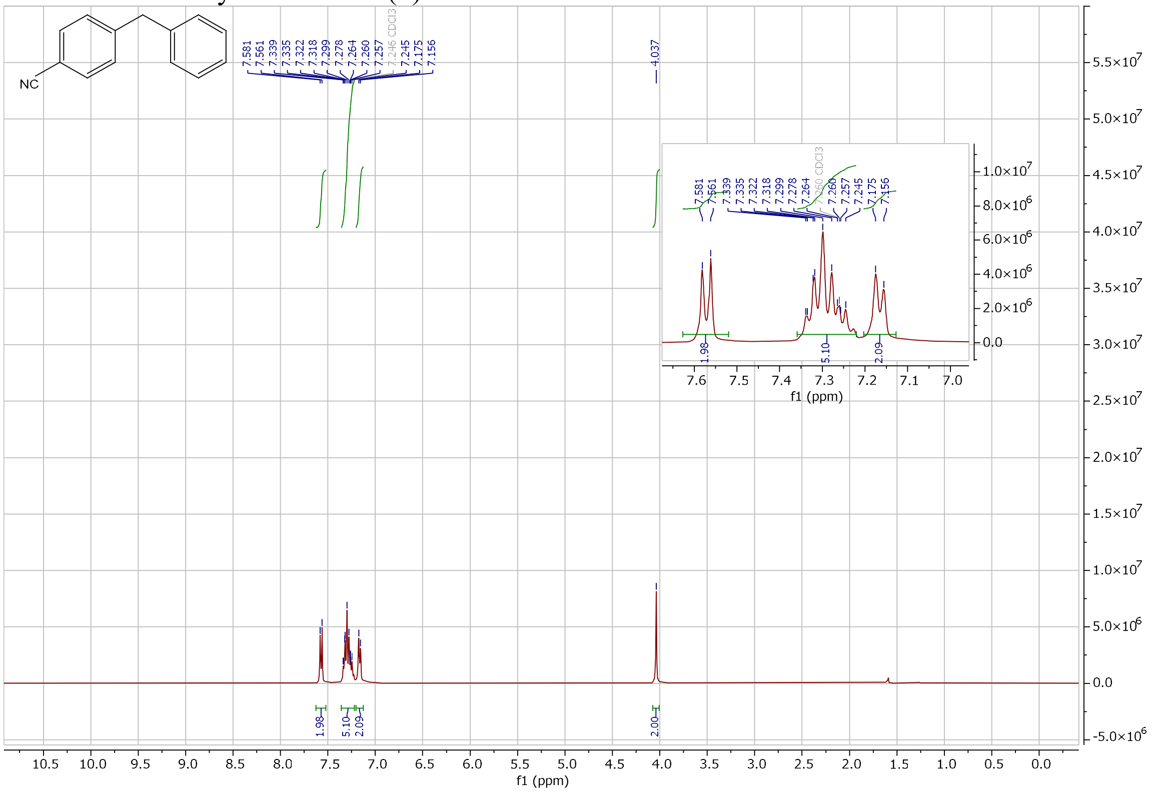

^{13}C NMR of 4-benzylbenzonitrile (**9**)

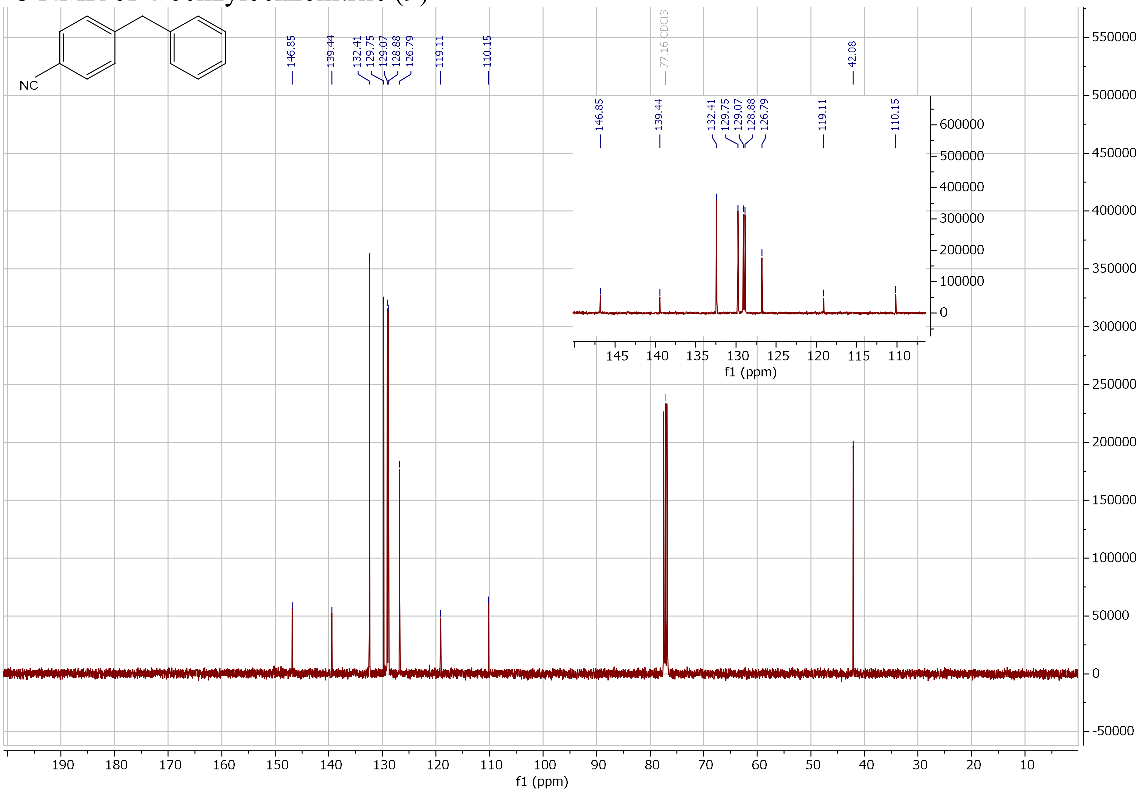

^{1}H NMR of 3-benzylbenzonitrile (**10**)

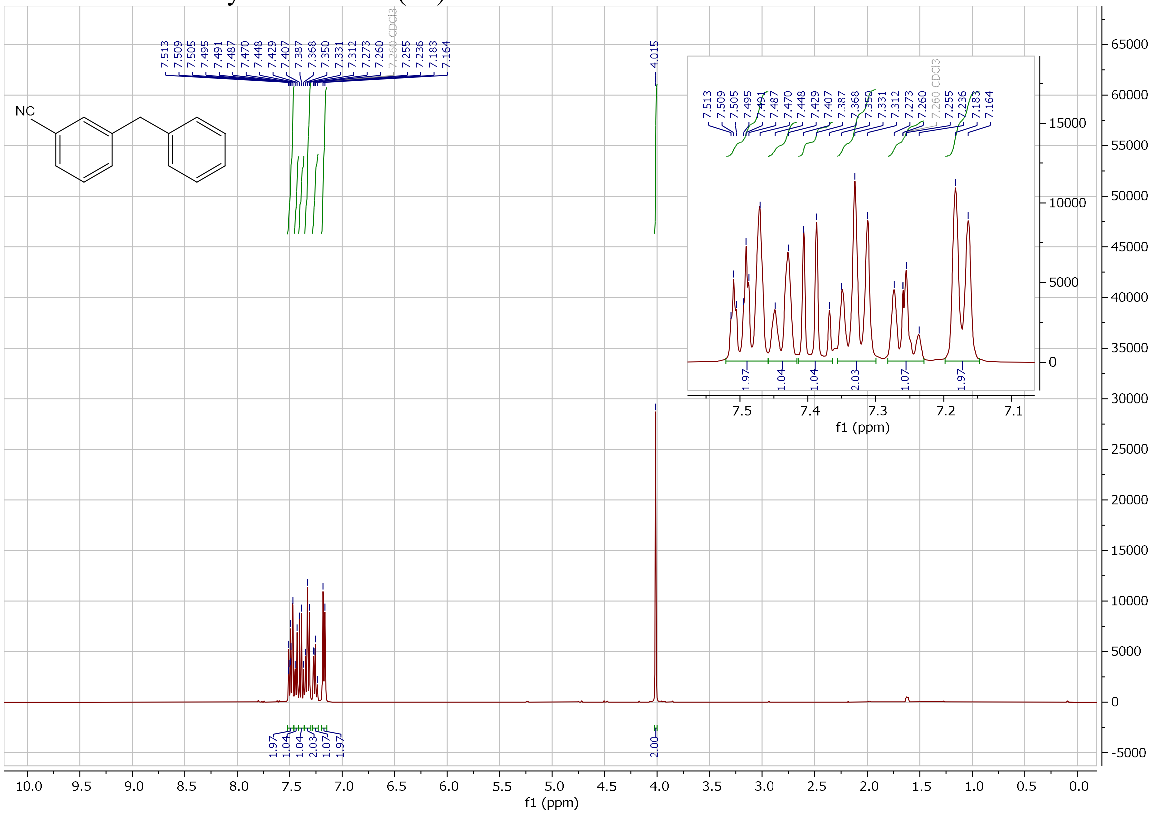

^{13}C NMR of 3-benzylbenzonitrile (**10**)

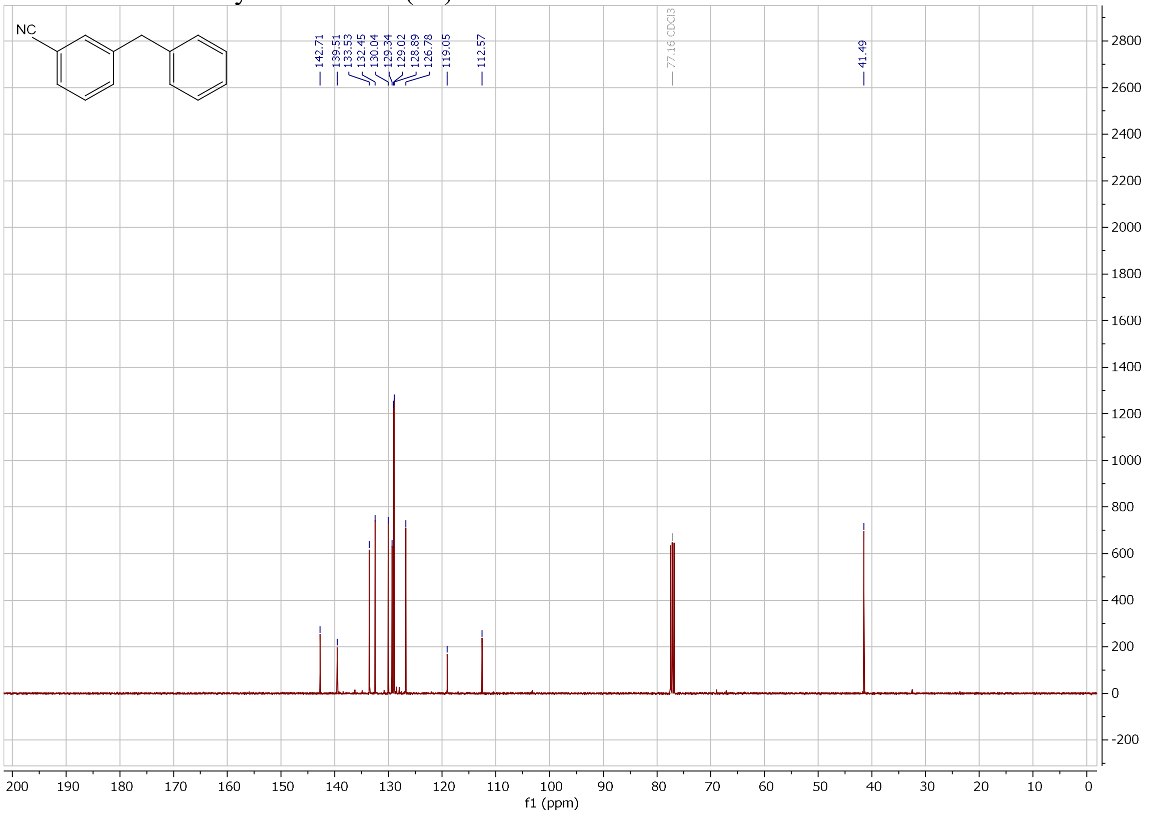

^{1}H NMR of 2-benzylbenzonitrile (**11**)

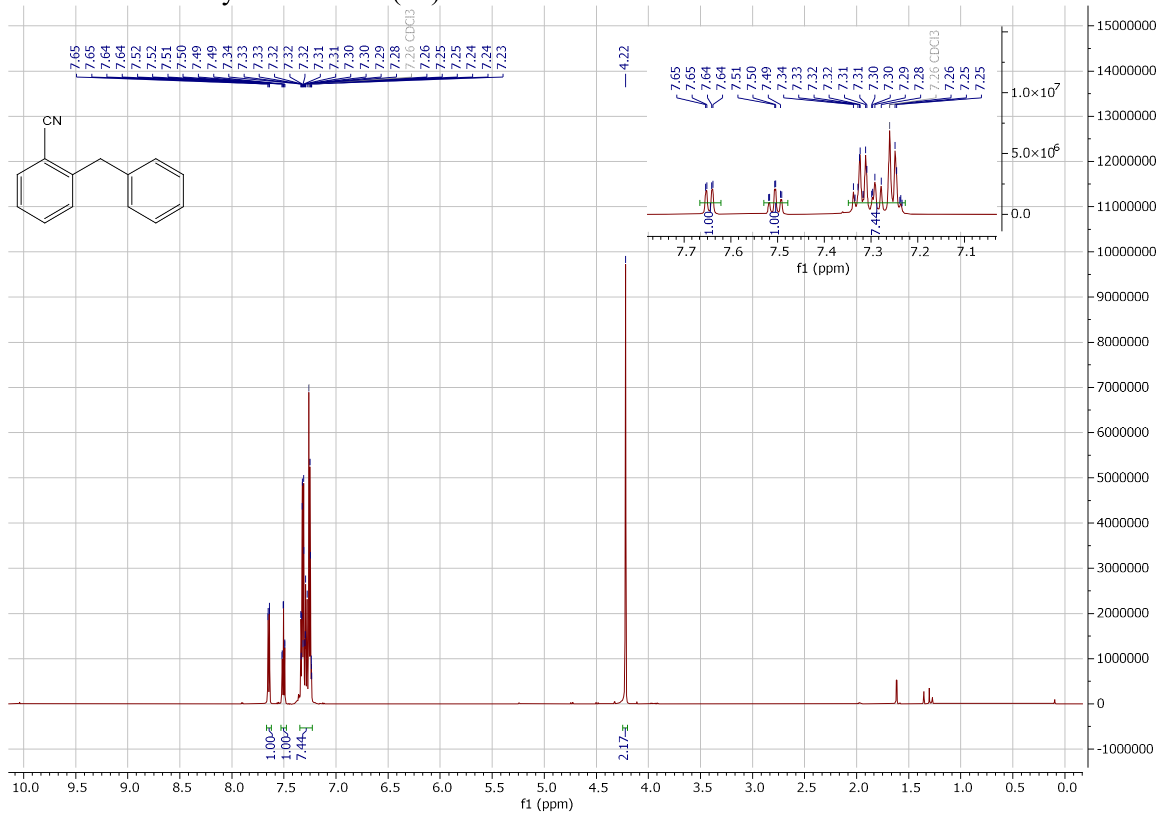

^{13}C NMR of 2-benzylbenzonitrile (**11**)

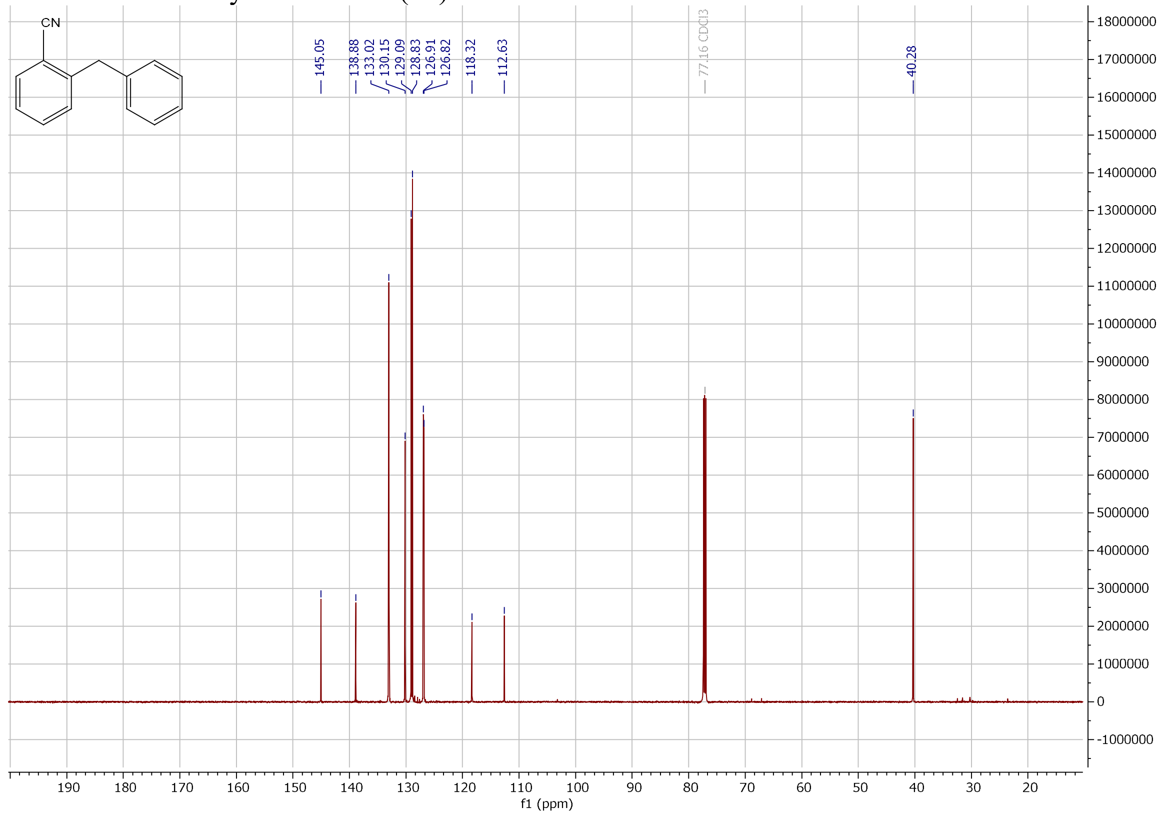

^{1}H NMR of 1-benzyl-4-(methylsulfonyl)benzene (**12**)

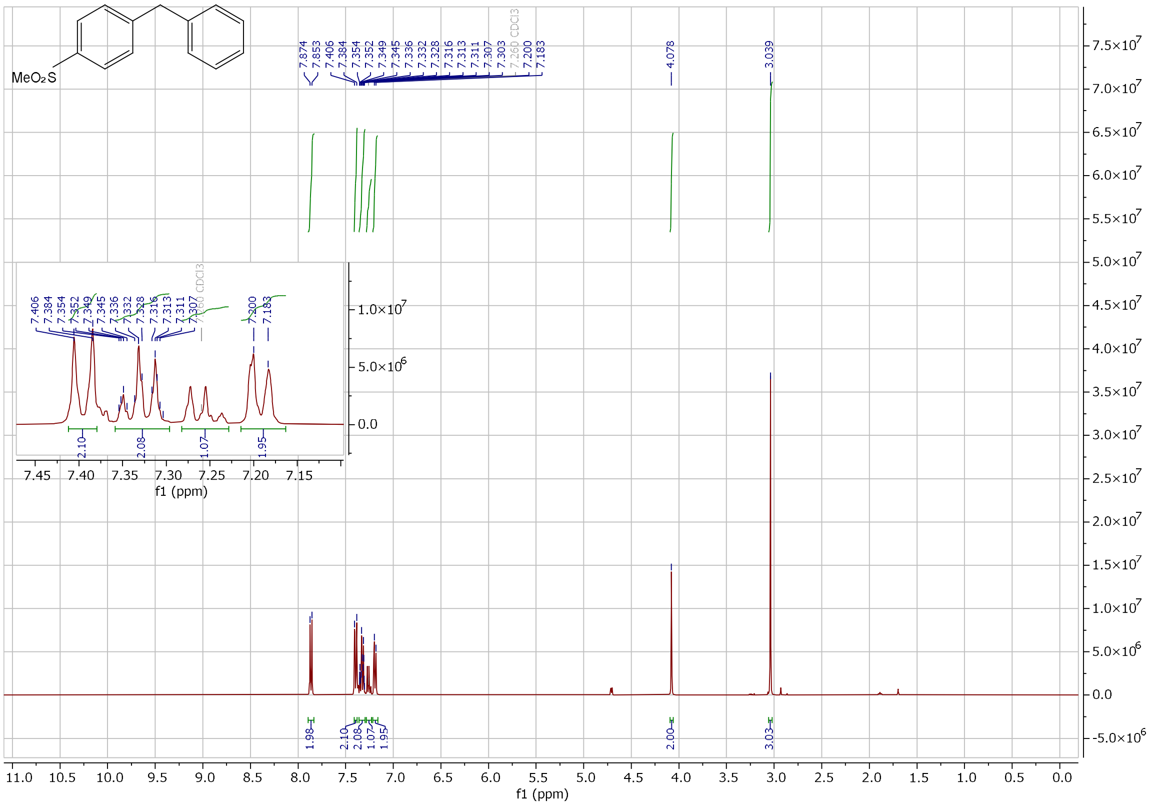

^{13}C NMR of 1-benzyl-4-(methylsulfonyl)benzene (**12**)

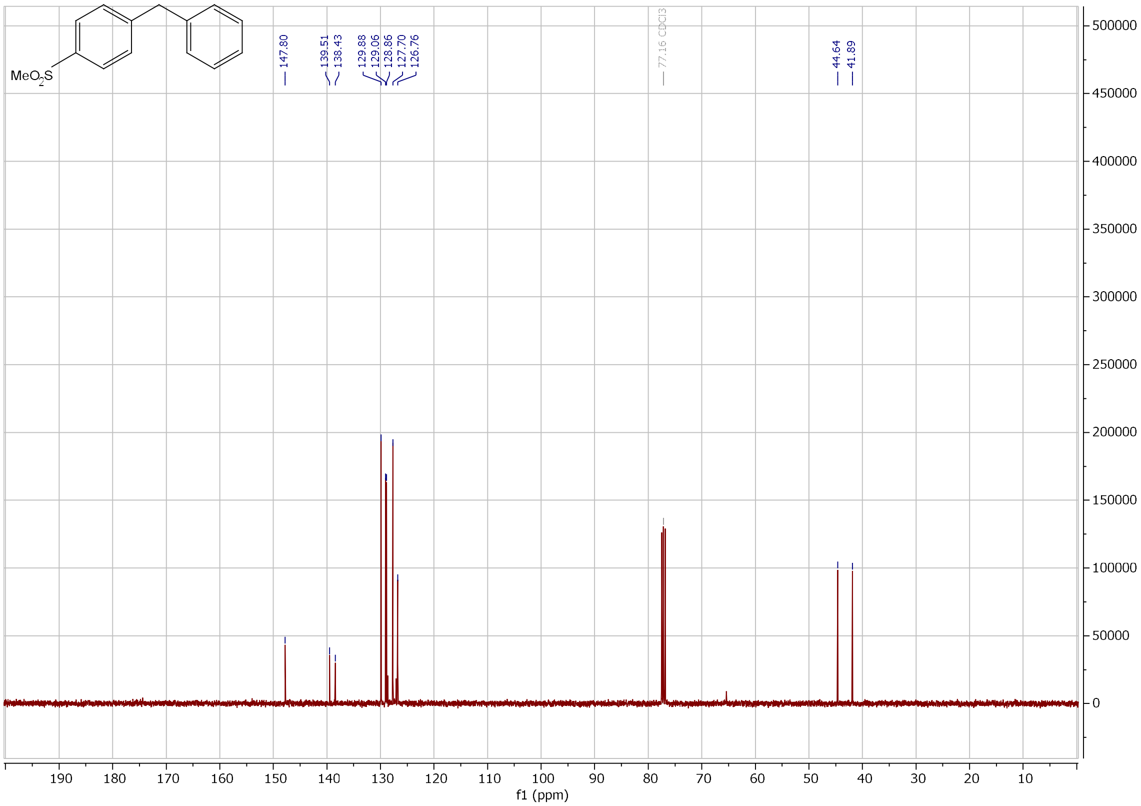

^{1}H NMR of methyl 3-benzylbenzoate (**13**)

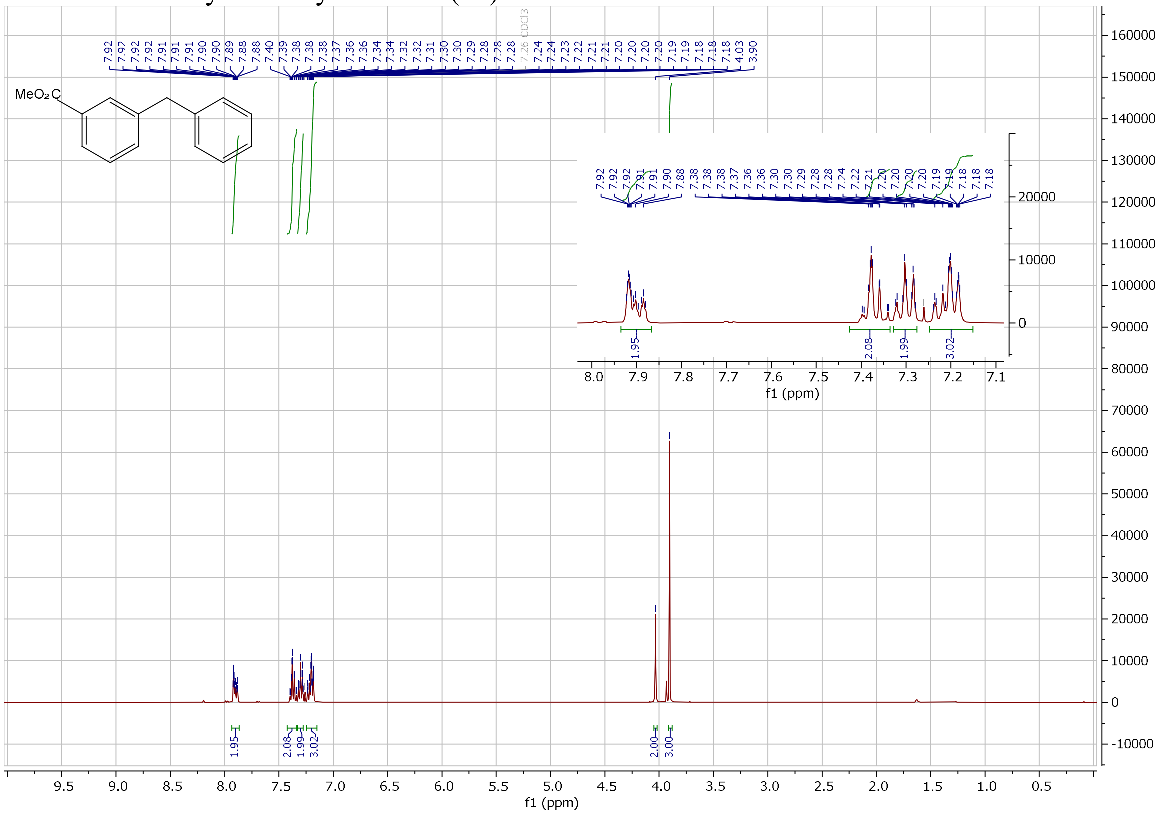

^{13}C NMR of methyl 3-benzylbenzoate (**13**)

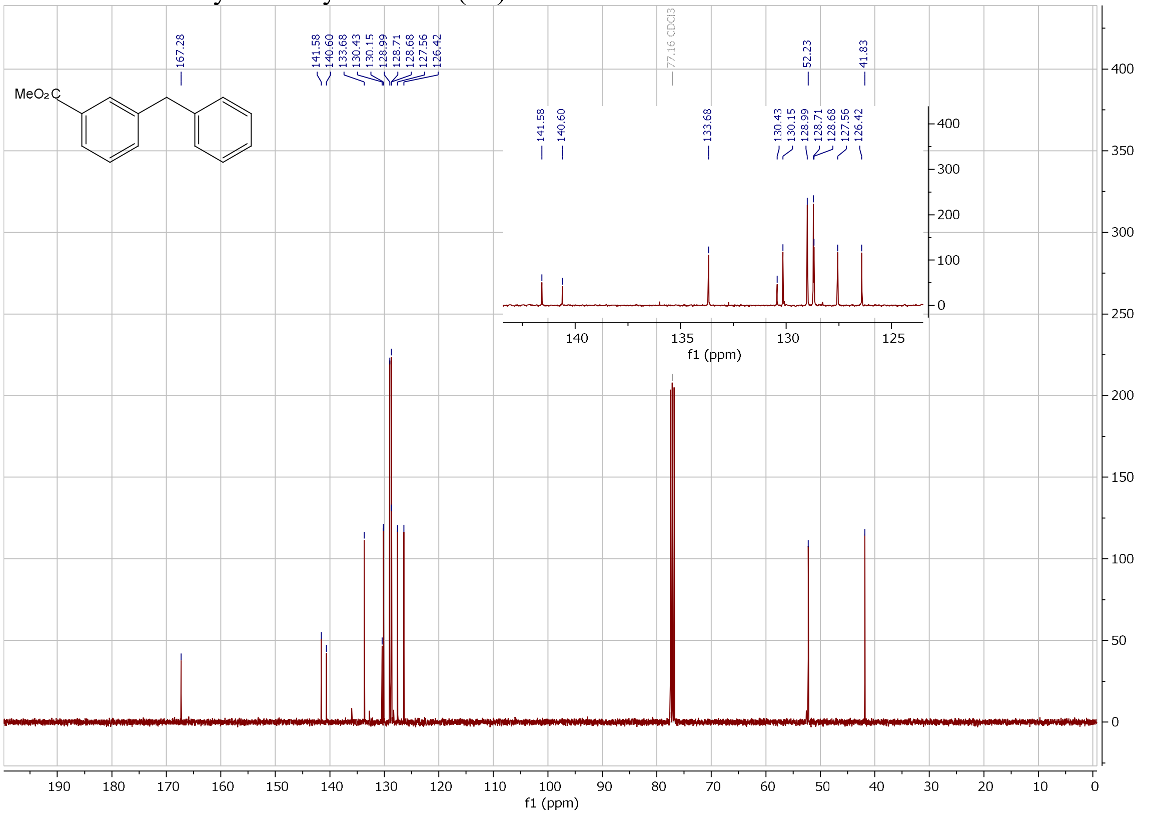

^{1}H NMR of 1-benzyl-2,3-dichlorobenzene (**14**)

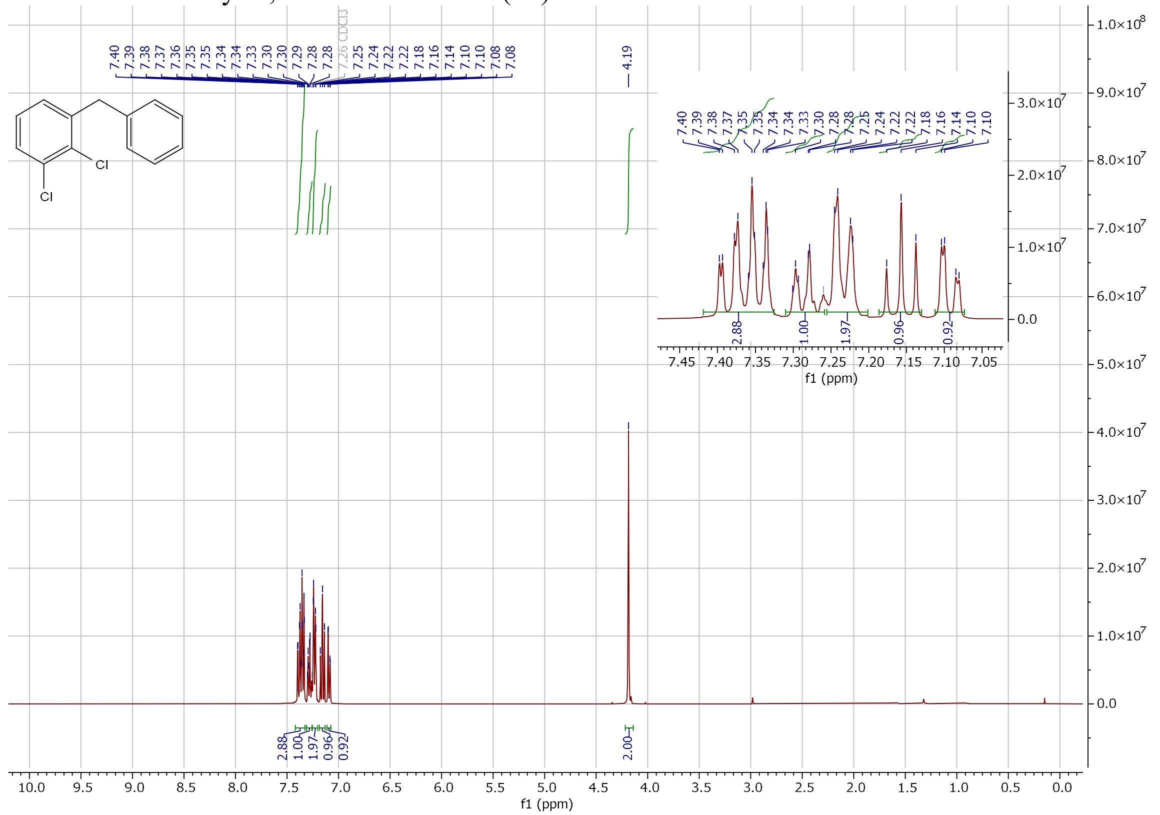

^{13}C NMR of 1-benzyl-2,3-dichlorobenzene (**14**)

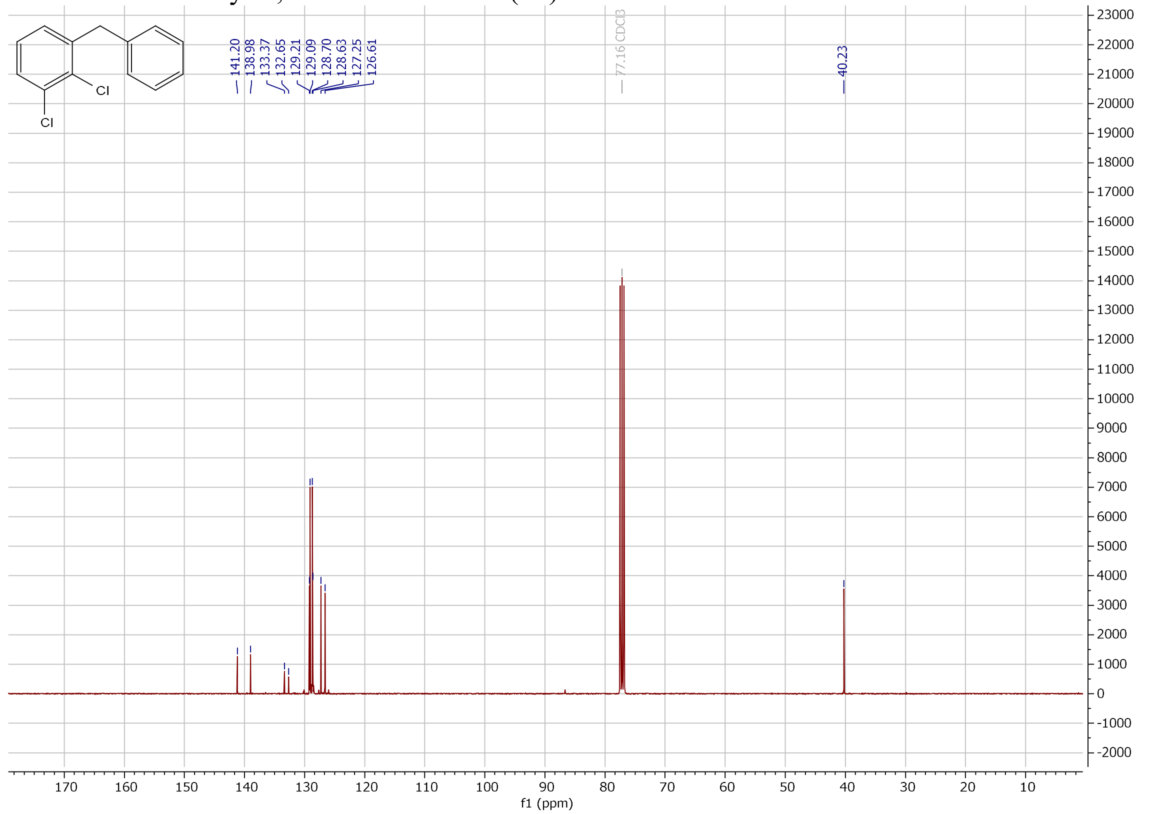

^{1}H NMR of 4-benzylbenzaldehyde (**15**)

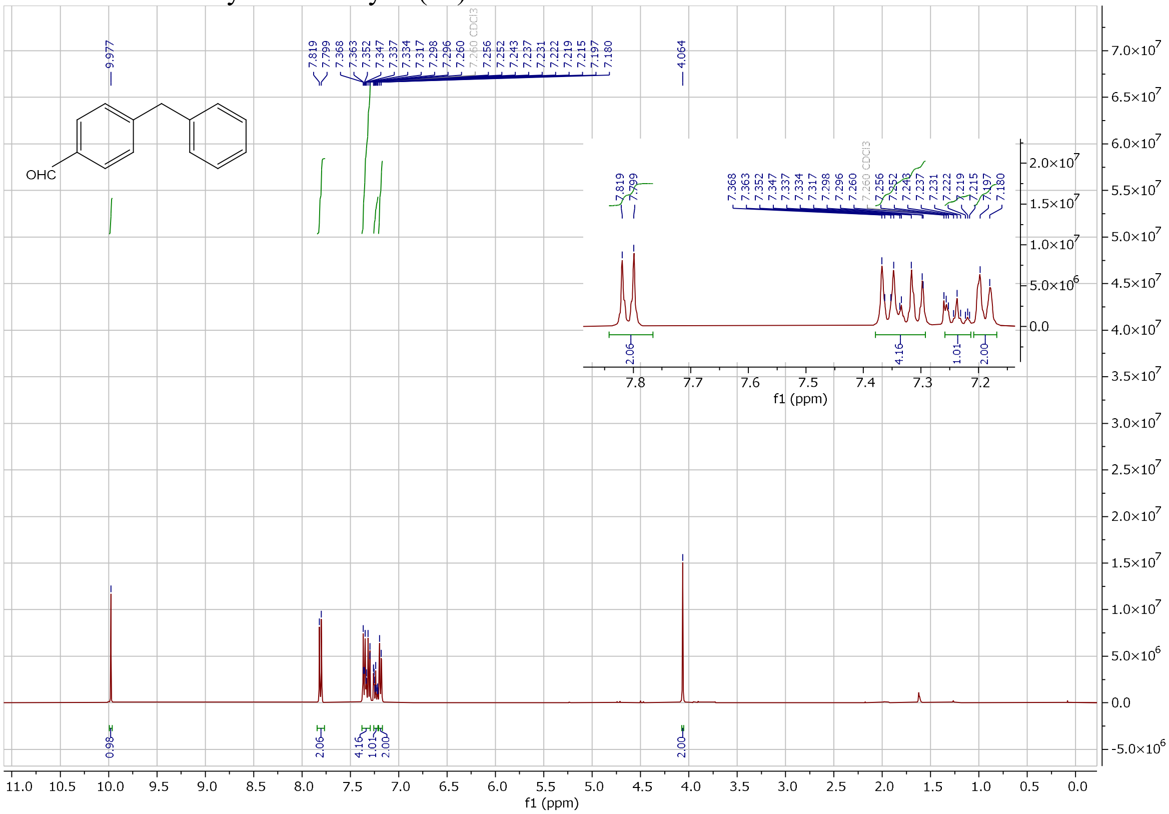

^{13}C NMR of 4-benzylbenzaldehyde (**15**)

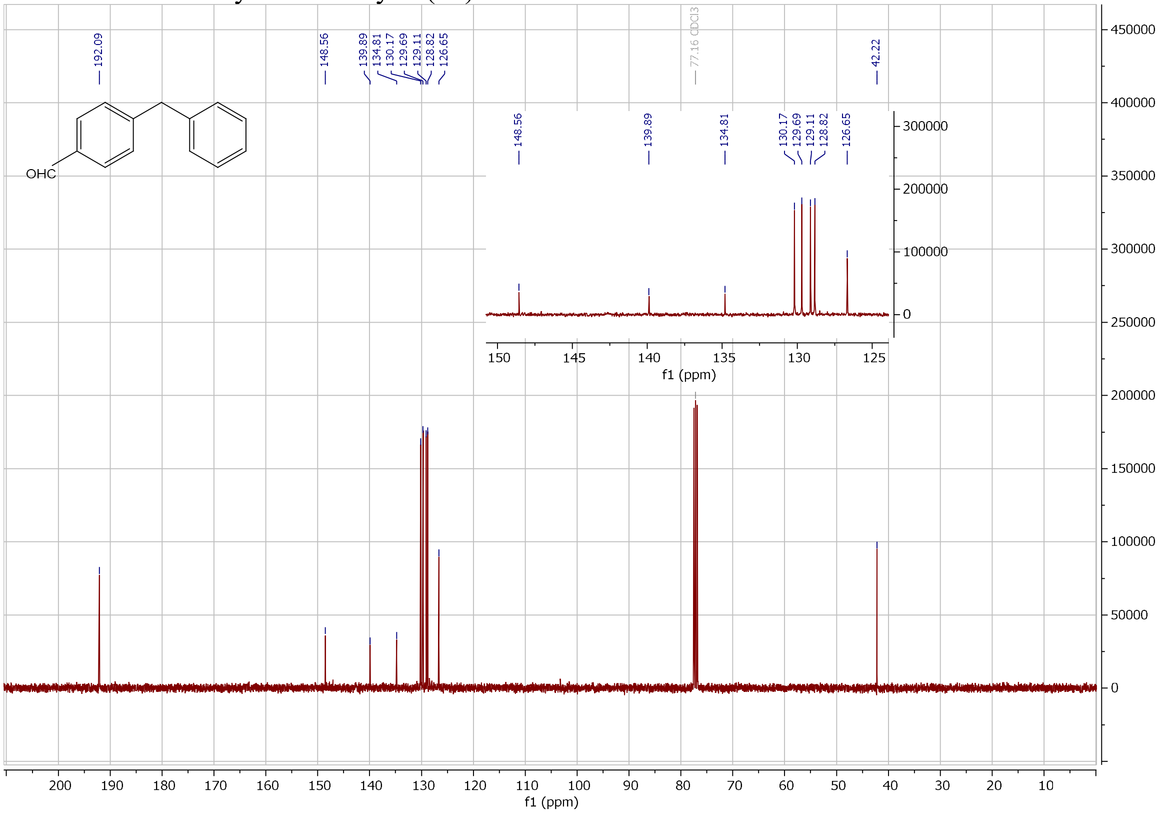

^{1}H NMR of 4-benzylbenzamide (**16**)

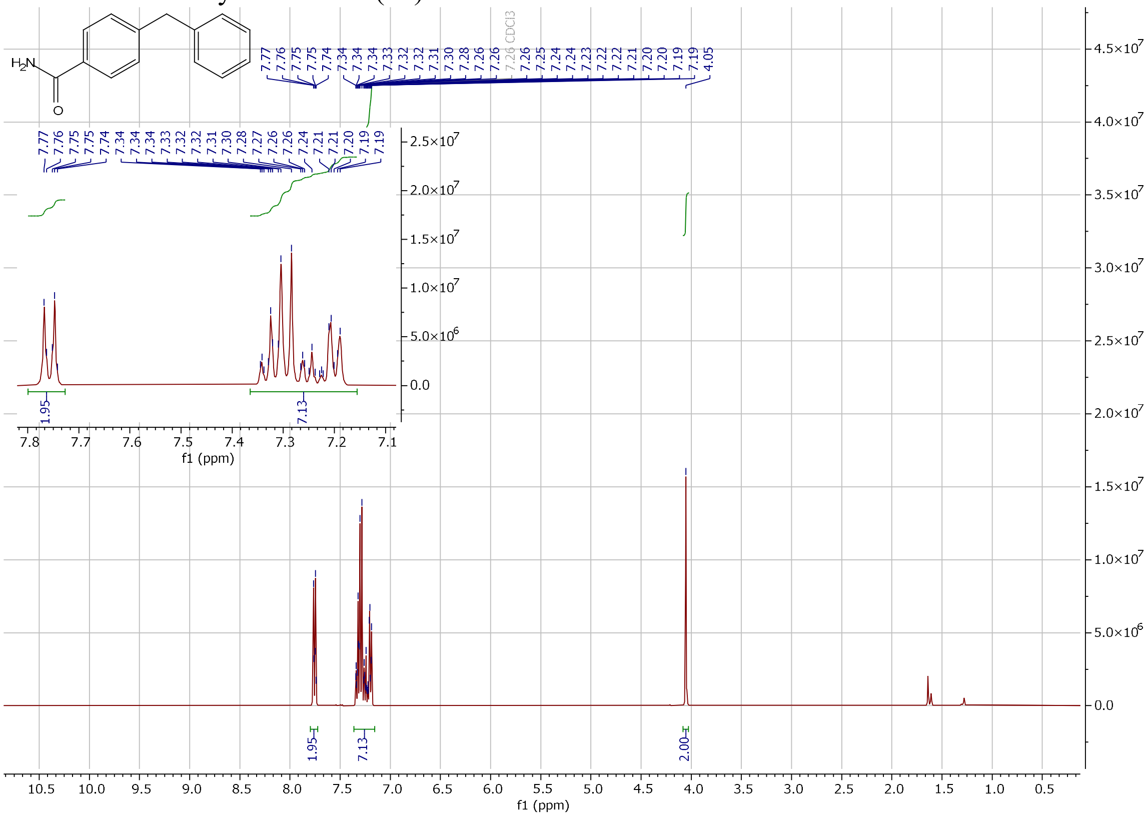

^{13}C NMR of 4-benzylbenzamide (**16**)

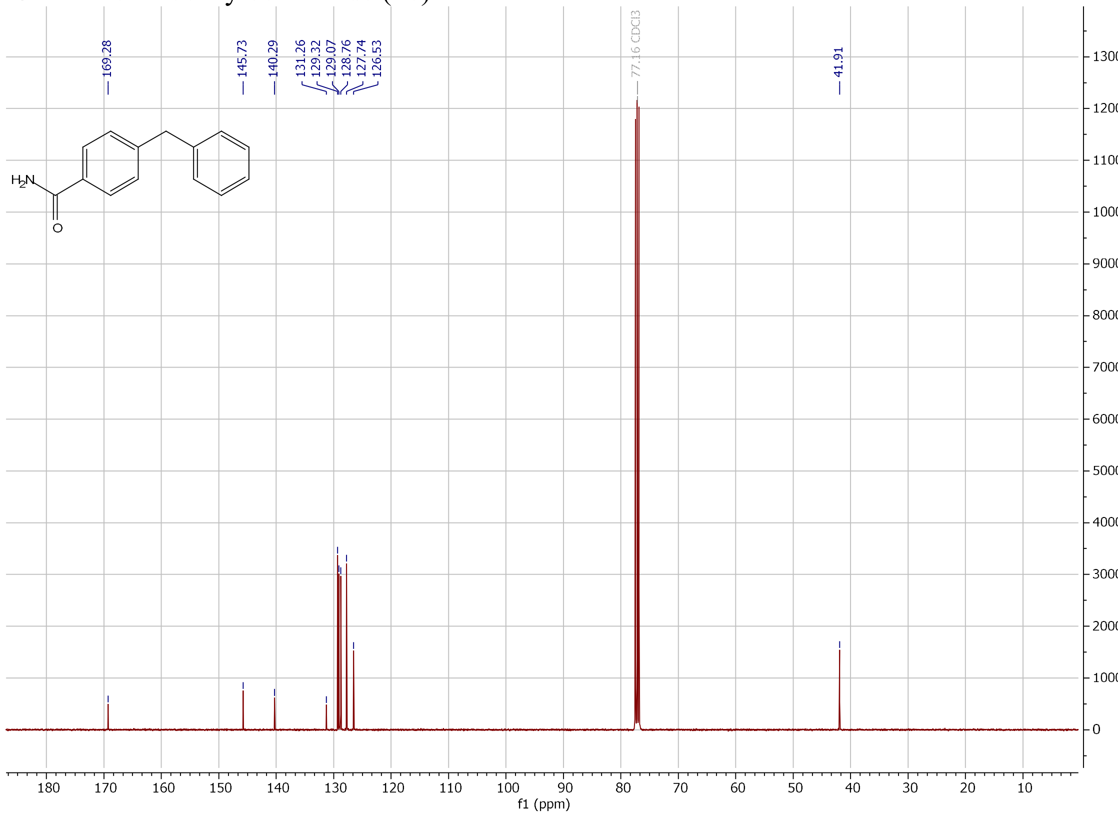

^{1}H NMR of 1-benzyl-3-(trifluoromethoxy)benzene (**17**)

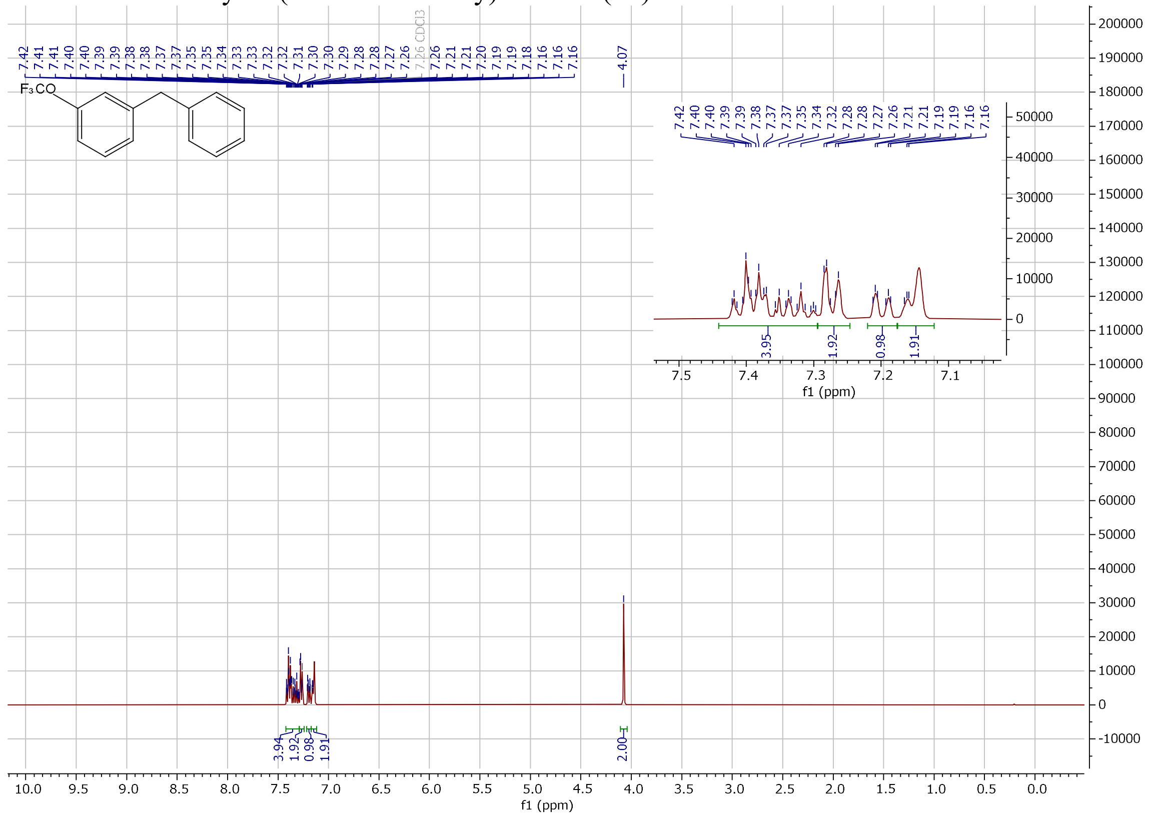

^{13}C NMR of 1-benzyl-3-(trifluoromethoxy)benzene (**17**)

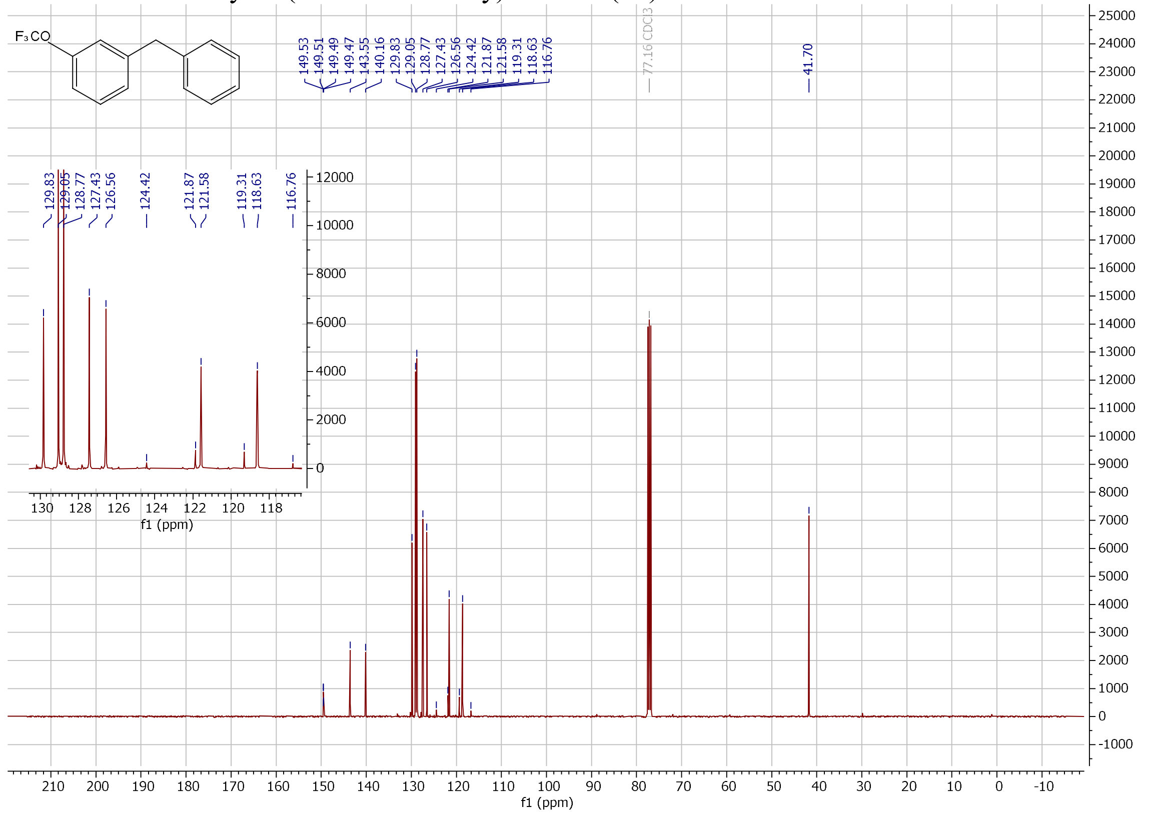

^{19}F NMR of 1-benzyl-3-(trifluoromethoxy)benzene (**17**)

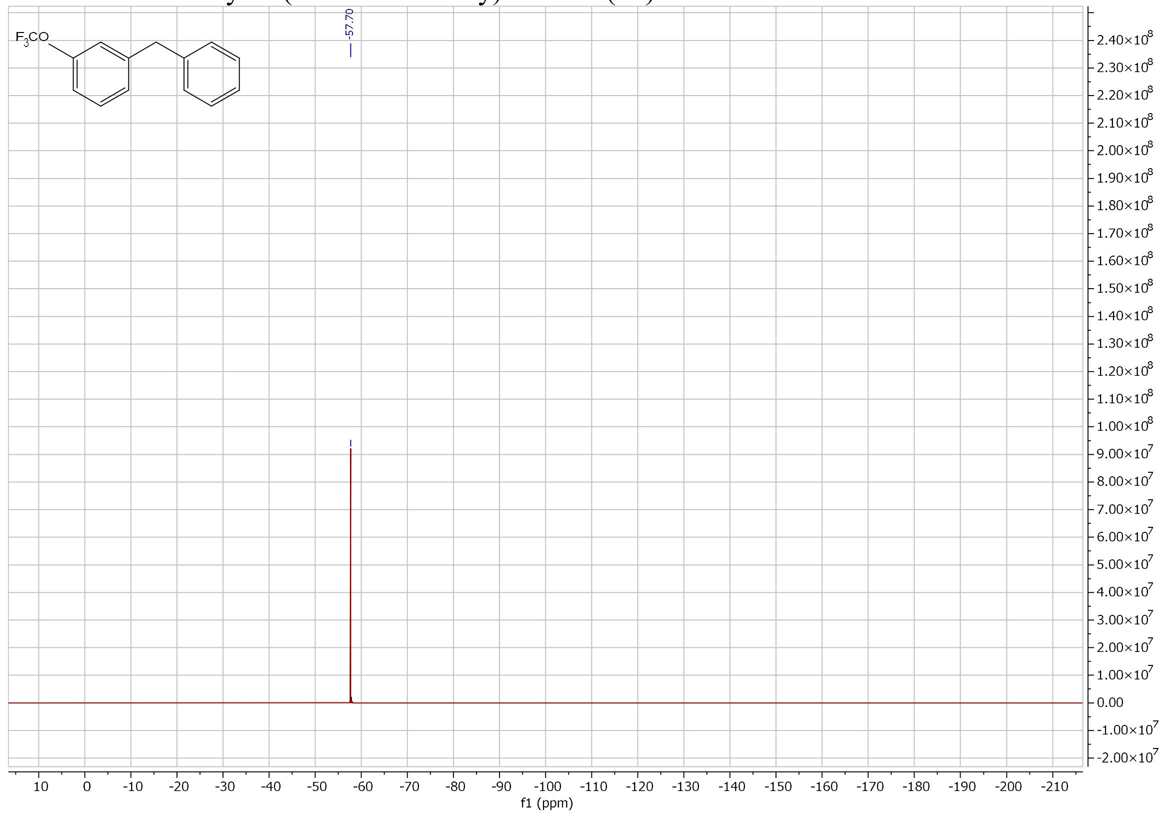

^{1}H NMR of (4-benzylphenyl)(phenyl)methanone (**18**)

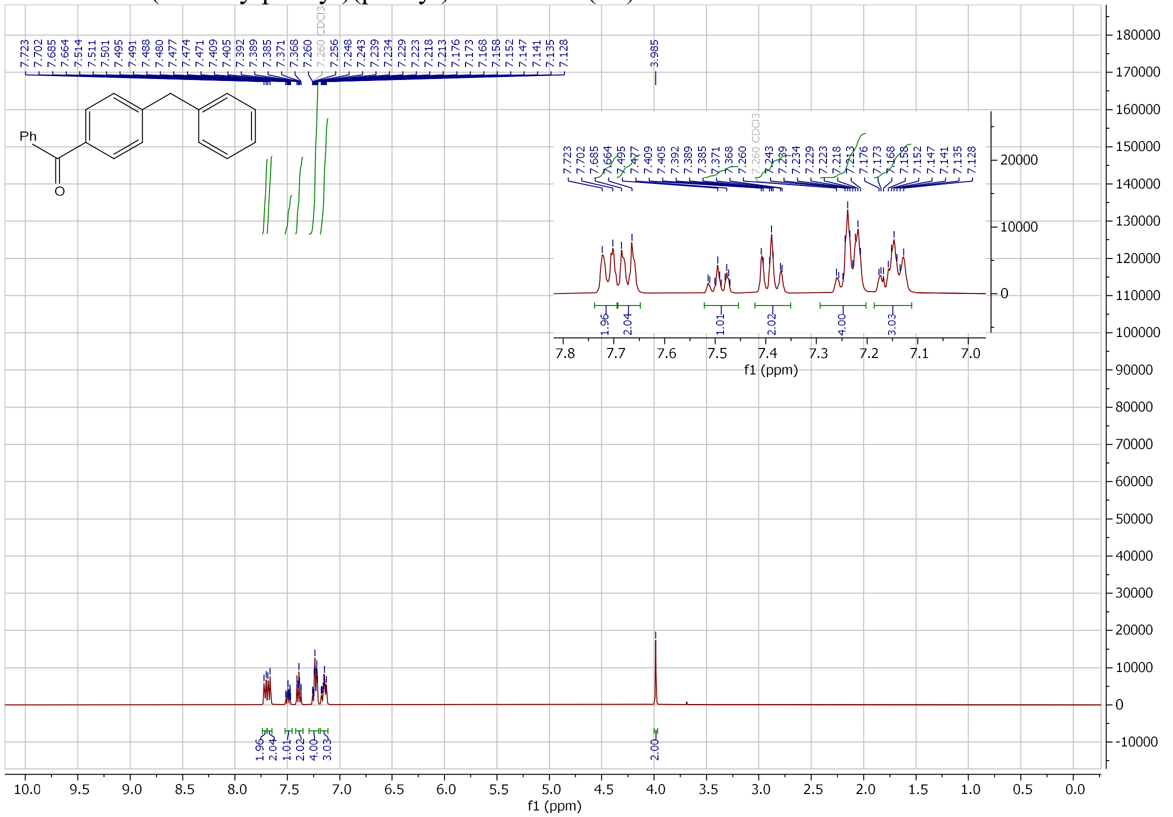

^{13}C NMR of (4-benzylphenyl)(phenyl)methanone (**18**)

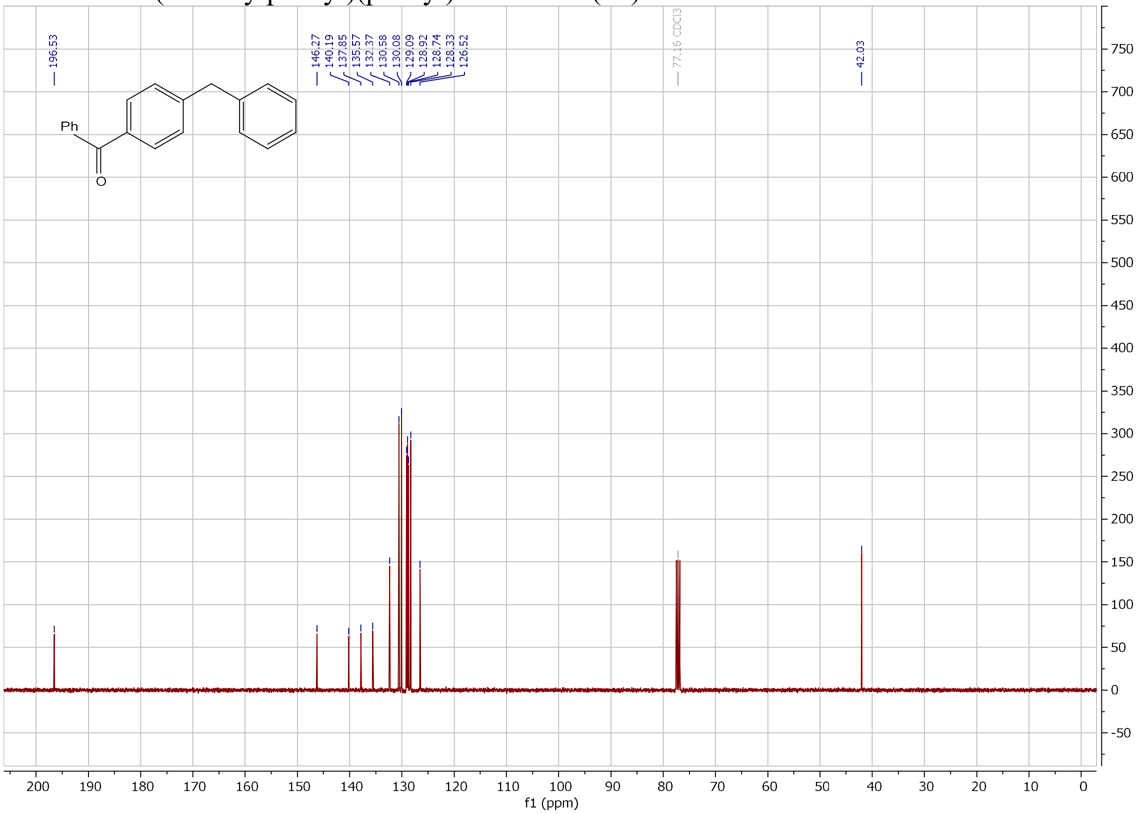

^{1}H NMR of diphenylmethane (**19**)

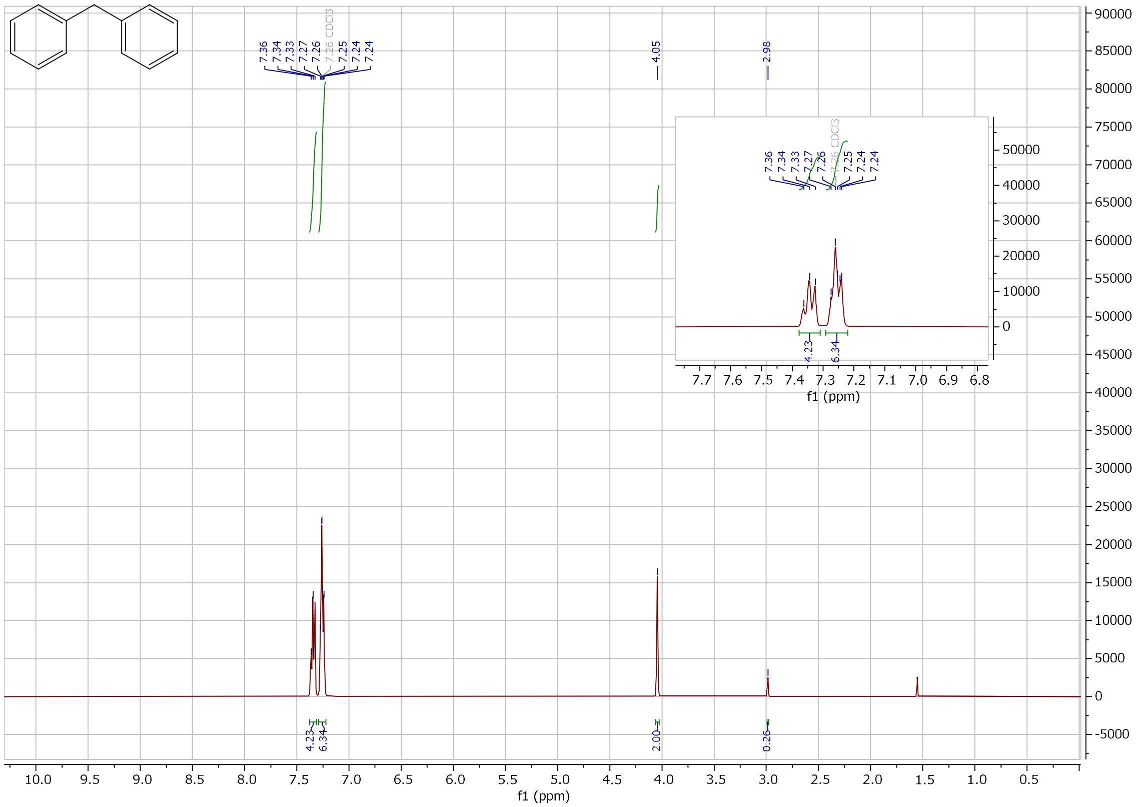

^{13}C NMR of diphenylmethane (**19**)

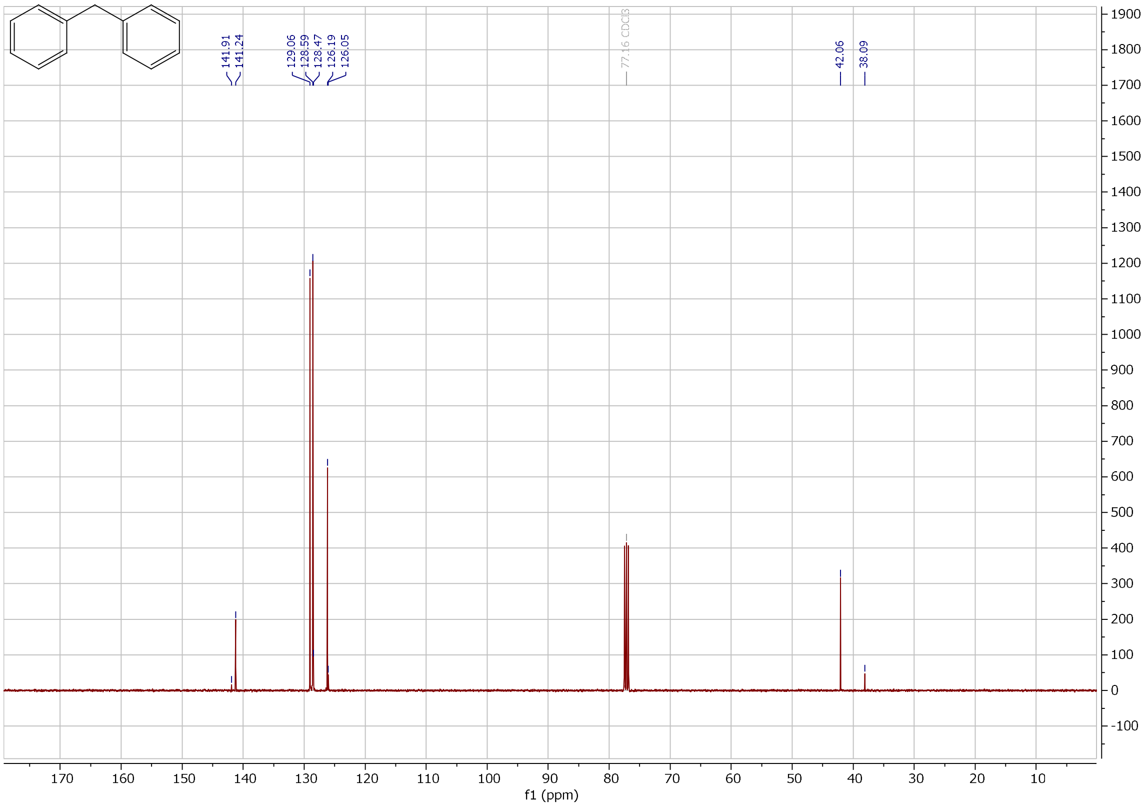

^{1}H NMR of 1-benzyl-fluorobenzene (**20**)

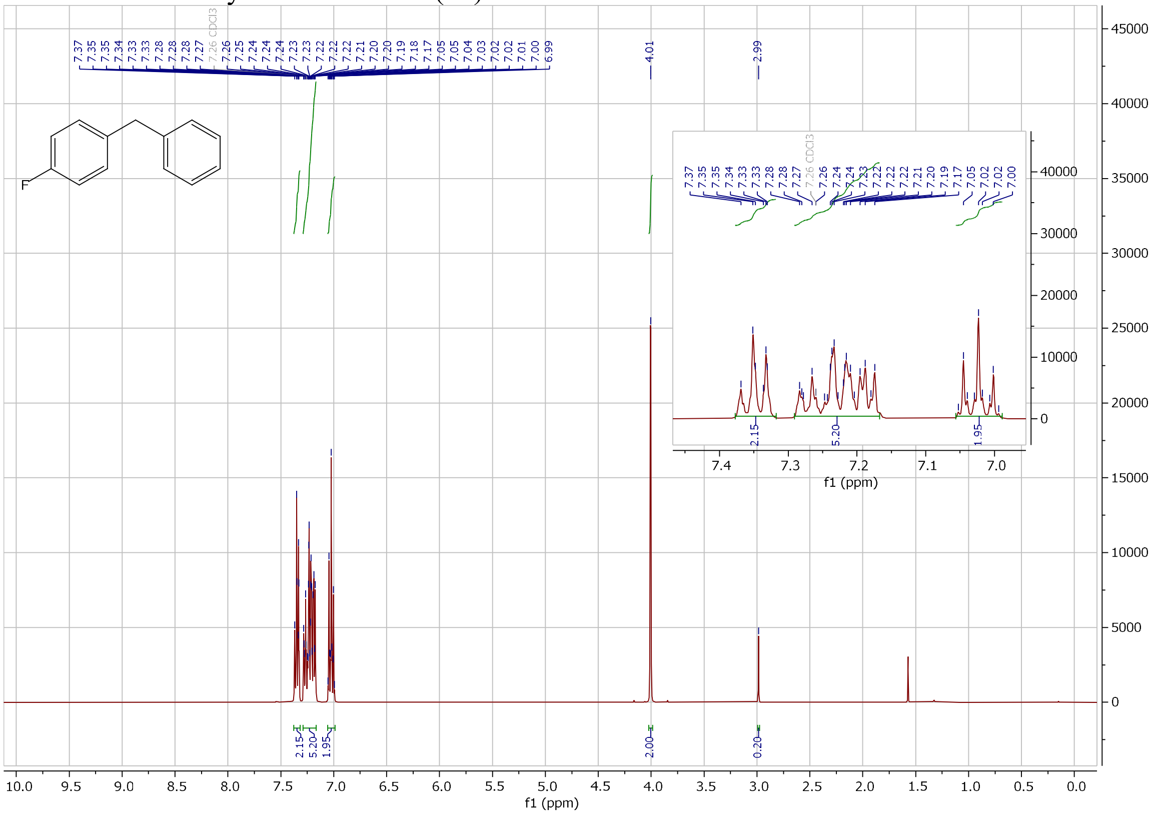

^{13}C NMR of 1-benzyl-fluorobenzene (**20**)

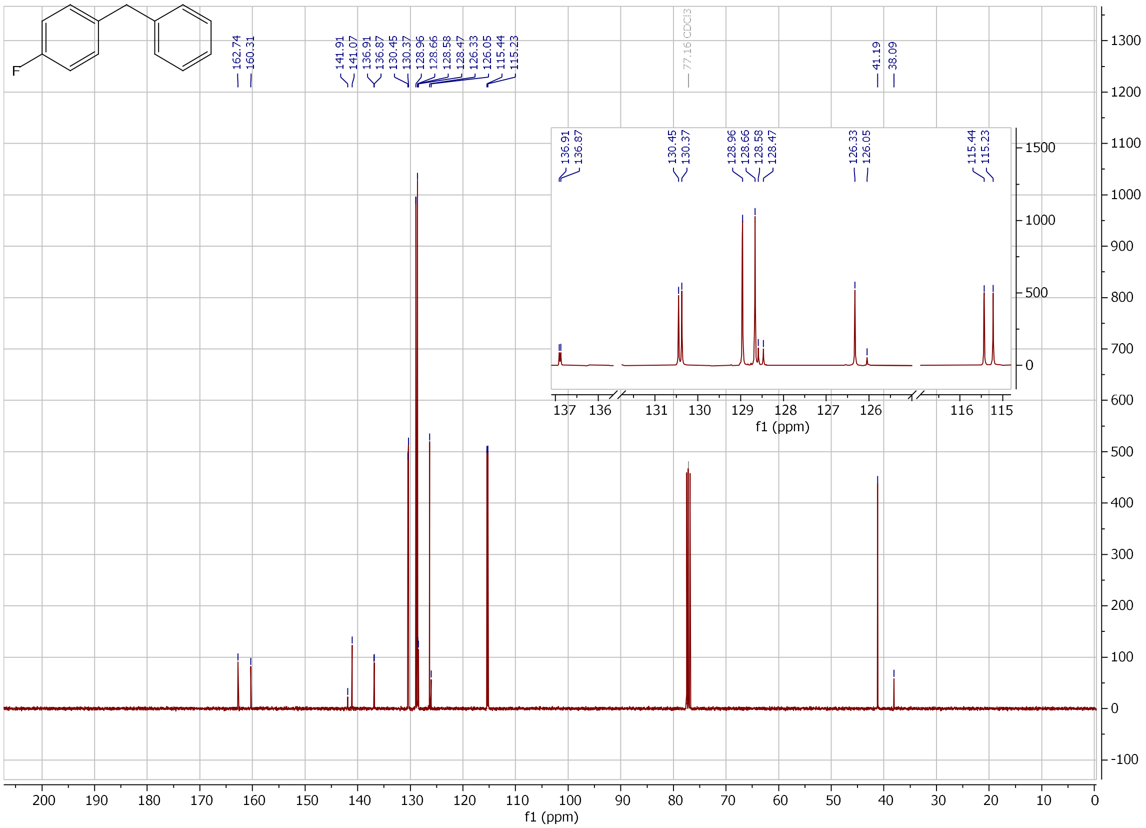

^{19}F NMR of 1-benzyl-fluorobenzene (**20**)

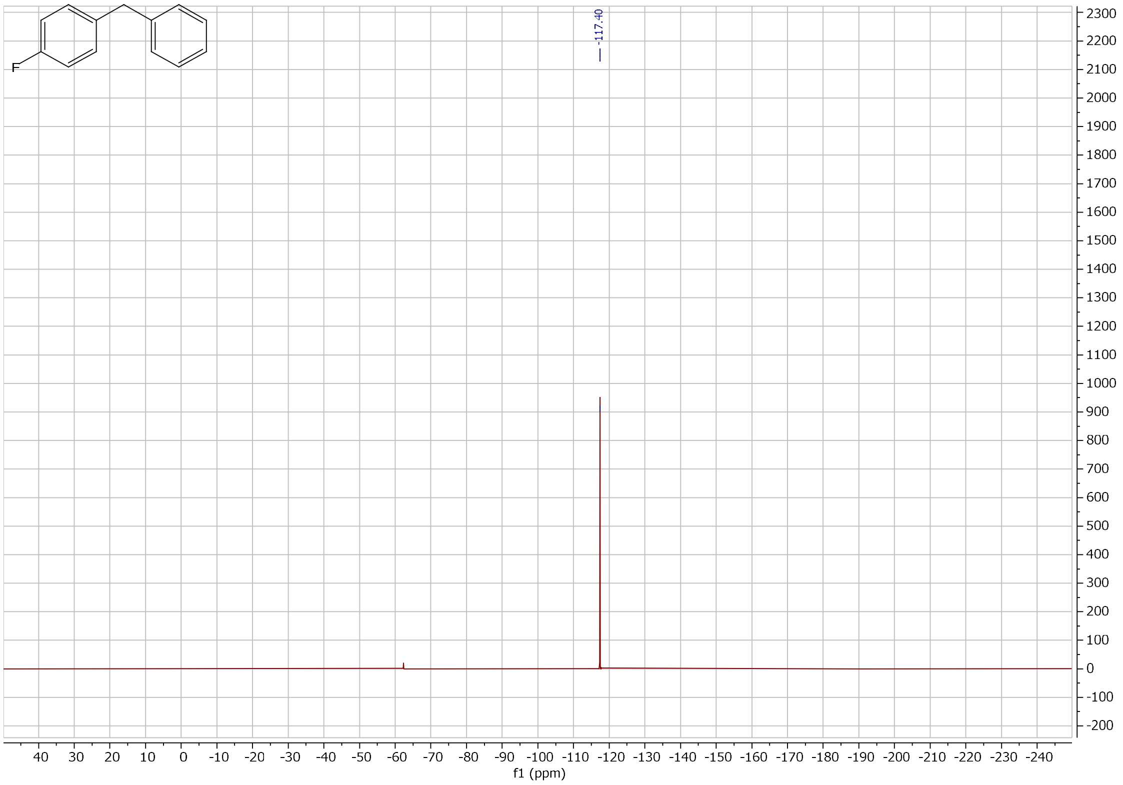

^{1}H NMR of 1-benzyl-2-methoxybenzene (**21**)

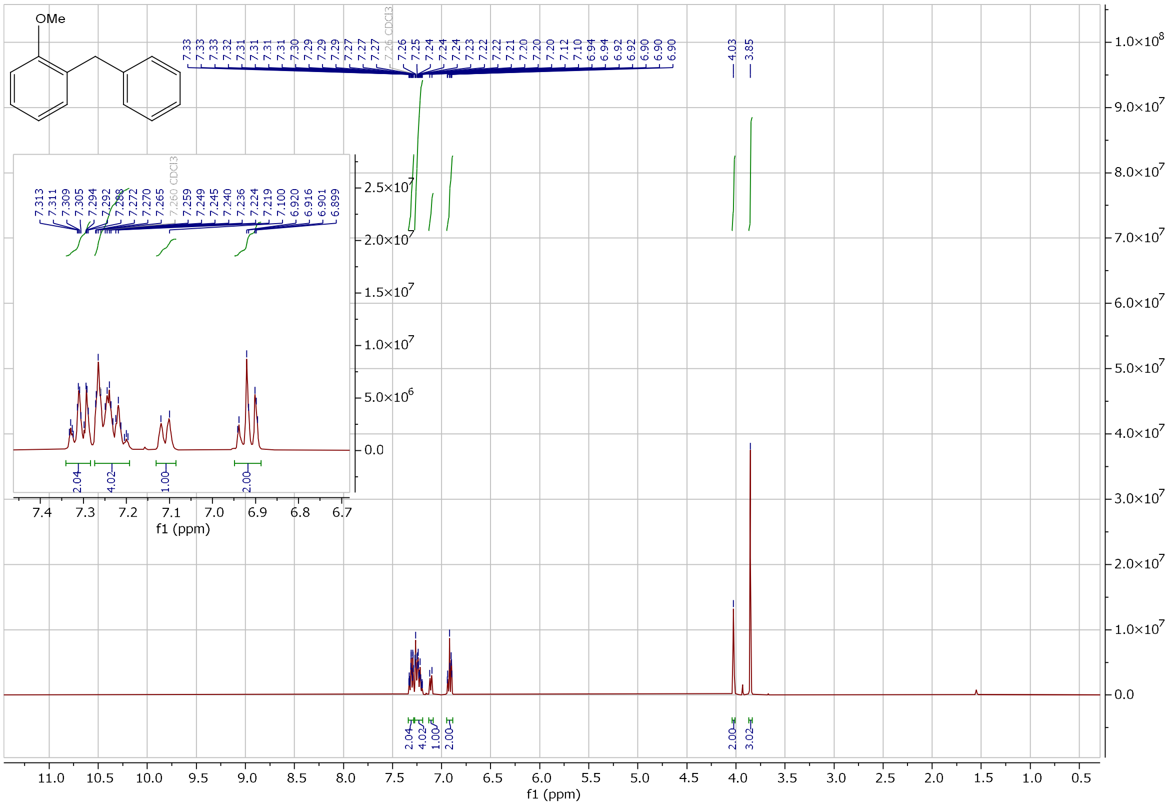

^{13}C NMR of 1-benzyl-2-methoxybenzene (**21**)

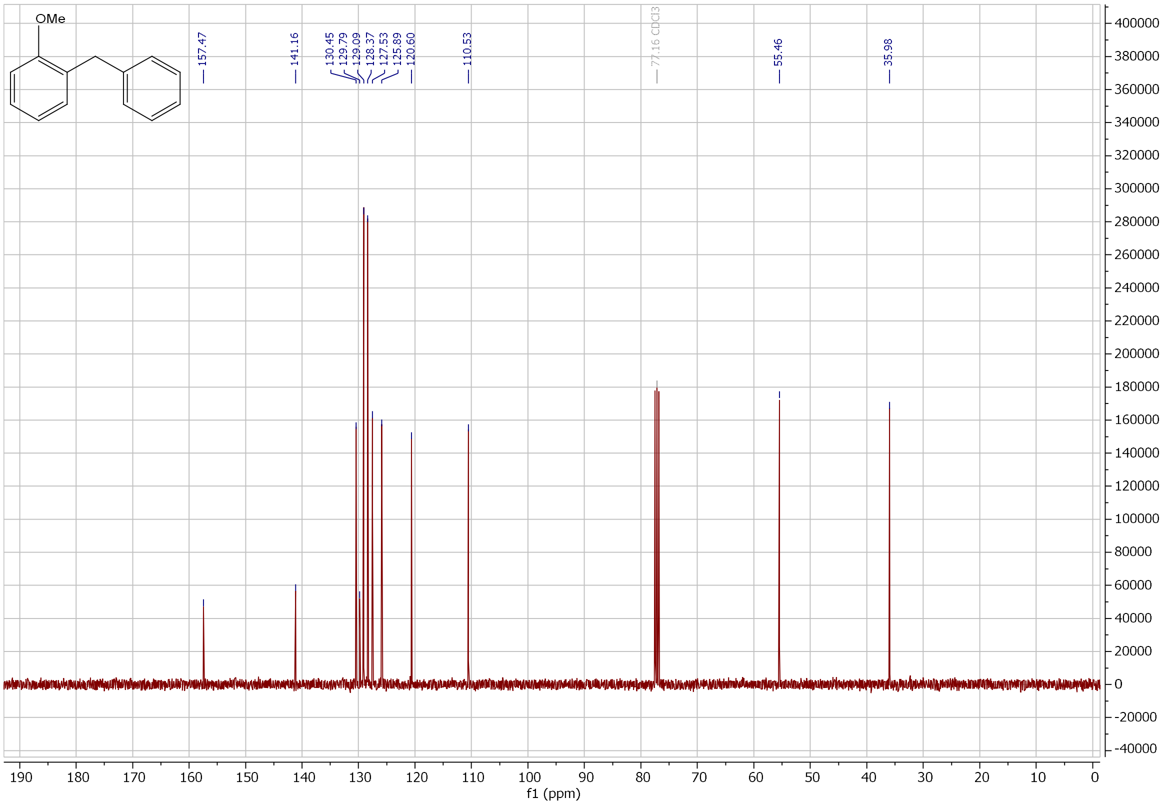

^{1}H NMR of 1-benzyl-3-methylbenzene (**22**)

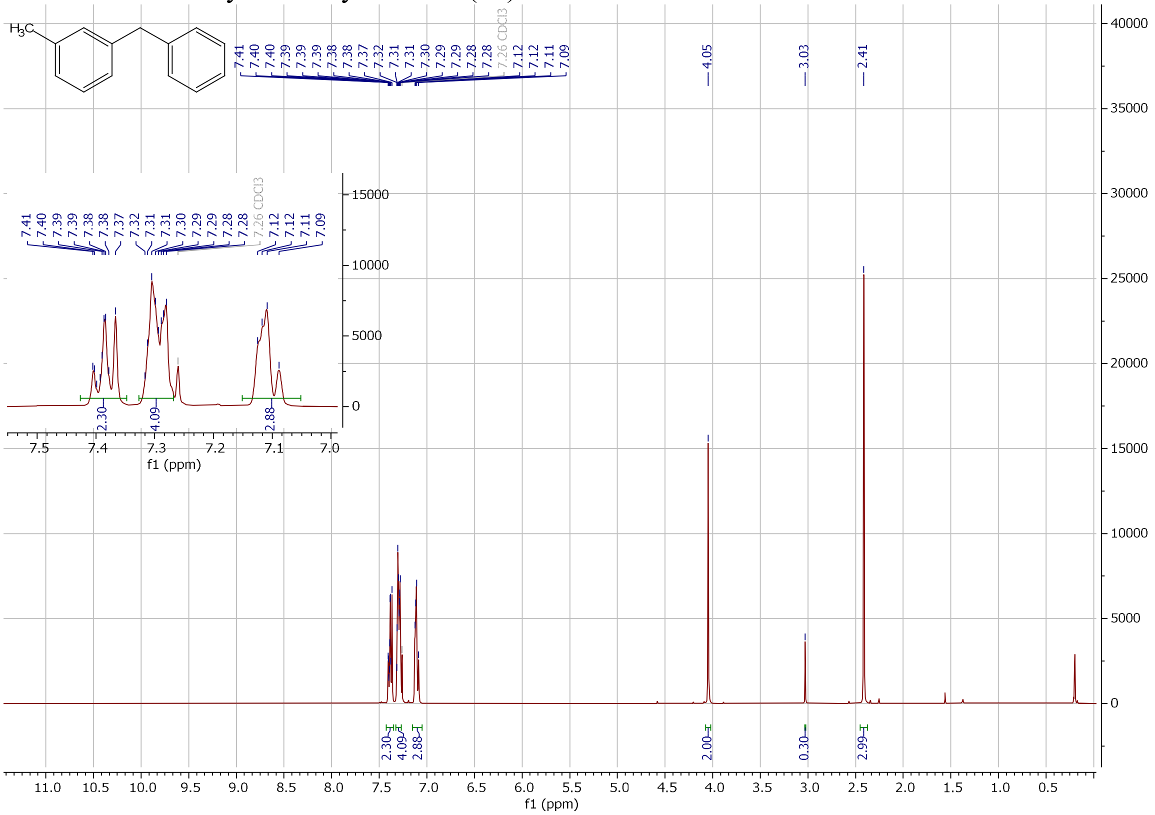

[13]H NMR of 1-benzyl-3-methylbenzene (**22**)

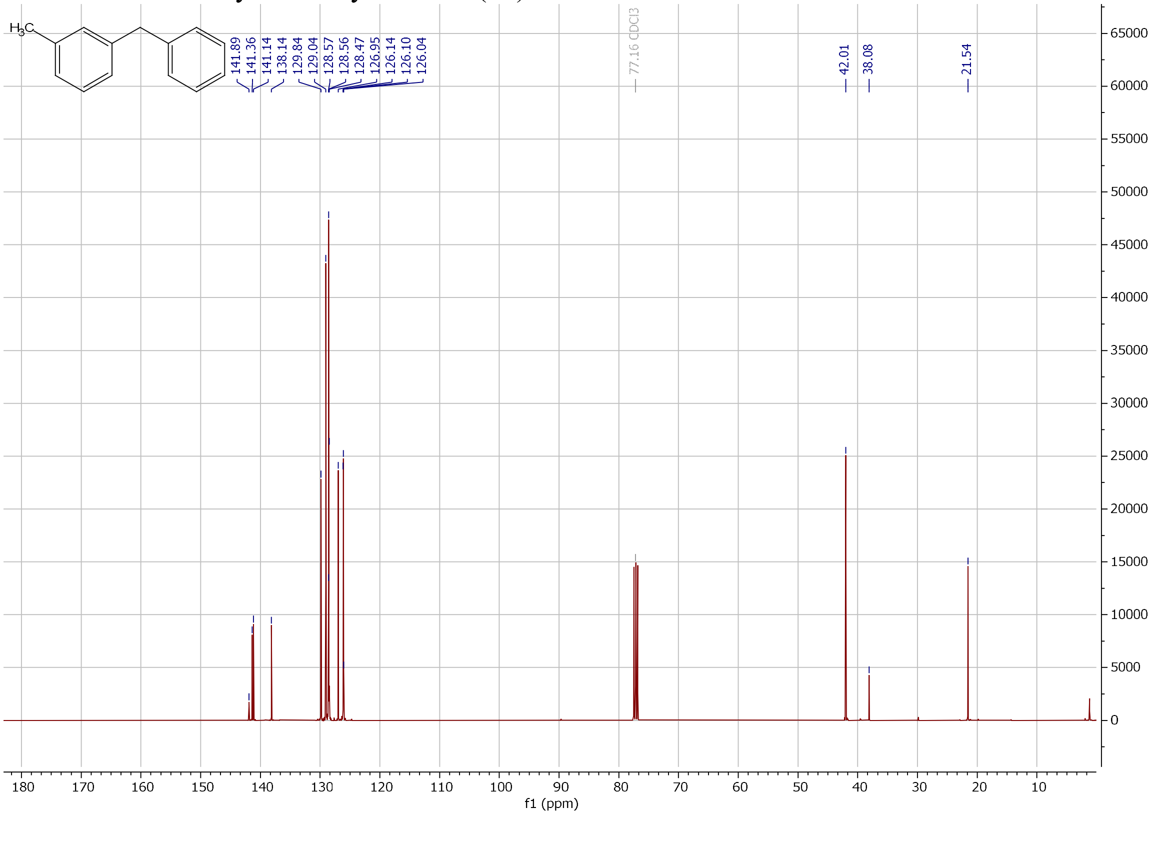

^{1}H NMR of 1-benzyl-2-methylbenzene (**23**)

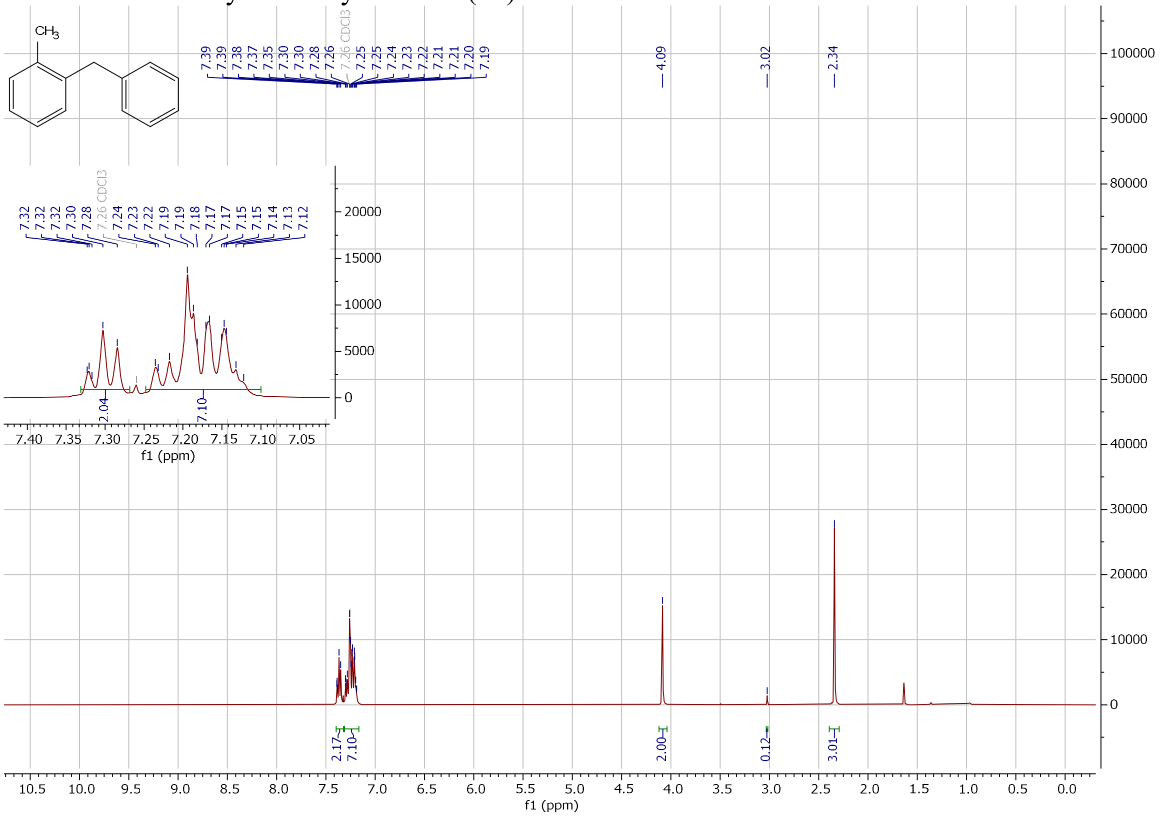

^{13}C NMR of 1-benzyl-2-methylbenzene (**23**)

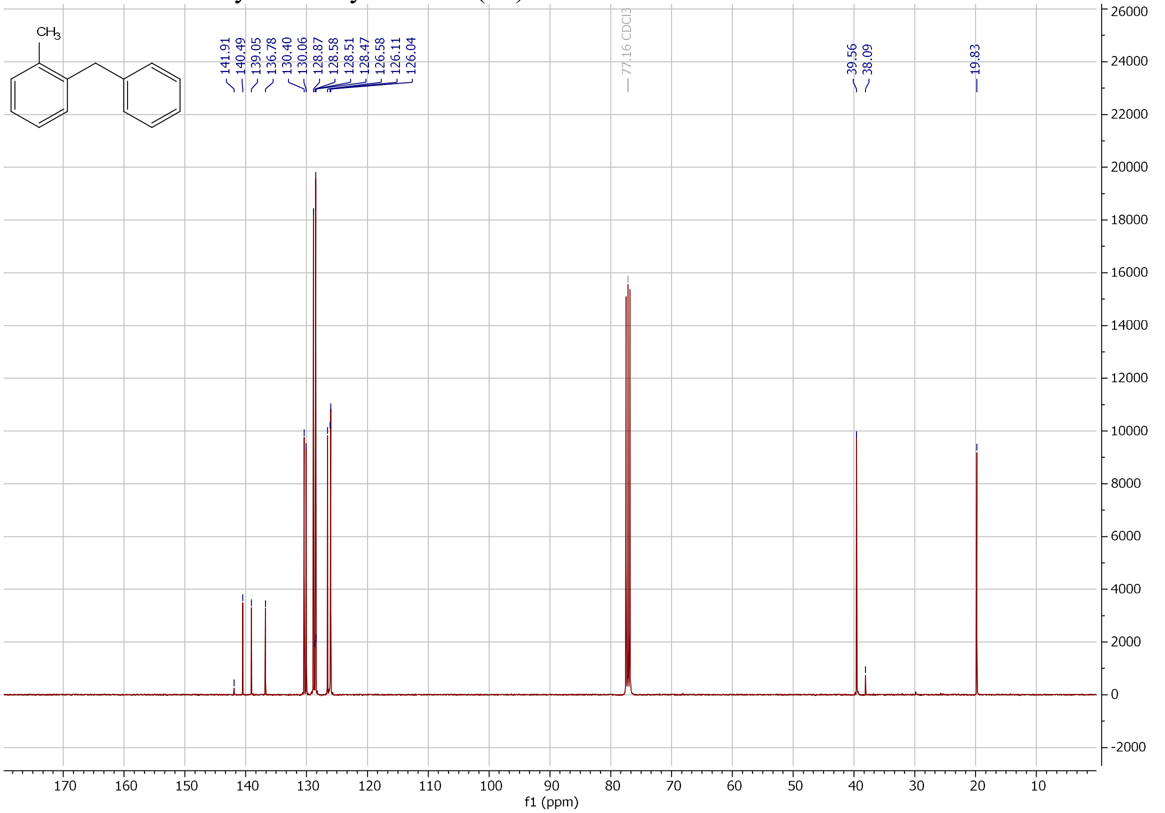

^{1}H NMR of 1-benzyl-4-(tert-butyl)benzene (**24**)

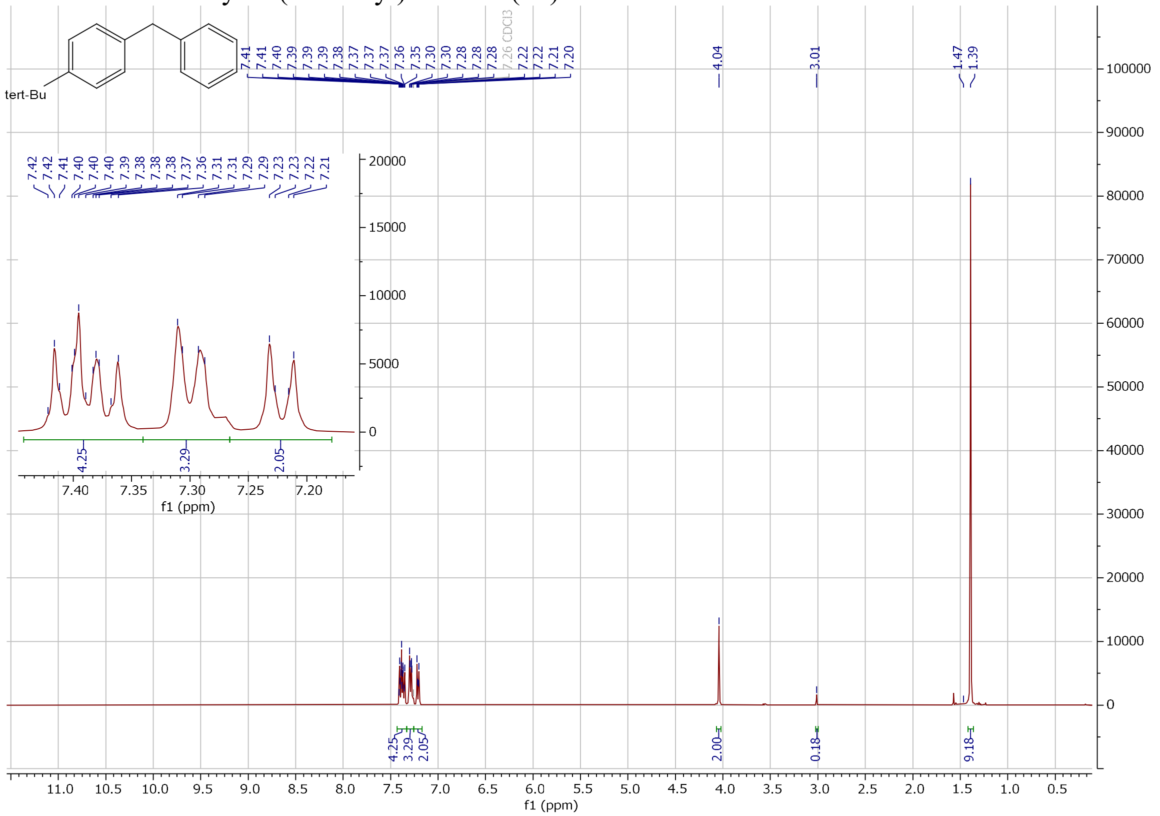

^{13}C NMR of 1-benzyl-4-(tert-butyl)benzene (**24**)

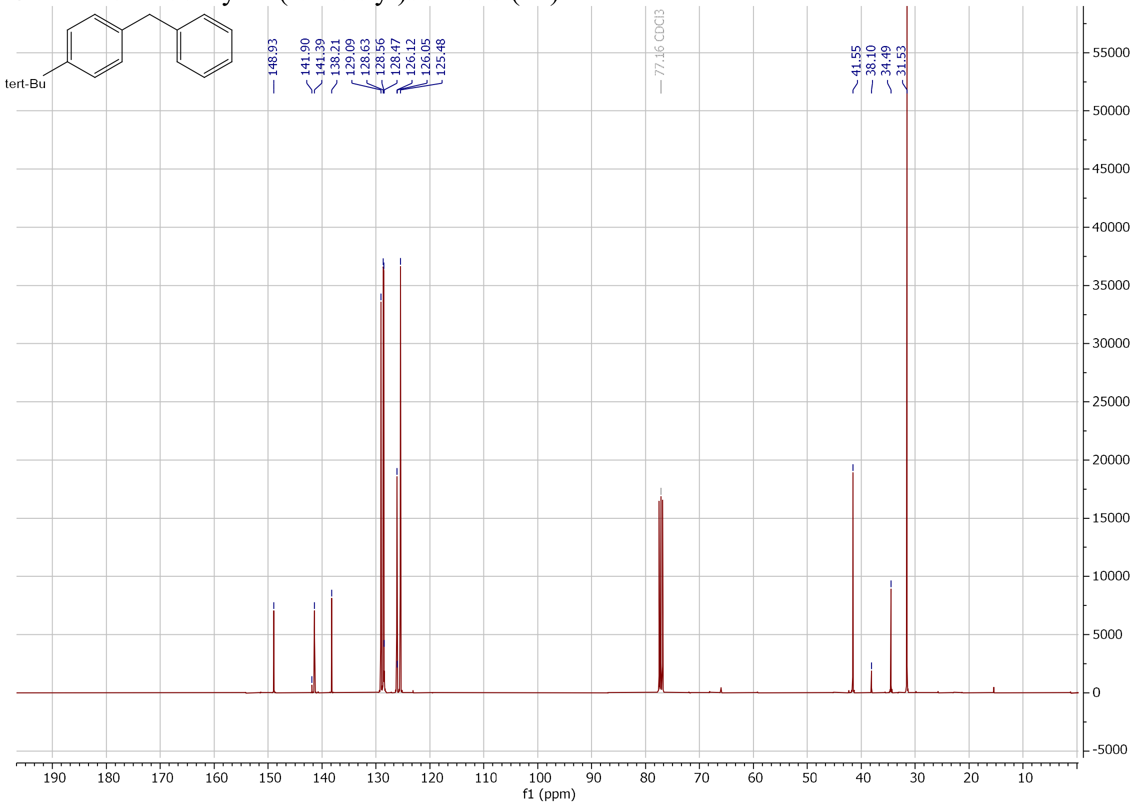

^{1}H NMR of 1-benzyl-4-methoxybenzene (**8**)

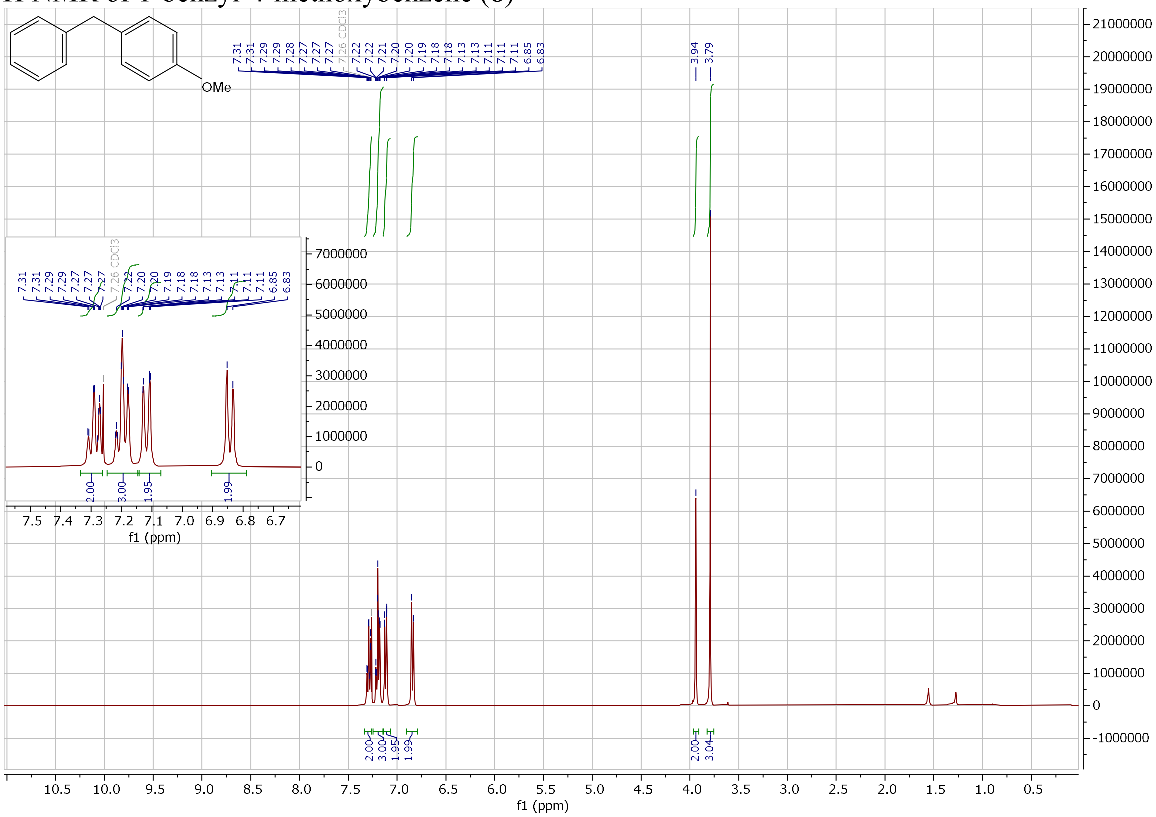

^{13}C NMR of 1-benzyl-4-methoxybenzene (**8**)

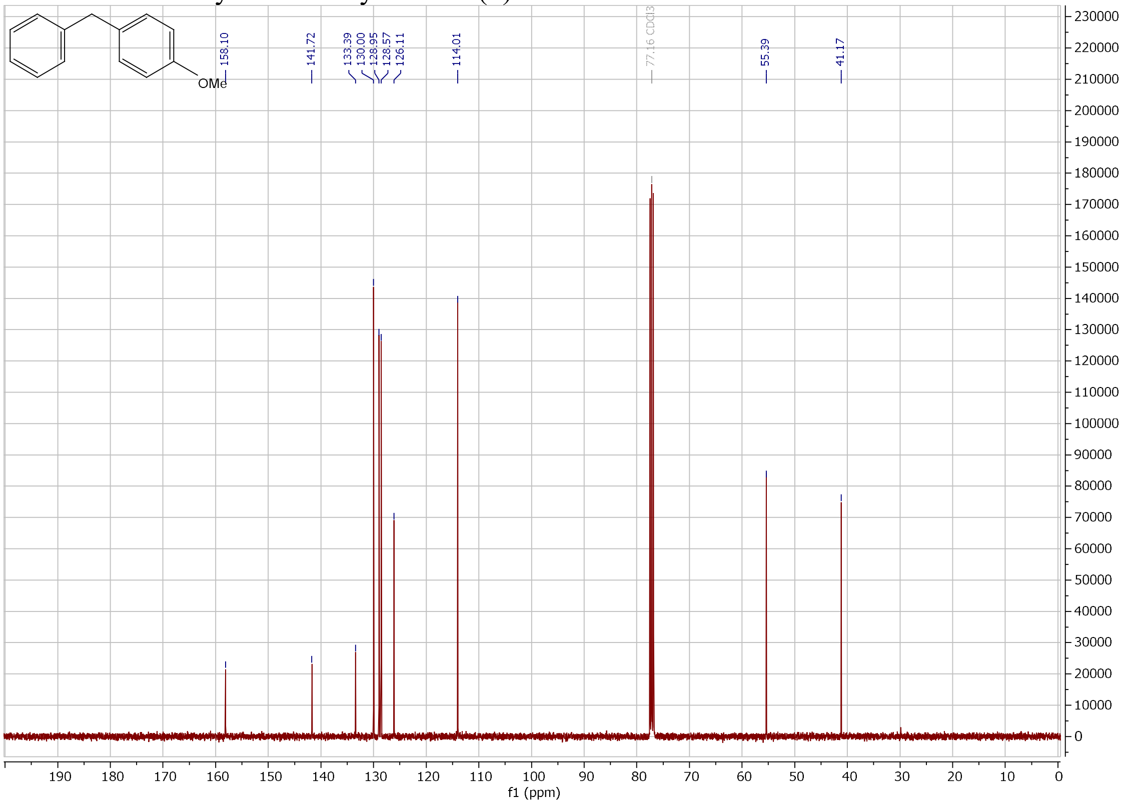

^{1}H NMR of 5-benzylbenzofuran (**25**)

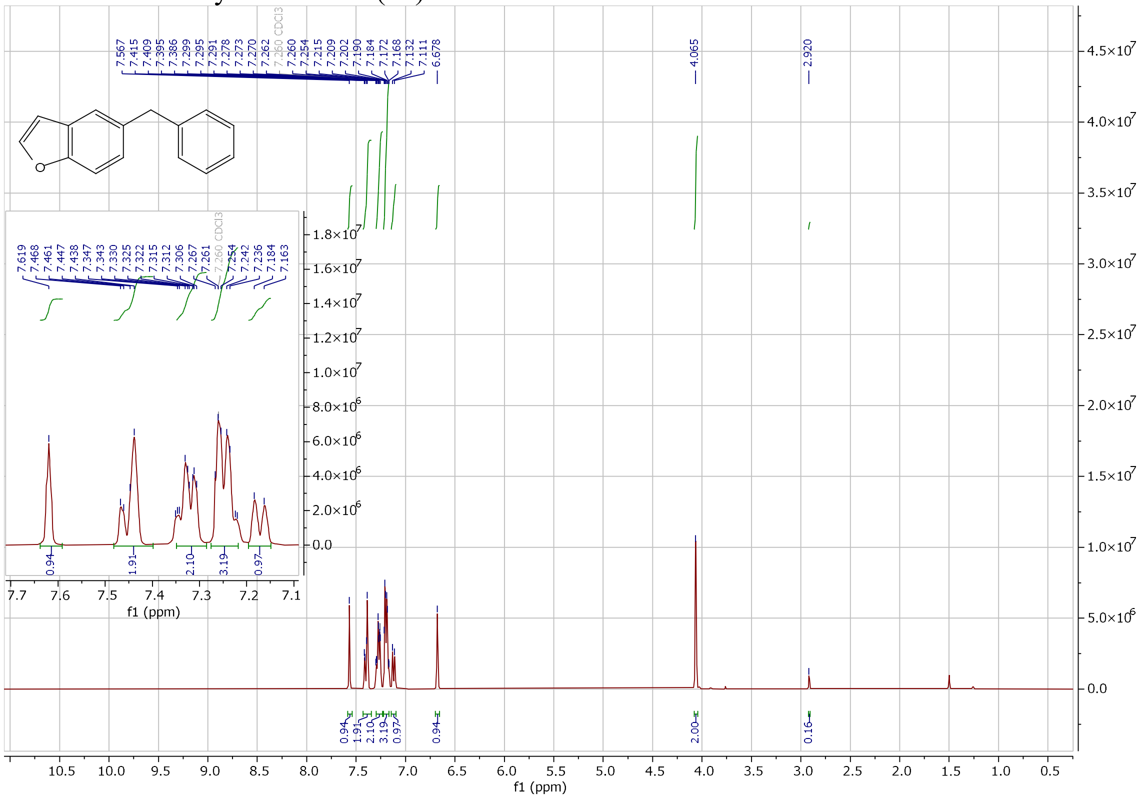

^{13}C NMR of 5-benzylbenzofuran (**25**)

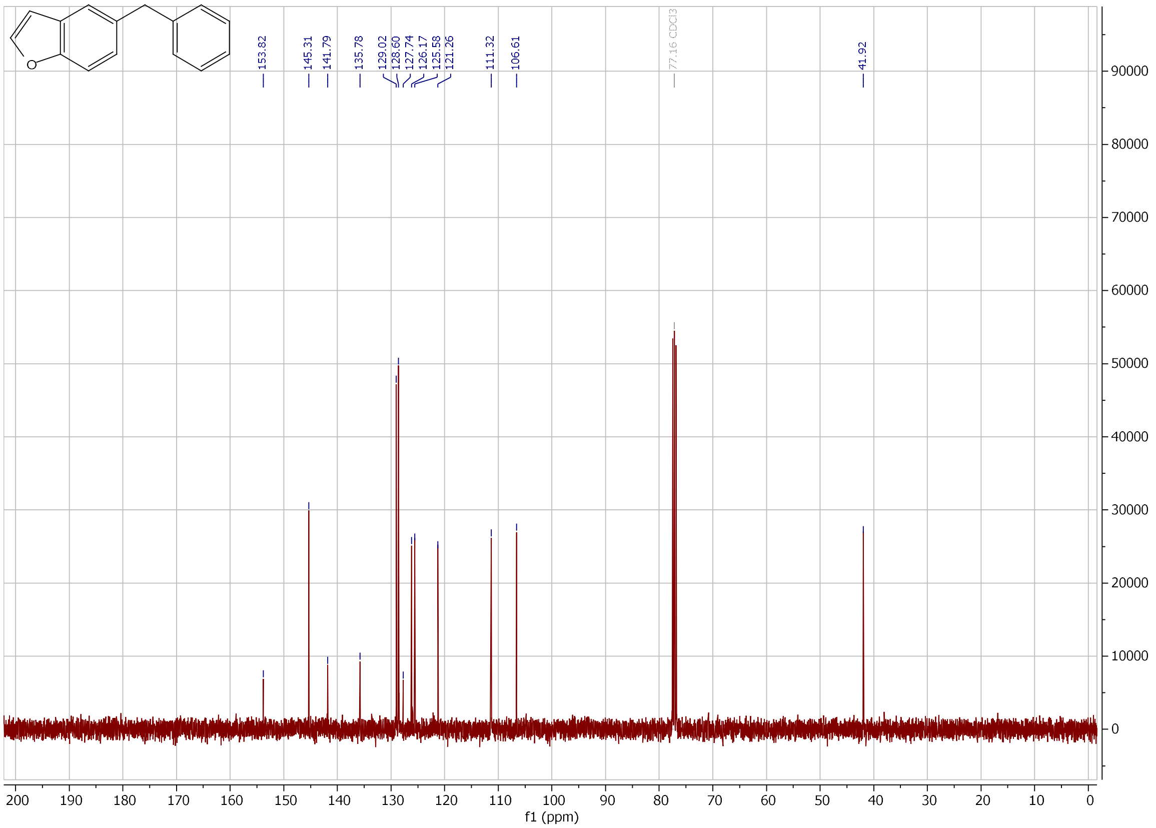

^{1}H NMR of 2-benzylpyridine (**26**)

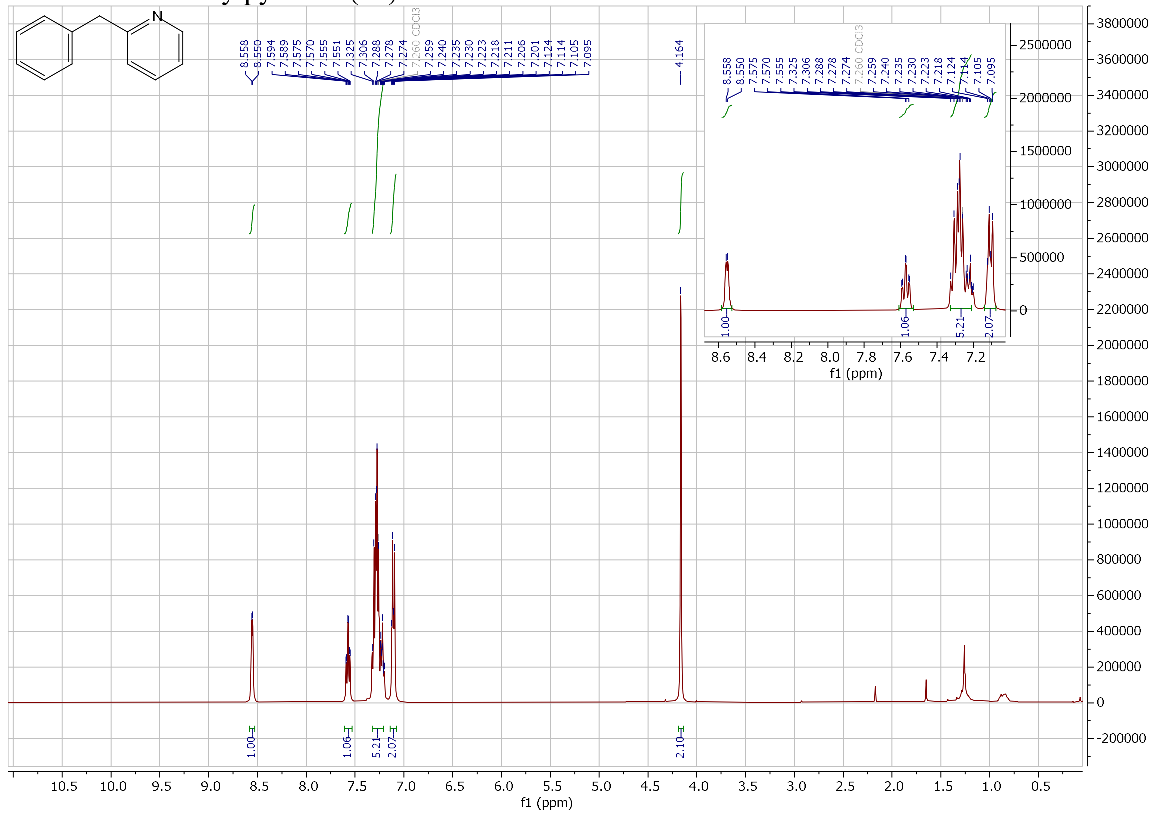

^{13}C NMR of 2-benzylpyridine (**26**)

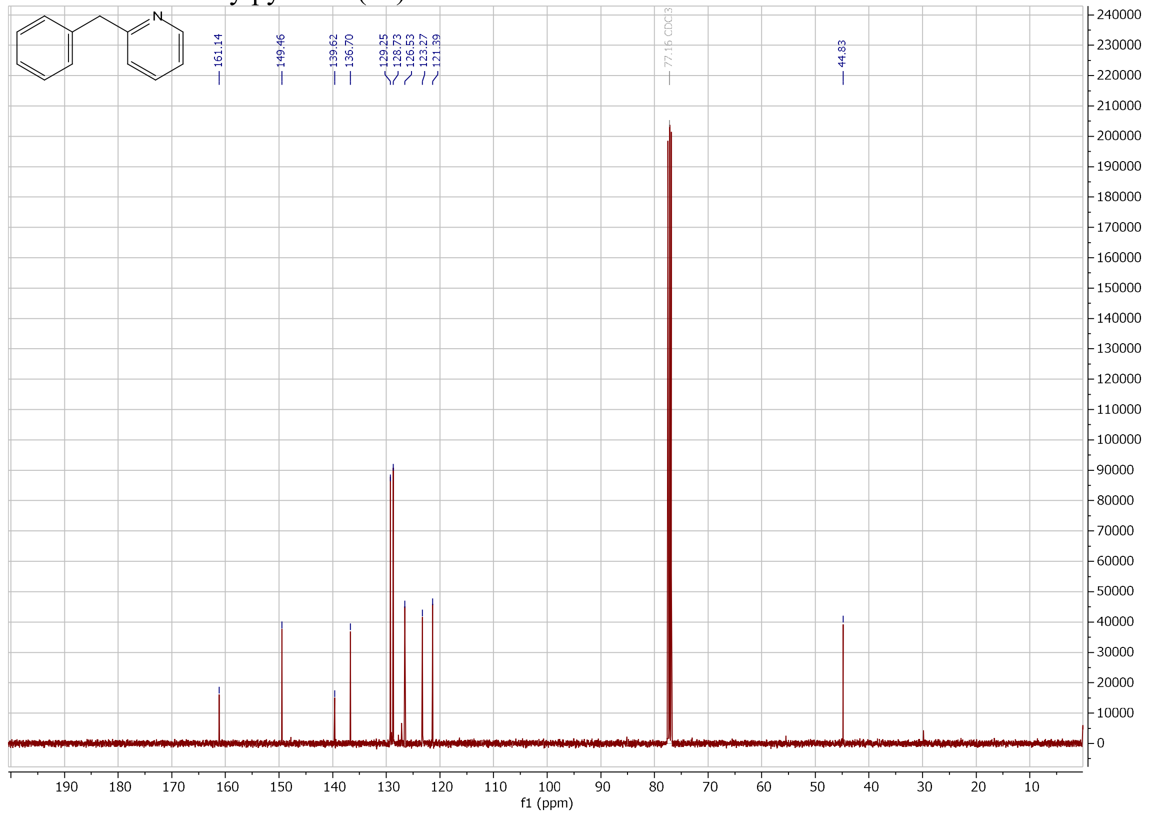

^{1}H NMR of 5-benzylpyrimidine (**27**)

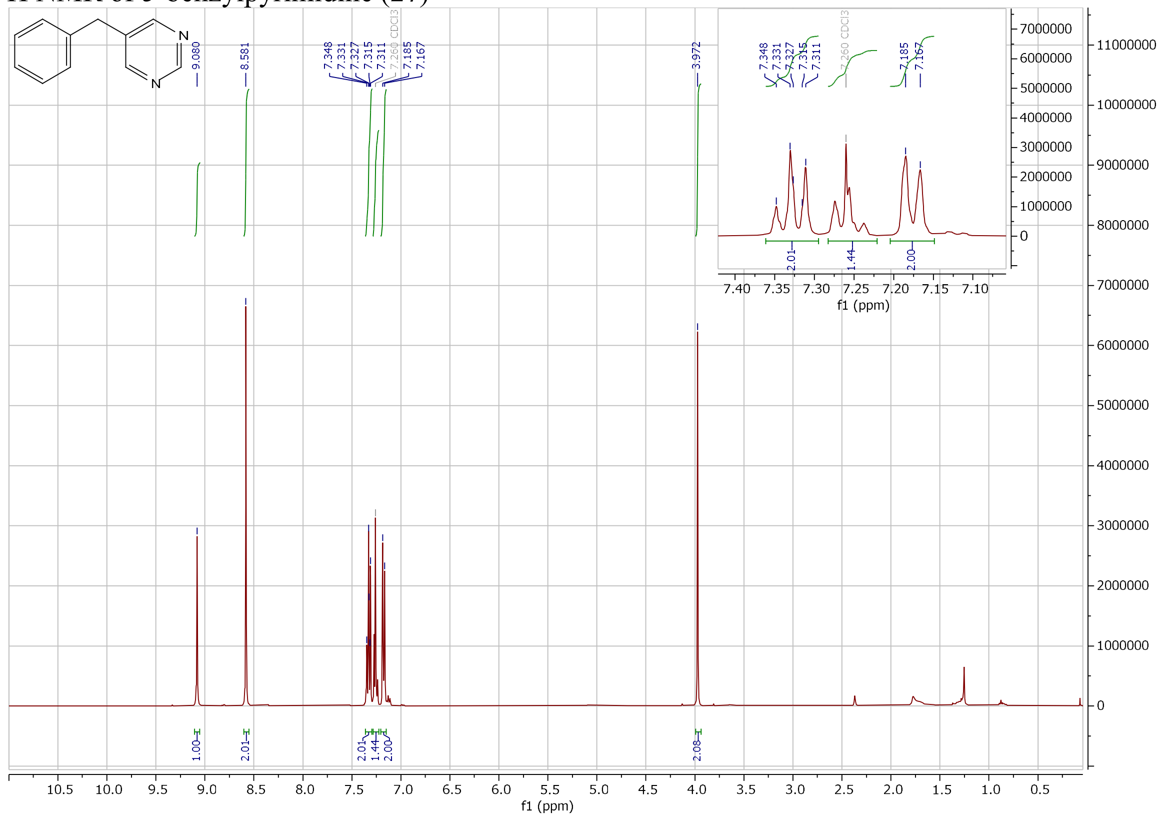

^{13}C NMR of 5-benzylpyrimidine (**27**)

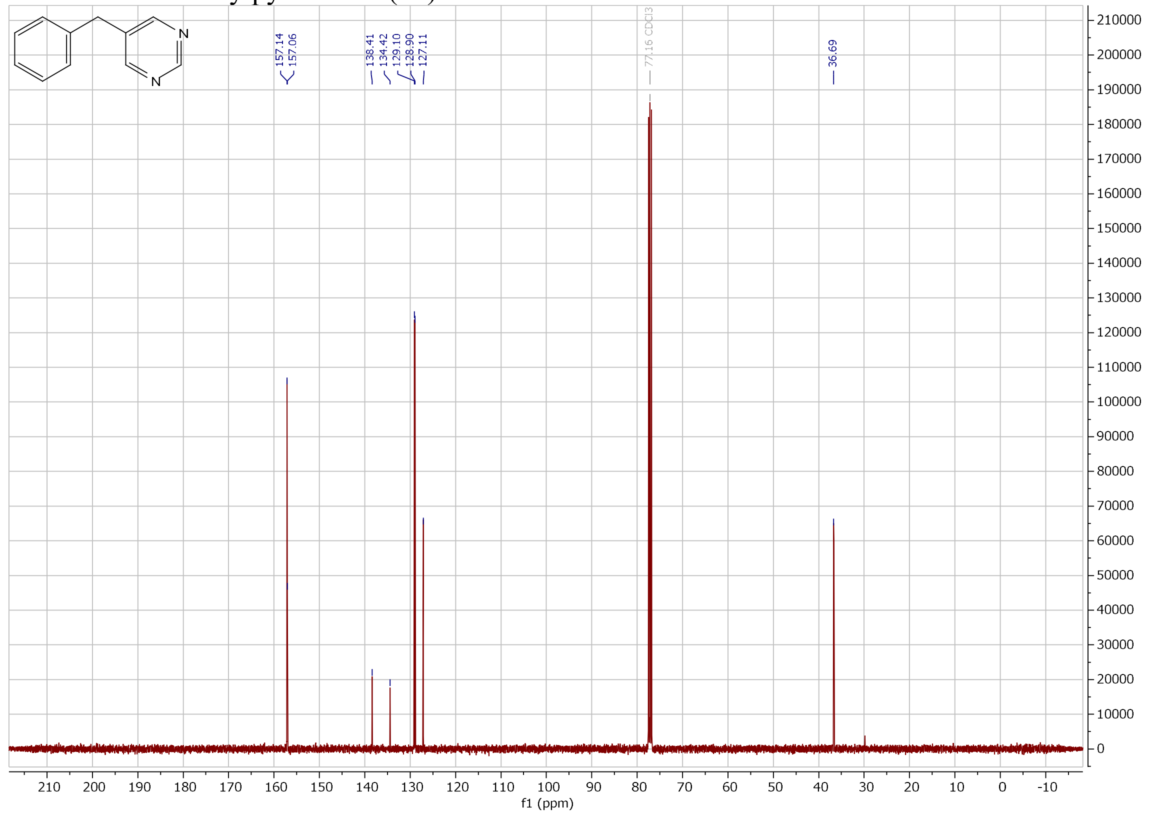

^{1}H NMR of 5-benzyl-2-chloropyrimidine (**29**)

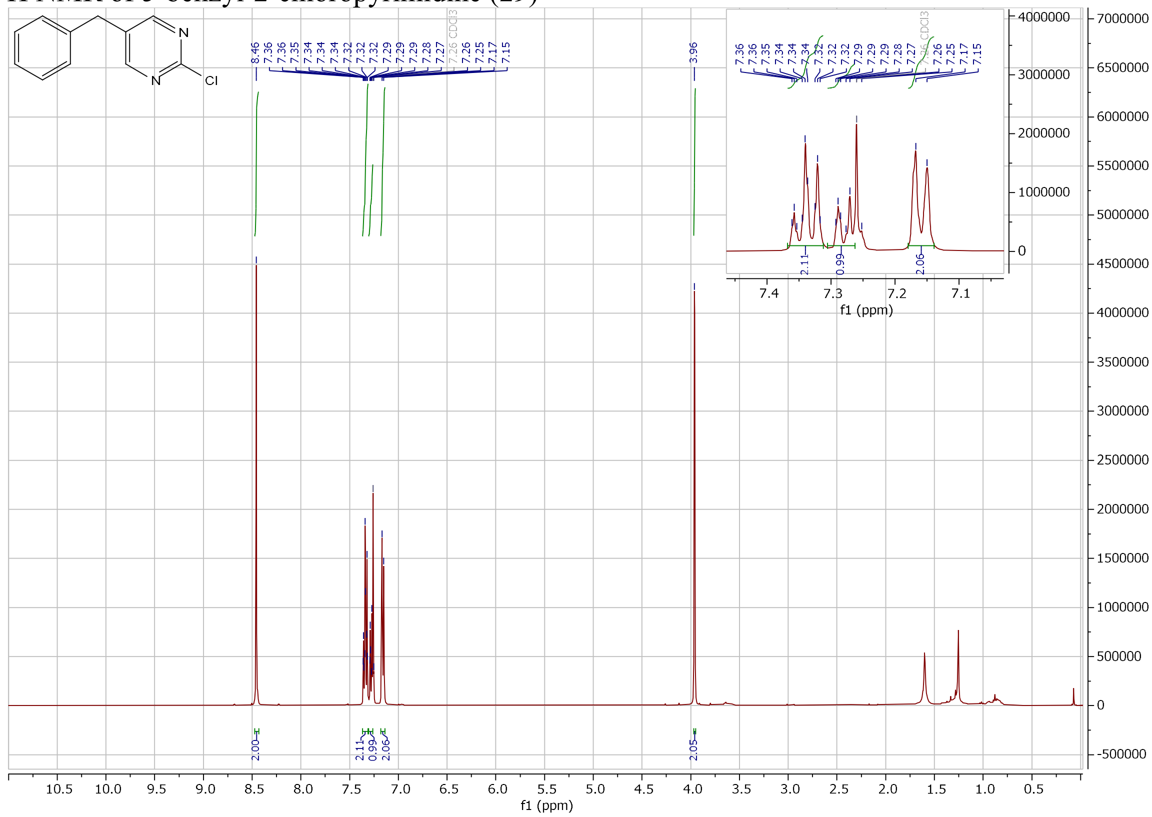

^{13}C NMR of 5-benzyl-2-chloropyrimidine (**29**)

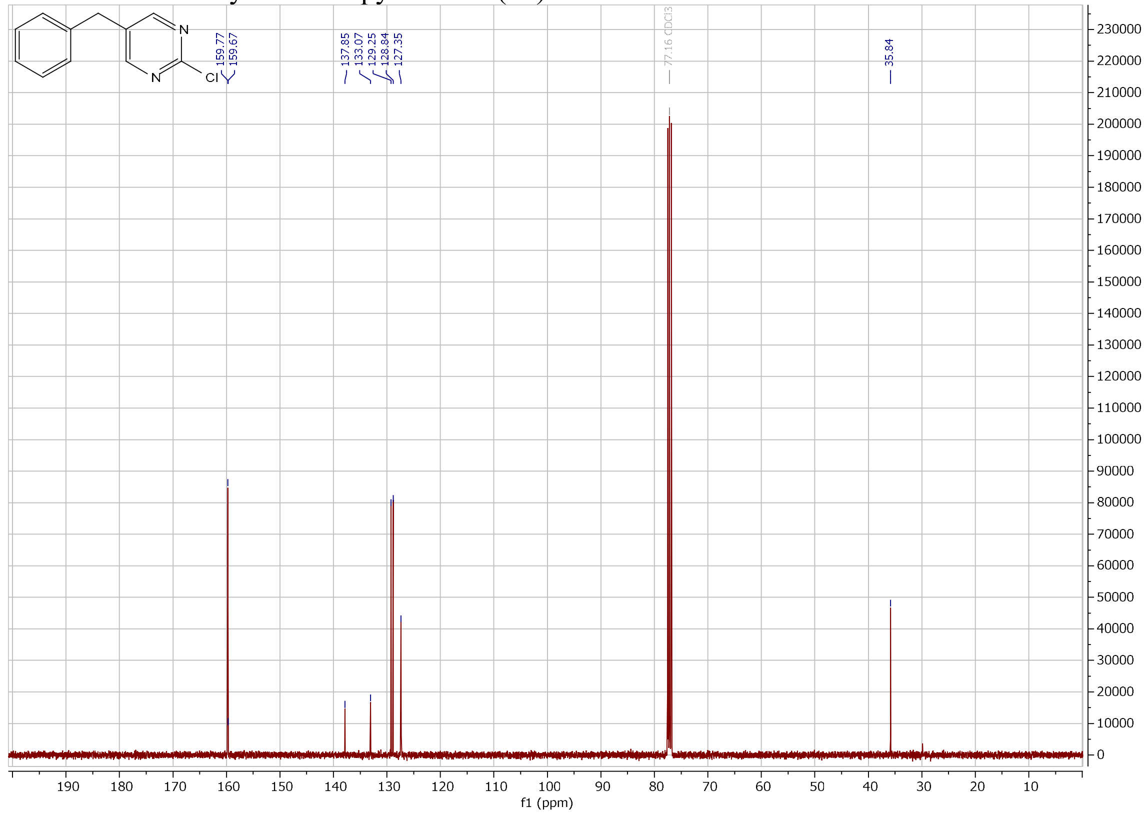

^{1}H NMR of 2,5-dibenzylpyrimidine (**30**)

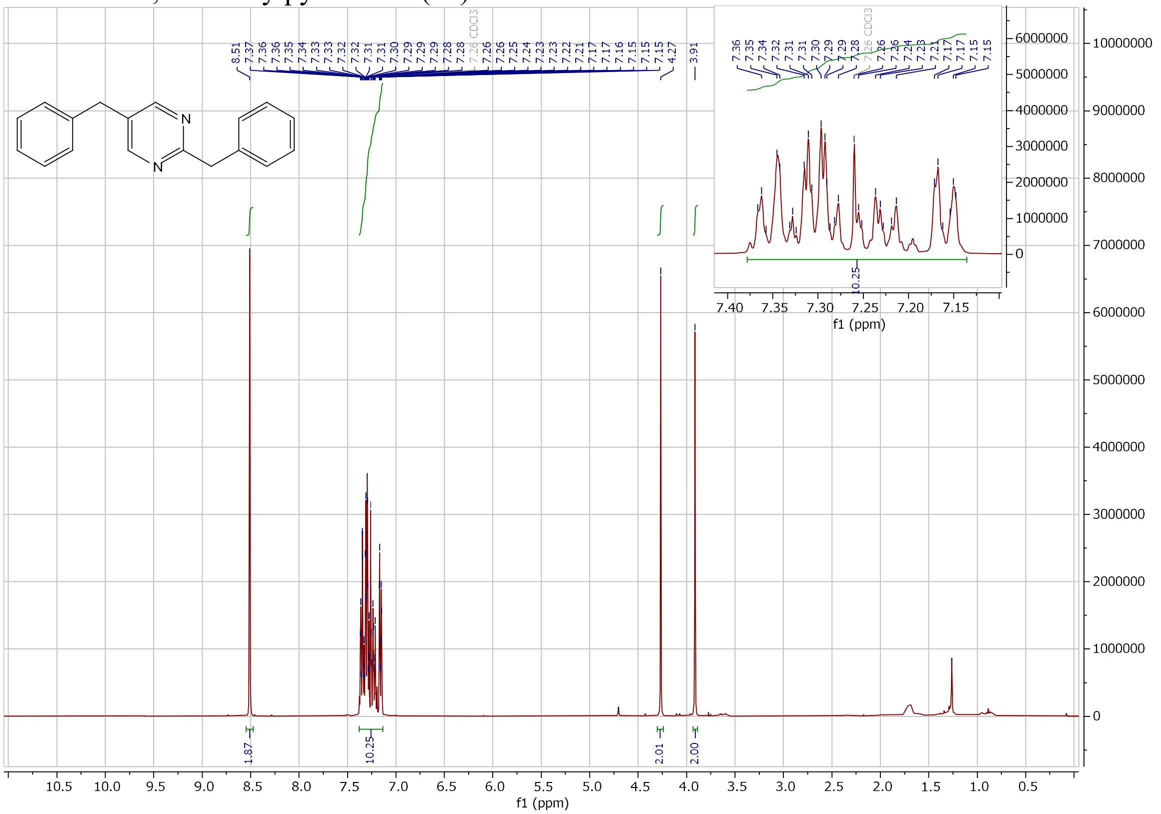

^{13}C NMR of 2,5-dibenzylpyrimidine (**30**)

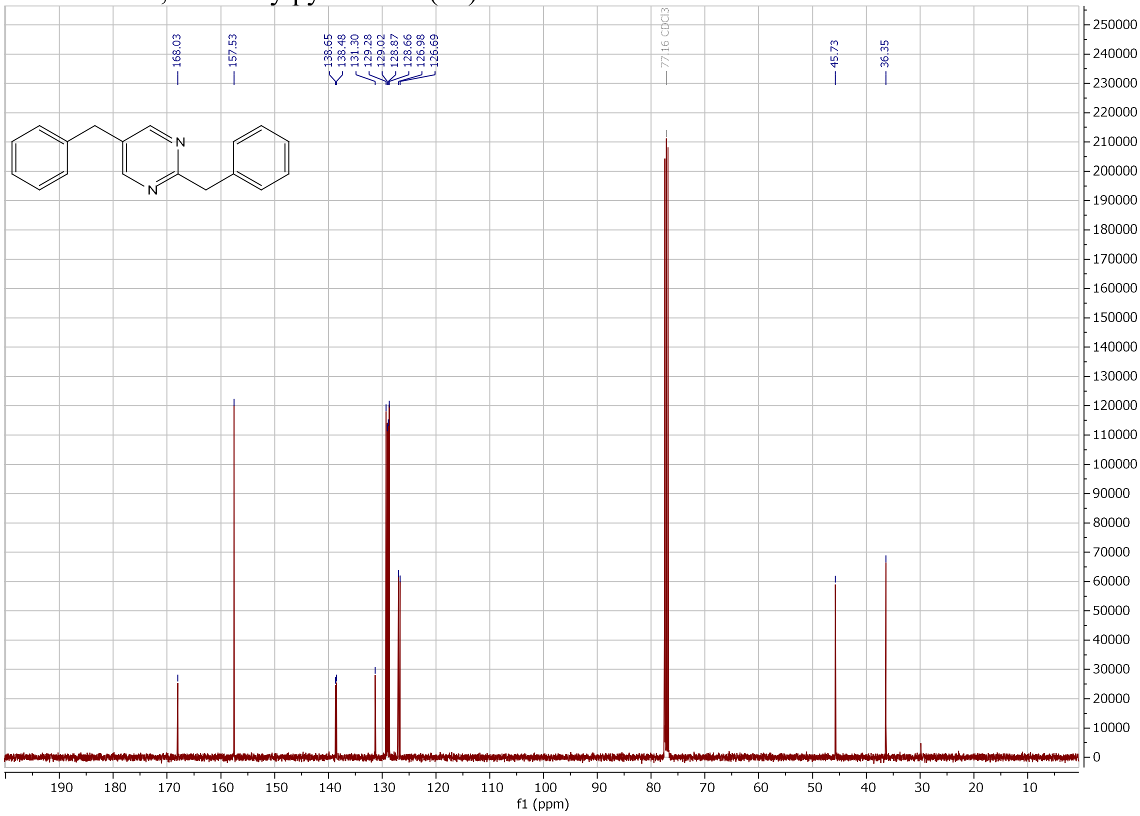

^{1}H NMR of 4-(4-(trifluoromethyl)benzyl)-1,1'-biphenyl (**31**)

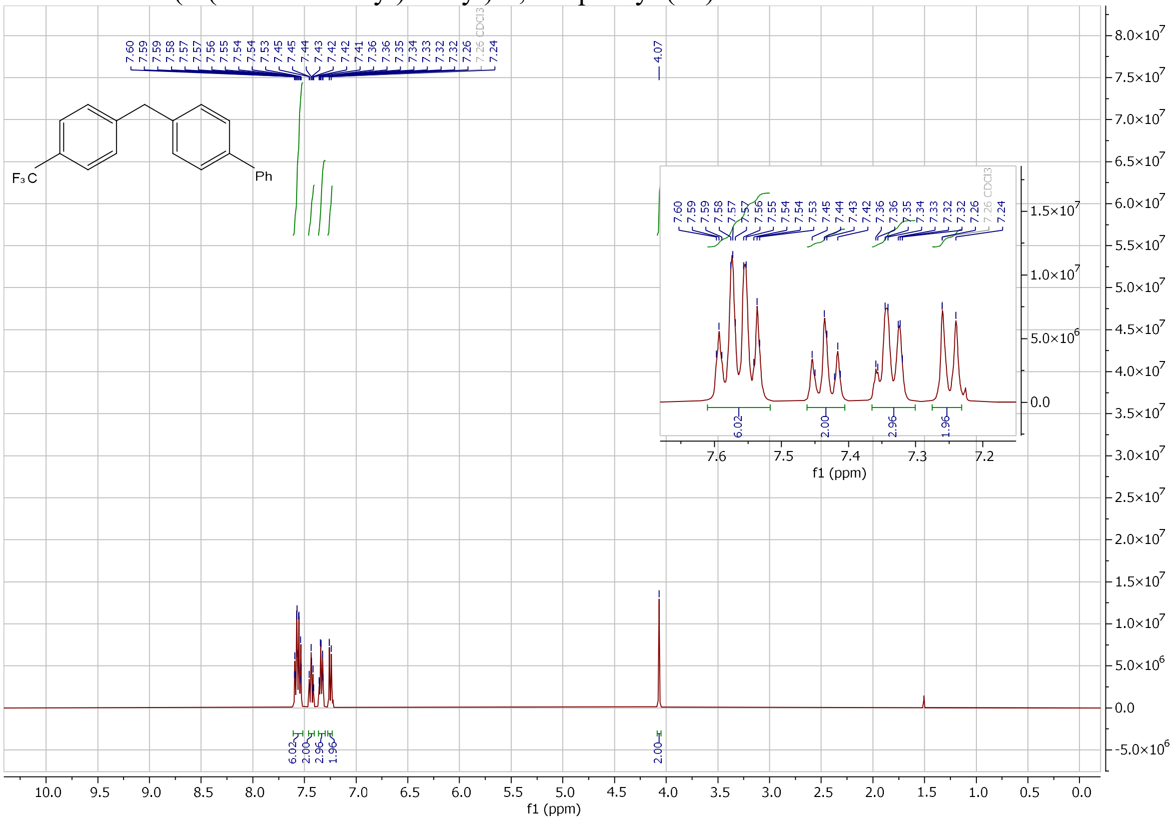

^{13}C NMR of 4-(4-(trifluoromethyl)benzyl)-1,1'-biphenyl (**31**)

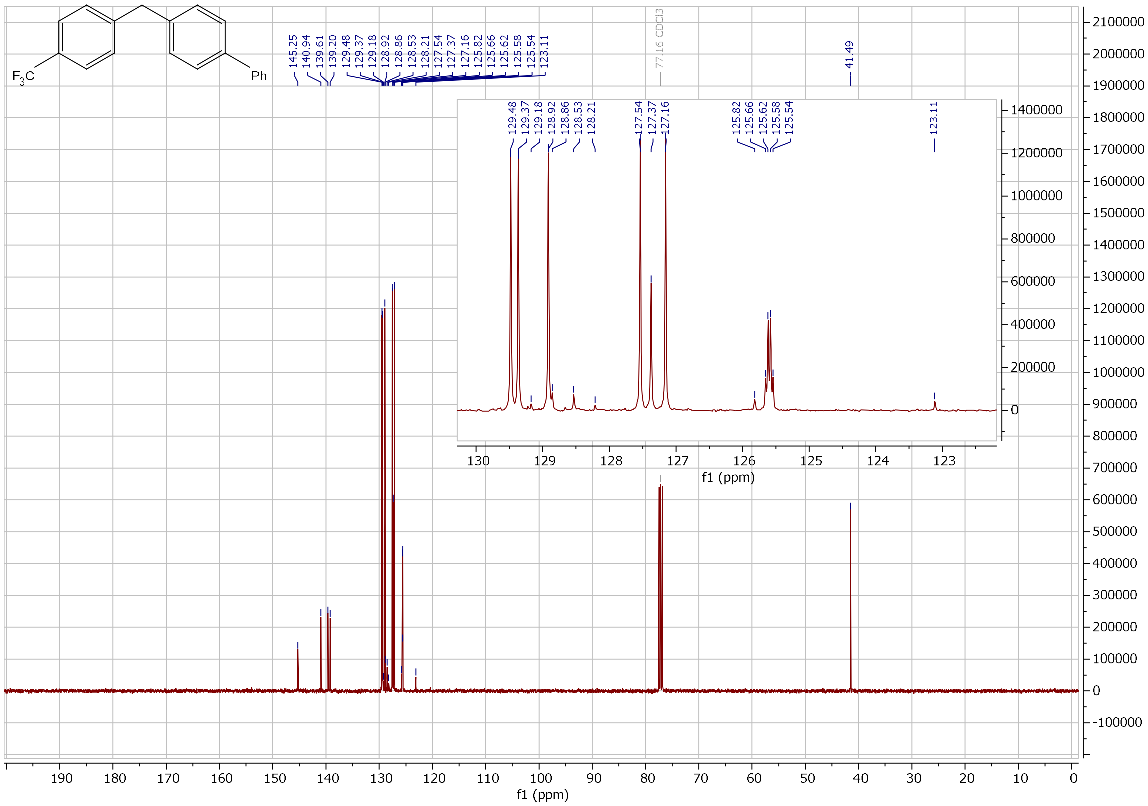

^{19}F NMR of 4-(4-(trifluoromethyl)benzyl)-1,1'-biphenyl (**31**)

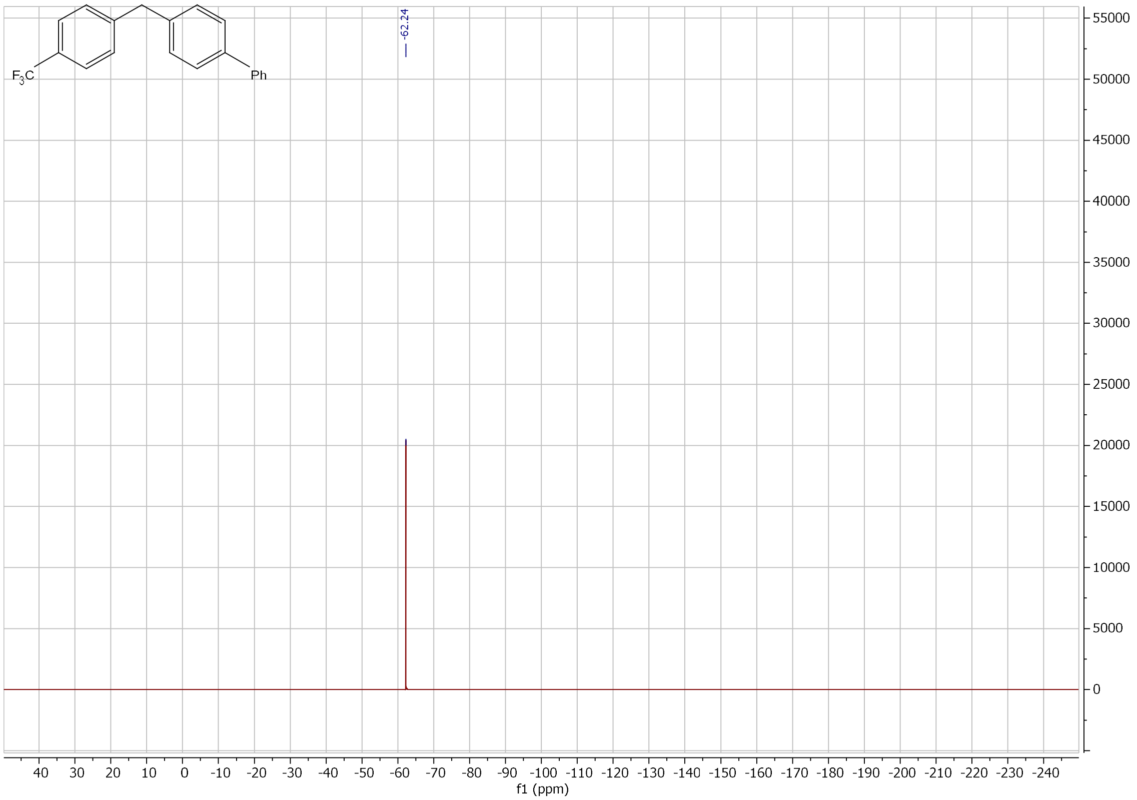

^{1}H NMR of 1-(tert-butyl)-4-(4-(trifluoromethyl)benzyl)benzene (**32**)

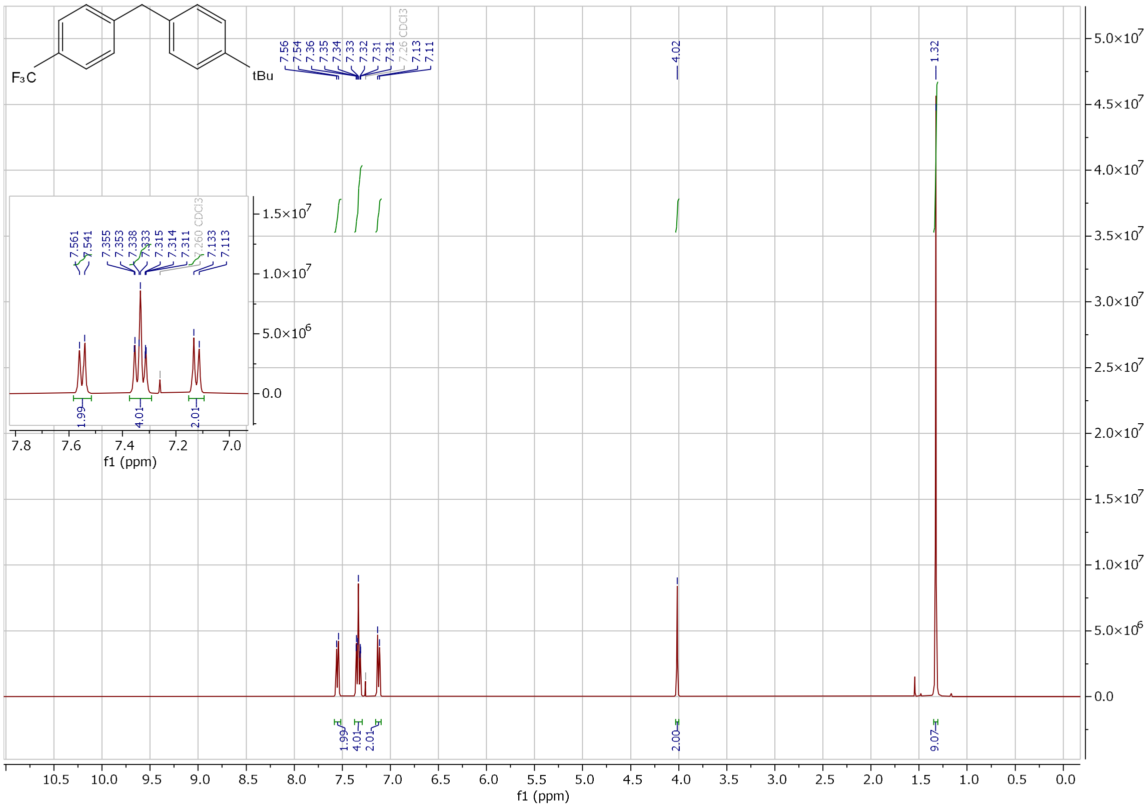

^{13}C NMR of 1-(tert-butyl)-4-(4-(trifluoromethyl)benzyl)benzene (**32**)

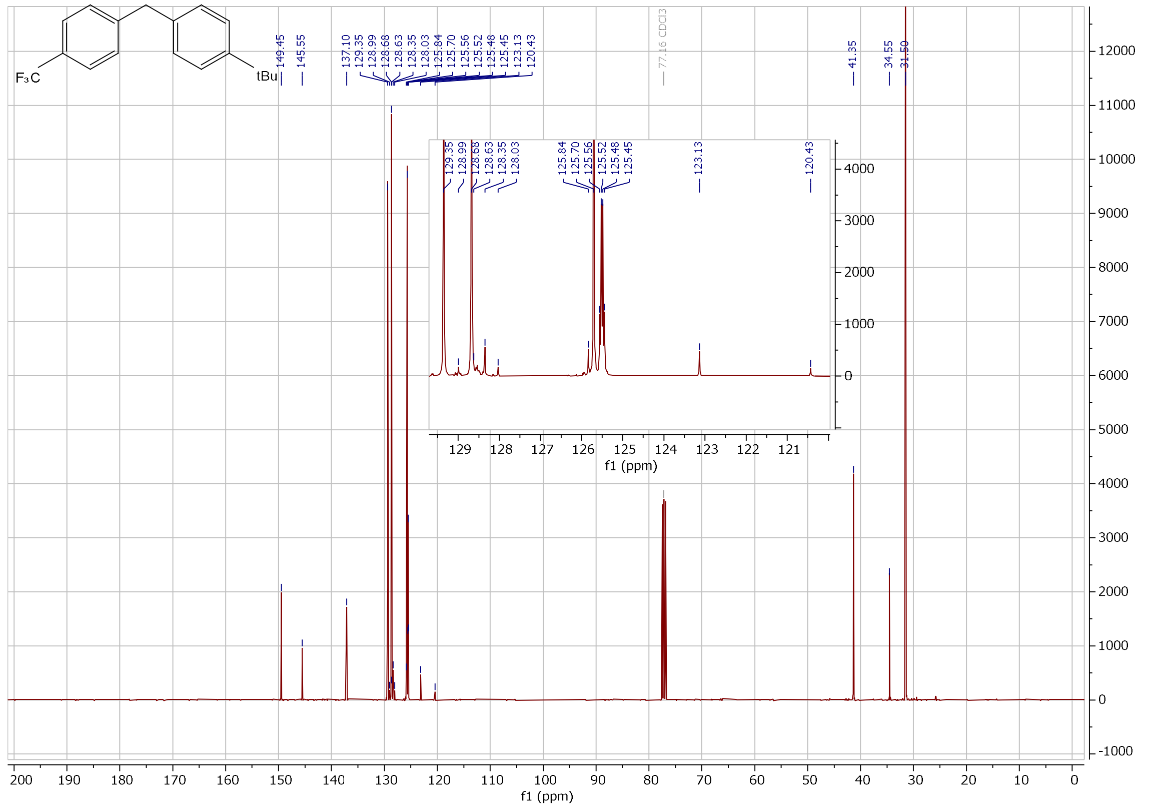

^{19}F NMR of 1-(tert-butyl)-4-(4-(trifluoromethyl)benzyl)benzene (**32**)

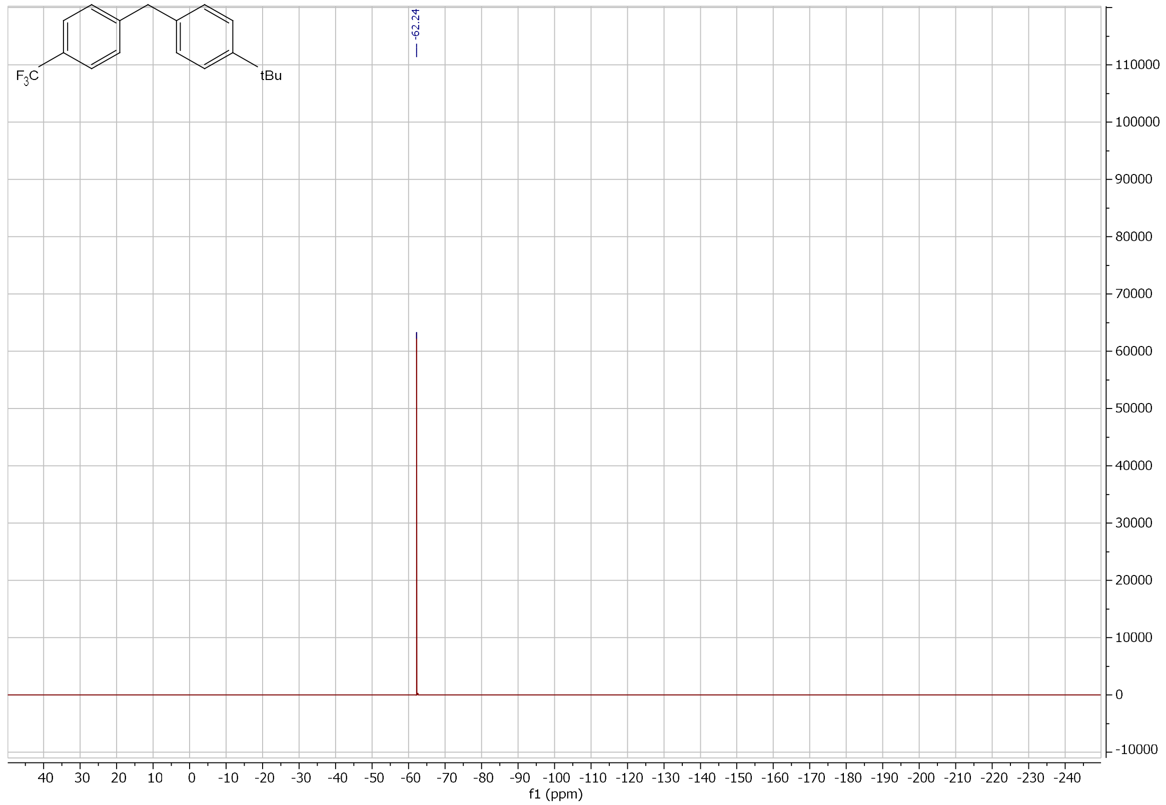

^{1}H NMR of 2-(4-(trifluoromethyl)benzyl)naphthalene (**33**)

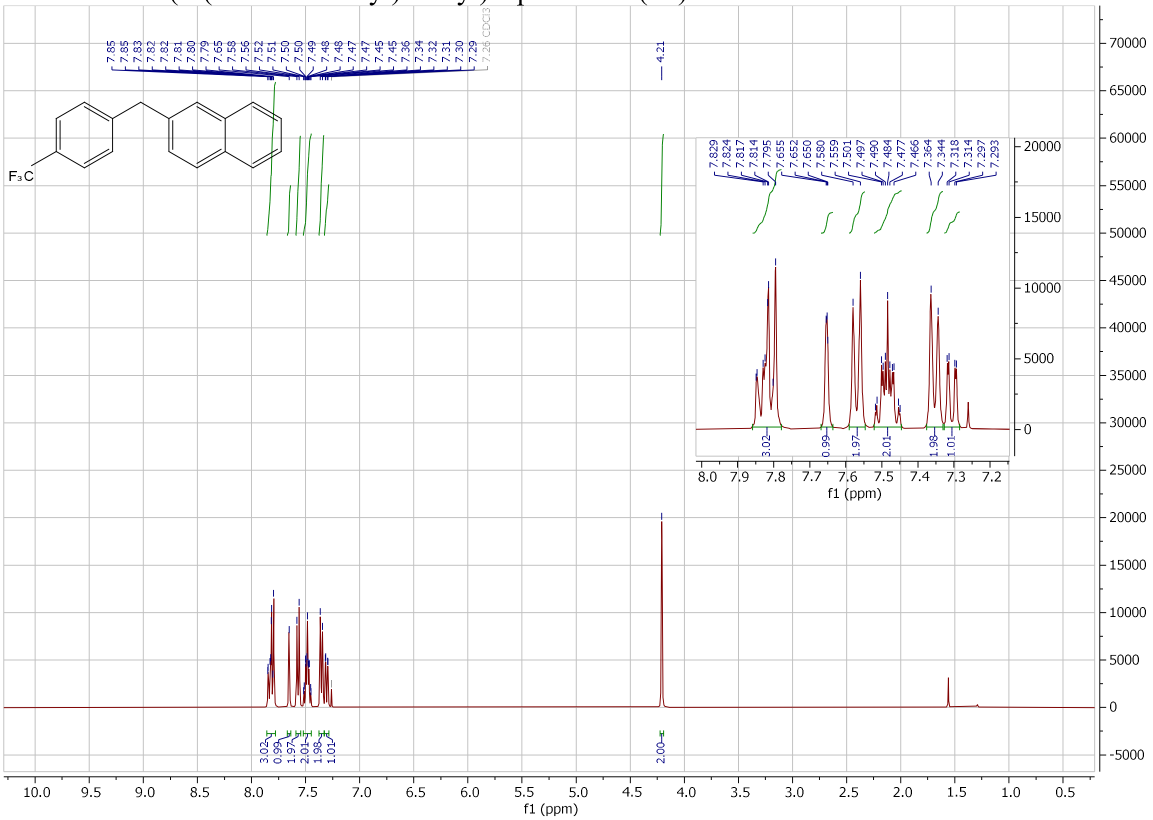

^{13}C NMR of 2-(4-(trifluoromethyl)benzyl)naphthalene (**33**)

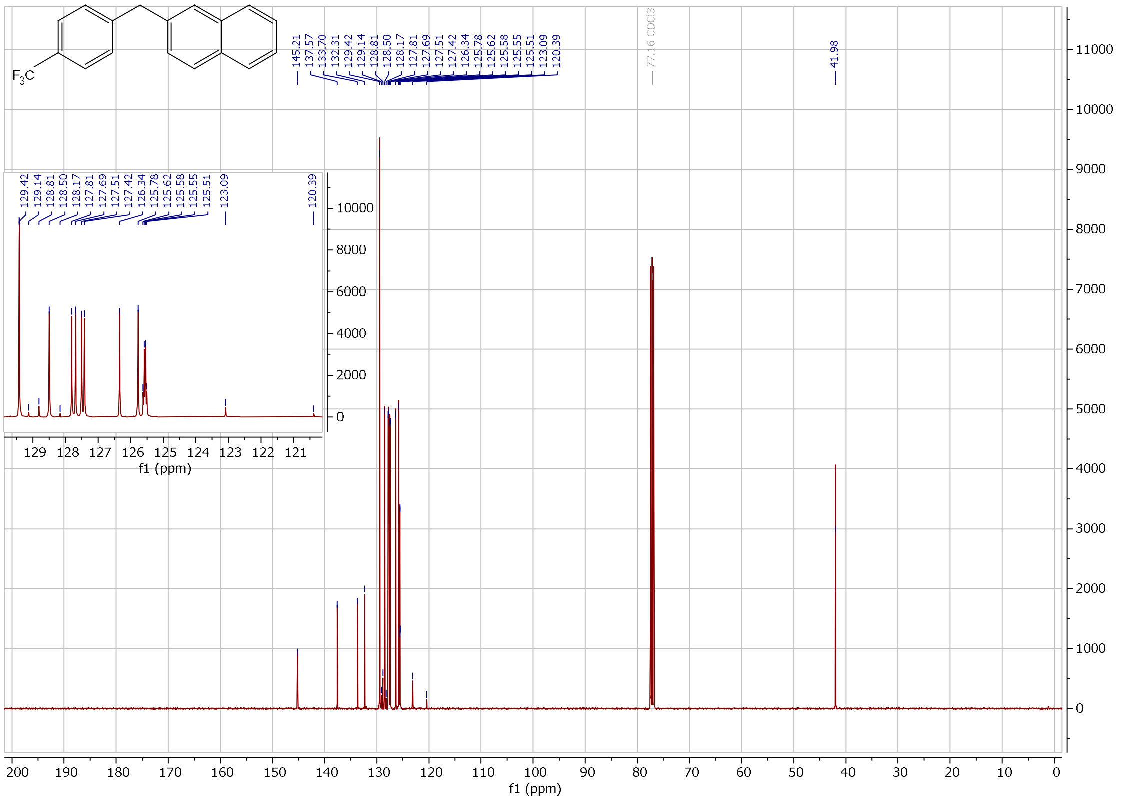

^{19}F NMR of 2-(4-(trifluoromethyl)benzyl)naphthalene (**33**)

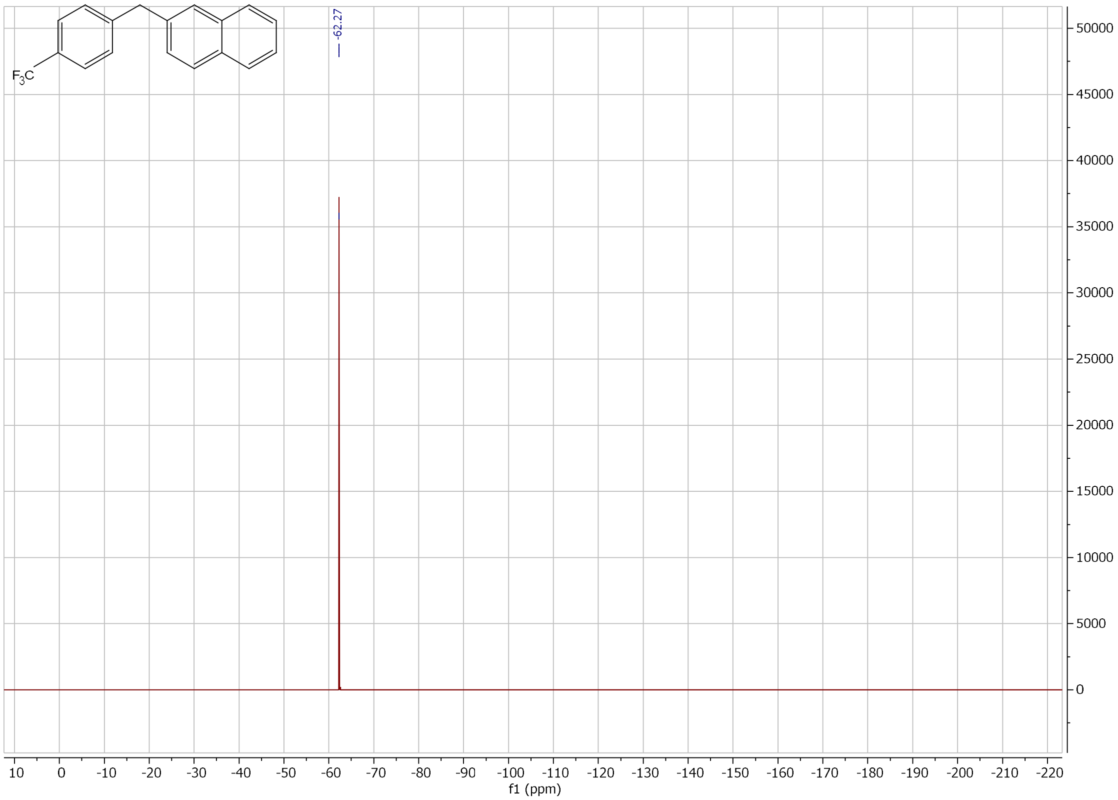

^{1}H NMR of 1-methoxy-4-(4-(trifluoromethyl)benzyl)benzene (**34**)

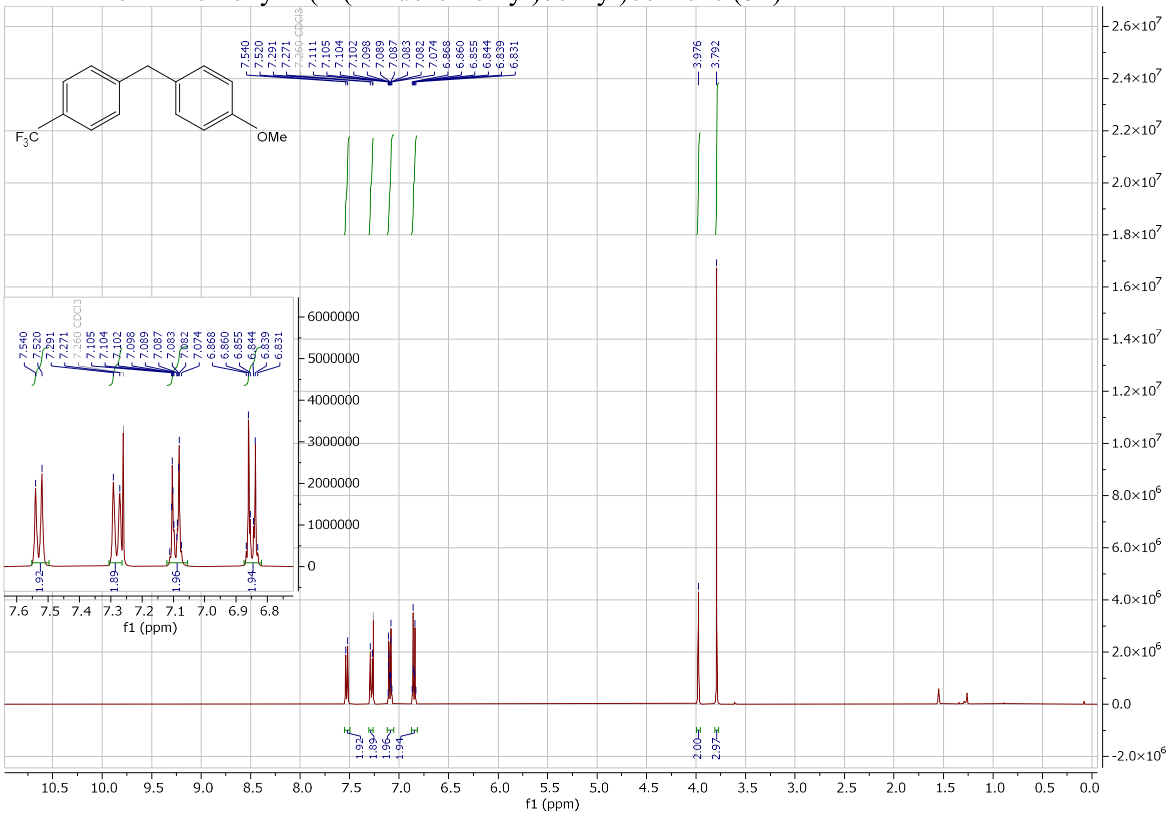

^{13}C NMR of 1-methoxy-4-(4-(trifluoromethyl)benzyl)benzene (**34**)

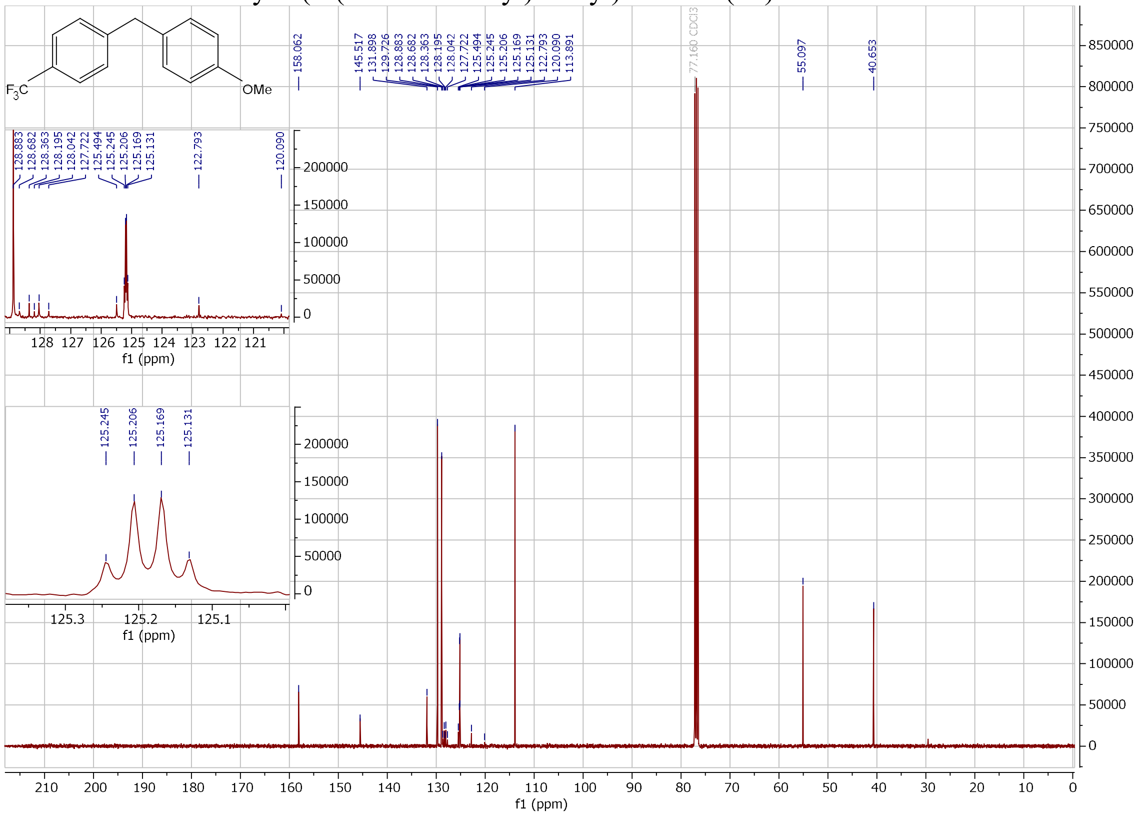

^{19}F NMR of 1-Methoxy-4-(4-(trifluoromethyl)benzyl)benzene (**34**)

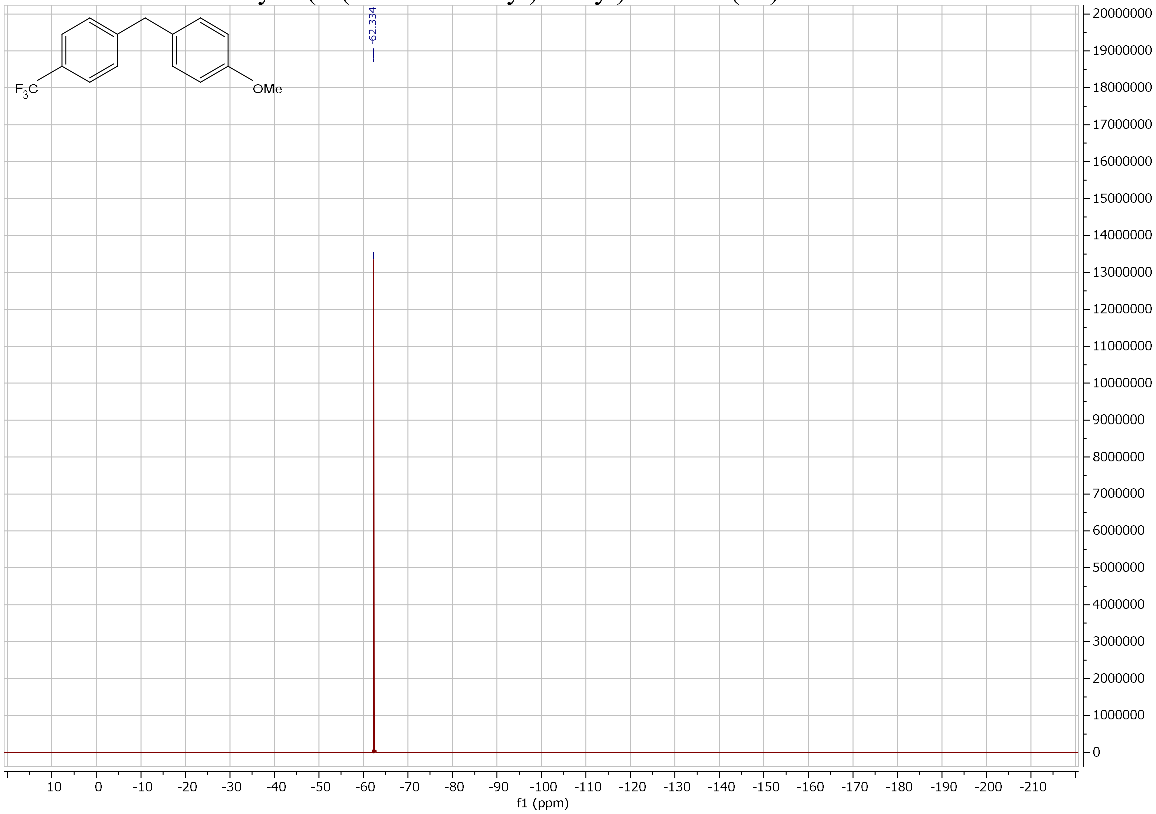

^{1}H NMR of 5-(4-(trifluoromethyl)benzyl)benzo[d][1,3]dioxole (**35**)

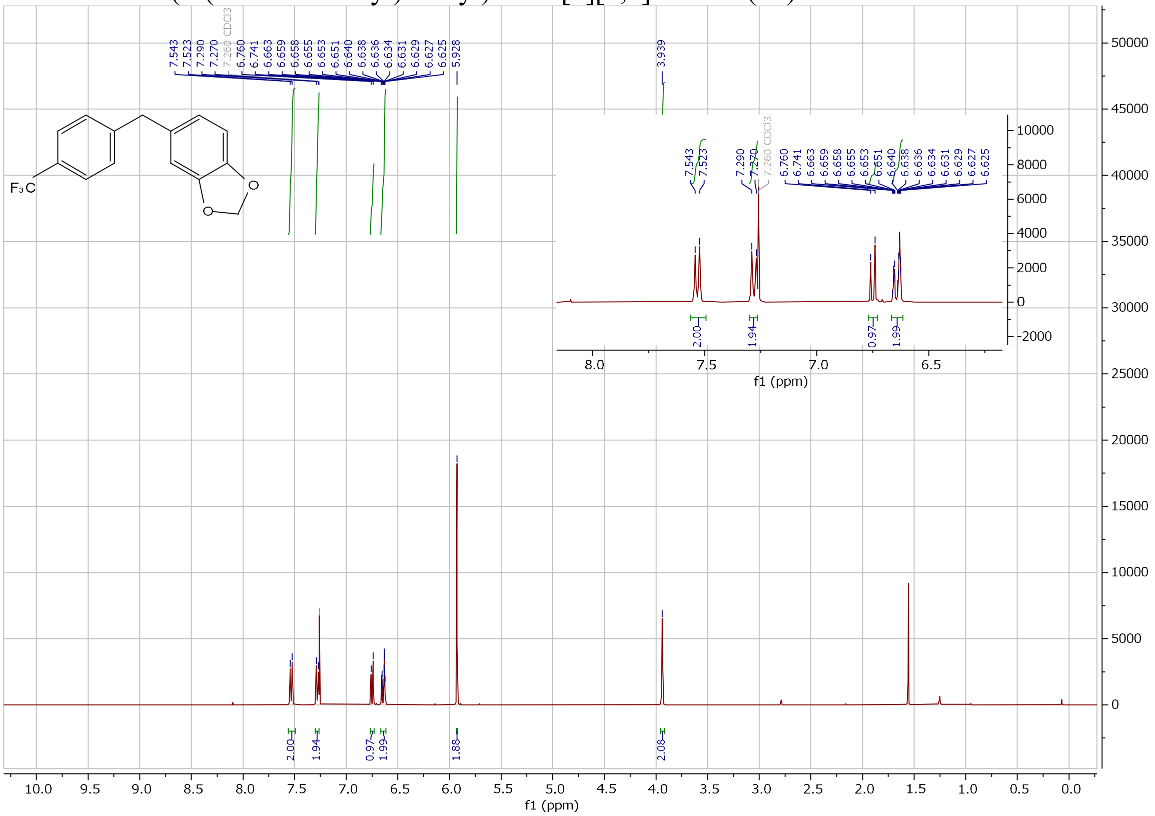

^{13}C NMR of 5-(4-(trifluoromethyl)benzyl)benzo[d][1,3]dioxole (**35**)

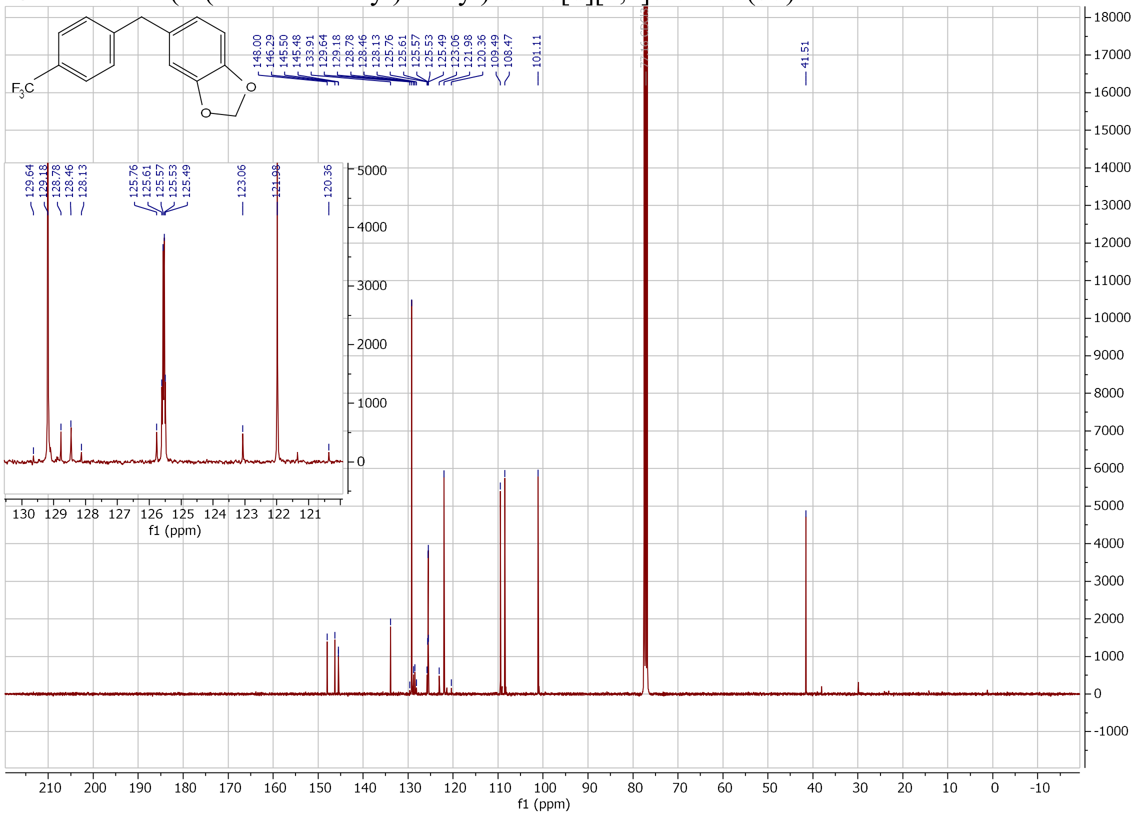

^{19}F NMR of 5-(4-(trifluoromethyl)benzyl)benzo[d][1,3]dioxole (**35**)

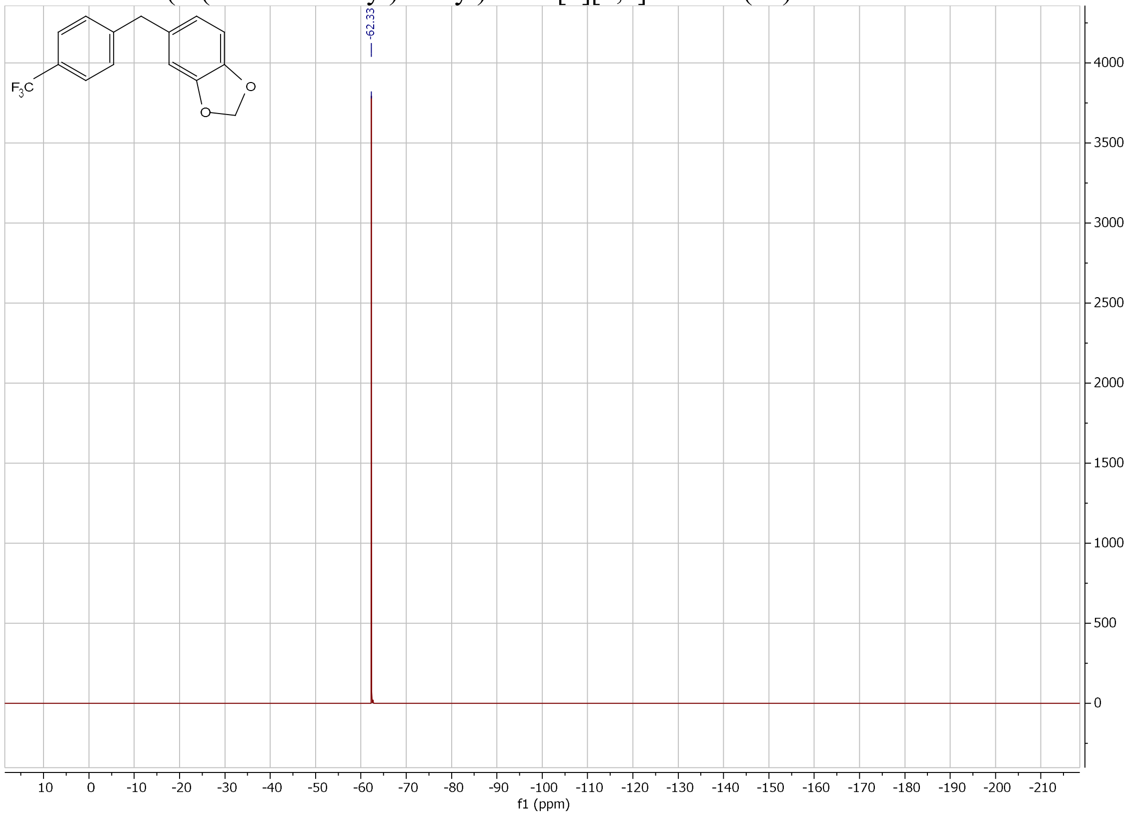

^{1}H NMR of 1,3-dimethoxy-5-(4-(trifluoromethyl)benzyl)benzene (**36**)

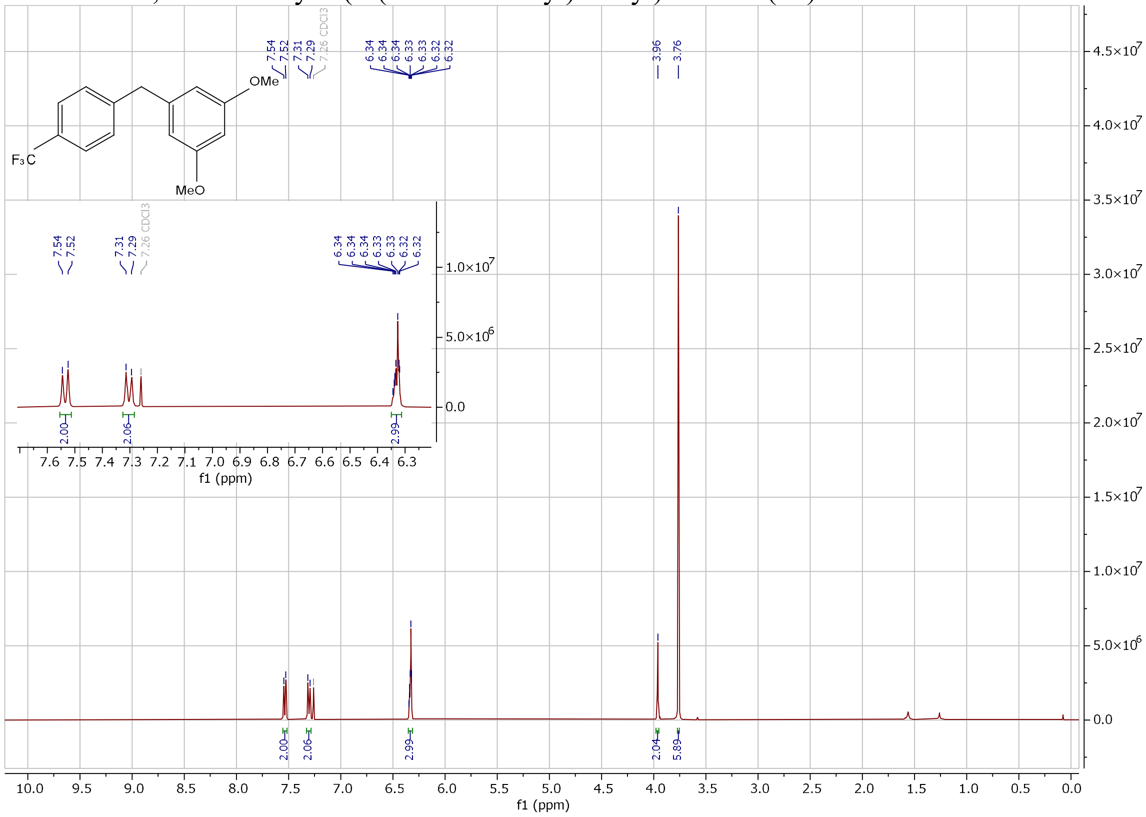

^{13}C NMR of 1,3-dimethoxy-5-(4-(trifluoromethyl)benzyl)benzene (**36**)

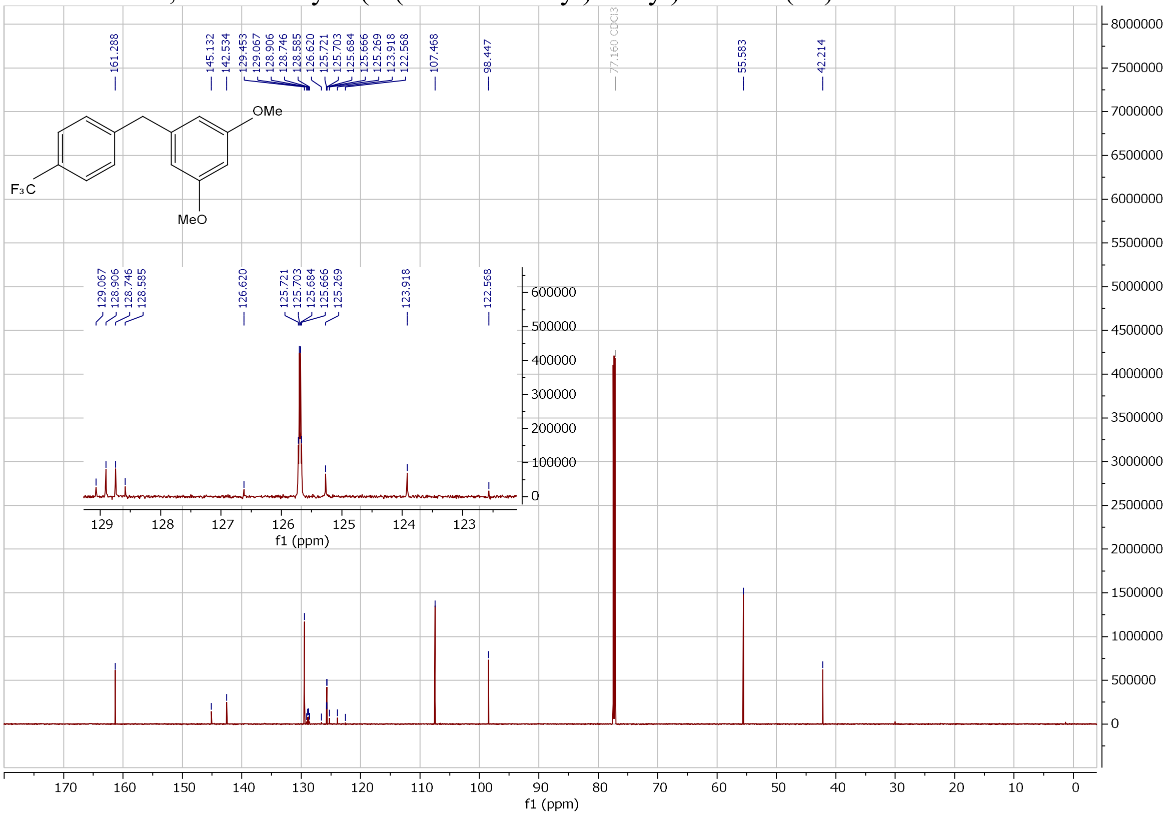

^{19}F NMR of 1,3-dimethoxy-5-(4-(trifluoromethyl)benzyl)benzene (**36**)

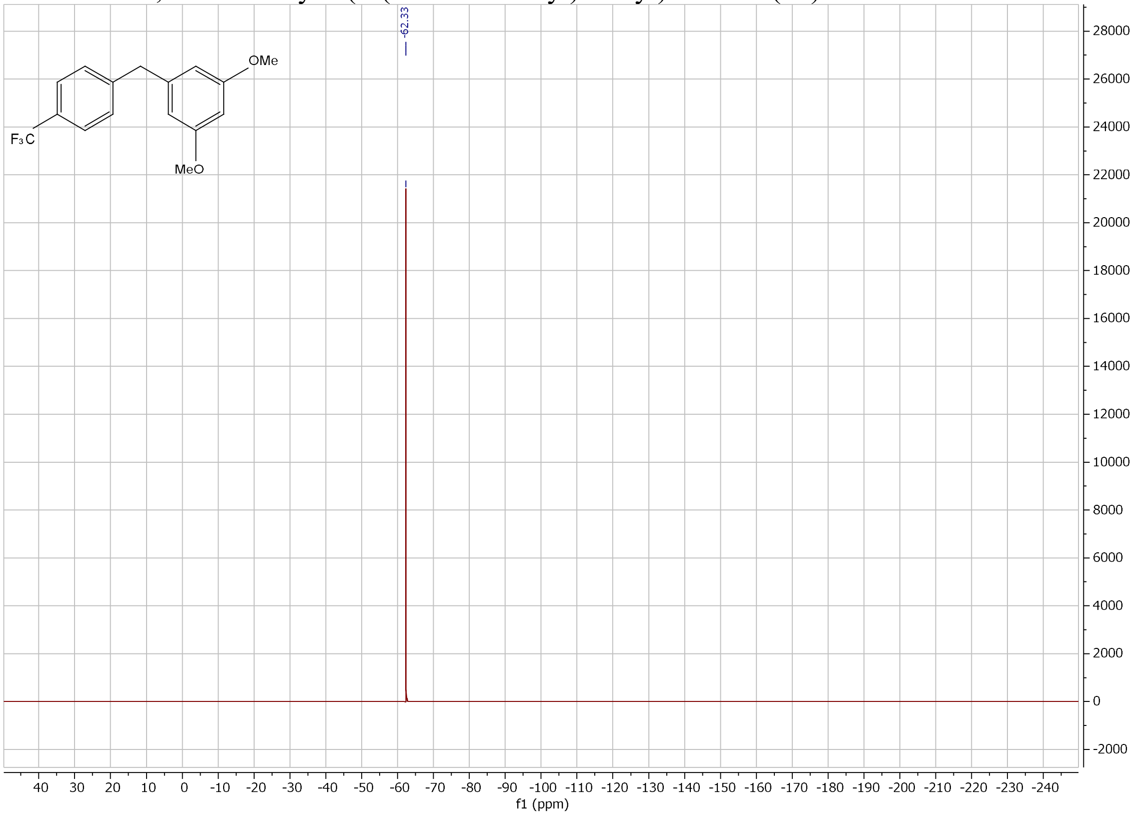

^{1}H NMR of 1-chloro-4-(4-(trifluoromethyl)benzyl)benzene (**37**)

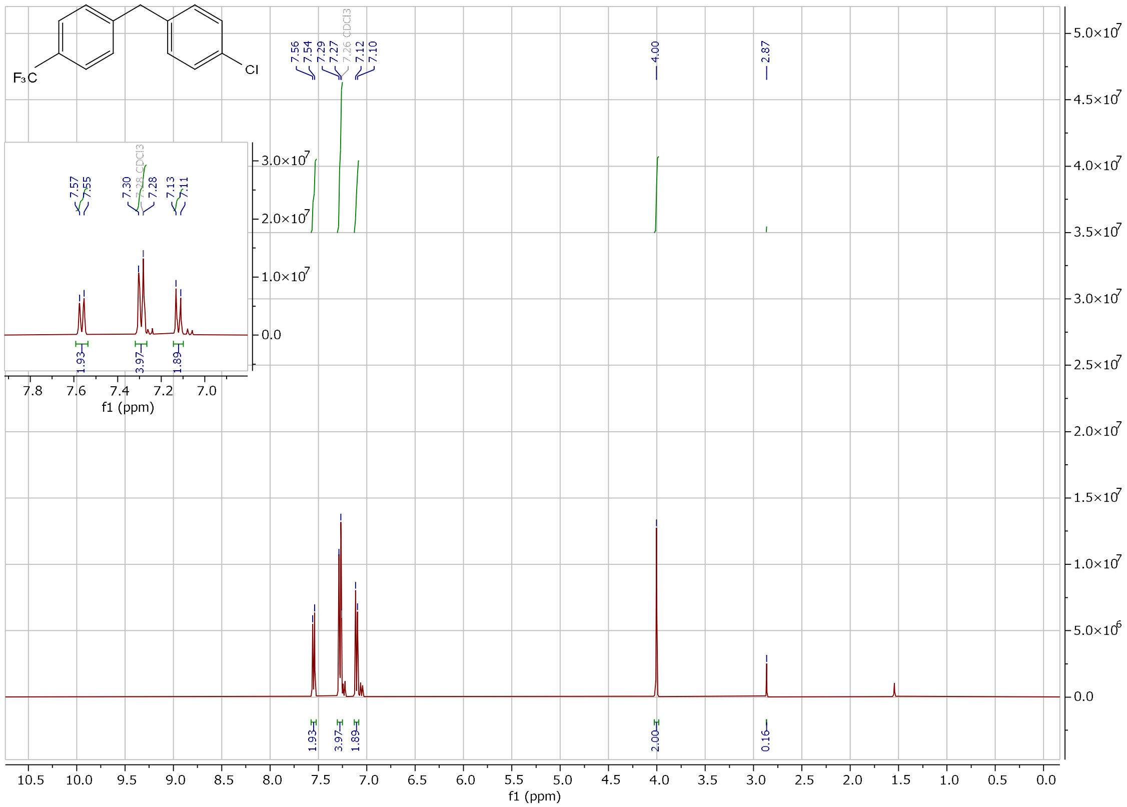

^{13}C NMR of 1-chloro-4-(4-(trifluoromethyl)benzyl)benzene (**37**)

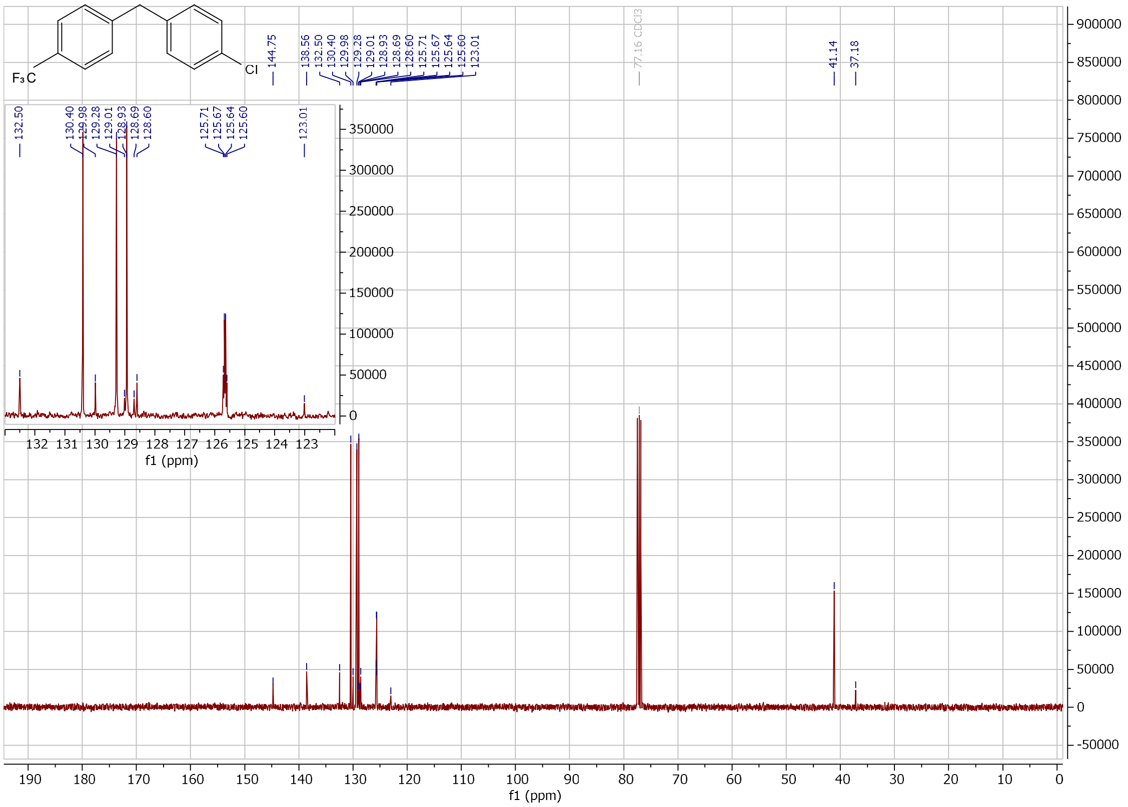

^{19}F NMR of 1-chloro-4-(4-(trifluoromethyl)benzyl)benzene (**37**)

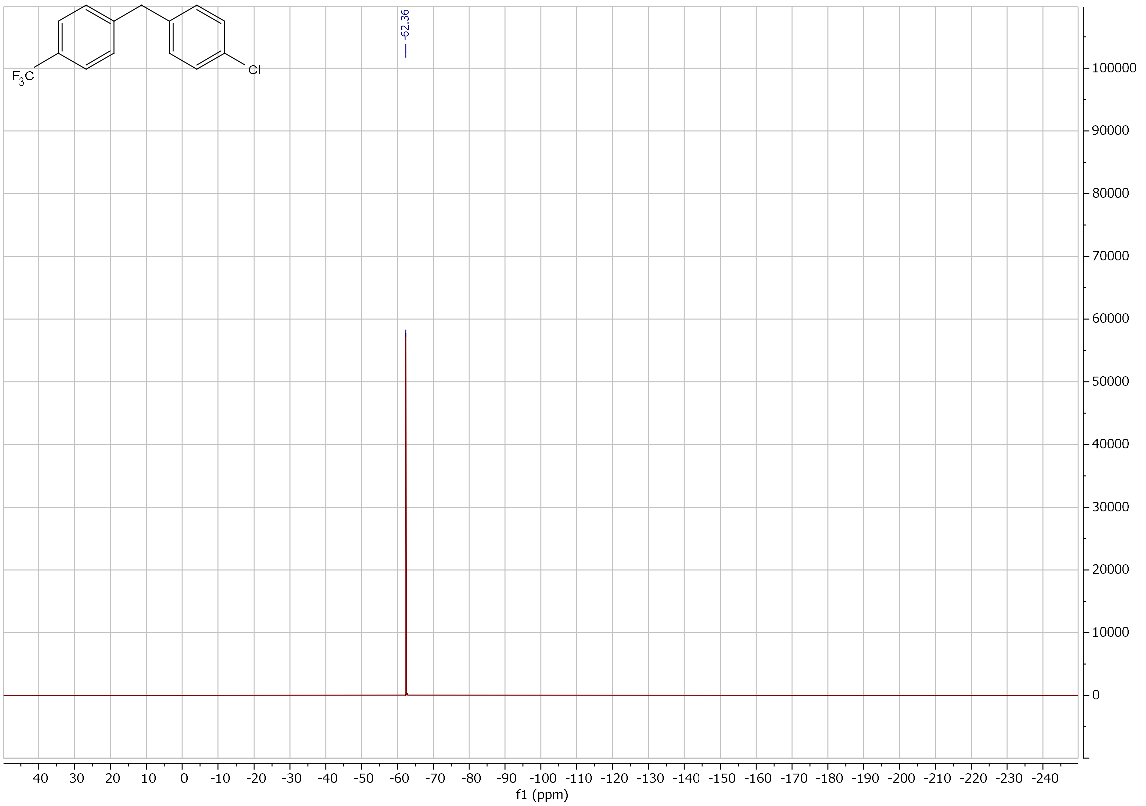

^{1}H NMR of 1-bromo-4-(4-(trifluoromethyl)benzyl)benzene (**38**)

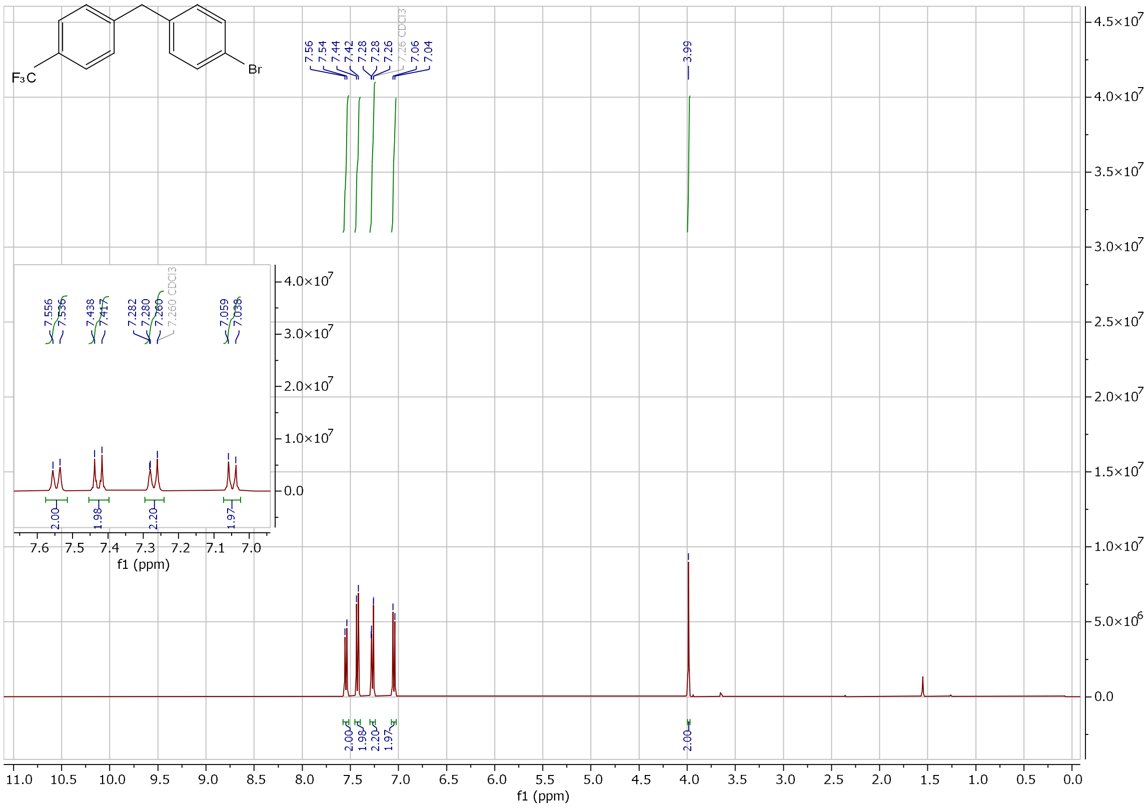

^{13}C NMR of 1-bromo-4-(4-(trifluoromethyl)benzyl)benzene (**38**)

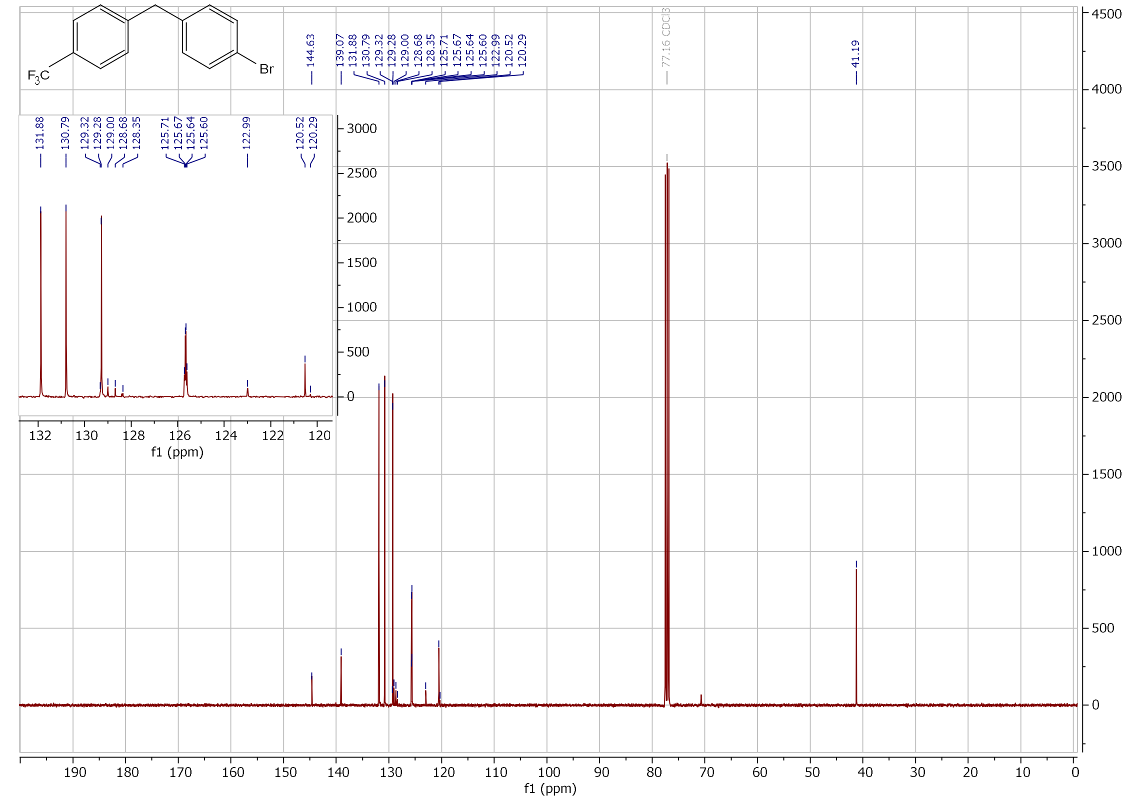

^{19}F NMR of 1-bromo-4-(4-(trifluoromethyl)benzyl)benzene (**38**)

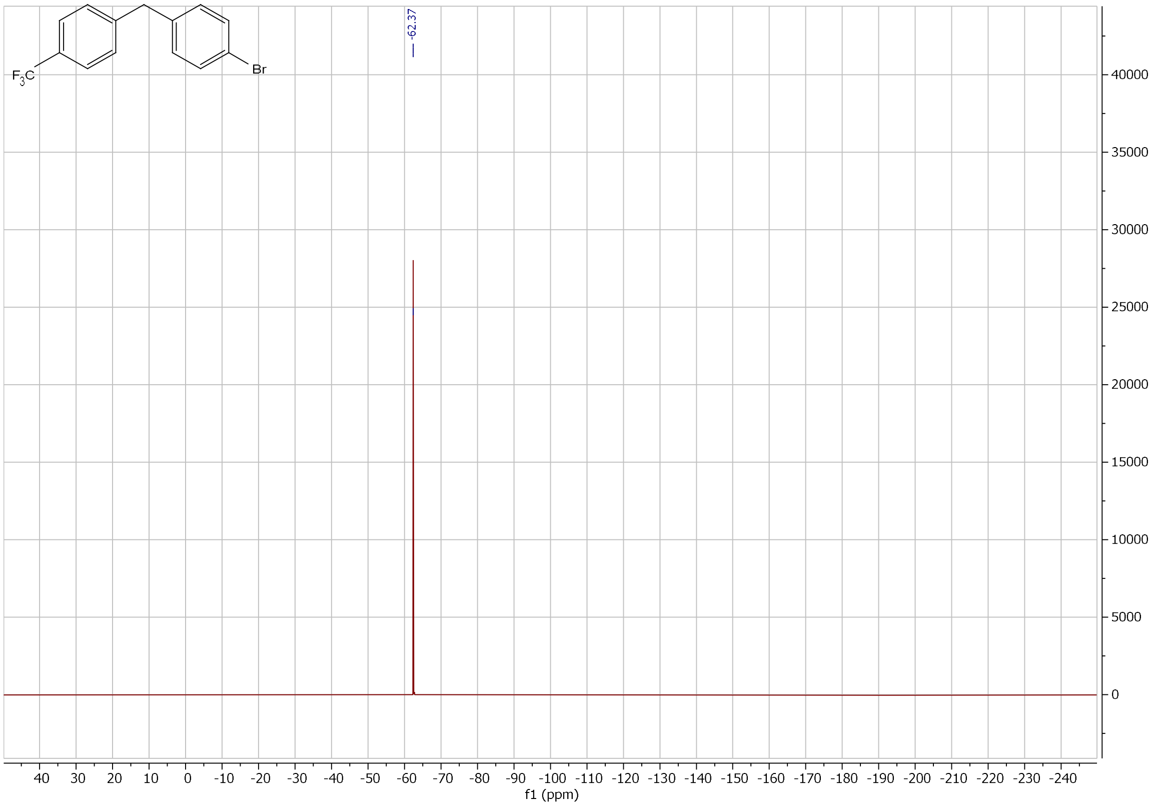

^{1}H NMR of 2,4-difluoro-1-(4-(trifluoromethyl)benzyl)benzene (**39**)

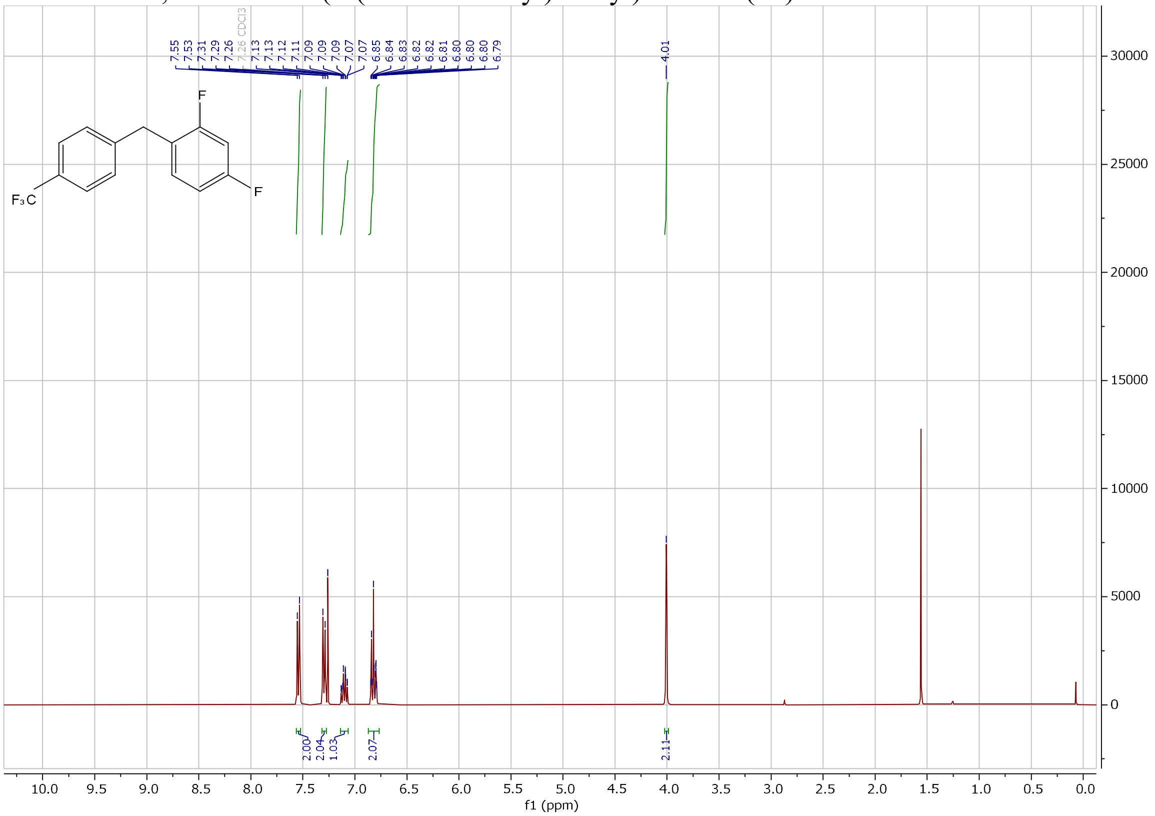

^{13}C NMR of 2,4-difluoro-1-(4-(trifluoromethyl)benzyl)benzene (**39**)

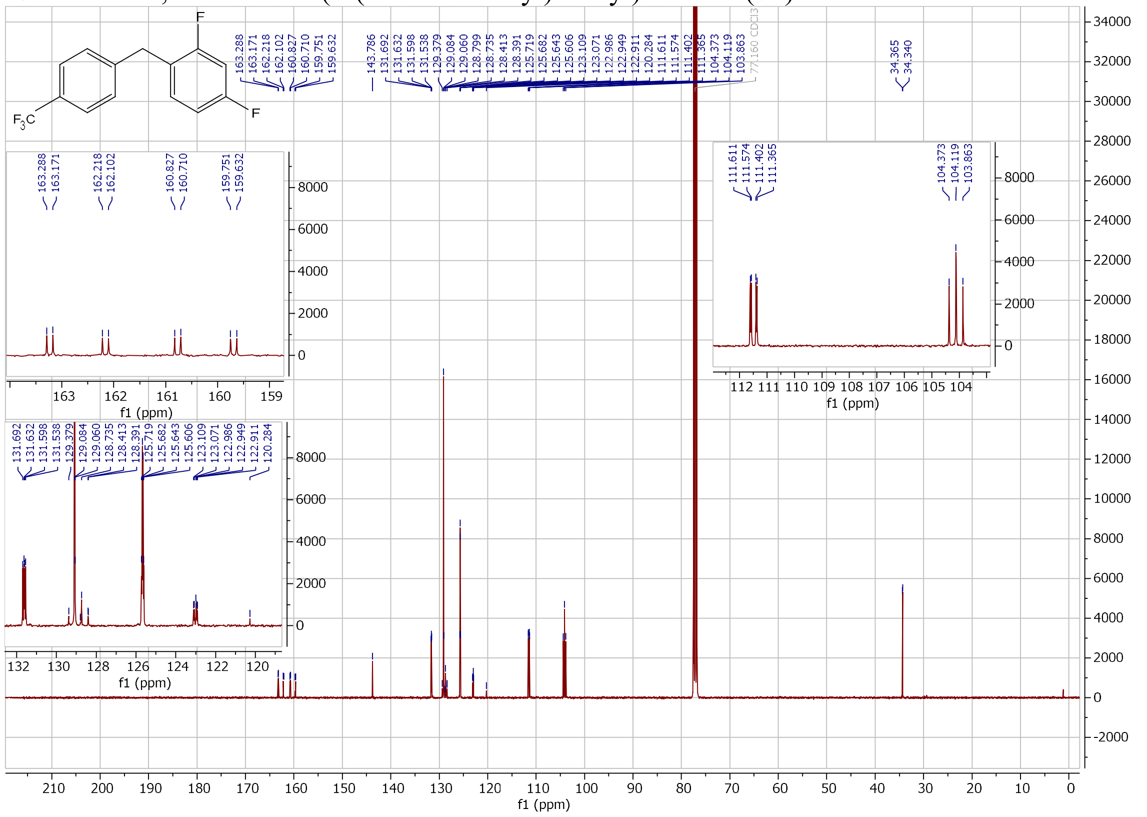

^{19}F NMR of 2,4-difluoro-1-(4-(trifluoromethyl)benzyl)benzene (**39**)

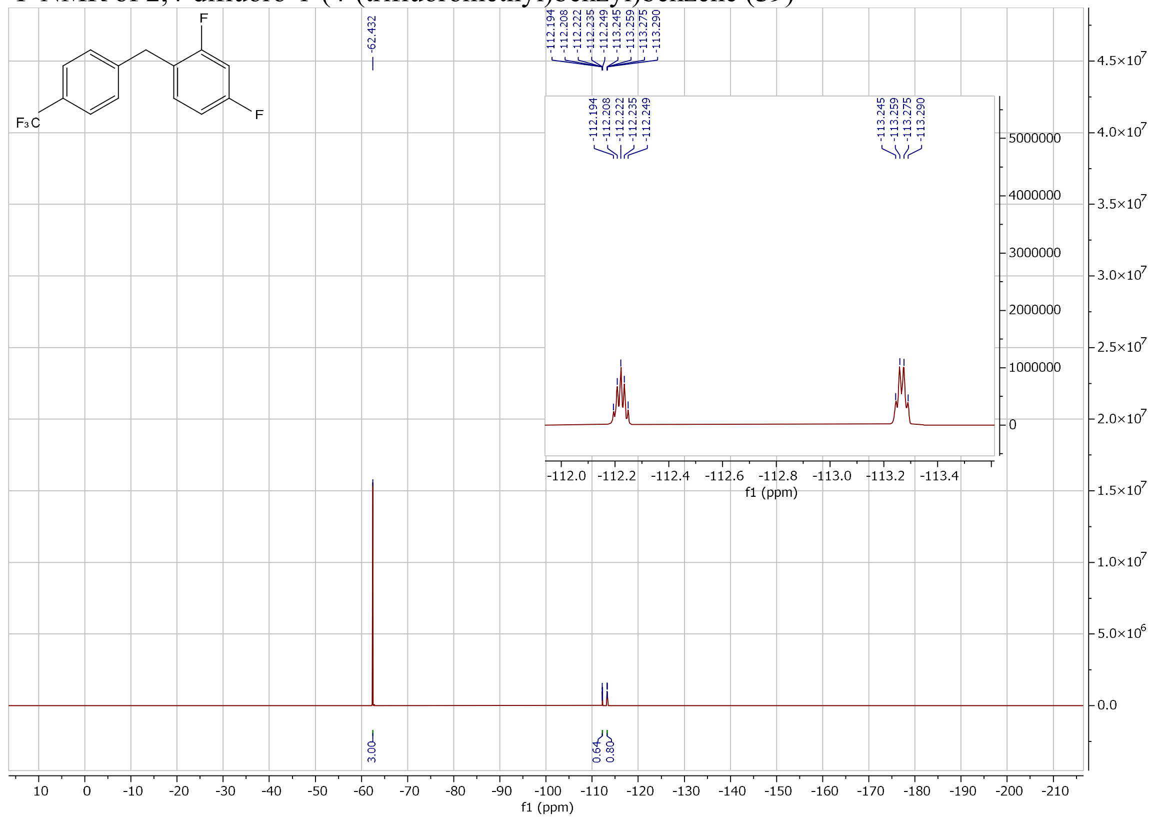

^{1}H NMR of 4-(4-(trifluoromethyl)benzyl)benzonitrile (**40**)

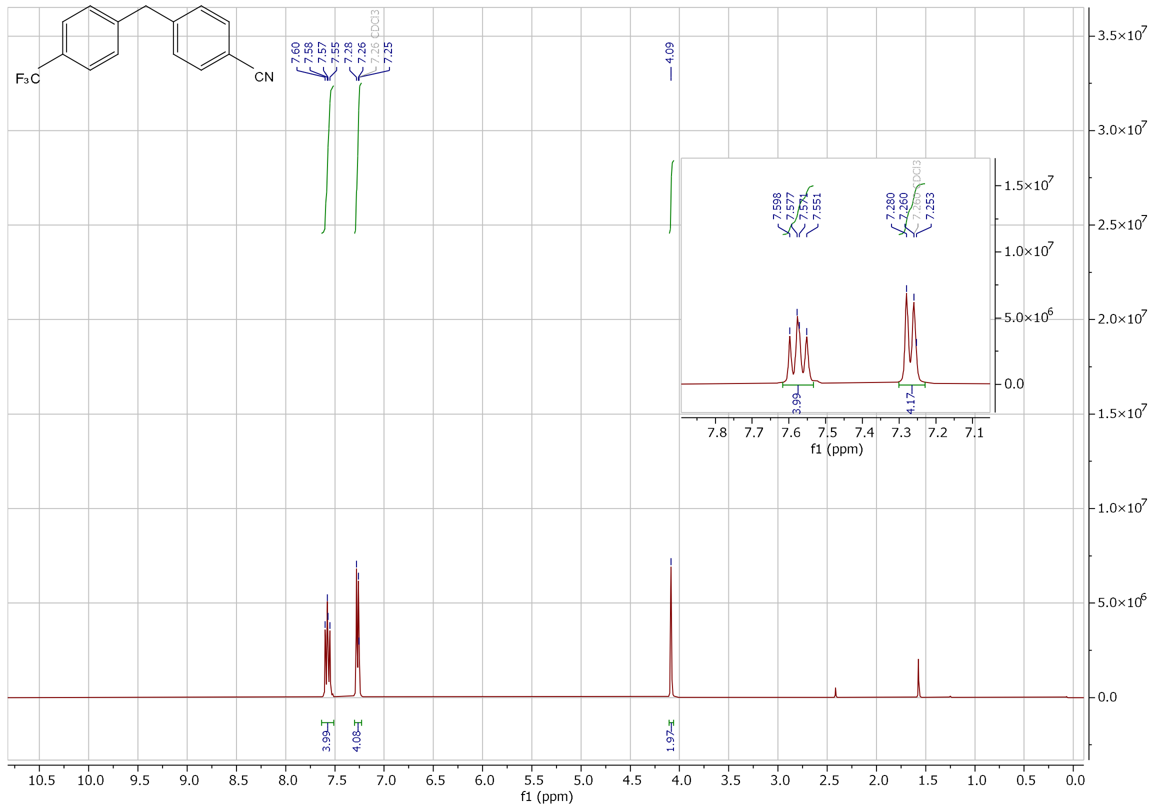

^{13}C NMR of 4-(4-(trifluoromethyl)benzyl)benzonitrile (**40**)

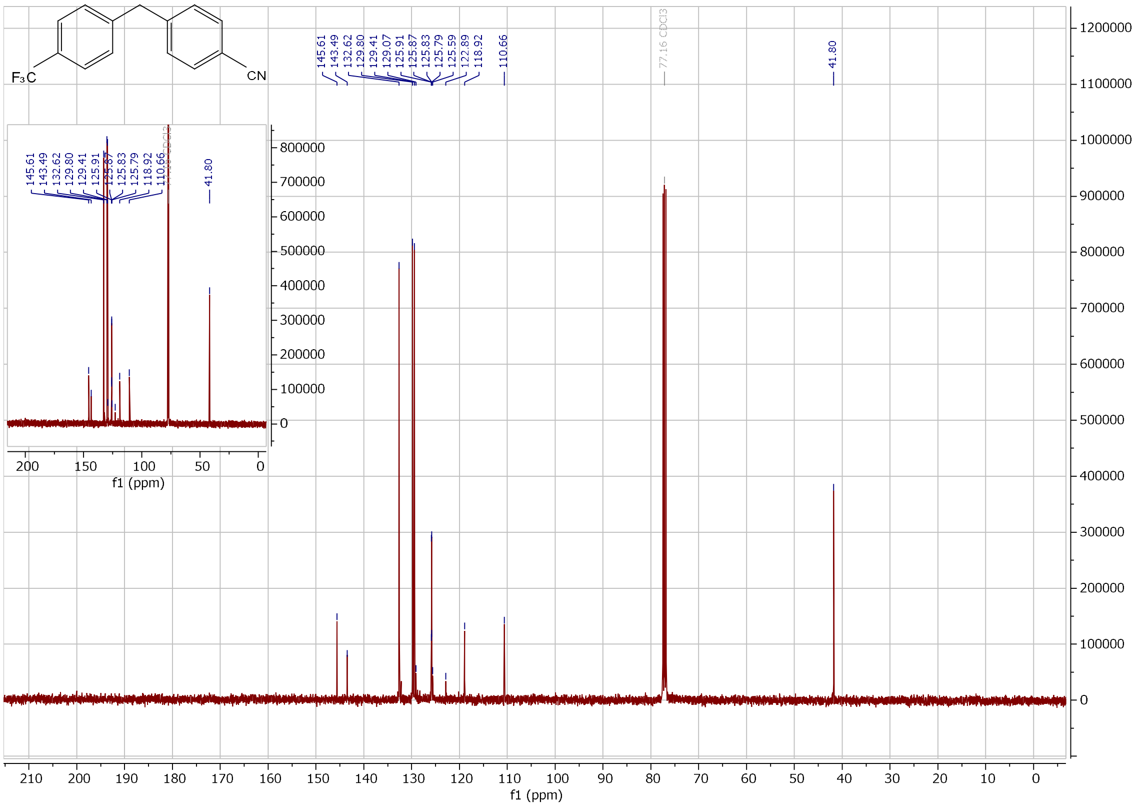

^{19}F NMR of 4-(4-(trifluoromethyl)benzyl)benzonitrile (**40**)

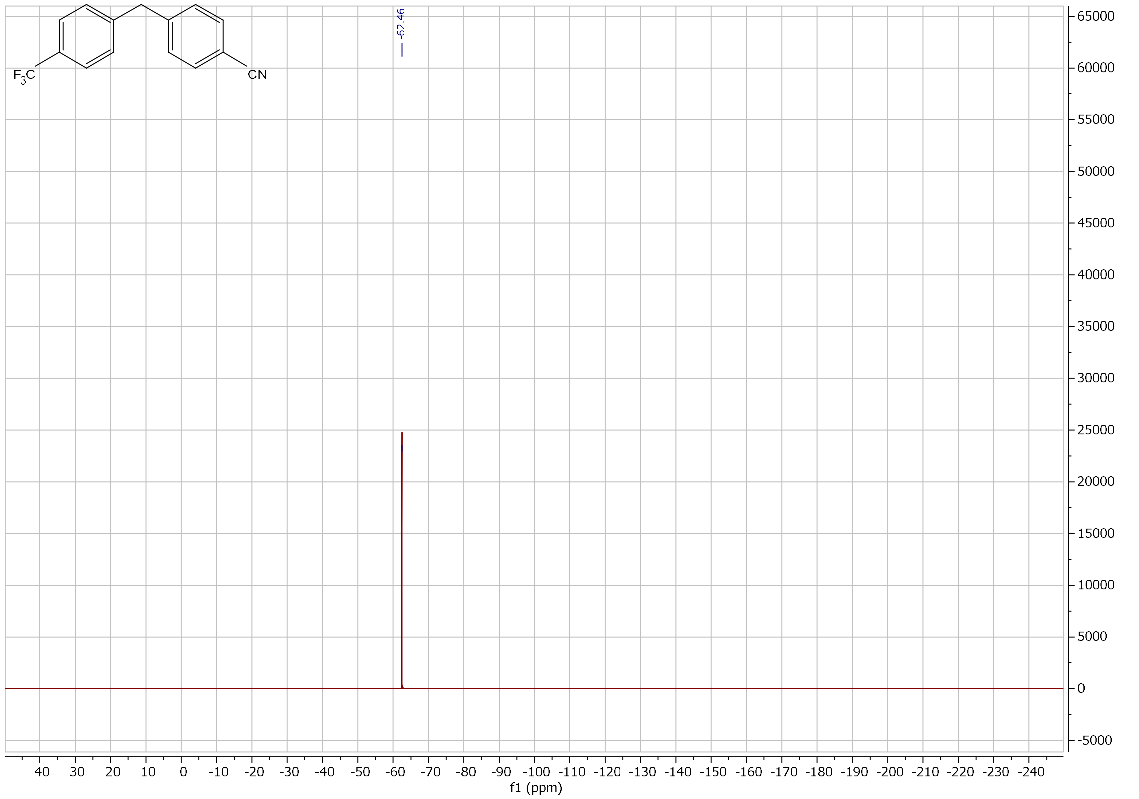

Optimized Geometries - Time-Dependent Density Functional Theory Studies

Optimized geometry of 4,4'-Czbpy (neutral, spin singlet). CAM-B3LYP-GD3/6-311+G(d,p).

1	C	1.520172	-0.171126	-0.016888
2	C	2.880046	-0.077229	-0.275571
3	C	3.43023	1.18275	-0.491525
4	C	2.59048	2.282586	-0.429882
5	H	1.045339	-1.121218	0.178306
6	H	2.988804	3.278952	-0.592829
7	C	-0.705765	0.926091	0.260059
8	C	-1.443809	2.095937	0.403829
9	C	-2.810121	2.000625	0.625449
10	H	-0.944135	3.051884	0.351569
11	C	-2.556735	-0.367769	0.571822
12	C	-3.383457	0.735199	0.70831
13	H	-2.972883	-1.368327	0.632346
14	C	0.76057	0.993588	0.002945
15	N	-1.250148	-0.288739	0.34788
16	N	1.285872	2.203929	-0.194581
17	N	-3.597088	3.158766	0.756095
18	C	-3.583539	4.25672	-0.109773
19	C	-4.543628	3.397336	1.755718
20	C	-2.843422	4.464832	-1.268878
21	C	-4.5281	5.197574	0.336442
22	C	-4.879546	2.62552	2.863108
23	C	-5.138019	4.6512	1.528666
24	C	-3.05222	5.646069	-1.961731
25	H	-2.129651	3.734612	-1.624735
26	C	-4.720591	6.377156	-0.376007
27	C	-5.843897	3.121135	3.725622
28	H	-4.400062	1.675692	3.056972
29	C	-6.104112	5.128726	2.408614

30	C	-3.976802	6.598344	-1.52175
31	H	-2.484994	5.832553	-2.865589
32	H	-5.44631	7.107766	-0.038731
33	C	-6.457787	4.357239	3.501866
34	H	-6.122928	2.53765	4.594678
35	H	-6.567103	6.093996	2.241011
36	H	-4.113115	7.511937	-2.086887
37	H	-7.208728	4.714394	4.19548
38	N	3.681352	-1.231873	-0.324596
39	C	3.393524	-2.395222	-1.045112
40	C	4.911737	-1.402556	0.315032
41	C	2.310399	-2.685243	-1.868128
42	C	4.446251	-3.310091	-0.868182
43	C	5.588872	-0.549264	1.180022
44	C	5.413102	-2.678495	0.002572
45	C	2.291319	-3.92074	-2.494346
46	H	1.508356	-1.976213	-2.020892
47	C	4.405196	-4.545282	-1.507439
48	C	6.793635	-0.987346	1.705529
49	H	5.188908	0.419459	1.446973
50	C	6.625102	-3.097188	0.542419
51	C	3.323309	-4.847203	-2.315677
52	H	1.456413	-4.171144	-3.13755
53	H	5.212271	-5.256337	-1.376131
54	C	7.314206	-2.245611	1.388255
55	H	7.339876	-0.34006	2.380966
56	H	7.018576	-4.078956	0.30682
57	H	3.275025	-5.804752	-2.81905
58	H	8.259317	-2.556307	1.815812
59	H	-4.447833	0.615786	0.857264
60	H	4.481019	1.298975	-0.71886

Optimized geometry of 4,4'-Czbpy (neutral, spin triplet). CAM-B3LYP-GD3/6-311+G(d,p).

1	C	1.535861	-0.168528	0.045044
2	C	2.878336	-0.057206	-0.215742
3	C	3.466602	1.219922	-0.401764
4	C	2.609667	2.309131	-0.308324
5	H	1.069591	-1.124164	0.235755
6	H	3.012603	3.308174	-0.459407
7	C	-0.651924	0.92265	0.361298
8	C	-1.437656	2.098707	0.467089
9	C	-2.788042	1.985557	0.682265
10	H	-0.947675	3.059964	0.412966
11	C	-2.533443	-0.384101	0.694213
12	C	-3.386919	0.705953	0.807389
13	H	-2.94988	-1.386329	0.767639
14	C	0.744018	1.006061	0.115832
15	N	-1.229305	-0.318947	0.490671
16	N	1.313996	2.245935	-0.055965
17	N	-3.589939	3.141838	0.76495
18	C	-3.559513	4.218642	-0.117371
19	C	-4.555653	3.392338	1.736747
20	C	-2.790963	4.396263	-1.264485
21	C	-4.519762	5.168558	0.284654
22	C	-4.890295	2.637772	2.857098
23	C	-5.158547	4.638111	1.47573
24	C	-2.982236	5.563616	-1.987461
25	H	-2.073467	3.652013	-1.579419
26	C	-4.6974	6.324781	-0.454647
27	C	-5.878196	3.13701	3.692366
28	H	-4.39015	1.703591	3.070151
29	C	-6.138635	5.120719	2.325836
30	C	-3.91755	6.52027	-1.590073

31	H	-2.393401	5.731913	-2.880439
32	H	-5.433858	7.06205	-0.158526
33	C	-6.501608	4.358194	3.43101
34	H	-6.163271	2.570064	4.569848
35	H	-6.6086	6.078823	2.139376
36	H	-4.042147	7.421358	-2.177552
37	H	-7.26732	4.72146	4.105061
38	N	3.677461	-1.21411	-0.31501
39	C	3.367714	-2.357447	-1.046962
40	C	4.916787	-1.399315	0.292891
41	C	2.261296	-2.61566	-1.851496
42	C	4.420452	-3.285189	-0.916189
43	C	5.597454	-0.562288	1.171853
44	C	5.414496	-2.670048	-0.05505
45	C	2.215207	-3.839502	-2.501178
46	H	1.46933	-1.889268	-1.965661
47	C	4.355879	-4.49905	-1.577497
48	C	6.815321	-1.006612	1.664032
49	H	5.184764	0.392651	1.46511
50	C	6.629755	-3.096514	0.452342
51	C	3.242999	-4.773978	-2.365845
52	H	1.363241	-4.07055	-3.128259
53	H	5.15932	-5.220228	-1.487979
54	C	7.331902	-2.253152	1.30694
55	H	7.370427	-0.37531	2.346665
56	H	7.023249	-4.072767	0.196042
57	H	3.175853	-5.720695	-2.887221
58	H	8.284463	-2.571805	1.711549
59	H	-4.454513	0.584498	0.915148
60	H	4.506579	1.334863	-0.668904

Optimized geometry of 5,5'-Czbpy (neutral, spin singlet). CAM-B3LYP-GD3/6-311+G(d,p).

1	C	-1.403769	-0.002768	-1.26076
2	C	-2.785179	0.005125	-1.276223
3	C	-3.471365	-0.010269	-0.068032
4	C	-2.726419	-0.028951	1.107453
5	H	-0.825018	0.009065	-2.173229
6	H	-3.335308	0.029571	-2.208837
7	H	-3.234858	-0.051758	2.065935
8	C	0.742694	-0.009587	0.033366
9	C	1.403769	-0.002767	1.260761
10	C	2.785179	0.005126	1.276223
11	H	0.825018	0.009066	2.173229
12	C	2.726419	-0.028951	-1.107453
13	C	3.471365	-0.010269	0.068032
14	H	3.335308	0.029573	2.208837
15	H	3.234858	-0.051758	-2.065934
16	C	-0.742694	-0.009587	-0.033366
17	N	1.402273	-0.018297	-1.126869
18	N	-1.402273	-0.018297	1.12687
19	C	5.71649	-0.918809	0.665739
20	C	5.673607	0.916452	-0.656904
21	C	5.38625	-2.028351	1.436242
22	C	7.054616	-0.579457	0.395446
23	C	5.293745	2.022723	-1.408987
24	C	7.027143	0.594397	-0.449535
25	C	6.425889	-2.787244	1.94825
26	H	4.355092	-2.29703	1.624706
27	C	8.081845	-1.357538	0.920902
28	C	6.298633	2.795362	-1.967338
29	H	4.251597	2.276809	-1.551473
30	C	8.018911	1.385821	-1.021365

31	C	7.762048	-2.456737	1.699025
32	H	6.196658	-3.657021	2.552017
33	H	9.116621	-1.106619	0.719502
34	C	7.649208	2.481746	-1.781553
35	H	6.03024	3.662285	-2.558933
36	H	9.065019	1.14755	-0.869152
37	H	8.550402	-3.071195	2.115589
38	H	8.409285	3.106517	-2.233806
39	C	-5.71649	-0.91881	-0.665739
40	C	-5.673606	0.916452	0.656905
41	C	-5.38625	-2.028352	-1.436242
42	C	-7.054616	-0.579457	-0.395447
43	C	-5.293745	2.022723	1.408987
44	C	-7.027143	0.594397	0.449534
45	C	-6.425889	-2.787244	-1.94825
46	H	-4.355092	-2.297032	-1.624705
47	C	-8.081845	-1.357537	-0.920903
48	C	-6.298633	2.795362	1.967338
49	H	-4.251597	2.276807	1.551474
50	C	-8.018911	1.385822	1.021364
51	C	-7.762048	-2.456736	-1.699026
52	H	-6.196658	-3.657022	-2.552017
53	H	-9.116621	-1.106618	-0.719504
54	C	-7.649208	2.481747	1.781551
55	H	-6.03024	3.662286	2.558933
56	H	-9.065019	1.147552	0.869149
57	H	-8.550403	-3.071194	-2.11559
58	H	-8.409285	3.106519	2.233804
59	N	-4.880227	-0.007371	-0.022793
60	N	4.880227	-0.00737	0.022794

Optimized geometry of 5,5'-Czbpy (neutral, spin triplet). CAM-B3LYP-GD3/6-311+G(d,p).

1	C	-1.398767	-0.136237	-1.297575
2	C	-2.753098	-0.097786	-1.312673
3	C	-3.454106	-0.051373	-0.081078
4	C	-2.691343	-0.08086	1.122937
5	H	-0.81618	-0.144811	-2.206817
6	H	-3.302948	-0.069865	-2.244699
7	H	-3.225942	-0.09774	2.069764
8	C	0.68702	-0.134895	0.030173
9	C	1.398762	-0.136061	1.297582
10	C	2.753101	-0.097609	1.312671
11	H	0.81618	-0.144534	2.206827
12	C	2.691344	-0.080976	-1.122924
13	C	3.454102	-0.051356	0.081079
14	H	3.302953	-0.069582	2.244694
15	H	3.225937	-0.097953	-2.069752
16	C	-0.687019	-0.134912	-0.030157
17	N	1.399421	-0.126621	-1.174992
18	N	-1.399428	-0.126509	1.175015
19	C	5.722521	-0.827335	0.752673
20	C	5.613124	0.864666	-0.751553
21	C	5.435975	-1.878381	1.616432
22	C	7.045782	-0.477022	0.433556
23	C	5.197205	1.894436	-1.587977
24	C	6.97594	0.602941	-0.528444
25	C	6.505041	-2.552591	2.184967
26	H	4.417695	-2.1728	1.830497
27	C	8.10288	-1.169156	1.013161
28	C	6.176113	2.636485	-2.229479
29	H	4.149145	2.122138	-1.728038
30	C	7.941083	1.362369	-1.1797

31	C	7.826325	-2.20118	1.894594
32	H	6.309243	-3.373722	2.863766
33	H	9.12659	-0.908324	0.772218
34	C	7.535008	2.372857	-2.035615
35	H	5.878621	3.442133	-2.88958
36	H	8.99432	1.169805	-1.013347
37	H	8.638389	-2.749234	2.355862
38	H	8.274238	2.972592	-2.551775
39	C	-5.722526	-0.827396	-0.75261
40	C	-5.613118	0.864728	0.751482
41	C	-5.435988	-1.87852	-1.616276
42	C	-7.045784	-0.477049	-0.433518
43	C	-5.197192	1.894568	1.587815
44	C	-6.975934	0.602992	0.528396
45	C	-6.50506	-2.552769	-2.184753
46	H	-4.417711	-2.172967	-1.830314
47	C	-8.102888	-1.169225	-1.013062
48	C	-6.176096	2.636675	2.229258
49	H	-4.149131	2.122283	1.72785
50	C	-7.941073	1.362478	1.179589
51	C	-7.82634	-2.201325	-1.894409
52	H	-6.309268	-3.373961	-2.863482
53	H	-9.126595	-0.908367	-0.772138
54	C	-7.534991	2.373035	2.03542
55	H	-5.878598	3.442378	2.889289
56	H	-8.994311	1.169906	1.013252
57	H	-8.638409	-2.749412	-2.355629
58	H	-8.274217	2.972817	2.551531
59	N	-4.846965	-0.009586	-0.028442
60	N	4.846969	-0.00958	0.028439

Optimized geometry of Ni(5,5'-Czbpy)Cl (neutral, spin doublet). CAM-B3LYP-GD3/6-311+G(d,p).

1	C	1.515548	-2.117075	0.063196
2	C	2.89173	-1.980877	0.052046
3	C	3.4535	-0.714972	-0.084273
4	C	2.59105	0.366767	-0.22563
5	H	1.070236	-3.092439	0.201852
6	H	3.533606	-2.842307	0.18287
7	H	2.968911	1.37378	-0.341893
8	C	-0.7546	-0.980856	0.037142
9	C	-1.555396	-2.108889	-0.077443
10	C	-2.930891	-1.966015	-0.065057
11	H	-1.114892	-3.085175	-0.224704
12	C	-2.618653	0.377578	0.2333
13	C	-3.48642	-0.698608	0.082421
14	H	-3.577001	-2.823086	-0.203458
15	H	-2.991546	1.385374	0.358464
16	C	0.720322	-0.984144	-0.041422
17	N	-1.303333	0.231165	0.196417
18	N	1.275023	0.226517	-0.190009
19	Ni	-0.010335	1.778919	0.010056
20	Cl	0.254009	3.973757	-0.019046
21	C	-5.838173	-1.210335	0.7333
22	C	-5.512465	0.46863	-0.766907
23	C	-5.68353	-2.220451	1.675098
24	C	-7.106984	-0.724498	0.37428
25	C	-4.971606	1.385311	-1.661909
26	C	-6.898791	0.34283	-0.581823
27	C	-6.83124	-2.757998	2.236799
28	H	-4.705455	-2.570222	1.978459
29	C	-8.244467	-1.278241	0.951901

30	C	-5.856603	2.196544	-2.353994
31	H	-3.905491	1.47933	-1.828317
32	C	-7.766709	1.168548	-1.289484
33	C	-8.100894	-2.29826	1.877378
34	H	-6.738646	-3.545962	2.974238
35	H	-9.227894	-0.910763	0.684125
36	C	-7.238794	2.096372	-2.170146
37	H	-5.460708	2.923711	-3.052022
38	H	-8.838194	1.081607	-1.154087
39	H	-8.977528	-2.739191	2.335048
40	H	-7.899571	2.749205	-2.726612
41	C	5.802738	-1.23252	-0.739638
42	C	5.485218	0.434792	0.775247
43	C	5.643174	-2.233549	-1.690275
44	C	7.073905	-0.756108	-0.37635
45	C	4.948823	1.346242	1.678241
46	C	6.870917	0.303798	0.589085
47	C	6.788254	-2.771785	-2.256664
48	H	4.663405	-2.575827	-1.996703
49	C	8.20868	-1.310348	-0.958801
50	C	5.837761	2.147007	2.377428
51	H	3.883171	1.444048	1.845438
52	C	7.742849	1.11899	1.303979
53	C	8.060136	-2.321478	-1.893205
54	H	6.691822	-3.552776	-3.000996
55	H	9.193889	-0.950081	-0.6878
56	C	7.219454	2.041643	2.192735
57	H	5.445409	2.869969	3.081797
58	H	8.8139	1.027967	1.16784
59	H	8.934613	-2.762681	-2.354723
60	H	7.883403	2.686312	2.754915

61	N	4.834412	-0.505082	-0.040779
62	N	-4.866284	-0.481552	0.04082

Optimized geometry of Ni(4,4'-Czbpy)Cl (neutral, spin doublet). CAM-B3LYP-GD3/6-311+G(d,p).

1	C	1.487872	2.121835	-0.315706
2	C	2.85455	2.014248	-0.550865
3	C	3.415948	0.742014	-0.624909
4	C	2.593123	-0.356394	-0.469581
5	H	1.028794	3.099061	-0.286536
6	H	2.982776	-1.365658	-0.51932
7	C	-0.720672	0.965263	0.107361
8	C	-1.475351	2.112245	0.286882
9	C	-2.841545	1.996359	0.520921
10	H	-1.020009	3.091516	0.27052
11	C	-2.571158	-0.37199	0.40819
12	C	-3.398097	0.721142	0.578239
13	H	-2.956967	-1.383297	0.444628
14	C	0.737527	0.969727	-0.151491
15	N	-1.262115	-0.262302	0.17421
16	N	1.283624	-0.254786	-0.234465
17	Ni	0.013611	-1.777744	-0.040452
18	N	-3.630302	3.140165	0.696011
19	C	-3.605698	4.283131	-0.1134
20	C	-4.555467	3.349089	1.727346
21	C	-2.89079	4.529947	-1.28021
22	C	-4.517345	5.220372	0.400814
23	C	-4.888257	2.530806	2.800764
24	C	-5.120324	4.626381	1.5747
25	C	-3.082742	5.75085	-1.907229
26	H	-2.218392	3.794976	-1.701762

27	C	-4.694824	6.439066	-0.245906
28	C	-5.824776	3.005485	3.705247
29	H	-4.430364	1.561151	2.940973
30	C	-6.058542	5.082268	2.495019
31	C	-3.969667	6.701793	-1.39521
32	H	-2.536352	5.967104	-2.817113
33	H	-5.395846	7.167793	0.143203
34	C	-6.411668	4.264925	3.554596
35	H	-6.102796	2.385481	4.548728
36	H	-6.500279	6.065431	2.38476
37	H	-4.09542	7.646274	-1.909417
38	H	-7.141696	4.604014	4.278728
39	N	3.638997	3.163269	-0.710448
40	C	3.609915	4.295179	0.11423
41	C	4.564148	3.389195	-1.738199
42	C	2.893392	4.523864	1.283736
43	C	4.518628	5.242389	-0.386742
44	C	4.900514	2.586539	-2.822259
45	C	5.124458	4.666276	-1.568044
46	C	3.080657	5.736934	1.927165
47	H	2.223298	3.78098	1.694951
48	C	4.691409	6.452938	0.276339
49	C	5.836011	3.076536	-3.7196
50	H	4.44608	1.61728	-2.97582
51	C	6.061719	5.137707	-2.481479
52	C	3.964576	6.697737	1.428537
53	H	2.532912	5.939085	2.839474
54	H	5.390132	7.189247	-0.102499
55	C	6.418426	4.335865	-3.551648
56	H	6.116763	2.468859	-4.571109
57	H	6.49996	6.120828	-2.357726

58	H	4.086671	7.635694	1.955411
59	H	7.147779	4.687152	-4.270627
60	H	-4.459077	0.586085	0.734123
61	H	4.477468	0.613041	-0.782248
62	Cl	0.017325	-3.924475	-0.055232

Optimized geometry of Ni(5,5'-Czbpy)Cl_2 (neutral, spin triplet). CAM-B3LYP-GD3/6-311+G(d,p).

1	C	1.535932	2.169685	-0.059574
2	C	2.911659	2.030149	-0.038206
3	C	3.469306	0.763477	0.107589
4	C	2.603197	-0.315502	0.247657
5	H	1.094032	3.145522	-0.205618
6	H	3.556578	2.889412	-0.168317
7	H	2.977725	-1.32292	0.370976
8	C	-0.737168	1.039185	-0.044408
9	C	-1.535929	2.16968	0.059626
10	C	-2.911656	2.03014	0.038251
11	H	-1.094032	3.145517	0.205679
12	C	-2.603187	-0.315506	-0.247632
13	C	-3.4693	0.763467	-0.107546
14	H	-3.556578	2.8894	0.16837
15	H	-2.977708	-1.322923	-0.370977
16	C	0.737174	1.039187	0.04445
17	N	-1.287795	-0.172171	-0.202197
18	N	1.287804	-0.17217	0.202222
19	Ni	0.000008	-1.722268	-0.000013
20	Cl	0.823882	-2.456893	-1.915635
21	Cl	-0.823884	-2.457024	1.915548
22	C	-5.81517	1.278112	-0.777089
23	C	-5.504128	-0.395027	0.732709

24	C	-5.651439	2.283686	-1.722205
25	C	-7.087663	0.796986	-0.424821
26	C	-4.971817	-1.309086	1.635484
27	C	-6.88881	-0.26663	0.537388
28	C	-6.793856	2.821578	-2.294269
29	H	-4.670401	2.629708	-2.020253
30	C	-8.219702	1.350979	-1.012804
31	C	-5.863643	-2.115081	2.324917
32	H	-3.907127	-1.404996	1.809752
33	C	-7.763703	-1.087084	1.242579
34	C	-8.06713	2.366555	-1.94172
35	H	-6.694153	3.606056	-3.034493
36	H	-9.205888	0.987106	-0.7503
37	C	-7.244263	-2.012317	2.130972
38	H	-5.474444	-2.84014	3.028879
39	H	-8.833996	-0.998102	1.099314
40	H	-8.939446	2.807623	-2.407438
41	H	-7.910533	-2.661064	2.685664
42	C	5.815195	1.278121	0.777085
43	C	5.504102	-0.39506	-0.732654
44	C	5.651495	2.283717	1.722183
45	C	7.087676	0.796985	0.424787
46	C	4.971753	-1.309155	-1.635368
47	C	6.88879	-0.26666	-0.537385
48	C	6.793932	2.821621	2.294197
49	H	4.670468	2.629753	2.020249
50	C	8.219735	1.350989	1.01272
51	C	5.863551	-2.115178	-2.324804
52	H	3.907053	-1.405072	-1.809581
53	C	7.763656	-1.087137	-1.242582
54	C	8.067194	2.366587	1.941618

55	H	6.694254	3.606118	3.034403
56	H	9.205911	0.987107	0.750196
57	C	7.244179	-2.012404	-2.13092
58	H	5.474324	-2.840267	-3.02872
59	H	8.833954	-0.998153	-1.099359
60	H	8.939525	2.807664	2.407298
61	H	7.910427	-2.661175	-2.685611
62	N	4.849961	0.549998	0.074672
63	N	-4.849958	0.549995	-0.074641

Optimized geometry of Ni(4,4'-Czbpy)Cl_2 (neutral, spin triplet). CAM-B3LYP-GD3/6-311+G(d,p).

1	C	1.478758	2.10901	-0.310919
2	C	2.847211	2.005706	-0.547725
3	C	3.41651	0.736811	-0.614587
4	C	2.599813	-0.363868	-0.44846
5	H	1.015091	3.083764	-0.293257
6	H	2.993574	-1.371393	-0.493229
7	C	-0.727921	0.953138	0.115008
8	C	-1.475395	2.103276	0.29341
9	C	-2.84354	1.994878	0.529744
10	H	-1.014498	3.079421	0.280822
11	C	-2.589432	-0.373436	0.418291
12	C	-3.409238	0.724053	0.590115
13	H	-2.980343	-1.382285	0.457937
14	C	0.73454	0.955856	-0.138466
15	N	-1.28605	-0.262549	0.182082
16	N	1.296112	-0.257885	-0.21175
17	Ni	0.007405	-1.802426	-0.01763
18	Cl	-0.223922	-2.48889	-2.101225
19	Cl	0.2411	-2.492644	2.064635

20	N	-3.621336	3.141602	0.706357
21	C	-3.57396	4.2949	-0.090698
22	C	-4.55886	3.349484	1.729626
23	C	-2.846717	4.545258	-1.24877
24	C	-4.482004	5.234812	0.423321
25	C	-4.910349	2.52621	2.79283
26	C	-5.106121	4.634477	1.583349
27	C	-3.019834	5.774794	-1.864747
28	H	-2.180993	3.807171	-1.675376
29	C	-4.640788	6.461904	-0.211875
30	C	-5.851236	3.003538	3.691627
31	H	-4.4627	1.551882	2.933296
32	C	-6.049066	5.092962	2.497261
33	C	-3.901136	6.729455	-1.350864
34	H	-2.463708	5.993802	-2.767972
35	H	-5.339157	7.193003	0.177407
36	C	-6.423014	4.270074	3.545376
37	H	-6.143436	2.379811	4.52744
38	H	-6.477633	6.082376	2.391544
39	H	-4.012393	7.680417	-1.856336
40	H	-7.156857	4.610994	4.264733
41	N	3.621847	3.15549	-0.718284
42	C	3.570252	4.304978	0.083999
43	C	4.558238	3.371658	-1.740898
44	C	2.842397	4.547197	1.243412
45	C	4.474648	5.250642	-0.42586
46	C	4.912585	2.554653	-2.807978
47	C	5.100725	4.658014	-1.588791
48	C	3.01108	5.774489	1.865073
49	H	2.179563	3.804618	1.666715
50	C	4.629059	6.475324	0.215036

51	C	5.851457	3.039679	-3.704774
52	H	4.468611	1.579301	-2.952878
53	C	6.041729	5.124251	-2.500763
54	C	3.8887	6.734801	1.355436
55	H	2.454383	5.987213	2.769447
56	H	5.324603	7.210834	-0.17098
57	C	6.418514	4.307644	-3.552776
58	H	6.145804	2.420923	-4.543522
59	H	6.466647	6.114748	-2.390548
60	H	3.996575	7.683794	1.865324
61	H	7.150899	4.654667	-4.2707
62	H	-4.470398	0.595309	0.747359
63	H	4.478055	0.611893	-0.7723

Optimized geometry of Ni(4,4'-Czbpy)Br$_2$ (neutral, spin triplet). CAM-B3LYP-GD3/6-311+G(d,p).

1	C	1.478194	2.111708	-0.314416
2	C	2.84577	2.007684	-0.555681
3	C	3.412069	0.737932	-0.635832
4	C	2.593921	-0.362453	-0.477609
5	H	1.016885	3.087281	-0.285315
6	H	2.985342	-1.370434	-0.531027
7	C	-0.729423	0.956062	0.10723
8	C	-1.47735	2.105827	0.286086
9	C	-2.844717	1.996872	0.526382
10	H	-1.017241	3.082269	0.270005
11	C	-2.589977	-0.371681	0.41619
12	C	-3.409436	0.725463	0.589454
13	H	-2.980167	-1.380767	0.456084
14	C	0.731656	0.958712	-0.151195
15	N	-1.286777	-0.260021	0.177227

16	N	1.290537	-0.255608	-0.23741
17	Ni	0.002983	-1.797848	-0.039819
18	N	-3.622918	3.143	0.703297
19	C	-3.577758	4.295814	-0.09483
20	C	-4.559122	3.351005	1.727949
21	C	-2.852943	4.545716	-1.254497
22	C	-4.485637	5.235401	0.420009
23	C	-4.908294	2.528413	2.792403
24	C	-5.107675	4.635382	1.581337
25	C	-3.028107	5.774637	-1.871164
26	H	-2.18768	3.807739	-1.682009
27	C	-4.646579	6.461816	-0.215914
28	C	-5.84837	3.005682	3.69212
29	H	-4.459593	1.554628	2.933253
30	C	-6.049559	5.093931	2.496284
31	C	-3.909189	6.729019	-1.356457
32	H	-2.473905	5.993247	-2.77566
33	H	-5.344925	7.192621	0.173945
34	C	-6.42133	4.271623	3.545638
35	H	-6.138875	2.3824	4.528848
36	H	-6.479017	6.082938	2.390403
37	H	-4.022161	7.679458	-1.862527
38	H	-7.154391	4.612583	4.265766
39	N	3.622648	3.157065	-0.716578
40	C	3.57591	4.298694	0.097397
41	C	4.559325	3.380085	-1.73763
42	C	2.850176	4.531857	1.259969
43	C	4.483175	5.246183	-0.403867
44	C	4.910025	2.572571	-2.813078
45	C	5.106516	4.662851	-1.572968
46	C	3.023756	5.752327	1.893637

47	H	2.185407	3.787396	1.676871
48	C	4.642517	6.46387	0.249006
49	C	5.850239	3.063103	-3.705485
50	H	4.462388	1.600383	-2.967655
51	C	6.048561	5.134878	-2.480864
52	C	3.904185	6.714589	1.392675
53	H	2.468809	5.957894	2.800736
54	H	5.340382	7.200664	-0.130304
55	C	6.421855	4.327463	-3.541185
56	H	6.141921	2.451685	-4.550517
57	H	6.476988	6.122742	-2.361057
58	H	4.01591	7.658066	1.911876
59	H	7.155079	4.679014	-4.256044
60	H	-4.47018	0.596217	0.748985
61	H	4.473019	0.612161	-0.796766
62	Br	-0.285742	-2.541141	-2.241801
63	Br	0.293015	-2.5675	2.152961

Optimized geometry of 4,4'-Czbpy (neutral, spin singlet). B3LYP-GD3/6-311+G(d,p).

1	C	1.41649	1.954849	-0.469777
2	C	2.808136	2.007602	-0.400441
3	C	3.478714	0.894373	0.111512
4	C	2.720998	-0.206793	0.505496
5	H	0.863938	2.776888	-0.90313
6	H	3.21947	-1.088407	0.898508
7	C	-0.742623	0.816191	-0.017957
8	C	-1.413279	1.948195	0.451209
9	C	-2.805085	1.996933	0.382161
10	H	-0.863284	2.769844	0.888538
11	C	-2.711058	-0.212744	-0.534542
12	C	-3.472199	0.884114	-0.135177

13	H	-3.206787	-1.093988	-0.93183
14	C	0.749367	0.818487	-0.006142
15	N	-1.377805	-0.262807	-0.491697
16	N	1.387909	-0.260815	0.462359
17	N	-3.497722	3.14257	0.821532
18	C	-3.131263	4.471241	0.549378
19	C	-4.617796	3.157416	1.668179
20	C	-2.112008	4.97079	-0.263345
21	C	-4.028803	5.336631	1.21818
22	C	-5.302223	2.096574	2.262071
23	C	-4.973477	4.502115	1.928085
24	C	-1.98144	6.352564	-0.369339
25	H	-1.444357	4.314941	-0.805345
26	C	-3.882091	6.7202	1.09203
27	C	-6.374242	2.402579	3.095988
28	H	-5.008409	1.068743	2.096491
29	C	-6.053556	4.78391	2.7678
30	C	-2.852292	7.222569	0.304386
31	H	-1.188793	6.758367	-0.987256
32	H	-4.565254	7.390524	1.601344
33	C	-6.754699	3.730433	3.343136
34	H	-6.921839	1.594266	3.566887
35	H	-6.333838	5.811155	2.971847
36	H	-2.723744	8.293675	0.201639
37	H	-7.595797	3.933145	3.995553
38	N	3.497201	3.157516	-0.834218
39	C	3.126597	4.483701	-0.555619
40	C	4.617289	3.179972	-1.68068
41	C	2.105722	4.976107	0.259427
42	C	4.021503	5.355126	-1.220102
43	C	5.305046	2.124171	-2.279684

44	C	4.968814	4.52702	-1.933986
45	C	1.970858	6.356934	0.372159
46	H	1.440054	4.315546	0.798136
47	C	3.870487	6.7376	-1.087206
48	C	6.37618	2.437567	-3.111993
49	H	5.014393	1.094642	-2.119167
50	C	6.048081	4.816261	-2.772213
51	C	2.839067	7.232915	-0.297218
52	H	1.176898	6.757252	0.991964
53	H	4.551606	7.412523	-1.593175
54	C	6.752537	3.767785	-3.352615
55	H	6.926316	1.633265	-3.586784
56	H	6.325188	5.845355	-2.971219
57	H	2.707188	8.303103	-0.189254
58	H	7.593055	3.976288	-4.003955
59	H	-4.549009	0.877724	-0.239621
60	H	4.555534	0.890841	0.215983

Optimized geometry of 4,4'-Czbpy (neutral, spin triplet). B3LYP-GD3/6-311+G(d,p).

1	C	1.434022	2.047346	-0.346518
2	C	2.779947	2.005275	-0.582703
3	C	3.432876	0.741852	-0.64186
4	C	2.626904	-0.417821	-0.479771
5	H	0.938218	3.006749	-0.333267
6	H	3.103657	-1.394528	-0.546318
7	C	-0.684538	0.816453	0.121235
8	C	-1.43126	2.041296	0.327442
9	C	-2.77693	1.993901	0.56407
10	H	-0.93846	3.002296	0.318544
11	C	-2.616434	-0.428182	0.449329
12	C	-3.425893	0.728183	0.617535

13	H	-3.090161	-1.406669	0.511161
14	C	0.691049	0.819222	-0.146389
15	N	-1.333765	-0.423981	0.214537
16	N	1.344107	-0.418734	-0.245647
17	N	-3.540411	3.173492	0.710619
18	C	-3.538523	4.272574	-0.159812
19	C	-4.447435	3.427789	1.744107
20	C	-2.826442	4.469822	-1.343128
21	C	-4.465007	5.231518	0.31652
22	C	-4.745142	2.658474	2.870021
23	C	-5.044285	4.693708	1.529445
24	C	-3.043876	5.655798	-2.038777
25	H	-2.133276	3.72608	-1.713617
26	C	-4.664237	6.414117	-0.398212
27	C	-5.687937	3.159876	3.763497
28	H	-4.252906	1.711712	3.051191
29	C	-5.98183	5.177235	2.443049
30	C	-3.947539	6.62286	-1.572197
31	H	-2.501216	5.833213	-2.960126
32	H	-5.371303	7.155657	-0.043823
33	C	-6.305305	4.402373	3.553398
34	H	-5.940547	2.580896	4.644292
35	H	-6.44598	6.145276	2.29195
36	H	-4.089092	7.537682	-2.135446
37	H	-7.031981	4.765393	4.270619
38	N	3.53982	3.187921	-0.723235
39	C	3.534052	4.282828	0.152427
40	C	4.446589	3.449972	-1.755011
41	C	2.820747	4.47221	1.336291
42	C	4.457784	5.24692	-0.318834
43	C	4.747296	2.686967	-2.884419

44	C	5.03937	4.716707	-1.533996
45	C	3.034115	5.655531	2.037696
46	H	2.129716	3.724555	1.702871
47	C	4.652947	6.426717	0.401626
48	C	5.68899	3.195566	-3.774983
49	H	4.258127	1.739542	-3.070366
50	C	5.975878	5.207513	-2.444778
51	C	3.934991	6.627624	1.576208
52	H	2.490426	5.82686	2.959591
53	H	5.35787	7.172145	0.051149
54	C	6.302361	4.438969	-3.558632
55	H	5.943874	2.621581	-4.658388
56	H	6.436913	6.176271	-2.288815
57	H	4.073388	7.540191	2.143883
58	H	7.028275	4.807672	-4.273721
59	H	-4.496546	0.652731	0.748285
60	H	4.503813	0.670348	-0.772503

Optimized geometry of 5,5'-Czbpy (neutral, spin singlet). B3LYP-GD3/6-311+G(d,p).

1	C	-1.409623	0.001214	-1.266753
2	C	-2.795783	0.007923	-1.283097
3	C	-3.486656	-0.008361	-0.069687
4	C	-2.736312	-0.027658	1.111469
5	H	-0.833244	0.01579	-2.181484
6	H	-3.344558	0.034261	-2.216888
7	H	-3.244639	-0.052622	2.070581
8	C	0.742376	-0.006903	0.033783
9	C	1.409623	0.001214	1.266753
10	C	2.795783	0.007923	1.283097
11	H	0.833244	0.01579	2.181484
12	C	2.736312	-0.027658	-1.111469

13	C	3.486656	-0.008361	0.069687
14	H	3.344558	0.034261	2.216888
15	H	3.244639	-0.052622	-2.07058
16	C	-0.742376	-0.006903	-0.033783
17	N	1.407677	-0.017234	-1.132991
18	N	-1.407677	-0.017234	1.132991
19	C	5.737749	-0.908542	0.688888
20	C	5.694533	0.906051	-0.678061
21	C	5.40794	-2.008061	1.481362
22	C	7.083781	-0.572218	0.406859
23	C	5.314999	2.003396	-1.451014
24	C	7.056231	0.583756	-0.462676
25	C	6.453132	-2.761311	2.008497
26	H	4.376669	-2.274424	1.675154
27	C	8.114724	-1.344723	0.947719
28	C	6.325284	2.767919	-2.027324
29	H	4.272776	2.257848	-1.595951
30	C	8.051537	1.367199	-1.052506
31	C	7.793773	-2.433766	1.750503
32	H	6.223991	-3.620584	2.628361
33	H	9.149933	-1.098812	0.739318
34	C	7.680401	2.453913	-1.836389
35	H	6.056998	3.625083	-2.634249
36	H	9.098148	1.131339	-0.895874
37	H	8.583334	-3.040743	2.177656
38	H	8.441667	3.069346	-2.301154
39	C	-5.737749	-0.908542	-0.688888
40	C	-5.694533	0.906051	0.678061
41	C	-5.40794	-2.008061	-1.481362
42	C	-7.083781	-0.572218	-0.406859
43	C	-5.315	2.003396	1.451014

44	C	-7.056231	0.583756	0.462676
45	C	-6.453132	-2.761311	-2.008497
46	H	-4.376669	-2.274424	-1.675154
47	C	-8.114724	-1.344722	-0.947719
48	C	-6.325284	2.767919	2.027324
49	H	-4.272776	2.257847	1.595951
50	C	-8.051537	1.367199	1.052506
51	C	-7.793773	-2.433766	-1.750503
52	H	-6.223991	-3.620584	-2.628361
53	H	-9.149933	-1.098812	-0.739319
54	C	-7.680401	2.453913	1.836389
55	H	-6.056998	3.625083	2.634249
56	H	-9.098148	1.131339	0.895874
57	H	-8.583334	-3.040743	-2.177656
58	H	-8.441667	3.069346	2.301153
59	N	-4.898135	-0.006403	-0.024911
60	N	4.898135	-0.006403	0.024911

Optimized geometry of 5,5'-Czbpy (neutral, spin triplet). B3LYP-GD3/6-311+G(d,p).

1	C	-1.406579	-0.13371	-1.294973
2	C	-2.768715	-0.095952	-1.314467
3	C	-3.474645	-0.045067	-0.079566
4	C	-2.710122	-0.070656	1.123498
5	H	-0.826249	-0.141022	-2.206713
6	H	-3.315328	-0.067674	-2.249128
7	H	-3.240219	-0.088619	2.073608
8	C	0.698704	-0.130649	0.031052
9	C	1.406579	-0.133697	1.294974
10	C	2.768716	-0.095939	1.314466
11	H	0.826249	-0.141002	2.206713
12	C	2.710122	-0.070662	-1.123498

13	C	3.474644	-0.045064	0.079566
14	H	3.315328	-0.067654	2.249128
15	H	3.240219	-0.08863	-2.073608
16	C	-0.698704	-0.130651	-0.031051
17	N	1.408038	-0.11884	-1.172648
18	N	-1.408038	-0.118836	1.172649
19	C	5.747752	-0.832138	0.753504
20	C	5.644011	0.870712	-0.74871
21	C	5.454779	-1.891695	1.61253
22	C	7.081311	-0.482185	0.434296
23	C	5.228672	1.910648	-1.580979
24	C	7.01526	0.600682	-0.525911
25	C	6.525385	-2.575562	2.182621
26	H	4.43411	-2.183838	1.820393
27	C	8.137454	-1.184765	1.013946
28	C	6.213558	2.655176	-2.224556
29	H	4.180292	2.142383	-1.714846
30	C	7.984058	1.363408	-1.177494
31	C	7.852953	-2.225002	1.894817
32	H	6.325489	-3.400484	2.85662
33	H	9.163479	-0.929103	0.774768
34	C	7.576636	2.383722	-2.033143
35	H	5.917752	3.465805	-2.880337
36	H	9.037813	1.169039	-1.012848
37	H	8.663163	-2.778495	2.354661
38	H	8.31798	2.983289	-2.548091
39	C	-5.747753	-0.832141	-0.753501
40	C	-5.64401	0.870715	0.748706
41	C	-5.454781	-1.891703	-1.612521
42	C	-7.081311	-0.482186	-0.434294
43	C	-5.22867	1.910656	1.58097

44	C	-7.015259	0.600685	0.525909
45	C	-6.525389	-2.575572	-2.182608
46	H	-4.434113	-2.183849	-1.820382
47	C	-8.137456	-1.184767	-1.01394
48	C	-6.213555	2.655187	2.224543
49	H	-4.18029	2.142391	1.714834
50	C	-7.984056	1.363415	1.177487
51	C	-7.852956	-2.225009	-1.894806
52	H	-6.325494	-3.400497	-2.856603
53	H	-9.16348	-0.929103	-0.774762
54	C	-7.576633	2.383734	2.033132
55	H	-5.917748	3.46582	2.88032
56	H	-9.037811	1.169047	1.012842
57	H	-8.663167	-2.778503	-2.354647
58	H	-8.317976	2.983304	2.548077
59	N	-4.872963	-0.005907	-0.030425
60	N	4.872964	-0.005906	0.030425
